# qcGEM: A Graph‐Based Molecular Representation With Quantum Chemistry Awareness

**DOI:** 10.1002/advs.77997

**Published:** 2026-10-18

**Authors:** Haoyu Wang, Ziyan Zhang, Swee Hong Ng, Haipeng Gong

**Affiliations:** ^1^ MOE Key Laboratory of Bioinformatics, School of Life Sciences Tsinghua University Beijing China; ^2^ State Key Laboratory of Green Manufacturing Tsinghua University Beijing China; ^3^ Center of Life Sciences and Artificial Intelligence, School of Life Sciences Tsinghua University Beijing China; ^4^ Beijing Frontier Research Center for Biological Structure Tsinghua University Beijing China

**Keywords:** drug discovery, quantum chemical information, molecular embedding, representation learning, physics‐inspired representation

## Abstract

The advancement of artificial intelligence (AI) has reshaped drug discovery. AI‐based models typically rely on molecular representations for prediction. However, the absence of physically grounded information in mainstream molecular representations not only limits the model performance in practical applications but also hinders mechanistic understanding and exploitation by humans. To overcome this issue, we introduce qcGEM, a quantum‐chemistry‐aware graph‐based embedding of molecules that incorporates physical priors into molecular representation learning. Through a specially designed model architecture to adequately process quantum chemistry information, qcGEM provides a compact, physics‐informed molecular representation that supports a diverse range of downstream applications. Particularly, qcGEM demonstrates state‐of‐the‐art performance across a broad range of molecule‐related benchmarks, as evidenced by comprehensive evaluations on 83 tasks including molecular property prediction, activity cliff detection, protein–ligand interaction modeling, and opioid drug regression, and simultaneously offers strong interpretability at multiple representation levels. We additionally propose a simplified variant, qcGEM‐Hybrid, with substantially accelerated embedding generation and robust performance. Overall, our method provides an advanced molecular representation that will benefit molecule‐related modeling and prediction, supporting further progress in AI‐aided drug discovery.

## Graphical abstract

In this work, we introduce qcGEM, a quantum‐chemistry‐aware graph‐based embedding of molecules that incorporates physical priors into molecular representation learning. Through a specially designed model architecture to adequately process quantum chemistry information as well as three‐dimensional molecular geometry, qcGEM provides a compact, physics‐informed representation of chemical molecules, which supports a diverse range of downstream applications in drug discovery.

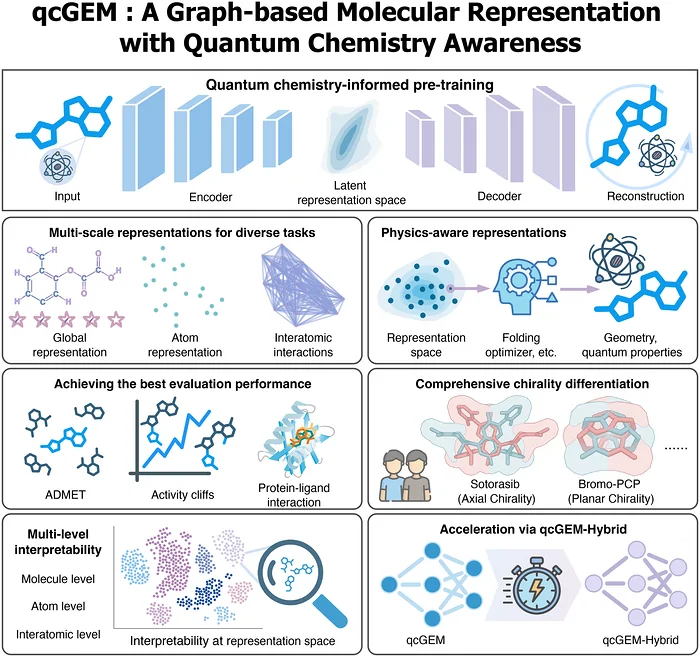

## 1 Introduction

Drug molecules exert therapeutic effects by modulating the activity of macromolecular targets. Therefore, drug discovery typically begins with target identification, followed by exploring the large chemical space for potential binders [1]. Yet, most early hits fail during development due to assay artifacts, promiscuous reactivity, unacceptable toxicity, poor pharmacokinetics, and/or inadequate potency [2]. Consequently, early triage strategies that incorporate physicochemical properties, ADME (i.e., absorption, distribution, metabolism, and excretion) profiles, safety assessments, as well as binding‐mode predictions are essential for enriching truly drug‐like candidates [3]. Driven by advances in deep learning, numerous computational approaches have emerged for filtering drug candidates, enormously accelerating the entire process [4, 5, 6, 7, 8, 9, 10]. Compared to traditional physics‐based approaches [11, 12, 13], deep learning models offer markedly faster inference once training is complete and support more flexible and more richly defined prediction tasks dictated by the available training labels.

Since deep learning models are fundamentally data‐driven, their performance depends critically on the quality and relevance of input data and labels [14]. In the past, numerous molecular representations have been developed and employed as input for deep learning models in the field of drug discovery, which generally fall into two categories: handcrafted features and embeddings learned from neural networks. SMILES [15] and fingerprints [16] encode a molecule as a linear text string defined by explicit syntax rules, yielding a compact 1D representation that inherently fits in large language models and thus holding a ubiquitous status in molecular machine learning [5, 17]. Based on advances in computer vision, several studies convert molecules into images and feed them to convolution‐based models for downstream property prediction [6, 7]. In addition, molecules are often naturally represented as atom‐bond graphs and processed by a range of graph neural network (GNN) architectures [8, 9, 10]. Albeit widely used, these handcrafted representations usually suffer from information redundancy, non‐unique representations, insufficient structural context, and slow convergence at downstream training. In recent years, an increasing number of deep‐learning‐based molecular representation approaches have been proposed [18, 19, 20, 21, 22, 23, 24, 25, 26, 27, 28, 29, 30, 31, 32, 33], which reportedly achieve superior performance over traditional methods in various applications. These methods automatically distill higher‐order semantic information from the pre‐trained model and transfer with outstanding accuracy across downstream tasks. However, their performance is highly contingent on both the volume and quality of pre‐training data, as well as on the design of pre‐training strategy [34, 35]. Despite the progress, rudimentary learning features and simplistic self‐supervised frameworks in these methods result in high data demands and representational dimensionality, limited generalizability, incomplete structure encoding, and, more importantly, a lack of physical interpretability.

Compared to conventional learning objectives, quantum chemical calculations offer more physically grounded targets for the pre‐training process [36, 37]. By solving the Schrödinger equation, complete quantum information of electrons can be obtained, which prescribes interactions between nuclei, including non‐local internuclear communications that are of critical importance in conjugate molecular systems but are often neglected by traditional methods [38]. In principle, electronic wave functions contain full quantum information for a specified configuration of atomic nuclei. However, deficits like the high dimensionality, numerical instability, and continuous distribution hinder their direct integration with deep learning models [39]. The electron density, a distribution function directly defined in the three‐dimensional Cartesian space, contains equivalent quantum information with substantially reduced dimensionality. However, the continuous nature and the lack of rotational invariance jointly hinder its effective modeling in mainstream network architectures [40, 41]. Localization of wave functions, as a further simplification approach, provides a separation of electronic orbitals into those residing on atomic centers and those falling in‐between [42, 43], perfectly aligning with the node and edge inputs of GNNs. Additionally, the corresponding quantum descriptors also provide informative learning objectives to guide the model in generating more compact and physically meaningful molecular representations. In a prior work, we have constructed a quantum chemically annotated database qcMol [44], which incorporates abundant quantum chemical descriptors and three‐dimensional molecular configurations for over 1.2 million chemical compounds, laying the foundation for quantum‐chemistry‐based molecular representation learning.

In this study, we propose qcGEM, a quantum‐chemistry‐aware g raph‐based embedding of molecules. Through pre‐training over a subset of molecules in the qcMol database, qcGEM learns to encode electronic quantum information and molecular structural features into a compact graph that preserves rich physicochemical and complete geometrical details. At the architectural level, the global‐node‐edge hierarchy in the encoder–decoder framework of our method enables the generation of a multiscale molecular embedding applicable for diverse prediction and molecular design tasks. To validate the advantage of our representation, we compared it with 32 widely adopted molecular embeddings. Under the unified frozen‐representation + probe network protocol, qcGEM comprehensively achieves the state‐of‐the‐art performance on 83 datasets covering versatile molecular property and protein–ligand related tasks. Subsequently, we developed a simplified variant, qcGEM‐Hybrid, by taking the trade‐off between accuracy and speed. Allowing enhanced batch embedding generation at an acceptable speed for practical applications, this hybrid version maintains a comparably robust performance in downstream tasks. Finally, our analysis shows that the representation space of qcGEM is highly interpretable, presenting a promising potential for molecular design.

## 2 Results

### 2.1 Overview of Framework

As shown in Figure 1a, the qcGEM framework generates representations for drug molecules in three steps: (1) searching from the qcMol library for rapid quantum chemical annotation, (2) pre‐training an encoder–decoder model to learn a graph‐based representation space from initial features, and (3) transferring the representation to practical molecules for downstream applications with frozen or fine‐tuned settings. Viewing a chemical molecule as a system composed of atomic nuclei that interact through the potential energy enforced by electrons, qcGEM substitutes traditional sparse graph representations with a fully connected graph to better capture all pairwise atomic interactions. Through specialized designs in model architecture (Figure 1b), information exchange (Figure 1c), and pre‐training strategy, qcGEM constructs a multi‐level molecular representation (Figure 1d) that delivers robust generalization across diverse tasks while preserving strong physical interpretability.

**FIGURE 1:**
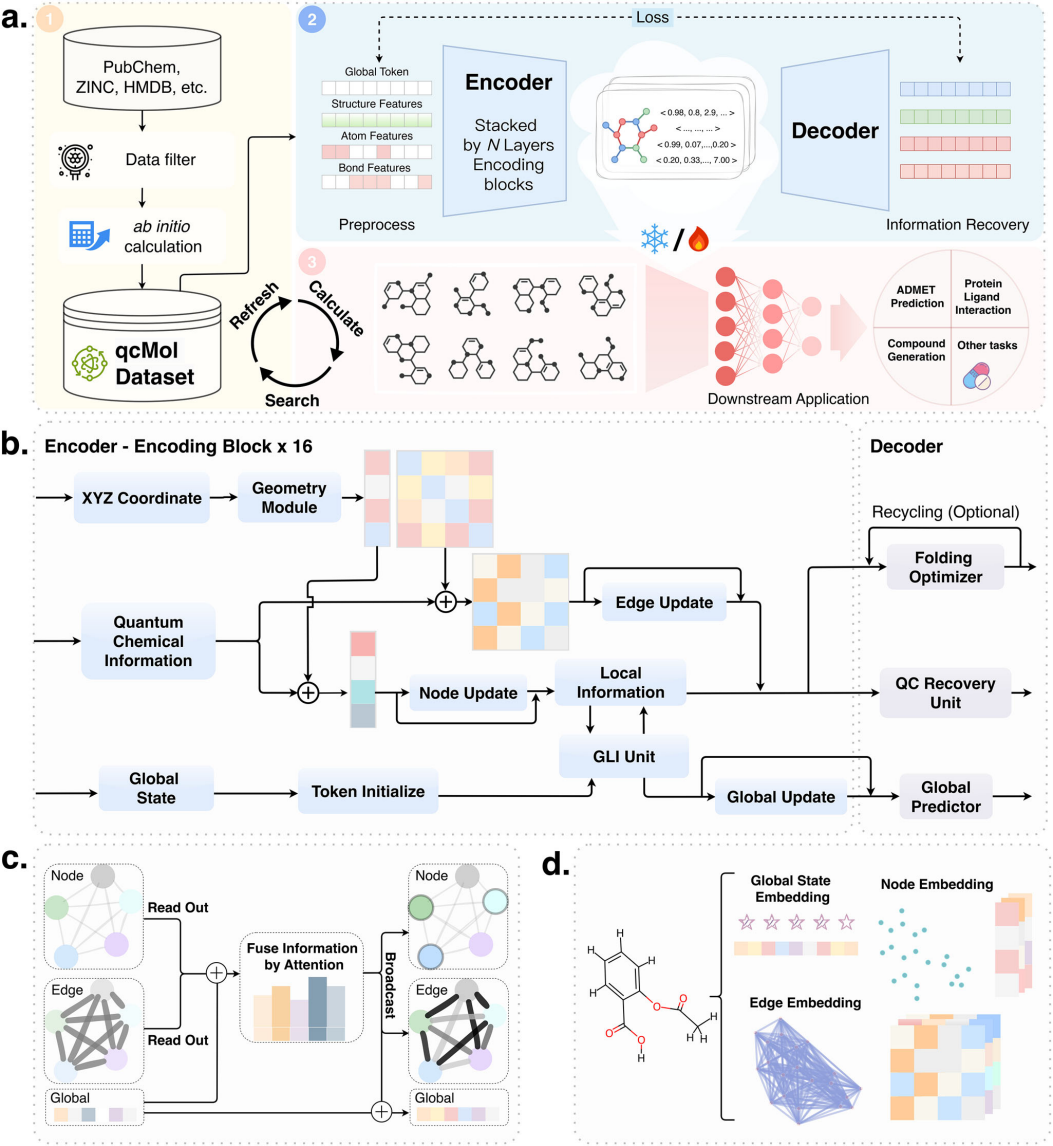
The model architecture of qcGEM. (a) Schematic overview of the qcGEM framework. The compound is first queried from the qcMol database or computed via quantum chemical calculations. The qcGEM model encodes the initial features into a unified molecular representation, from which all input information could be reconstructed after decoding. The encoded representation is finally transferred to downstream tasks for diverse applications. (b) The encoder–decoder architecture of the qcGEM model. The encoder consists of 16 repeated encoding blocks, and allows sufficient information exchange between the structural, quantum chemical, and global features. The decoder is composed of a folding optimizer, a QC recovery unit and a global predictor, for the reconstruction of three‐dimensional configuration, quantum chemical descriptors and global features, respectively. (c) Model design of the GLI unit in panel (b) for effective representation learning of molecular information at the global, node, and edge levels. (d) Schematic showing the compact and multi‐level graph representation generated by qcGEM for an exemplar molecule.

### 2.2 Model Architecture and Pre‐Training Strategy

The model architecture of qcGEM consists of an encoder and a decoder (Figure 1b). The encoder employs stacked encoding blocks to embed quantum chemical information, three‐dimensional molecular structure, and other global properties into a compact, multi‐level representation. The decoder leverages this learned representation to reconstruct molecular structure, quantum descriptors, and global characteristics via the folding optimizer, the QC recovery unit, and the global predictor, respectively. The information flow in qcGEM is consistent with the physical intuition behind quantum chemical features, allowing iterative exchanges between information of various sources. First, the geometric information of atomic nuclei is encoded into nodes and edges from the complete molecular structure in a roto‐translation‐invariant manner. Next, these structure‐aware features are fused into the localized electronic quantum information for node and edge updating. Finally, the global state and local node‐edge information are exchanged in the global–local interaction (GLI) unit (Figure 1c) to produce a condensed global representation for the molecule. These designs enable efficient multi‐scale learning and fast convergence, yielding a compact representation with quantum chemistry awareness.

The pre‐training tasks include geometry denoising, electronic feature recovery and correction, and global state condensation. For geometry denoising, qcGEM is specially designed with a geometry module for complete geometry encoding, a folding optimizer for effective structure decoding (Figure S1a), and a tailored FAPE loss [45] for constraining the structural reconstruction of small molecules (Figure S1b). To capture electronic quantum information, qcGEM employs corruption and masking of node and edge features, followed by a correction and reconstruction process. The global state condensation enhances the permutation‐invariant readout capability of graph embedding by feature decoupling [33, 46]. These components jointly endow qcGEM the capacity to capture high‐level semantics, while supporting multi‐level molecular prediction and design at the same time.

qcGEM is pre‐trained on only 300 thousand samples, requiring substantially fewer resources than many recently proposed methods (Table S5), and supports applications in both parameter freezing and rapid fine‐tuning settings. The fixed representations generally achieve sufficiently strong performance, and require only a single‐pass inference for repeated uses. Pre‐calculated representations are available for download through the qcMol database, while the embeddings for unseen molecules could be computed from scratch from the online server of qcGEM.

### 2.3 Benchmark on Molecular Property Prediction With qcGEM

To evaluate the performance of qcGEM, we compared it against mainstream handcrafted features and model‐based embeddings (Table S5) across a series of downstream tasks using a probe network of the multiple‐layer perceptron (MLP) architecture (Figure 2a). We first tested on molecular property prediction tasks, involving 30 widely used benchmarks collected from MoleculeNet [47] and the TDC dataset [48] as well as 11 benchmarks from the recently reported PharmaBench dataset [49]. These datasets cover molecular properties of multiple aspects, and are categorized into prediction tasks of absorption (9 sets), distribution (7 sets), metabolism (12 sets), excretion (6 sets), and toxicity (7 sets), which are abbreviated in conglomerate as ADMET properties (Table S6).

**FIGURE 2:**
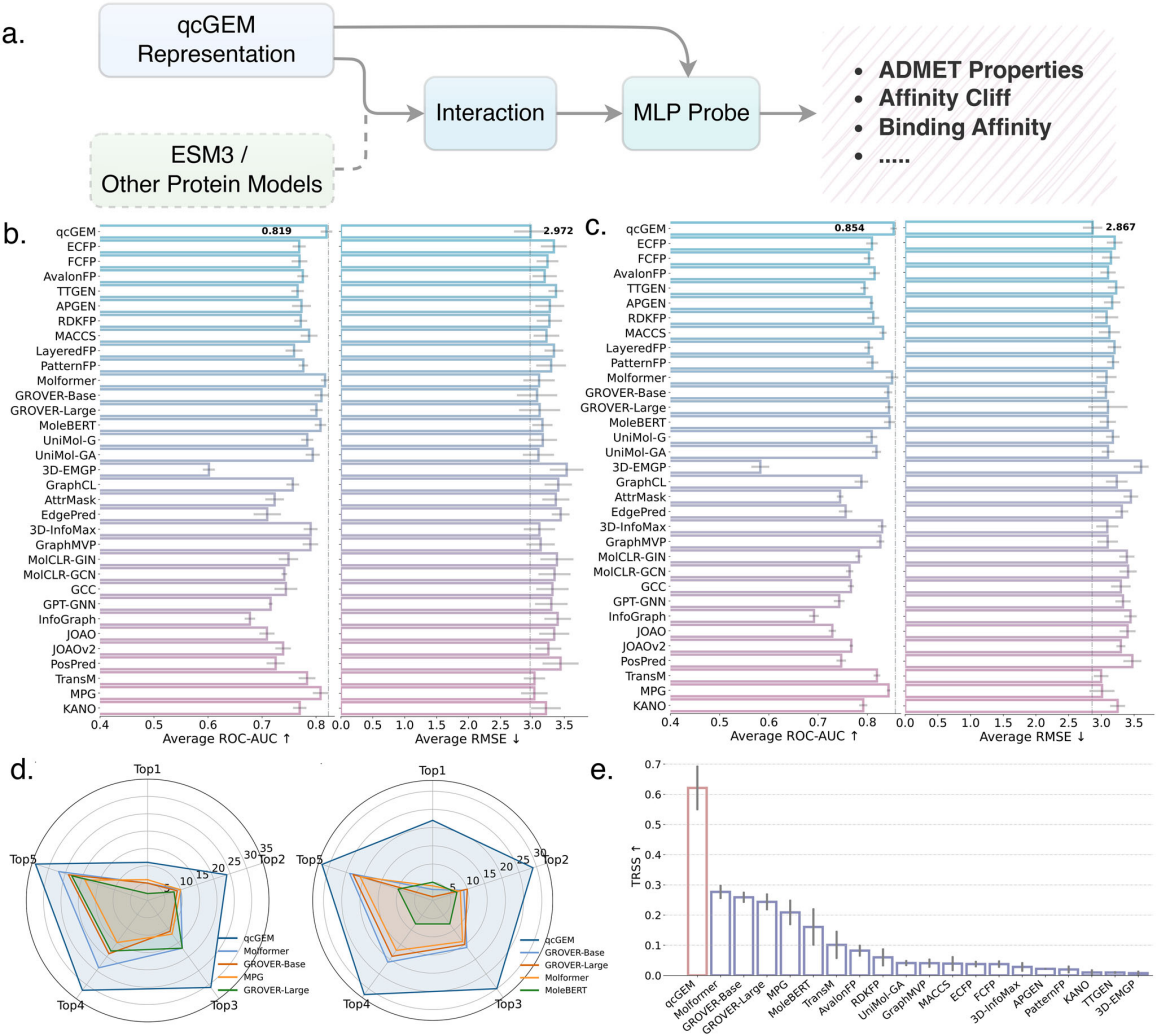
Performance of qcGEM on molecular properties prediction. (a) In downstream evaluations, qcGEM and/or rival representations are directly utilized for prediction through an MLP probe network for compound‐only tasks, but are first integrated with protein representation models via an interaction module for protein‐related tasks. (b) Comparison of the 33 molecular representations on 41 molecular property prediction tasks in the scaffold split setting. ROC‐AUC is shown for classification tasks (left), while RMSE is reported for regression tasks (right), with arrows indicating the desired directions. Vertical dash‐dotted lines indicate the best‐performing results. (c) Comparison of the 33 molecular representations on 41 molecular property prediction tasks in the random split setting. The results are presented in the same convention as in panel (b). (d) Radar plots of the overall performance profiles for the top methods in the combination of random and scaffold split settings. Here, occurring counts of each method in the top 1–5 rankings are shown for the regression tasks (left) and classification tasks (right), respectively. (e) The overall TRSS scores of the top methods. Here, TRSS scores are calculated across split settings and task categories, and their mean values and standard errors are shown in the bar plot.

The evaluation was performed in both the standard random split setting (i.e., random splitting of training and testing data) and the more challenging scaffold split setting (i.e., scaffold‐based splitting of training and testing data), using the metric of root mean square error (RMSE) for regression tasks and area under curve (AUC) of the receiver operating characteristic (ROC) curve for classification tasks. As shown in Figure 2b,c, qcGEM achieves the state‐of‐the‐art performance in both regression and classification tasks across both split settings. Particularly, our representation evidently outperforms the other methods in regression tasks. Its advantage in classification tasks is slightly weakened, likely due to the diminished assistance of high‐precision numerical quantum features in the prediction of categorical labels (see our systematic investigation of this effect in Section S4.1.3). Detailed ranking information of qcGEM in all individual tasks could be found in Figure S8. Furthermore, qcGEM exhibits a robust, strong performance across the 5 ADMET task categories, consistently achieving the top 3 ranking for more than half of the tasks in each category (Figure S9a,b). We further summarize the occurring counts of tested methods in the top k (k=1,2,3,4,5) rankings for regression and classification tasks, respectively (Figure S10). The radar plots in Figure 2d clearly demonstrate the comprehensive superiority of qcGEM in this respect (see Figure S9c,d for the separate plots for individual data splits). In this study, we propose the top‐ranking stability score (TRSS), a metric that mitigates the impact of occasional outliers by rewarding consistent top‐tier performances (Equation 8), to comprehensively evaluate the performance and robustness of tested methods. Figure 2e presents the distribution of TRSS scores across four evaluation scenarios (two split settings and two task types). Again, qcGEM markedly surpasses the other methods in this metric.

Hence, results on the total of 41 ADMET datasets demonstrate that qcGEM offers a powerful molecular representation, leading to strong performance in molecular property prediction. Detailed per dataset results could be found in Tables S10–S27.

### 2.4 Benchmark on Activity Cliff Prediction With qcGEM

In drug screening, subtle structural modifications can lead to substantial changes in binding affinity [50, 51], as exemplified by the activity cliff molecules. To assess qcGEM's sensitivity to such variations, we curated 30 tasks from the MoleculeACE dataset [52], which incorporates experimental cases where minor structural changes result in pronounced differences in bioactivity (Table S7). Following the convention of MoleculeACE, all ligands are categorized into common molecules and activity cliff molecules to evaluate the accuracy and generalization of different molecular representations.

As shown in Figure 3a, qcGEM on average achieves the lowest prediction error (in RMSE) across the 30 datasets, regardless of whether the evaluation considers all molecules or only focuses on activity cliff compounds. To assess performance across task types, we further categorized all testing data into 30 prediction datasets, 6 protein classes and 2 experimental categories, respectively (Table S7). qcGEM exhibits strong performance across all task subdivisions among the 33 molecular representations, achieving the highest ranking in 40%, 66%, and 100% of the corresponding task categories, respectively, for all tested molecules (Figure 3d). This superiority persists when the focus is shifted to activity cliff molecules (Figure S11a). Moreover, either for all molecules or activity cliff molecules, qcGEM maintains a consistent advantage over the other methods in respect of the occurring counts within the top 1–5 rankings (Figure S11b; see Figure S12 for detailed statistics). We further calculated the TRSS score across all task types. As expected, qcGEM achieves the highest overall score, indicating its superior robustness (Figure 3c).

**FIGURE 3:**
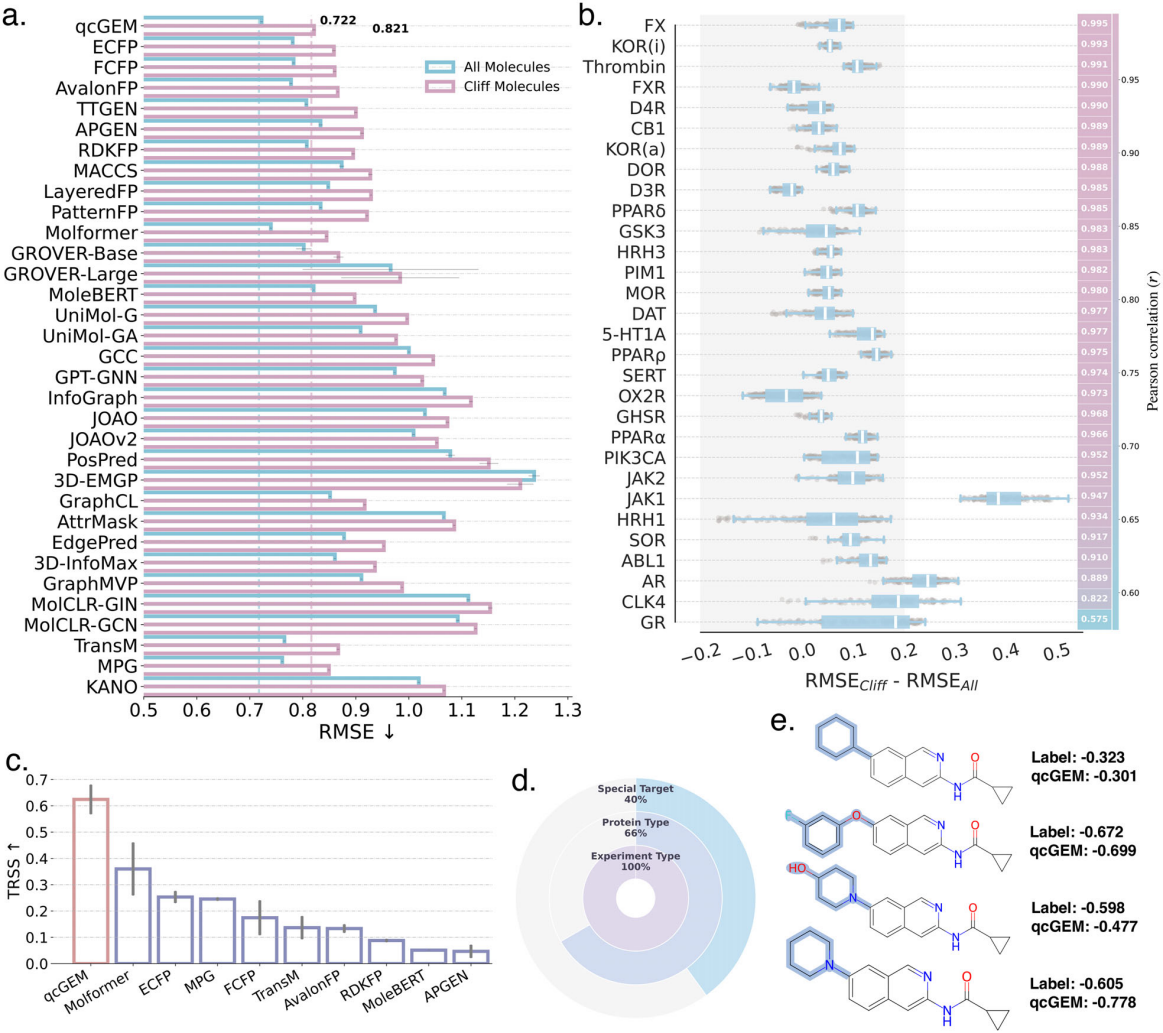
Performance of qcGEM in capturing subtle structural variations. (a) Comparison of prediction errors for all molecules (blue) and activity cliff molecules (pink) across 33 molecular representation methods. The prediction error is evaluated by RMSE, with the arrow aside indicating the desired direction. Vertical dash‐dotted lines indicate the best‐performing results. (b) The box plot (left) shows the distribution of differences in prediction errors (using qcGEM) between activity cliff molecules and all molecules (i.e., ${\text{RMSE}}_{\text{Cliff}}-{\text{RMSE}}_{\text{All}}$) across 30 datasets, where box limits correspond to quantiles with whiskers extending to 1.5 inter‐quartile range. The heatmap (right) illustrates the Pearson correlation coefficient r between inference values of activity cliff molecules and all molecules during the training of MLP module. (c) The overall TRSS scores of the top methods. Here, TRSS scores are calculated with five random seeds across activity cliff molecules and all molecules, respectively, and their mean values and standard errors are shown in the bar plot. (d) The multi‐layer pie chart illustrates proportions of qcGEM achieving the best ranking in 30 tasks under three task classification criteria: individual prediction tasks (outer), protein classes (middle), and experimental categories (inner). (e) An example of the identification of activity cliff molecules for the protein kinase ABL1. For each ligand, the difference in chemical structure is highlighted in blue shadow, while the ground‐truth binding affinity and prediction based on qcGEM are listed aside.

Following the MoleculeACE [52] work, we evaluated the difference of prediction error between activity cliff molecules and all molecules to assess the generalizability of qcGEM on bioactivity prediction in the presence of activity cliff molecules. In most protein targets, prediction errors of the two sets of molecules only exhibit a minor deviation, falling between −0.2 and 0.2, laterally indicating good generalization (Figure 3b, left; Figure S13a). Consistently, during the optimization of MLP parameters (see Figure 2a), models trained on activity cliff molecules and all molecules present a strong Pearson correlation coefficient for most protein targets, precluding biased training (Figure 3b, right; Figure S13b). In addition, qcGEM also demonstrates consistent performance across different protein types (Figure S14), achieving the leading position in most types. Furthermore, qcGEM effectively captures the minor structural modifications that lead to notable changes in bioactivity. In case studies on protein targets of various classes, despite the high similarity among candidate ligands, qcGEM successfully distinguishes them and yields reliable predictions on binding affinity (Figure 3e and Figure S15). More detailed analysis on the contribution of individual quantum features in the activity cliff prediction through qcGEM embeddings could be found in Section S4.1.2.

Besides the above conventional analyses that focus either on general model performance on all versus activity cliff compounds or on case studies, we specifically set up a pair‐level evaluation to systematically compare qcGEM and all baseline models on the power to recognize an authentic activity cliff pair (i.e., two compounds with similar structures but different bioactivities). As described in Section S4.4, qcGEM maintains its superiority in this aspect.

Overall, these results demonstrate the capacity of qcGEM in effectively capturing the subtle structural variations within chemical compounds. The strong robustness and generalization across diverse application scenarios underscore its potential in high‐throughput screening of active drug candidates. Detailed per‐dataset results could be found in Tables S28–S39.

### 2.5 Benchmark on Protein–Ligand Binding Prediction With qcGEM

To comprehensively assess the representational capability of qcGEM, we examined it against the other methods on 11 protein–ligand interaction prediction tasks. When ESM‐3 was required, outputs from the protein language model ESM‐3 [53] were consistently adopted to supplement each molecular representation for probe‐network‐based evaluation, ensuring a fair comparison across all methods (Figure 2a). The datasets [54, 55, 56, 57, 58] cover five general protein–ligand interaction tasks (Table S8) and six opioid‐type tasks (Table S9).

In the general protein–ligand interaction prediction, qcGEM attains the second‐best average rank across the five tasks among the 33 evaluated representations. It ranks 3rd on the 2 regression tasks (Figure 4a), with RMSE within 0.001–0.005 of the best entries (the DAVIS gap lies within the seed error). Although ranking 5th on the 3 classification tasks (Figure S16b), its ROC‐AUC is within 0.01 of the best‐performing method on each individual task. Notably, the classification tasks are close to saturation: 9, 7, and 15 out of the 33 methods fall within 0.01 ROC‐AUC of the top score on Human, *C. elegans* and BindingDB, respectively, with nominal rankings separated only at the third decimal place. In addition, the four methods having higher mean ROC‐AUC than qcGEM were pre‐trained on 11 million to 1.1 billion molecules, 36.7 to 3700 times more than the 300 thousand compounds used by qcGEM (Table S5). Although pre‐training scale alone does not determine performance, qcGEM outperforms 6 of the 10 methods pre‐trained on larger corpora and ranks 1st among the 14 methods pre‐trained on 300 thousand molecules or fewer. Moreover, qcGEM‐identified ligands often present physically plausible interaction patterns with the target protein (Figure 4e and Figure S17).

**FIGURE 4:**
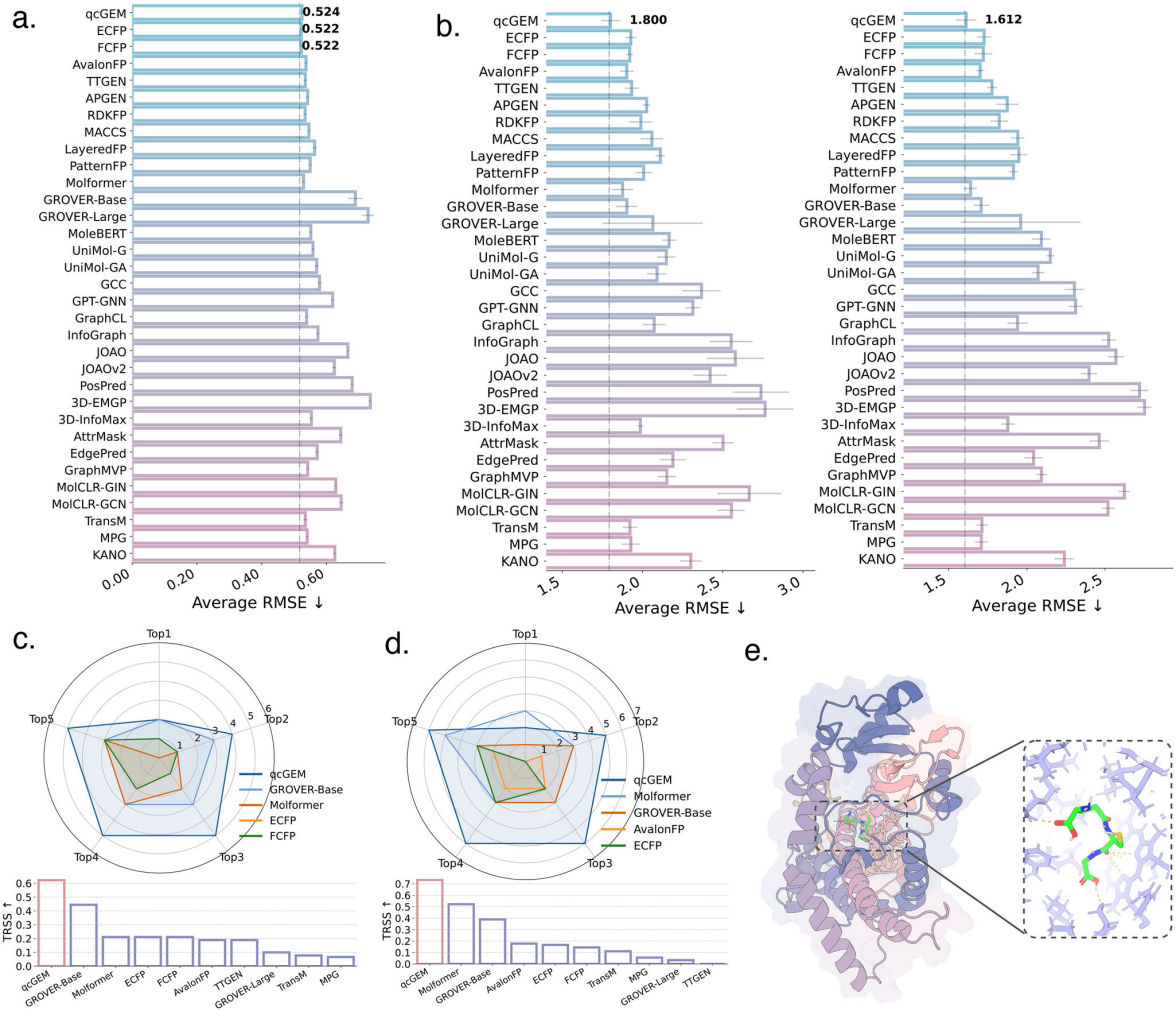
Performance of qcGEM on protein‐ligand prediction. (a) Performance comparison of qcGEM and other methods on the regression tasks of general protein–ligand interaction prediction. Arrows indicate the desired directions. Vertical dash‐dotted lines indicate the best‐performing results. (b) Performance comparison of qcGEM and other methods on the opioid‐type molecule–receptor interaction prediction tasks in the scaffold (left) and random (right) split setting. The prediction error is evaluated by RMSE, with the arrow indicating the desired direction. The vertical dash‐dotted line indicates the best‐performing result. (c) Ranking summary of the opioid‐type molecule–receptor interaction prediction tasks under the scaffold split. The radar plot (upper) shows, for each method, the number of tasks in which it ranks within the top 1 to top 5, and the bar plot (lower) presents the corresponding TRSS scores. (d) The same tasks and plotting convention as in panel (c), but under the random split. (e) The docking result of the predicted positive ligand for an exemplar protein target. The inset shows the atomic‐level interaction details, with the ligand colored by elemental types and protein side‐chains colored in purple.

We then evaluated qcGEM on opioid–receptor interaction prediction. Unlike previous tasks involving diverse interaction types, this setting focuses exclusively on interactions with six opioid receptor subtypes. This time, qcGEM not only achieves the best overall performance in both the scaffold and random split settings (Figure 4b), but also demonstrates strong robustness in ranking (Figure 4c,d; see Figure S16d,e for the detailed statistics). These results support the strong generalizability of qcGEM to domain‐specific scenarios.

In addition to the above tests, we further benchmarked qcGEM on the more challenging CASF‐2016 dataset [59], which is specially designed to evaluate score functions on protein–ligand binding affinity given the knowledge of fine‐grained interaction interface. To fully leverage the spatial information, we extracted edge‐level features from the well‐performed protein structure predictor MiniFold [60] to supplement the original protein features encoded by ESM‐3, and limited the comparison to other graph‐based embeddings (using the same protein features). Results in Figure S18 demonstrate the superiority of qcGEM in this regression task.

These results confirm that qcGEM can be effectively integrated with external models to deliver stable performance across a wide range of protein–ligand related tasks. In practice, the protein encoder (in Figure 2a) can be easily replaced with other high‐quality backbones for seamless adaptation to task‐specific applications. Detailed per dataset results could be found in Tables S40–S45.

### 2.6 Scalable and Robust Performance of qcGEM‐Hybrid

Previous evaluations have demonstrated that qcGEM abstracts essential molecular information into a compact form through pre‐training and achieves strong performance across a broad spectrum of downstream tasks. However, the standard qcGEM relies on the full B3LYP‐D3/def2‐SV(P) pipeline for geometric optimization of candidate molecules, which is slow and thus hinders efficient feature derivation for molecules without pre‐calculated quantum chemical annotations. We attempted to accelerate the computational process by leveraging the semiempirical GFN2‐xTB [61] model to achieve more rapid, albeit less accurate, geometric optimization, under the assumption that qcGEM has learned to handle suboptimal structures through the pre‐training task of geometry denoising (Figure S4). To this end, we proposed a hybrid variant qcGEM‐Hybrid, by simply replacing the full B3LYP‐D3/def2‐SV(P) quantum chemistry calculation with B3LYP‐D3/def2‐SV(P)//GFN2‐xTB while keeping the remainder of the pipeline unchanged.

In terms of quantum feature calculation, qcGEM‐Hybrid offers substantial time savings over qcGEM (Figure 5a). Its advantage becomes more pronounced with the increase of molecular size, as evidenced by the reduction of power‐law scaling exponent from 2.58 to 1.76. Specifically, for a large molecule of 100 atoms, the time consumption of quantum chemistry calculation on a single CPU core drops from 18 h to 28 min, and will be further reduced to within 2 min if 16 CPU cores are employed (Figure 5b). Considering the nearly negligible time for model inference by the encoder, the overall computational efficiency of qcGEM‐Hybrid is fully acceptable in practical embedding generation for unseen molecules (see Section S4.2.1 for the end‐to‐end runtime evaluation).

**FIGURE 5:**
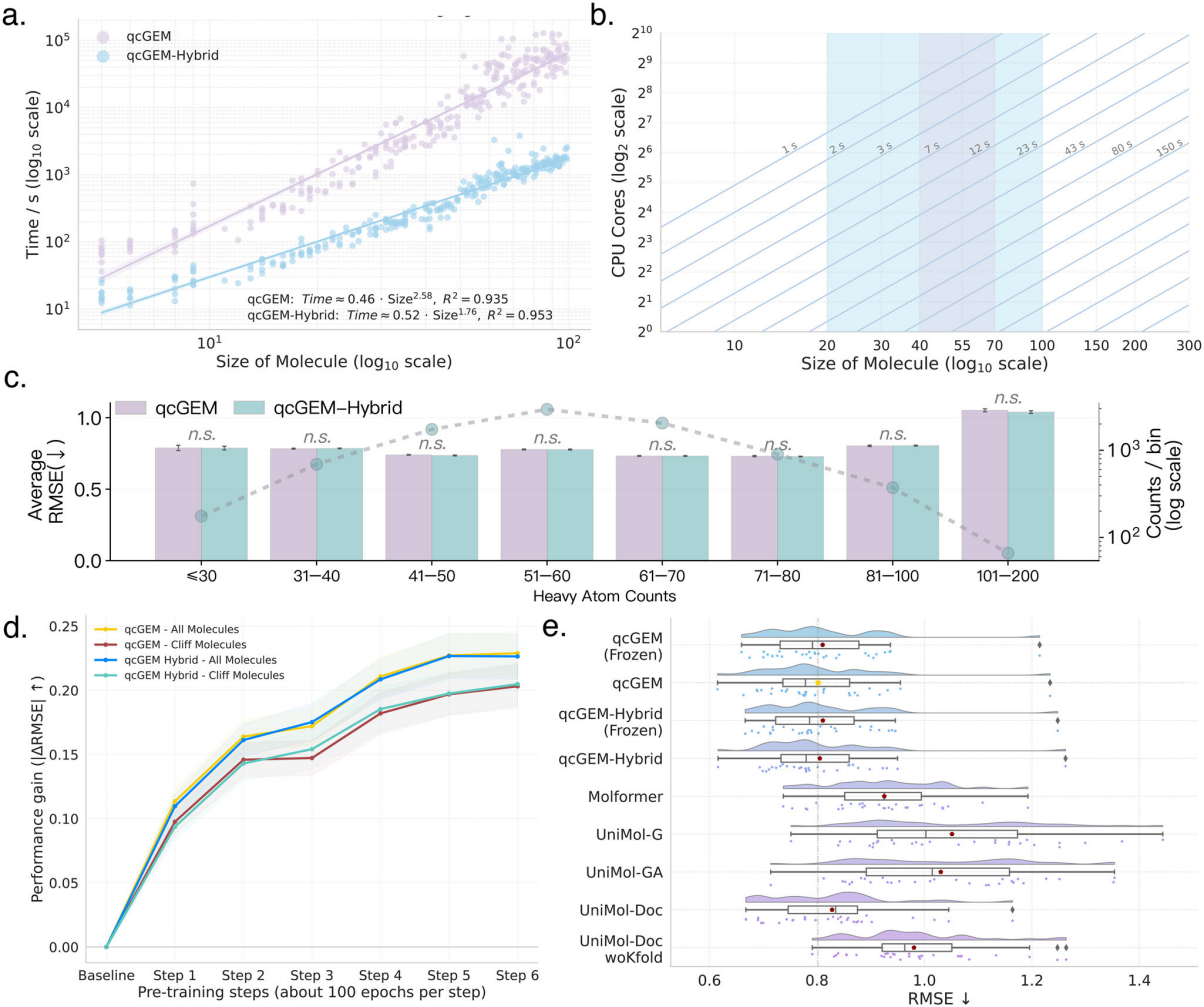
Evaluation of the efficiency and accuracy of qcGEM‐Hybrid. (a) Computation time versus molecular size for qcGEM (purple) and qcGEM‐Hybrid (blue) on the double logarithmic scale. The optimal parameters for the power‐law fitting are shown in the legend, with $R^{2}$ values indicated aside. (b) Dependence of the time consumption by qcGEM‐Hybrid on molecular size and CPU cores. The time cost is presented in the format of isolines, with the horizontal and vertical axes representing the molecular size and CPU core number, respectively, in the logarithmic scale. The shaded areas colored in blue and purple highlight ranges of molecular sizes for molecules of common interest and of high frequency in practical use, respectively. (c) Prediction errors of qcGEM (purple) and qcGEM‐Hybrid (green) on molecules of different sizes in activity cliff prediction. “n.s.” means the lack of statistically significant difference between the two groups. The curve of dashed line indicates the distribution of atom counts within the molecules tested. (d) Evaluation of qcGEM and qcGEM‐Hybrid in activity cliff prediction based on various model checkpoints collected in the pre‐training stage. The performance gain (in the vertical axis) is quantified by the reduction in prediction error (i.e., |ΔRMSE|), when compared to the baseline (the 20th epoch). (e) Comparison of the fixed (i.e., frozen) and fine‐tuned qcGEM/qcGEM‐Hybrid representations with various fine‐tuned versions of Molformer and UniMol in activity cliff tasks. Here, Molformer was fine‐tuned following the official guideline, while the fine‐tuning of UniMol was attempted in a number of strategies, including global‐token‐based fine‐tuning (UniMol‐G), global‐atom‐based fine‐tuning (UniMol‐GA), fine‐tuning according to the official documentation (UniMol‐Doc), and fine‐tuning without K‐fold cross‐validation (UniMol‐Doc woKfold). In each box plot, the central bar indicates the median, the box limits correspond to the lower and upper quartiles, the whiskers extend to 1.5 inter‐quartile range, and the star denotes the mean value. The vertical dash‐dotted line indicates the best performance in terms of the mean value.

In activity cliff tasks, the encoder shows a highly consistent pattern of prediction error (without significant differences in statistics), when using the two pipelines for quantum chemistry calculation (Figure 5c). In addition, when evaluated on model checkpoints collected from various stages in the pre‐training phase of the original qcGEM, qcGEM‐Hybrid also attains similar gains of performance (Figure 5d). These observations demonstrate that the pre‐trained qcGEM encoder is robust upon potential structural noises introduced by low‐precision geometry optimization procedures like GFN2‐xTB, further supporting our prior assumption. Results of a systematic performance–cost analysis could be found in Section S4.2.2.

Owing to its markedly enhanced efficiency, we evaluated the qcGEM‐Hybrid embedding under the fine‐tuning scenario against two state‐of‐the‐art baselines (Molformer [18] and UniMol [19]) that also support encoder fine‐tuning (Figure 5e). In activity cliff tasks, the fixed qcGEM‐Hybrid with frozen parameters has already surpassed fine‐tuned Molformer and UniMol, reaching a comparable performance to qcGEM. Moreover, with only minimal fine‐tuning on the encoder parameters, the performance of qcGEM/qcGEM‐Hybrid further improves, underscoring the flexible application modes of our representation in downstream tasks. Detailed information of fine‐tuning experiments could be found in Tables S46 and S47.

Overall, qcGEM‐Hybrid achieves a favorable trade‐off between efficiency and accuracy, ensuring both scalability and reliability for downstream applications. For applications requiring high‐precision predictions, qcGEM is recommended to generate high‐quality representations for repeated reuse. In contrast, qcGEM‐Hybrid provides a more efficient and reliable alternative in the large‐scale screening and/or fine‐tuning scenarios. By supporting representation reuse and rapid inference, qcGEM and its hybrid variant offer a practical and scalable solution as an efficient molecular filter.

### 2.7 Interpretability and Quantum Chemistry Awareness of qcGEM

After the previous systematic evaluations, in this section, we further illustrate the advantage of qcGEM embedding in practical application scenarios as well as physical interpretability.

In drug discovery, tasks grow increasingly complex from massive virtual screening to precise candidate optimization, shifting from simple ADMET predictions to intricate physical and geometric modeling. We categorize mainstream molecular representations into four paradigms of increasing information richness: Hash‐based fingerprints (e.g., ECFP), string‐based language models (e.g., Molformer), 2D topology‐based graphs (e.g., GROVER), and 3D structure‐based graphs (e.g., UniMol). As Figure 6a shows, unlike existing models that reach their application limits sooner or later due to their restricted formats and information, qcGEM's multi‐level representation and rich quantum features make it highly adaptable to all drug discovery scenarios, although the reliance on quantum chemical features may hinder its application in virtual screening beyond the million scale.

**FIGURE 6:**
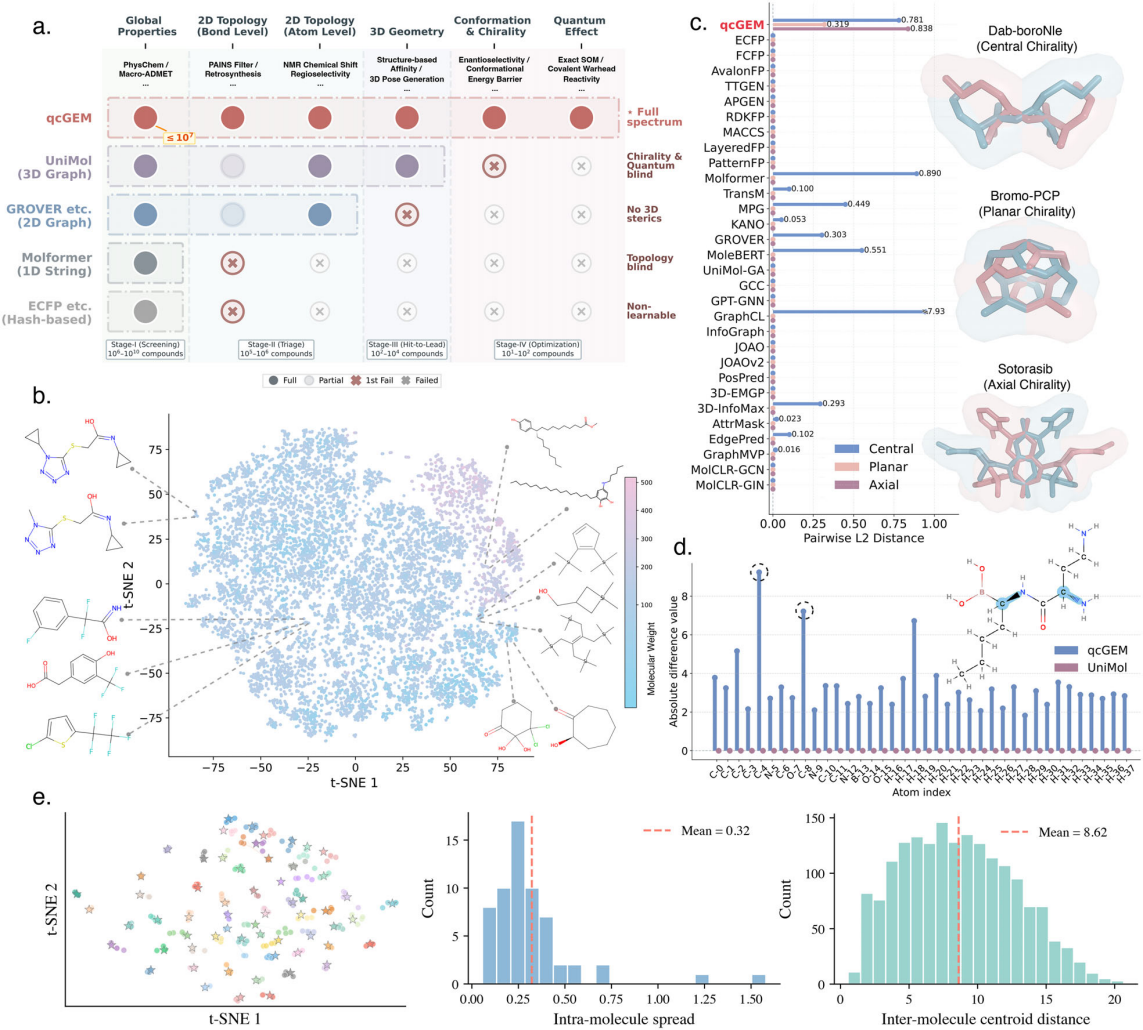
Analysis of the embedding of qcGEM. (a) Capability matrix of representative molecular representation across drug‐discovery stages ($10^{1}$–$10^{10}$ compounds). qcGEM spans all categories but is limited to under $10^{7}$. Filled, faded and crossed circles denote full, partial and failed capability, respectively. (b) t‐SNE visualization of qcGEM global‐level representations, colored by molecular weight. Representative molecules from distinct regions highlight meaningful geometric clustering in the learned embedding space. (c) Pairwise $L_{2}$ distance between enantiomeric embeddings across 31 encoders for three chirality classes: central (Dab‐boroNle), planar (Bromo‐PCP), and axial (Sotorasib). qcGEM uniquely discriminates all three classes, including axial and planar chirality, where all baselines fail. 3D superpositions of the corresponding enantiomeric pairs are shown on the right. (d) Atom‐level embedding differences between stereoisomers from two geometry‐encoding models. In the schematic chemical representation, atoms highlighted by a blue shadow represent chiral centers. In the bar plot, the difference between stereoisomers is shown for qcGEM (blue) and UniMol (red) embeddings in the atom‐wise manner, with chiral centers (i.e., C4 and C8) indicated by dashed circles. (e) Conformation‐resolved embedding of molecules with multiple conformers. Embeddings are projected into the 2D t‐SNE subspace (left), where conformers of the same molecular identity are indicated by the same color with lines connected to their centroid, and the lowest‐energy conformer is marked by star. Among these conformers, the intra‐molecular spread (middle, mean = 0.32) is about 27‐fold smaller than inter‐molecular centroid distance (right, mean = 8.62).

From the perspective of representation format, qcGEM provides a versatile hierarchical representation framework (at the global, node, and edge levels) to accommodate diverse downstream tasks. Unlike coarse‐grained formats (e.g., ECFP and Molformer) that lose atom/edge‐level fine‐grained resolution, or standard graph formats that fail in edge‐centric tasks due to the sparsity or absence of edge features, qcGEM preserves a complete multi‐resolution representation, which ensures not only robust task adaptability but also sufficient interpretability. We employed t‐distributed stochastic neighbor embedding (t‐SNE) [62] to visualize the landscape of the qcGEM representation space (Figure 6b). Although molecular weight predominantly determines the global distribution, molecules of high similarity in chemical formulas tend to cluster locally, with observable gradual transitions between adjacent clusters. The atomic‐level representations also show notable clustering: atoms of different types occupy distinct regions, and atoms of the same kind are further divisible based on their chemical environments (Figure S5a). We also present a case study on the edge‐level representation for 1‐naphthaldehyde (Figure S5b), a commonly used lead compound in drug discovery and fluorescence probe design. Clearly, qcGEM captures the characteristic non‐local interatomic interactions in this conjugate molecular system, for instance, the evident communication between C5 and O8 in the attention map. Systematic analysis on the distribution of all cross‐atom attention scores further reflects a physically meaningful hierarchy (Figure S5c): bonded atoms receive the highest attention scores, consistent with their role in defining molecular connectivity; non‐bonded pairs also receive sufficiently high attention, highlighting the model's sensitivity to through‐space non‐covalent interactions such as dispersion and π–π stacking; self‐loops, in contrast, receive the least attention, reflecting a trade‐off between preserving atomic identity and message updating.

From the perspective of information richness, qcGEM intrinsically fuses quantum chemistry with complete 3D structural encoding. The abundant quantum information can be reconstructed using a simple MLP (Figure S6), and the precise geometry can be recovered via the folding optimizer (Figure S7). Crucially, the adequate absorption of molecular geometry and quantum features renders qcGEM the power to effectively distinguish enantiomers arising from various symmetry operations, without external labels. When evaluated on exemplar molecules, qcGEM is the only representation that successfully distinguishes enantiomers of central, planar, and axial chiralities (Figure 6c). The success of Sotorasib deserves a further emphasis, since the precise pharmacological activity of this milestone drug that broke the “undruggable” curse of KRAS strictly relies on its axial chirality. Besides qcGEM, some other representations discriminate the central chirality via explicit symbols (e.g., Molformer) or RDKit‐based tags (e.g., GraphMVP). However, such chiral acquisition is susceptible to decay in the pre‐training phase due to the neglect of geometric features in these models. Conversely, in representative 3D models like UniMol, despite the large scale of molecular geometries utilized for pre‐training, chiral information is inherently absent due to the exploitation of chirality‐blind geometric features such as pairwise atomic distances (Figure 6d). On the other hand, albeit pre‐trained by equilibrium geometries only, qcGEM embeddings derived from different conformers of the same molecule present clustered distributions in the 2D t‐SNE subspace, with the intra‐molecule spread markedly smaller than the inter‐molecule centroid distance (Figure 6e). This observation indicates that qcGEM captures the essential chemical characteristics without losing the power of reflecting the conformational flexibility (see Section S4.3 for a series of systematic evaluations).

Collectively, qcGEM provides multi‐level representations for a wide range of tasks with strong interpretability. Equipped with rich quantum chemical knowledge and fully encoded geometric information, the model accommodates not only simple tasks in the early stages of drug discovery but also sophisticated geometry‐dependent tasks in later stages, making it applicable to diverse scenarios across the entire drug discovery pipeline.

## 3 Discussion

In this study, we propose qcGEM, a quantum‐chemistry‐aware graph‐based embedding of molecules, based on the localized quantum descriptors and the most stable molecular configurations, to address the shortcomings of nowadays molecular representations. Its physics‐intuition‐consistent architecture and rationally designed information flow enable efficient training, comprehensive encoding and strong interpretability. Under our experimental settings, that is, the unified frozen representation + MLP‐probe protocol, evaluation results demonstrate the robustness and generalizability of qcGEM across 41 molecular property tasks, 30 activity cliff tasks, and 12 protein–ligand interaction tasks. The comprehensive superiority over 32 tested baseline methods underscores its potential for filtering promising drug candidates. qcGEM further exhibits consistent physical relevance and interpretability across multiple levels. The complete geometry encoding enables accurate discrimination of chiral enantiomers, reflecting sensitivity to subtle yet critical molecular variations. Furthermore, the qcGEM‐Hybrid variant supports fast and reusable inference with robust accuracy, enabling scalable deployment for drug discovery.

According to the Born–Oppenheimer approximation, motions of nuclei and electrons are roughly separable due to their markedly different time scales, and nuclei are conceived to move slowly within the electronic potential energy surface. Hence, in a graph‐based molecular representation, nuclei are naturally modeled as nodes, with edges corresponding to the pairwise interactions between nuclei, which, in principle, should be quantified by electronic wave functions. However, in mainstream molecular representations, edges are frequently embedded from empirical chemical bonds (e.g., generated by RDKit [63] or Open Babel [64]), a scheme that inherently overlooks the diffusive electronic orbitals spanning over more than two atomic centers, such as the π orbitals in conjugate molecular systems. The neglect of such non‐local quantum effects will inevitably introduce systemic errors, which can hardly be rectified by simply amplifying the scale of data and labels as in many popular pre‐training frameworks. To remedy this deficit, we train qcGEM by incorporating several localized quantum descriptors (e.g., natural bond orbitals, electron localization function, etc.; see Section S4.1.2 for the ablation study on their contributions) as well as the three‐dimensional molecular structure. By this means, our framework effectively treats nuclei as well as inner‐shell and lone‐pair electrons as nodes, while considers the other valence‐shell electronic orbitals as edges, agreeing with chemical intuition. The evident advantage of our representation over traditional ones in the large number of downstream tasks further reinforces the necessity of considering non‐local (i.e., beyond empirical bonds) atomic interactions in molecular representation learning.

Despite the advantages, there remain a few limitations in the current qcGEM framework. First, qcGEM relies on pre‐generated quantum chemical annotations for model training and inference. Although the quantum chemistry computation is substantially accelerated in the qcGEM‐Hybrid pipeline, the lack of instantaneous embedding generation hinders its direct application in computational intensive tasks like the large‐scale compound screening. With pre‐computed high‐quality quantum descriptors for 1.2 million molecules in the qcMol database, qcGEM is more suitable for the late stage of drug discovery, such as lead optimization, which demands high‐precision, physics‐rich embeddings to guarantee the evaluation accuracy. Second, although capable of generating highly consistent embeddings when starting from different conformers, qcGEM is pre‐trained by globally optimal molecular conformations only. In the future, multiple conformers of the same molecule should be incorporated into the training set, aiming to improve the sensitivity of qcGEM embedding to conformational flexibility. Third, incorporating additional level information like coarse‐grained functional groups or molecular fragments may further enrich the representation with a new dimension of granularity, improving its chemical interpretability as well as practical applicability to a broader range of downstream tasks. Fourth, the study of qcGEM‐Hybrid suggests that while quantum chemical information is essential, low precision computation often suffices to achieve excellent performance. This is partly because overly precise signals tend to attenuate as they propagate through deep network layers, limiting their constraining ability. Moreover, downstream tasks often depend more on the distributional characteristics of representations than on their exact values. Therefore, future work will explore contrastive learning strategies to generate low‐precision quantum chemical features for the further improvement of computational efficiency. Last, although this study emphasizes the use of a relatively small pre‐training dataset, further performance gains may be achieved by scaling up data volume and model capacity.

## 4 Methods

### 4.1 Molecular Graph Construction

qcGEM abstracts each molecule as a complete graph with multi‐scales: M=(G,R,V,E), where G stands for the global state (i.e., molecular properties), $R={\left\{r_{i}\right\}}_{i\in [1,N_{v}]}$ refers to the coordinates of atomic nuclei in the initial geometry (i.e., molecular structure), $V={\left\{v_{i}\right\}}_{i\in [1,N_{v}]}$ denotes the set of nodes (i.e., atomic centers), $E={\left\{e_{ij}\right\}}_{i,j\in [1,N_{v}]}$ describes the set of edges (i.e., interatomic interactions), and $N_{v}$ is the number of atoms.

The global state is initialized with a learnable token, while the nodes and edges are initialized using localized quantum chemical features. Quantum chemical features could be obtained from the qcMol database or computed from scratch using GFN2‐xTB [61] for geometry optimization and ORCA [65] at the level of B3LYP‐D3/def2‐SV(P) for quantum chemistry calculation, followed by wave function analysis with NBO [66] and Multiwfn [42]. The complete feature details of nodes and edges are provided in Tables S1 and S2, respectively. We denote the initial features of global molecular state G, coordinates R, node V, and edge E as g, $r_{i}$, $v_{i}$, and $e_{ij}$, respectively. Notably, in the final representation graph $M_{\text{rep}}$, R is incorporated into V in the roto‐translation‐invariant manner after encoding.

### 4.2 Model Architecture

The qcGEM model adopts an autoencoder framework to extract the representation of a compound (Algorithm 1). After feature pre‐processing (Algorithm 2), the encoder employs 16 repeated encoding blocks to encode molecular graph M as the representation $M_{\text{rep}}$ (Algorithm 3). This representation is then passed to the decoder (Algorithm 4), where it is used to reconstruct the molecular geometry, quantum chemical information, and global state (Algorithm 5).

At the beginning of the encoding process, the geometry module extracts geometric features from the Cartesian coordinates R, which are then used as positional embeddings $PE_{\text{node}}$ and $PE_{\text{edge}}$ for nodes and edges, respectively (Algorithm 6)

(1) $$\begin{array}{c} PE_{\text{node}},PE_{\text{edge}}=\text{GeometryModule}\left(R\right) \end{array}.$$

In every encoding block, the node features $v_{i}$ are first updated to obtain the node embeddings $h_{v_{i}}$ via a self‐attention mechanism applied over all node pairs (including self‐loops) based on both node features $v_{i}$ and pairwise edge information $e_{ij}$ (Algorithm 7). In this process, the geometric features $PE_{\text{node}}$ and $PE_{\text{edge}}$ are also incorporated through attention bias and feature concatenation

(2) $$\begin{array}{c} v_{i}^{\prime }=W_{1}v_{i}^{l}+\sum_{j\in [1,N_{v}]}\alpha_{ij}\left(W_{2}v_{j}^{l}+W_{3}e_{ij}^{l}\right)\\ \alpha_{ij}=\text{softmax}\left(\frac{{\left(W_{4}v_{i}^{l}\right)}^{⊤}\left(W_{5}v_{j}^{l}+W_{3}e_{ij}^{l}\right)}{\sqrt{d}}+{PE}_{\text{edge},ij}\right)\\ h_{v_{i}}=\text{concat}\left(v_{i}^{\prime },{PE}_{\text{node},i}\right) \end{array},$$

where the superscript l denotes the index of encoding block, W represents the trainable matrices for linear projection, d is the feature dimension of node v, $\alpha_{ij}$ stands for the attention score between $v_{i}$ and $v_{j}$, while softmax(·) and concat(·) refer to the softmax function and concatenation operator, respectively.

Subsequently, the GLI unit is applied to facilitate information exchange between global and local representations (Algorithm 8). In this module, node and edge representations are read out to form local representations, concatenated with the global token, and further processed to enable information fusion. The output is then broadcast to node and edge to update their representations

(3) $$\begin{array}{c} {\tilde{h}}_{o},{\tilde{h}}_{v_{i}},{\tilde{h}}_{e_{ij}}=\text{GLI}\left(g^{l},h_{v_{i}},e_{ij}^{l}\right) \end{array},$$

where $\tilde{h}$ denotes the hidden states of output o, node v, and edge e, respectively.

After sufficient information exchange, the node v and edge e are updated based on the contextual information learned through the GLI module (Algorithm 9). The global token is refined with aggregated local information to produce a meaningful global embedding (Algorithm 10)

(4) $$\begin{array}{c} v_{i}^{l+1}=\sigma \left(f_{\text{lin}}\left({\tilde{h}}_{v_{i}}\right)\right)\\ e_{ij}^{l+1}=\sigma \left(f_{\text{lin}}\left(\text{concat}\left(v_{i}^{l+1},{\tilde{h}}_{e_{ij}},v_{j}^{l+1}\right)\right)\right)\\ g^{l+1}=\sigma \left(f_{\text{lin}}\left({\tilde{h}}_{o}\right)\right) \end{array},$$

where $f_{\text{lin}}$ denotes linear layers of the module, and σ represents the nonlinear activation function.

To construct a hierarchical molecular representation and promote stable gradient propagation, we aggregate the output of encoding blocks from a pre‐defined number (L) of layers, each with different semantic information. Finally, the input molecular graph M is progressively transformed into a compact, quantum‐chemistry‐aware representation $M_{\text{rep}}$ comprising global, node, and edge levels,

(5) $$\begin{array}{c} M_{\text{rep}}=\frac{1}{\left|L\right|}\sum_{l\in L}M_{l},\;L=\left\{1,4,8,12,16\right\} \end{array}.$$

In the decoding stage, the folding optimizer, QC recovery unit and global predictor independently decode the graph molecular representation to recover the molecular geometry, quantum chemical features, and global molecular state, respectively

(6) $$\begin{array}{c} \hat{M}=\left(\hat{G},\hat{R},\hat{V},\hat{E}\right)=\text{Decoder}\left(M_{\text{rep}}\right) \end{array},$$

where Decoder(·) denotes the decoder function that consists of the folding optimizer, QC recovery unit and global predictor, and symbols with a hat ($\hat{\cdot }$) denote reconstructed results.

#### 4.2.1 Geometry Module

To mitigate geometry information loss observed in previous methods [67, 68], we referred to and modified the ComENet [69] framework to enhance extraction efficiency while preserving compatibility with molecules processed by qcGEM (Algorithm 6). We split the entire geometry information into multiple local, complete geometries, utilizing (d,θ,ϕ,τ) in the spherical coordinate system to achieve local completeness. Notably, for an atom or bond, such variables are computed from the local 3D vector geometry falling within a distance cutoff, and thus are inherently independent of atom order assignment. By incorporating complete local geometries as positional embeddings (PEs) into each qcGEM encoder block, the representation preserves a complete memory of the geometry. Furthermore, this operator could mitigate the over‐smoothing problem in GNNs [70, 71]. The detailed formulation is provided in Section S1.2.

#### 4.2.2 GLI Unit

The GLI unit is designed for information exchange between g, $v_{i}$, and $e_{ij}$. It is a bidirectional unit that facilitates both the bottom‐up flow of local information to global representations and the top‐down refinement from global to local features (Algorithm 8). In the local‐to‐global information flow, node and edge embeddings are aggregated via mean pooling and then used to update the global token. In the global‐to‐local phase, the global embedding is propagated back to local nodes, providing contextual guidance for refining the local representation. The concatenated information is further fused through the self‐attention mechanism. This strategy provides a multi‐level representation, facilitating adaptation to diverse tasks, while simultaneously enhancing the readout training of node and edge features to reduce global feature entanglement [33]. The detailed formulation is provided in Section S1.3.

#### 4.2.3 Folding Optimizer

The folding optimizer is designed with a dual‐flow architecture that incorporates both 1D and 2D geometry information (Figure S1a; Algorithm 12). An initial geometry is generated from 1D features under the guidance of 2D structural information. This geometry is first evaluated through pairwise distance constraints in the 2D channel and then fed back into the 1D channel. The model then uses 1D features to predict the difference between initial and ground‐truth geometries. Through iterative refinement, the folding optimizer drives the predicted structure toward a more stable and energetically favorable conformation. The detailed formulation is provided in Section S1.4.

### 4.3 Loss Design

The total loss (Algorithm 18) is composed of three components: global prediction loss (Algorithm 19), node and edge feature recovery losses (Algorithms 20 and 21), and geometry loss (Algorithm 22). Non‐geometry‐related tasks are supervised under the $L_{1}$ and cross‐entropy losses. In contrast, for geometry‐specific tasks, we introduce a tailored geometric constraining loss to enhance learning stability and convergence,

(7) $$\begin{array}{c} L_{\text{total}}=L_{\text{global}}+L_{\text{node}}+L_{\text{edge}}+L_{\text{geometry}}\\ L_{\text{geometry}}=L_{\text{PV}}+L_{\text{PD}}+L_{\text{RMSD}}+L_{\text{FAPE}}. \end{array}.$$

The geometric constraint loss contains PV loss, PD loss, RMSD loss, and FAPE loss for model learning. The PV loss (pairwise vector difference), PD loss (pairwise distance difference), and RMSD loss (root mean square deviation) are used to constrain the overall molecular geometry. We specifically propose a small‐molecule‐oriented FAPE [45] (i.e., frame aligned point error) loss to impose more effective structural constraints (Algorithm 23).

**FAPE loss**: To better constrain local geometric details, we define rigid triangles formed by heavy atoms as local frames (Figure S1b). A local coordinate system is constructed for each frame to facilitate the calculation of geometric deviations. By evaluating geometric errors across all frames within a molecule, the model enforces constraining from multiple local perspectives. The FAPE loss serves as a strong geometric constraint on local details and is used when the convergence is challenging. The detailed formulation is provided in Section S1.6.

### 4.4 Pre‐Training Strategy

The qcGEM model utilizes geometry denoising, node‐edge feature recovery and correction, and global condensation for pre‐training. For the denoising task, random noise in the range of −1 to 1 Å  is added to the native coordinates, and the model is trained to refine the perturbed geometry toward the ground truth. For node‐edge feature recovery and correction, input features are either randomly masked with a learnable token or mutated by randomly sampled features within the same training batch. The model learns to identify and differentiate between true and erroneous features and subsequently recover the correct one. We experimented with various masking and replacement ratios, and ultimately trained the model with a 30% masking ratio, a 30% replacement ratio (Algorithm 2). For the global condensation task, the model condenses the global state from nodes and edges to update the token‐initialized global features, which are subsequently used to predict target descriptors.

The overall pre‐training process involves multiple stages (Figure S2), with hyperparameters summarized in Table S3 and details listed in Table S4. Ablation studies on the robustness of hyperparameters and training strategies are shown in Figure S3.

### 4.5 Evaluation Metrics

For compound properties, we use RMSE to evaluate regression tasks and adopt the area under the ROC curve (ROC‐AUC) for classification tasks. To further assess the robustness of different methods, we propose the TRSS score as a novel metric

(8) $$\begin{array}{c} w_{k}=\frac{K-k+1}{\sum_{i=1}^{K}i},\\ \text{TRSS}=\frac{1}{N}\sum_{k=1}^{K}w_{k}\cdot T_{k} \end{array}$$

where N denotes the total number of tasks, and $T_{k}$ refers to the occurring count of a method within the top k ranks. TRSS quantifies the robustness of a method by measuring how frequently it ranks within the top K across multiple tasks. A linearly decaying weight $w_{k}$ is assigned to the rank position k to favor higher rankings. Unlike metrics that penalize occasional poor performance, TRSS rewards consistent top‐tier performance, thus mitigating the influence of outliers. This design makes it particularly suitable for real‐world evaluation scenarios, where stability and reliability across diverse datasets are often more critical than occasional peak performance on a limited number of subsets.

In addition, we also provide a traditional metric, the average rank (AR), as a supplement to quantify the performance:

(9) $$\mathrm{AR}=\frac{1}{N}\sum_{t=1}^{N}r_{t},$$

where $r_{t}$ refers the ranking of a method in task t (among N benchmarks). Unlike the TRSS metric, a better method generally achieves a lower AR.

### 4.6 More Computational Details

We performed quantum chemical calculation with B3LYP‐D3/def2‐SV(P)//GFN2‐xTB by ORCA 5.0.3 [65, 72, 73, 74, 75, 76, 77, 78] and GFN2‐xTB 6.5.1 [61]. The post‐analysis of wave functions was done by Multiwfn 3.8 [42] and NBO 7.0 [66]. Molecules were initially processed by RDKit 2022.09.5 [63] and Open Babel 3.1.1 [64]. The qcGEM model was implemented with PyTorch [79] version 2.0.0 and PyTorch Geometric [80] version 2.5.2 with CUDA version 12.0 and Python 3.8.19. Adam optimizer with learning rate ranging between $3\times 10^{-4}$ and $3\times 10^{-5}$ was used to optimize the model (Table S4). The model comprises approximately 37 million parameters, of which about 36 million belong to the encoder. Learning rate of the MLP probe network was set to $1\times 10^{-4}$ with a weight decay of 0.01.

## Author Contributions

H.G. proposed the theory and concept. H.W. and H.G. designed the methodology. H.W. accomplished coding, model training, performance evaluation, and data analysis. H.G. supervised the whole process. Z.Z. and S.H.N. contributed to data analysis and figure generation. H.W. and H.G. wrote the initial draft. All authors were involved in revision and agreed with the final manuscript.

## Conflicts of Interest

The authors declare no conflicts of interests.

## Code availability

The source code of qcGEM can be accessed from the GitHub repository at https://github.com/GHUSER‐haoyu/qcGEM, with model parameters obtainable from the Zenodo repository at https://doi.org/10.5281/zenodo.17364257.

Pre‐calculated qcGEM embeddings are freely downloadable from the website of qcMol database at https://structpred.life.tsinghua.edu.cn/qcmol/embedding_download.html. The online server of qcGEM provides embedding generation service for submitted molecules at https://structpred.life.tsinghua.edu.cn/server_qcgem.html.

## Supporting information


**Supporting File**: advs77997‐sup‐0001‐SuppMat.pdf.

## Data Availability

**Pre‐training dataset**: We used the subset of PubChem quantum chemical annotation from the qcMol [44] database as our pre‐training materials. **Evaluation dataset**: qcGEM was evaluated on the MoleculeNet [47] dataset, the ADMET subset from TDC [48] dataset and the PharmaBench dataset [49] for compound properties tasks, on the MoleculeACE [52] dataset for activity cliff estimation tasks, and on the BindingDB [54], Human [55], C. elegans [55], KIBA [56], Davis [57], Opioids [58] and CASF‐2016 [59] datasets for multi‐level protein‐ligand interaction tasks. The pre‐training data are available from the Zenodo repository at https://doi.org/10.5281/zenodo.17576389. The downstream‐task evaluation results, including the numerical data underlying the corresponding figures, are provided in the Supplementary Information.
