# Supplementary material for: qcGEM: A Graph‐Based Molecular Representation With Quantum Chemistry Awareness
Source: Adv Sci (Weinh). 2026 Oct 18:e77997. Online ahead of print. doi: 10.1002/advs.77997 (PMC13676819; doi:10.1002/advs.77997)
Supplement: Supplementary file 1 [file ADVS-9999-e77997-s001.pdf]

Supplementary Information

qcGEM: a graph-based molecular representation with quantum chemistry awareness

Haoyu Wang[1,2,3,4], Ziyan Zhang[1,2,3,4], Swee Hong Ng[1,2,3,4], and Haipeng Gong[1,2,3,4,*]

[1]MOE Key Laboratory of Bioinformatics, School of Life Sciences, Tsinghua University, Beijing, 100084, China

[2]State Key Laboratory of Green Manufacturing, Tsinghua University, Beijing, 100084, China

[3]Center of Life Sciences and Artificial Intelligence, School of Life Sciences, Tsinghua University, Beijing, 100084, China

[4]Beijing Frontier Research Center for Biological Structure, Tsinghua University, Beijing, 100084, China

[*]To whom correspondence should be addressed: hgong@tsinghua.edu.cn

1 Supplementary Methods

1.1 Model architecture

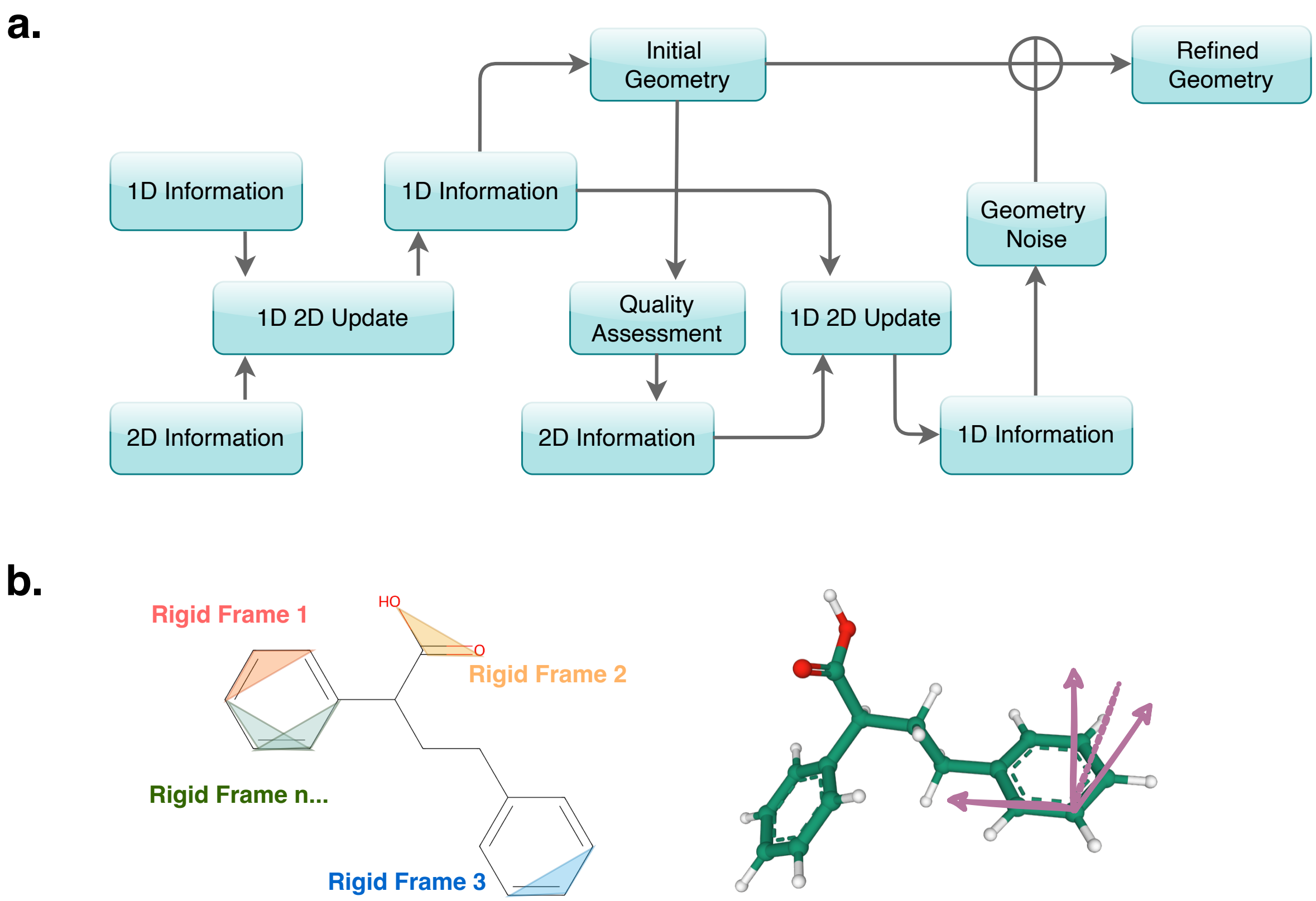

Supplementary Figure 1. Detail of qcGEM model design. a) The folding optimizer produces an initial and refined geometry, supporting iterative geometry refinement. **b)** The FAPE loss specifically tailored for small molecules enhances geometric supervision by constructing chemically reasonable local coordinate systems from multiple perspectives.

1.2 Geometry module

Let $\mathcal{AT} = \{at_i\}$ denote the atom types and let $\mathcal{R} = \{\mathbf{r}_i\}$ denote the set of atomic coordinates in a molecule, where $\mathbf{r}_i \in \mathbb{R}^3$ represents the position of the i-th atom. The set of edges $\mathcal{E}$ is defined as:

- $(i, j) \in \mathcal{E} \quad \text{if} \quad \|\mathbf{r}_i - \mathbf{r}_j\| < r_{\text{cutoff}}$,

where i and j refer to indices of atomic nodes, r_{cutoff} denotes a pre-defined hyper-parameter that determines the spatial extent of local geometric neighborhoods.

To define the geometric feature for an edge, *e.g.*, starting from node i and ending at node j, a local coordinate system is first established by finding the nearest and the second nearest neighboring atoms of node i, $n_0(i)$ and $n_1(i)$, based on Euclidean distances in the Cartesian space. Using these reference atoms, we derive four roto-translationally invariant variables:

- $d_{ij} = \|\mathbf{r}_i - \mathbf{r}_j\|$;

- $\theta_{ij} = \angle_{\text{angle}}(\mathbf{r}_{n_0(i)} - \mathbf{r}_i, \mathbf{r}_j - \mathbf{r}_i)$;
- $\phi_{ij} = \angle_{\text{dihedral}}(\mathbf{r}_{n_1(i)} - \mathbf{r}_{n_0(i)}, \mathbf{r}_{n_0(i)} - \mathbf{r}_i, \mathbf{r}_i - \mathbf{r}_j)$;
- $\tau_{ij} = \angle_{\text{dihedral}}(\mathbf{r}_{n_0(i)} - \mathbf{r}_i, \mathbf{r}_i - \mathbf{r}_j, \mathbf{r}_j - \mathbf{r}_{n_0(j)})$.

The set of variables $(d_{ij}, \theta_{ij}, \phi_{ij}, \tau_{ij})$ are then used to compute relative geometric features in the local space,

$$\begin{aligned} \text{torsion}_{ij} &= \sigma(f_{\text{lin}}(d_{ij}, \theta_{ij}, \phi_{ij})) \\ \text{angle}_{ij} &= \sigma(f_{\text{lin}}(d_{ij}, \tau_{ij})) \end{aligned} \quad , \tag{S1}$$

where $f_{\text{lin}}(\cdot)$ denotes linear layers and $\sigma(\cdot)$ represents the non-linear activation function.

The local geometric features are first transformed by non-linear operation,

$$\begin{aligned} \text{geo\_feat}_{1,ij} &= \sigma(f_{\text{lin}}(\text{torsion}_{ij})) \\ \text{geo\_feat}_{2,ij} &= \sigma(f_{\text{lin}}(\text{angle}_{ij})) \end{aligned} \ , \tag{S2}$$

and then act as edge weights to integrate atom type information from neighborhood $\mathcal{N}$ in the graph,

$$\begin{aligned} at_i &= \sigma(f_{\text{lin}}(at_i)) \\ \text{at\_feat}_{1,i} &= W_6\, at_i + W_7 \sum_{j \in \mathcal{N}(i)} \text{geo\_feat}_{1,ij} \cdot at_j \\ \text{at\_feat}_{2,i} &= W_8\, at_i + W_9 \sum_{j \in \mathcal{N}(i)} \text{geo\_feat}_{2,ij} \cdot at_j \end{aligned} , \tag{S3}$$

where W represents the trainable matrices for linear projection.

We subsequently perform a nonlinear transformation and aggregate these local information to derive spatial positional embeddings PE_{node} and PE_{edge} for nodes and edges, respectively,

$$\begin{aligned} PE_{\text{node},i} &= \sigma(f_{\text{lin}}(\text{concat}(\text{at\_feat}_{1,i}, \text{at\_feat}_{2,i}))) \\ PE_{\text{edge},ij} &= d_{ij} \end{aligned} \quad , \tag{S4}$$

where $\text{concat}(\cdot)$ refers to the concatenation operator.

1.3 GLI unit

For interactions between the local information and the global state, we employ GNN readouts combined with PyTorch's broadcasting operations to facilitate message propagation:

$$
\begin{aligned}
v' &= \text{readout}(\mathcal{V}) = \frac{1}{|\mathcal{V}|}\sum_{v_i \in \mathcal{V}} v_i \\
g' &= \sigma(f_{\text{lin}}(\text{concat}(v', g)))
\end{aligned}
\quad , \tag{S5}
$$

where g and v_i represent the input feature of global state and nodes, respectively.

A multi-head attention mechanism is further utilized to enable deep fusion of global and local information (Figure 1c):

$$
\begin{aligned}
o &= \text{softmax}\left(\frac{(W_{10}g')^{\top}(W_{11}g')}{\sqrt{d}}\right)(W_{12}g') \\
\tilde{o} &= g' + o
\end{aligned}
\quad , \tag{S6}
$$

$$
\begin{aligned}
\tilde{v}_i &= \text{concat}(v_i, \tilde{o}), \quad \forall i \in \{1, 2, \ldots, N_v\} \\
\tilde{e}_{ij} &= \text{concat}(e_{ij}, \tilde{o}), \quad \forall i, j \in \{1, 2, \ldots, N_v\}
\end{aligned}
. \tag{S7}
$$

Here, v_i and e_{ij} represent the original inputs of nodes and edges, while $\tilde{o}$, $\tilde{v}_i$ and $\tilde{e}_{ij}$ are the final outputs for the global state, nodes and edges, respectively. W represents the trainable matrices for linear projection, d is the dimension of key vector, and N_v denotes the number of atoms.

1.4 Folding optimizer

First, we apply a nonlinear transformation to the global representation g_{rep}, the node representation $n_{\text{rep},i}$, and the edge representation $e_{\text{rep},ij}$:

$$
\begin{aligned}
g &= \sigma(f_{\text{lin}}(g_{\text{rep}})) \\
v_i &= \sigma(f_{\text{lin}}(v_{\text{rep},i})) \\
e_{ij} &= \sigma(f_{\text{lin}}(e_{\text{rep},ij}))
\end{aligned}
. \tag{S8}
$$

Here, g, n_i and e_{ij} are updated intermediate values of the global state, node and edge, respectively.

The node-edge exchange (NEE) module (see **the following section** for details) then aggregates node-edge information to produce updated representations. Using the updated node states, the decoder generates an initial structural prediction $\mathbf{r}_i^{\text{baseline}}$ as a baseline:

$$
\begin{aligned}
v_i^{(1)}, e_{ij}^{(1)} &= \text{NEE}^{(1)}(v_i, e_{ij}) \\
\mathbf{r}_i^{\text{baseline}} &= \sigma(f_{\text{lin}}(v_i^{(1)}))
\end{aligned}
\quad , \tag{S9}
$$

where the superscript represents the order of the NEE module.

This baseline is discretized via binning on the distance d_{ij} to estimate pairwise distance ranges, which are fed back as b_{ij} to edges for refinement:

$$
\begin{aligned}
{d_{ij}^{(1)}}^2 &= \|\mathbf{r}_j^{\text{baseline}} - \mathbf{r}_i^{\text{baseline}}\|^2 \\
b_{ij}^{(1)} &= \left[\mathbb{I}({d_{ij}^{(1)}}^2 < c_1),\ \mathbb{I}({d_{ij}^{(1)}}^2 < c_2),\ \ldots,\ \mathbb{I}({d_{ij}^{(1)}}^2 < c_K)\right], \\
e'_{ij} &= \text{concat}(e_{ij}^{(1)}, b_{ij}^{(1)})
\end{aligned}
\tag{S10}
$$

where $(c_1, c_2, \ldots, c_K)$ is series of bin ranges, and $\mathbb{I}(\cdot)$ is the indicator function, which outputs 1 if the condition is satisfied and 0 otherwise.

With the refined node and edge features, the model predicts the current structural noise $\delta\mathbf{r}_i$ to correct the baseline $\mathbf{r}_i^{\text{baseline}}$, yielding the final structure $\hat{\mathbf{r}}_i$:

$$
\begin{aligned}
v_i^{(2)}, e_{ij}^{(2)} &= \text{NEE}^{(2)}(v_i^{(1)}, e'_{ij}) \\
\delta\mathbf{r}_i &= \sigma(f_{\text{lin}}(v_i^{(2)})) \\
\hat{\mathbf{r}}_i &= \mathbf{r}_i^{\text{baseline}} + \delta\mathbf{r}_i
\end{aligned}
\quad .
\tag{S11}
$$

1.5 NEE module

In the three-step process of updating node v_i and edge e_{ij} representations, the model first computes pairwise node differences by comparing the features of node pairs. Specifically, vector differences Δ_{ij}, Euclidean distance squares d_{ij}^2, and cosine similarity $\cos\theta_{ij}$ are calculated to construct node difference features v_{diff}:

$$
\begin{aligned}
\Delta_{ij} &= v_j - v_i \\
d_{ij}^2 &= \|\Delta_{ij}\|^2 = \|v_j - v_i\|^2 \\
\cos\theta_{ij} &= \frac{\langle v_i, v_j\rangle}{\|v_i\| \cdot \|v_j\|} \\
v_{\text{diff}} &= \text{concat}(\Delta_{ij}, d_{ij}^2, \cos\theta_{ij})
\end{aligned}
\quad .
\tag{S12}
$$

In the second step, these difference features are concatenated with the original node attributes v_i and v_j, which are then used to update the corresponding edge features e_{ij}:

$$
e'_{ij} = \sigma(f_{\text{lin}}(\text{concat}(v_i, v_j, v_{\text{diff}}, e_{ij}))) \, .
\tag{S13}
$$

Finally, the updated edge features e'_{ij} are aggregated as messages m_i, which are integrated back into the node representations v'_i, enabling bidirectional information exchange between nodes and edges:

$$
\begin{aligned}
m_i &= \sum_{j \in \mathcal{N}(i)} e_{ij} \\
v'_i &= \sigma(f_{\text{lin}}(\text{concat}(v_i, m_i)))
\end{aligned}
\quad .
\tag{S14}
$$

1.6 Geometry loss design

To more effectively constrain molecular structures and facilitate rapid convergence, we introduce a set of loss functions targeting different aspects of geometric consistency. These include:

distance error loss

$$\mathcal{L}_{\mathrm{PD}} = \frac{1}{N^2}\sum_{i=1}^{N}\sum_{j=1}^{N}\left(\|\hat{\mathbf{r}}_i - \hat{\mathbf{r}}_j\| - \|\mathbf{r}_i - \mathbf{r}_j\|\right)^2, \tag{S15}$$

which penalizes deviations in inter-atom distances;

root mean square deviation (RMSD) loss

$$\mathcal{L}_{\mathrm{RMSD}} = \sqrt{\frac{1}{N}\sum_{i=1}^{N}\|\hat{\mathbf{r}}_i - \mathbf{r}_i\|^2}, \tag{S16}$$

which evaluates global structural differences;

and vector difference loss

$$\mathcal{L}_{\mathrm{PV}} = \frac{1}{N^2}\sum_{i=1}^{N}\sum_{j=1}^{N}\left\|\frac{\hat{\mathbf{r}}_i - \hat{\mathbf{r}}_j}{\|\hat{\mathbf{r}}_i - \hat{\mathbf{r}}_j\|} - \frac{\mathbf{r}_i - \mathbf{r}_j}{\|\mathbf{r}_i - \mathbf{r}_j\|}\right\|^2, \tag{S17}$$

which jointly constrains both distance and directional deviations.

Here, the $\hat{\mathbf{r}}_i$ and $\mathbf{r}_i$ represent the predicted and ground-truth coordinates of atom i, respectively.

These losses serve to enforce the global structural integrity of molecular configurations. In addition, qcGEM incorporates the Frame Aligned Point Error (FAPE) loss

$$\mathcal{L}_{\mathrm{FAPE}} = \frac{1}{FN}\sum_{f=1}^{F}\sum_{i=1}^{N}\min\left(\|\hat{\mathbf{r}}_{fi} - \mathbf{r}_{fi}\|^2, c\right), \tag{S18}$$

to provide localized structural supervision. Here, $\mathbf{r}_{fi}$ corresponds to the relative coordinate of atom i in the f-th local frame, and c is the cutoff, which is set as 10 Å by default. Our adapted version of FAPE is particularly effective in correcting local geometric distortions, thereby enhancing the fidelity of small-molecule reconstructions.

2 Supplementary Information for Model Pre-training

2.1 Model features

Supplementary Table 1. Node features of qcGEM

Type	Description
Geometry	Cartesian atomic positions (x, y, z) defining the molecular geometry.
Atomic Number	Atomic number is the proton count that uniquely identifies an element.
Node Degree	Node degree is the count of bonds an atom forms with other atoms.
Formal Charge	Formal charge is the net atomic charge assigned from electron counting rules.
Linked Hydrogens	Linked hydrogens is the count of H atoms attached to an atom.
Radical Electron	Radical electron is the count of unpaired electrons on an atom.
Charge of NPA	Net atomic charge assigned by NPA.
Core of NPA	Number of electrons assigned to core (inner-shell) orbitals.
Valence of NPA	Number of electrons assigned to valence orbitals (involved in bonding).
Rydberg of NPA	NPA Rydberg population represents electrons assigned to diffuse, high-energy orbitals beyond the valence shell.
LI feature	LI quantifies the number of localized electrons associated with an atom, indicating electron localization.
ADCH charge	ADCH charge is a dipole-corrected variant of Hirshfeld atomic charges that better reproduces molecular dipole moments.
NAO feature	NAOs are orthogonalized atomic orbitals used as the localized basis in NBO analysis.
NBO-LP feature	Occupancy of a nonbonding localized orbital associated with a single atom (*i.e.* lone electron pair).

Abbreviations:
NPA: Natural Population Analysis
LI: Localization Index
ADCH: Atomic Dipole Corrected Hirshfeld
NAO: Natural Atomic Orbital
NBO: Natural Bond Orbital
LP: Lone Pair

Supplementary Table 2. Edge features of qcGEM

Type	Description
Bond type	Classification of the bond (*e.g.*, single, double, triple, or aromatic).
Ring indicator	Specifies whether an atom is part of a ring structure.
Bond stereochemistry	Spatial configuration of a bond (*e.g.*, cis/trans or E/Z).
Aromaticity indicator	Specifies whether an atom or bond belongs to an aromatic system.
Conjugation indicator	Specifies whether a bond is part of a conjugated system.
ELF feature	ELF measures the probability of finding localized electron pairs (with opposite spins), such as bonds or lone pairs in real space.
LBO feature	LBO quantifies bond strength from the Laplacian of electron density at the bond region, reflecting electron localization between atoms.
DI feature	DI measures the number of electron pairs shared between two atoms, serving as a real-space definition of bond order.
MBO feature	MBO quantifies bond strength using molecular orbital overlap and electron density, serving as an MO-based measure of bond order.
NBO-BD feature	Occupied bonding orbital between two atoms, describing shared electron density.

Abbreviations:
ELF: Electron Localization Function
LBO: Laplacian Bond Order
DI: Delocalization Index
MBO: Mayer Bond Order
NBO: Natural Bond Orbital
BD: Bonding orbital

2.2 Loss function

The loss function is composed of four distinct components: a global loss, a geometry prediction loss, a reconstruction loss (node, edge), and a right information keeping loss (node, edge).

$$
\begin{aligned}
\mathcal{L}_{\text{total}} &= 0.25 \times \mathcal{L}_{\text{global}} + 1.0 \times \mathcal{L}_{\text{reconstruction}} + 0.1 \times \mathcal{L}_{\text{keeping}} + 1.0 \times \mathcal{L}_{\text{geometry}} \\
\mathcal{L}_{\text{geometry}} &= 1.0 \times \mathcal{L}_{\text{PV}} + 1.0 \times \mathcal{L}_{\text{PD}} + 1.0 \times \mathcal{L}_{\text{RMSD}} + 1.0 \times \mathcal{L}_{\text{FAPE}} \\
\mathcal{L}_{\text{reconstruction}} &= 1.0 \times \mathcal{L}_{\text{node}} + 1.0 \times \mathcal{L}_{\text{edge}} \\
\mathcal{L}_{\text{keeping}} &= 1.0 \times \mathcal{L}_{\text{node}} + 1.0 \times \mathcal{L}_{\text{edge}}
\end{aligned}
\tag{S19}
$$

As indicated by the formulation, we deliberately avoided complex weighting schemes. The loss composition is straightforward, with only two specific coefficients assigned: a weight of 0.25 for the global loss and 0.1 for the preservation of uncorrupted information. The rationale behind these settings is as follows:

- **Global Loss Weight (0.25)** Because the global prediction task encompasses numerous specific sub-targets, we assigned it a reduced weight to prevent its gradients from disproportionately dominating the overall learning objective.
- **Uncorrupted Information Preservation Weight (0.1)** Many previous masked-modeling works focus exclusively on reconstructing masked features, often ignoring the uncorrupted ones. However, we argue that a robust encoder must holistically encode the entire molecule and explicitly learn to distinguish between intact and corrupted signals. Therefore, we assigned a modest weight of 0.1 to this preservation objective to encourage comprehensive representation learning.

2.3 Hyperparameters

Supplementary Table 3. Comprehensive hyperparameter settings for the main pre-training setup of qcGEM

Category	Hyperparameter	Setting
Encoder	Layers	16
Encoder	Attention Heads	8
Encoder	Global Head Dimension	32
Encoder	Node Head Dimension	32
Encoder	Edge Head Dimension	32
Encoder	Global Bottleneck Dim.	128
Encoder	Node Bottleneck Dim.	128
Encoder	Edge Bottleneck Dim.	128
Encoder	Spatial Cutoff	8.0
Decoder	Folding Optimizer Block	3 Times (6 xyz predictions)
Decoder	Distance Bin	[0.01, 0.05, 0.1, 0.12, 0.3, 0.5, 0.8, 1.0, 2.0, 3.0, 5.0, 8.0]
Model (Encoder + Decoder)	Normalization	LayerNorm
Model (Encoder + Decoder)	Initialization	None
Training	Global Mask Ratio	1.0 (training + inference)
Training	Node and Edge Mask Ratio	0.3 (training), 0.0 (inference)
Training	Node and Edge Replace Ratio	0.3 (training), 0.0 (inference)
Training	Grad Clip	None
Optimization	Learning Rate	$1 \times 10^{-4} \rightarrow 8 \times 10^{-5}$
Optimization	Optimizer	Adam
Optimization	Scheduler	None
Data	Split Method	Random
Data	Random Seed	42
Data	Data Shuffle	True
Loss Weighting	Global Inference Branch	Weight = 0.25
Loss Weighting	Structure Prediction Branch (FAPE + RMSD + Distance + Vector)	Weight = 1.0
Loss Weighting	Node and Edge Reconstruction Branch (For Masking and Replacing)	Weight = 1.0
Loss Weighting	Node and Edge Keeping Branch (For Keeping Information)	Weight = 0.1
Loss Weighting	Total Loss	$L = 0.25L_{\text{global}} + 1.0L_{\text{geomtry}} + 1.0L_{\text{reconstrute}} + 0.1L_{\text{keep}}$

2.4 Pre-training process

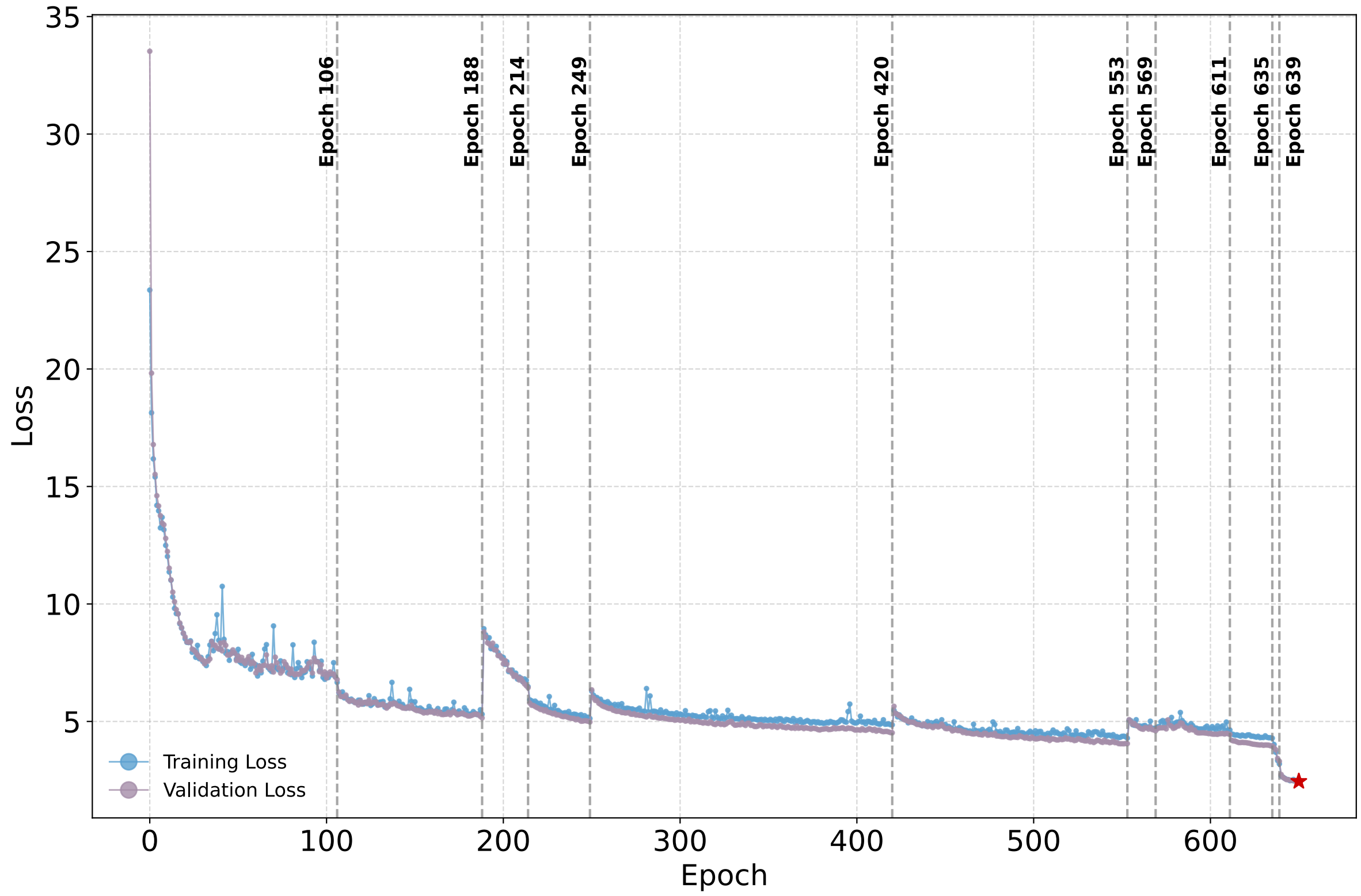

Supplementary Figure 2. Pre-training loss curves across different stages. The model underwent 11 stages of manual fine-tuning, during which training loss consistently declined, resulting in enhanced downstream performance. Gray dashed lines mark the checkpoints connecting consecutive stages. The model selected for representation evaluation (Epoch 650, or Checkpoint 649) is labeled with a red star.

Supplementary Table 4. Pre-training process

Stage	Device	Batch Size	Learning Rate	Saved Epoch	Task	Loss
1	4090	84	8×10^{-5}	106	Mask	All
2	4090	85	5×10^{-5}	188	Mask	woFAPE
3	4090	83	8×10^{-5}	214	Mask + Denoise	All
4	4090	83	8×10^{-5}	249	Mask + Denoise	woRMSD woRD woDistance
5	4090	85	5×10^{-5}	420	Mask + Denoise	woFPAE woRMSD
6	A100	32	5×10^{-5}	553	Mask + Denoise	woFPAE woRMSD
7	4090	16	5×10^{-5}	569	Mask + Denoise	woFPAE woRMSD
8	4090	12	5×10^{-5}	611	Mask + Denoise	woFPAE woRMSD
9	4090	12	3×10^{-5}	635	Mask + Denoise	woFPAE woRMSD
10	A100	86	3×10^{-4}	639	Mask + Denoise	woFPAE woRMSD
11	A100	86	1×10^{-4}	650	Mask + Denoise	woFPAE woRMSD

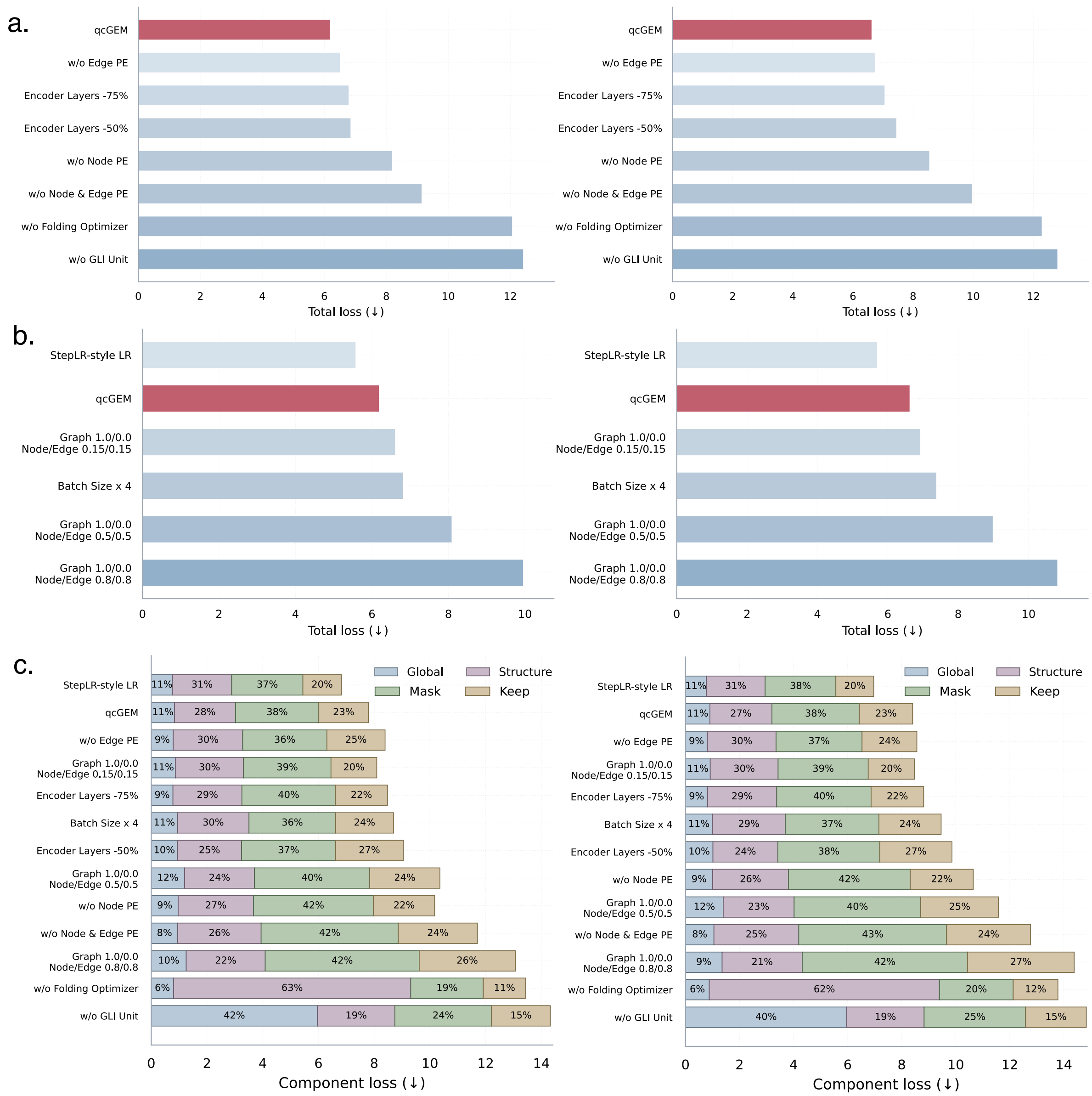

Supplementary Figure 3. Ablation studies on the architecture and training strategies of qcGEM. All model variants were evaluated over a 40-epoch training process. The baseline qcGEM model (highlighted in red) utilizes the complete architecture and is trained with a batch size of 16, without a learning rate (LR) scheduler. The default mask and replace ratios are set to 1.0/0.0 for the global representation, and 0.3/0.3 for nodes and edges. In all subfigures, the left panels present the best total or component loss achieved, while the right panels display the average loss computed over the final 10 epochs to indicate convergence stability. **a)** Impact of ablating key architectural components (*e.g.*, GLI Unit, folding optimizer, and positional encoding (PE)) on the total loss. **b)** Effects of varying training schemes, including learning rate schedules and different masking ratios, on the total loss. **c)** Detailed breakdown of individual loss components (Global, Structure, Mask, and Keep) across different experimental settings.

2.5 Pre-training results

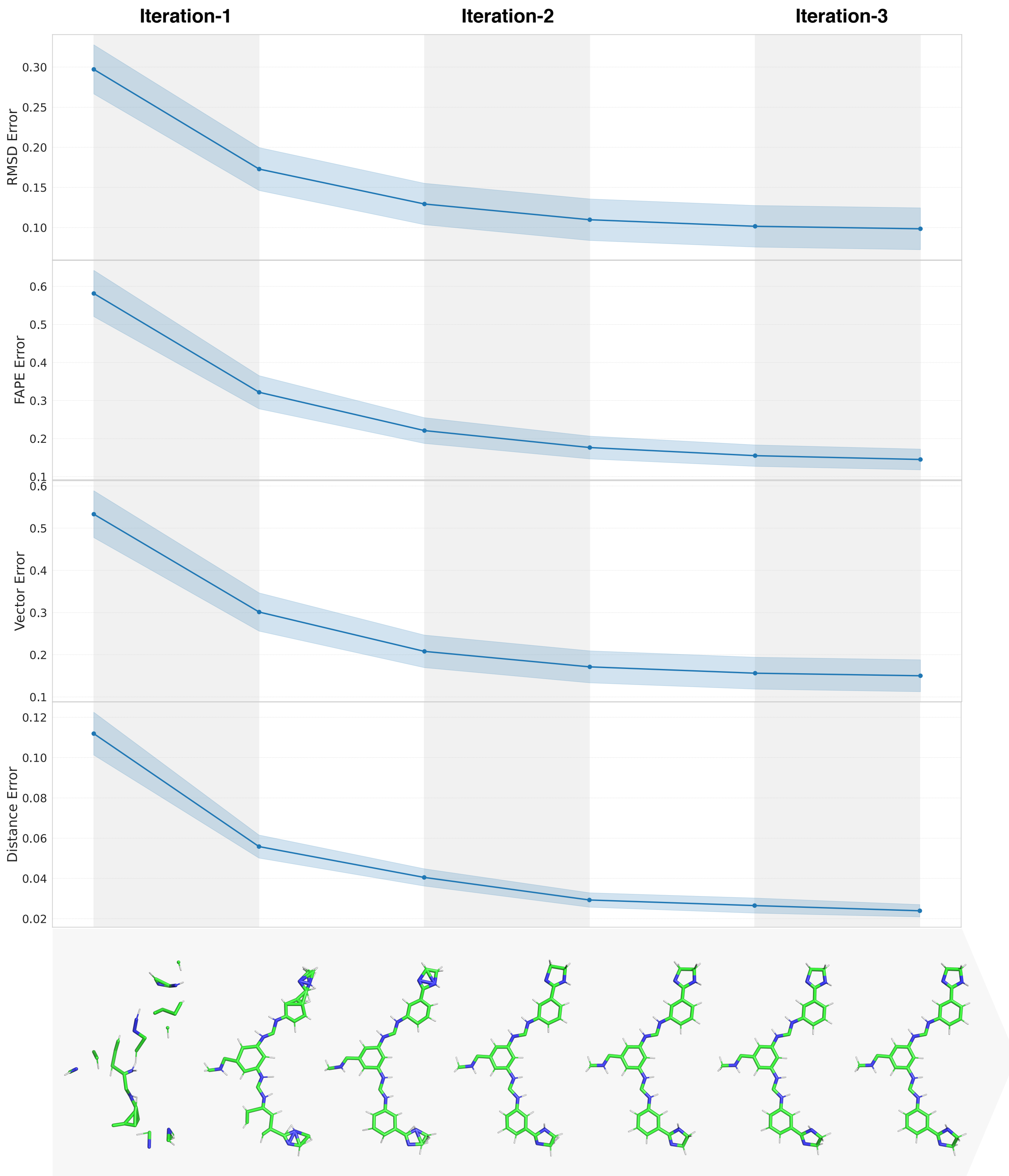

Supplementary Figure 4. Effective structural reconstruction by the folding optimizer. During the pre-training of qcGEM, the folding optimizer learns to optimize the denoised molecular structure, through the comprehensive use of multiple geometry loss terms.

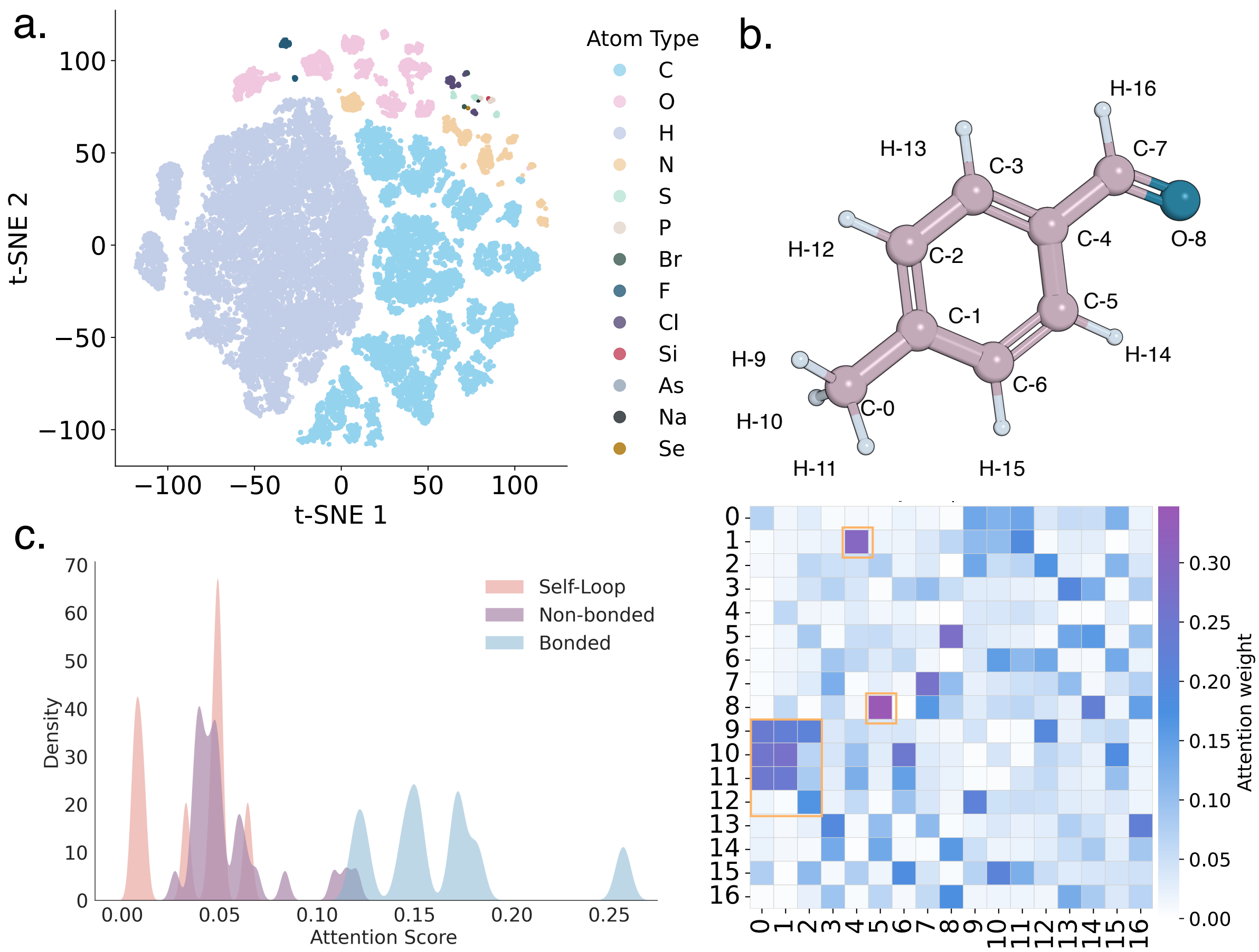

Supplementary Figure 5. Analysis of the embedding of qcGEM. a) t-SNE visualization of atom-level embeddings learned by qcGEM, colored by atom type, showing clear clustering by chemical element. **b)** Showcase of edge-level embeddings on an exemplar molecule (top). In the attention map (bottom), non-local interactions captured by qcGEM are highlighted by orange boxes. **c)** Distribution of attention scores for self-loops as well as bonded and non-bonded atom pairs.

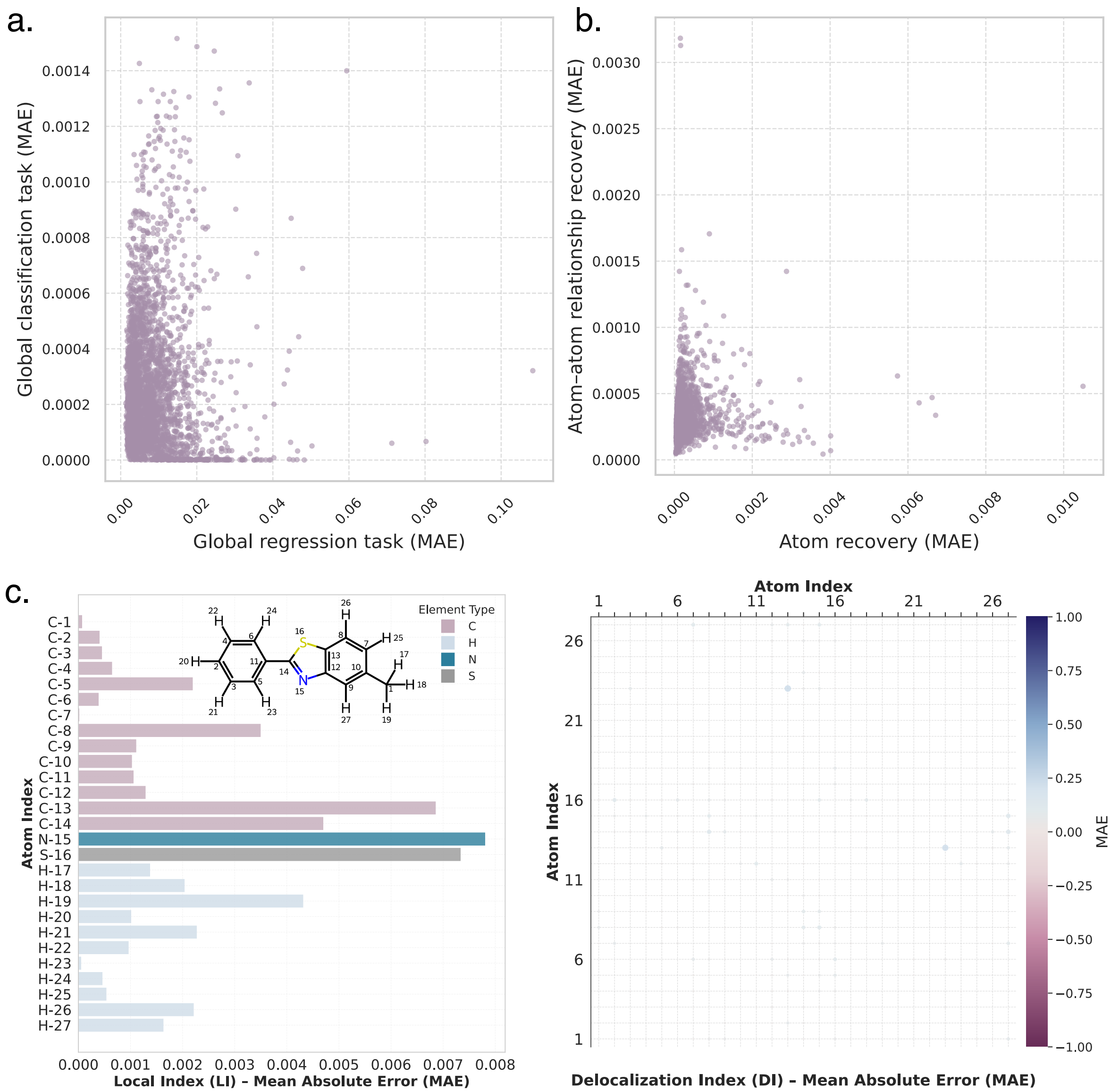

Supplementary Figure 6. Recovery of quantum descriptors from the learned representation. a) Errors in the reconstruction of global properties. Each point refers to a compound, with the horizontal and vertical axes representing regression and classification tasks, respectively. **b)** Errors in the reconstruction of detailed quantum chemical information. Here, the horizontal and vertical axes represent node-level (*i.e.* atom) and edge-level (*i.e.* atom-atom relationship) quantum descriptors, respectively. **c)** Reconstruction of local features for an exemplar molecule. Errors of the localization index (LI, a node-level quantum feature) are shown in the left panel as a bar plot, whereas those of the delocalization index (DI, an edge-level quantum feature) are shown in the right panel as a heat map. The mean absolute error (MAE) is uniformly taken as the evaluation metric in this figure.

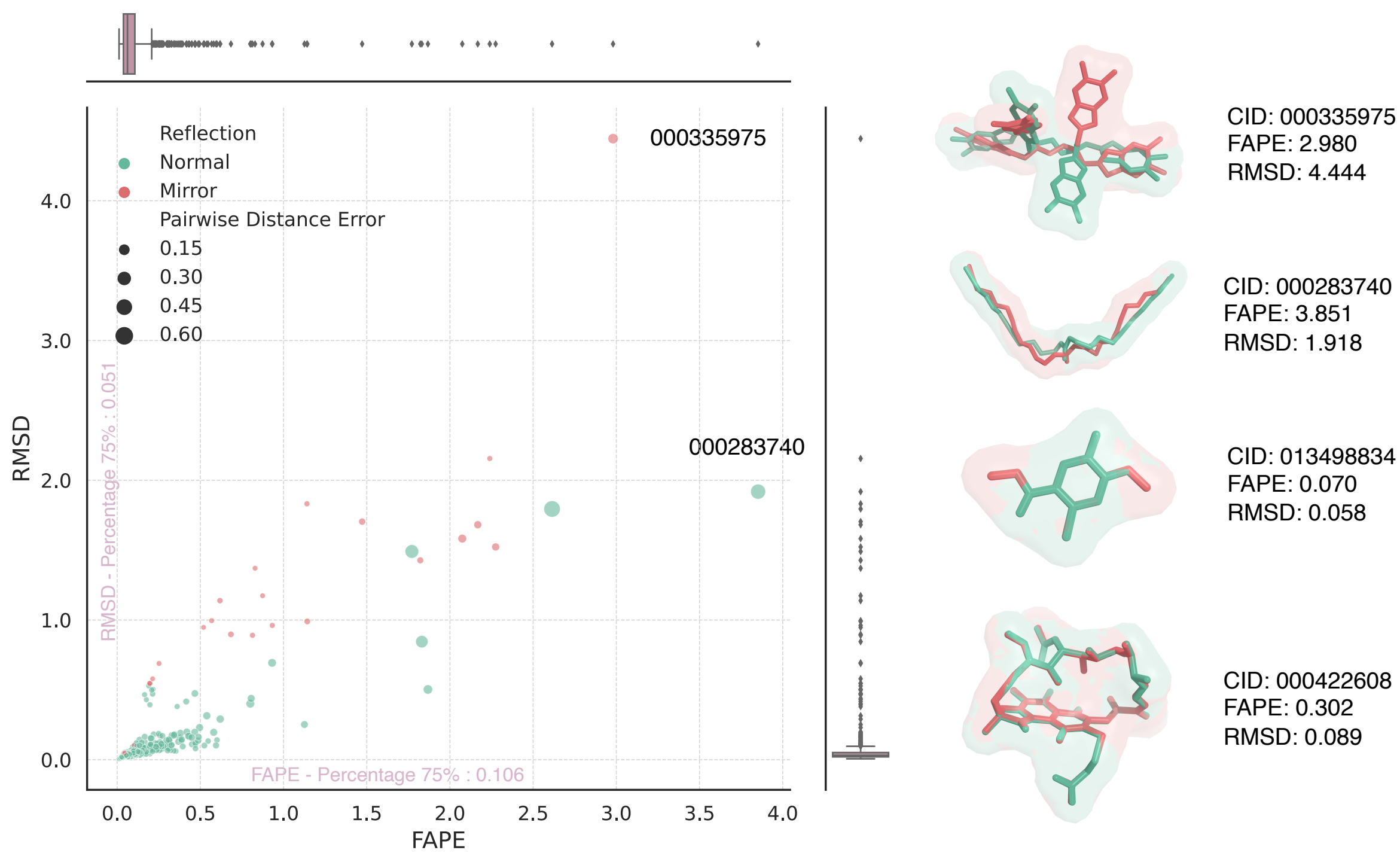

Supplementary Figure 7. Reconstruction of molecular geometry from the learned representation. The scatter plot in the left panel presents the FAPE, RMSD, and PD (*i.e.* pairwise distance error) loss terms for molecules generated from the structure decoder, where the horizontal and vertical axes stand for FAPE and RMSD values, respectively, and each dot refers to one generated molecule, with dot size proportional to PD value. The PD term alone could not provide effective constraining, since molecules with large structure deviations (in RMSD and FAPE) frequently have small errors. Moreover, RMSD is ineffective in identifying mirror structures (*i.e.* stereoisomers). Figures in the right panel showcase exemplar generated molecules, with FAPE and RMSD values indicated aside. Clearly, the joint use of FAPE and RMSD ensures proper evaluation of structure generation. In general, the structure decoder of qcGEM allows successful generation of molecular structures for most tested molecules, as evidenced by the low FAPE and RMSD values.

3 Supplementary Results for Evaluation

3.1 Evaluated methods

Supplementary Table 5. Representation methods tested in this study

Method	Type	Data demand	Dimension
qcGEM	Neural Embedding	300 thousand	128/128/128
ECFP[1]	Molecular Fingerprint	/	384
FCFP[1]	Molecular Fingerprint	/	384
AvalonFP[2]	Molecular Fingerprint	/	384
TTGEN[3]	Molecular Fingerprint	/	384
APGEN[4]	Molecular Fingerprint	/	384
RDKFP[5]	Molecular Fingerprint	/	384
MACCS[6]	Molecular Fingerprint	/	384
LayeredFP[5]	Molecular Fingerprint	/	384
PatternFP[5]	Molecular Fingerprint	/	384
Molformer[7]	Neural Embedding	1.1 billion	768
GROVER-Base[8]	Neural Embedding	11 million	3,400
GROVER-Large[8]	Neural Embedding	11 million	5,000
MoleBERT[9]	Neural Embedding	2 million	300
UniMol-G[10]	Neural Embedding	12 million + 19 million	512
UniMol-GA[10]	Neural Embedding	12 million + 19 million	1,024
GCC[11]	Neural Embedding	100 thousand	128
GPT-GNN[12]	Neural Embedding	100 thousand	128
GraphCL[13]	Neural Embedding	100 thousand	128
InfoGraph[14]	Neural Embedding	100 thousand	128
JOAO[15]	Neural Embedding	100 thousand	128
JOAOv2[15]	Neural Embedding	100 thousand	128
PosPred[16]	Neural Embedding	100 thousand	128
3D-EMGP[16]	Neural Embedding	100 thousand	128
3D-InfoMax[17]	Neural Embedding	100 thousand	128
AttrMask[18]	Neural Embedding	100 thousand	128
EdgePred[19]	Neural Embedding	100 thousand	128
GraphMVP[20]	Neural Embedding	250 thousand	300
MolCLR-GCN[21]	Neural Embedding	10 million	512
MolCLR-GIN[21]	Neural Embedding	10 million	512
TransM[22]	Neural Embedding	3.37 million	768
MPG[23]	Neural Embedding	11 million	768
KANO[24]	Neural Embedding	250 thousand	300

Basic characteristics of different representation methods are shown in the table. Fingerprint-based representations are generated from pre-defined rules, whereas other approaches are learned from data.

3.2 Evaluation datasets

Supplementary Table 6. Molecular property datasets

Dataset	Description	Task Type	Metric
FreeSolv	Experimental and calculated hydration free energies of small molecules in water.	Regression	RMSE
ESOL	Water solubility data (log solubility in mols per litre) for common organic small molecules.	Regression	RMSE
Lipophilicity	Experimental results of octanol/water distribution coefficient (logD at pH 7.4).	Regression	RMSE
BBBP	Binary labels of blood-brain barrier penetration (permeability).	Classification	ROC-AUC
BACE	Quantitative (IC50) and qualitative (binary label) binding results for a set of inhibitors of human β-secretase 1 (BACE-1).	Classification	ROC-AUC
Bioavailability	Extent and rate of drug absorption into systemic circulation.	Classification	ROC-AUC
Caco2	In vitro drug permeability via Caco-2 cells, simulating intestinal absorption.	Regression	RMSE
HIA	Human intestinal absorption (HIA), reflecting a drug's ability to enter the bloodstream after oral administration.	Classification	ROC-AUC
PAMPA	PAMPA estimates passive permeability across artificial membranes, widely used for absorption prediction.	Classification	ROC-AUC
Pgp	Predict whether a compound inhibits P-glycoprotein (Pgp), affecting drug bioavailability and resistance.	Classification	ROC-AUC
Solubility AqSolDB	Estimate the log-scale solubility of a drug candidate in water, crucial for oral bioavailability.	Regression	RMSE
VDss	Estimate drug distribution extent in tissues vs. blood at steady state (VDss).	Regression	RMSE
BBB	Predict blood-brain barrier (BBB) penetration for central nervous system (CNS) drug design.	Classification	ROC-AUC
Clearance Hepatocyte	Predict drug clearance rate from hepatocyte assays.	Regression	RMSE
Clearance Microsome	Predict drug clearance rate from microsome assays.	Regression	RMSE
Half Life	Predict drug half-life, reflecting how long a drug stays active in the body.	Regression	RMSE
CYP1A2 Veith	CYP1A2 inhibition prediction related to metabolism and toxicity risk.	Classification	ROC-AUC

Dataset	Description	Task Type	Metric
CYP2C19 Veith	CYP2C19 inhibition prediction to assess drug metabolism risks.	Classification	ROC-AUC
CYP2C9 Substrate	CYP2C9 substrate classification to evaluate compound metabolism.	Classification	ROC-AUC
CYP2C9 Veith	CYP2C9 inhibition prediction related to drug and endogenous metabolism.	Classification	ROC-AUC
CYP2D6 Substrate	CYP2D6 substrate classification related to hepatic and CNS metabolism.	Classification	ROC-AUC
CYP2D6 Veith	CYP2D6 inhibition prediction related to metabolism and CNS effects.	Classification	ROC-AUC
CYP3A4 Substrate	CYP3A4 substrate classification to assess metabolism of xenobiotics.	Classification	ROC-AUC
CYP3A4 Veith	CYP3A4 inhibition prediction related to drug metabolism and clearance.	Classification	ROC-AUC
Ames	Ames test-based mutagenicity classification for assessing genetic toxicity.	Classification	ROC-AUC
Carcinogens	Carcinogenicity prediction to assess cancer-causing potential.	Classification	ROC-AUC
DILI	DILI prediction to assess risk of drug-induced liver toxicity.	Classification	ROC-AUC
hERG Blockers	hERG inhibition prediction to assess cardiotoxicity risk.	Classification	ROC-AUC
LD50	LD_{50}-based acute toxicity prediction to assess lethal dose risks.	Regression	RMSE
SkinReac	Skin sensitization prediction based on rLLNA to assess allergic response risk.	Classification	ROC-AUC
BACEreg	Identical sample set to BACE with raw continuous IC50 values. **This set is only reported for analysis but excluded from final summary to avoid redundancy.**	Regression	RMSE
Log D	pH-adjusted lipophilicity (distribution coefficient) of compounds	Regression	RMSE
Water Solubility	Aqueous solubility of compounds in water	Regression	RMSE
BBB(PharmaBench)	Blood-brain barrier penetration classification	Classification	ROC-AUC
PPB	Plasma protein binding percentage in human plasma	Regression	RMSE
CYP 2C9	Inhibitory activity toward cytochrome P450 2C9	Regression	RMSE
CYP 2D6	Inhibitory activity toward cytochrome P450 2D6	Regression	RMSE
CYP 3A4	Inhibitory activity toward cytochrome P450 3A4	Regression	RMSE

**Dataset**	**Description**	**Task Type**	**Metric**
HLMC	Human liver microsomal clearance	Regression	RMSE
RLMC	Rat liver microsomal clearance	Regression	RMSE
MLMC	Mouse liver microsomal clearance	Regression	RMSE
AMES(PharmaBench)	Ames mutagenicity toxicity classification	Classification	ROC-AUC

Supplementary Table 7. MoleculeACE datasets

ChEMBL ID	Target	Abbreviation	Experimental Category	Protein Class
CHEMBL1871	Androgen Receptor	AR	K_i	NR
CHEMBL218	Cannabinoid receptor 1	CB1	EC_{50}	GPCR
CHEMBL244	Coagulation factor X	FX	K_i	Protease
CHEMBL236	Delta opioid receptor	DOR	K_i	GPCR
CHEMBL234	Dopamine D3 receptor	D3R	K_i	GPCR
CHEMBL219	Dopamine D4 receptor	D4R	K_i	GPCR
CHEMBL238	Dopamine transporter	DAT	K_i	Other
CHEMBL4203	Dual specificity protein kinase	CLK4	K_i	Kinase
CHEMBL2047	Farnesoid X receptor	FXR	EC_{50}	NR
CHEMBL4616	Ghrelin receptor	GHSR	EC_{50}	GPCR
CHEMBL2034	Glucocorticoid receptor	GR	K_i	NR
CHEMBL262	Glycogen synthase kinase-3 beta	GSK3	K_i	Kinase
CHEMBL231	Histamine H1 receptor	HRH1	K_i	GPCR
CHEMBL264	Histamine H3 receptor	HRH3	K_i	GPCR
CHEMBL2835	Janus kinase 1	JAK1	K_i	Kinase
CHEMBL2971	Janus kinase 2	JAK2	K_i	Kinase
CHEMBL237	Kappa opioid receptor agonism	KOR(a)	EC_{50}	GPCR
CHEMBL237	Kappa opioid receptor inhibition	KOR(i)	K_i	GPCR
CHEMBL233	μ-opioid receptor	MOR	K_i	GPCR
CHEMBL4792	Orexin receptor 2	OX2R	K_i	GPCR
CHEMBL239	Peroxisome proliferator-activated receptor alpha	PPARα	EC_{50}	NR
CHEMBL3979	Peroxisome proliferator-activated receptor delta	PPARδ	EC_{50}	NR
CHEMBL235	Peroxisome proliferator-activated receptor gamma	PPARγ	EC_{50}	NR
CHEMBL4005	PI3-kinase p110-alpha subunit	PIK3CA	K_i	Transferase
CHEMBL2147	Serine/threonine-protein kinase	PIM1	K_i	Kinase
CHEMBL214	Serotonin 1a receptor	5-HT1A	K_i	GPCR
CHEMBL228	Serotonin transporter	SERT	K_i	Other
CHEMBL287	Sigma opioid receptor	SOR	K_i	Other
CHEMBL204	Thrombin	Thrombin	K_i	Protease
CHEMBL1862	Tyrosine-protein kinase	ABL1	K_i	Kinase

NR denotes nuclear receptor, while GPCR refers to G-protein coupled receptor.

Supplementary Table 8. Protein-ligand interaction datasets

Dataset	Description	Task Type	Metric
Human	Derived from the DrugBank database, containing experimentally validated compound–protein interactions involving human proteins.	Classification	ROC-AUC
C.elegans	Contain interactions between compounds and *C. elegans* proteins, sourced from curated public databases.	Classification	ROC-AUC
KIBA	A model-based integration of bioactivities into a unified drug-target interaction matrix.	Regression	RMSE
DAVIS	The interaction of 72 kinase inhibitors with 442 kinases covering >80% of the human catalytic protein kinome.	Regression	RMSE
BindingDB	BindingDB is a public database of experimentally measured binding affinities between drug-target proteins and small molecules.	Classification	ROC-AUC

Supplementary Table 9. Opioid-type datasets

Dataset	Protein Name	ChEMBL ID	Task Type	Metric
CYP2D6	Cytochrome P450 2D6	ChEMBL 289	Regression	RMSE
CYP3A4	Cytochrome P450 3A4	ChEMBL 340	Regression	RMSE
DOR	δ-opioid receptor	ChEMBL 236	Regression	RMSE
KOR	κ-opioid receptor	ChEMBL 237	Regression	RMSE
MDR1	Multidrug resistance protein 1 (P-glycoprotein 1)	ChEMBL 4302	Regression	RMSE
MOR	μ-opioid receptor	ChEMBL 233	Regression	RMSE

3.3 Evaluation results

3.3.1 Molecular property prediction

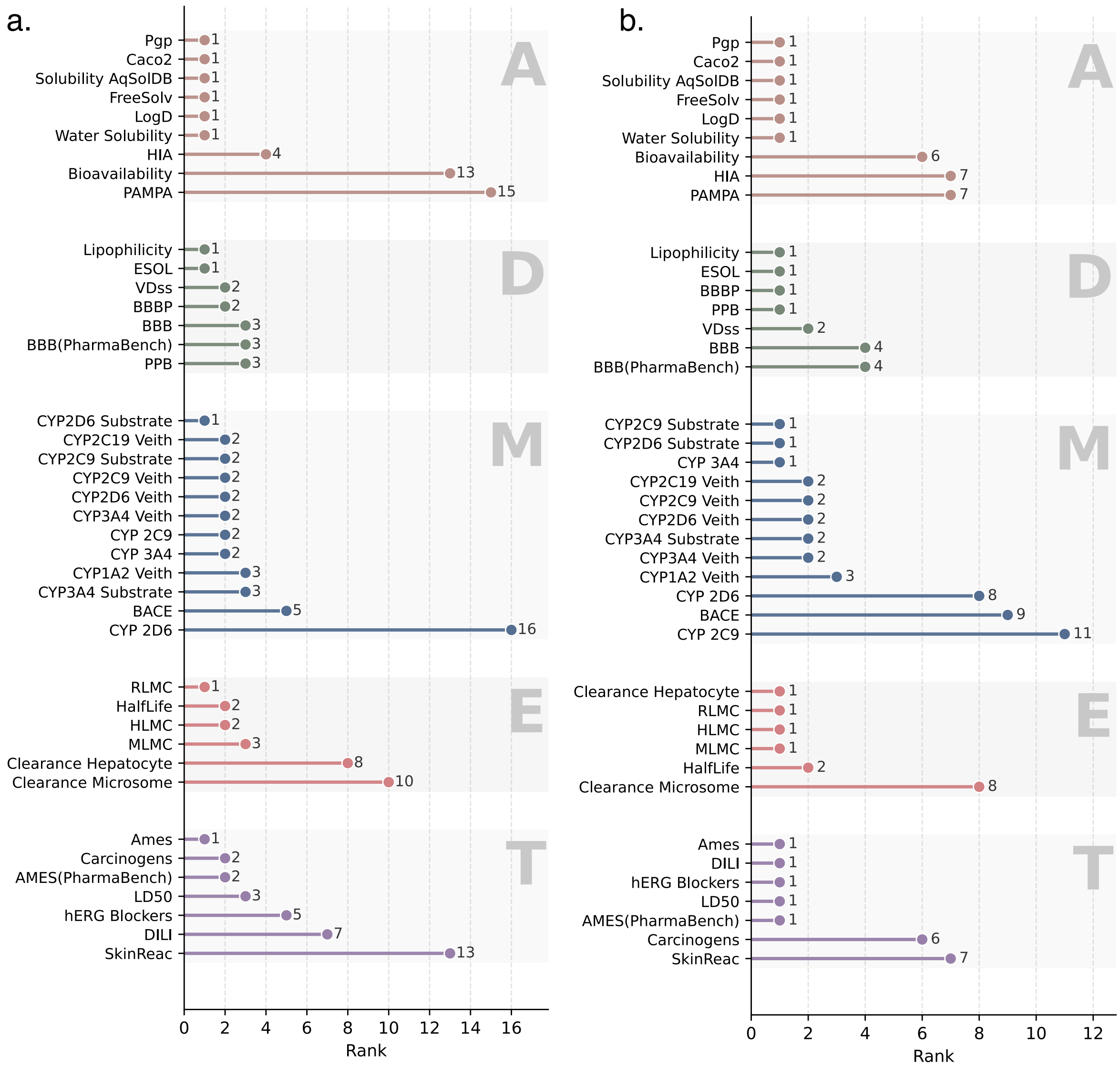

Supplementary Figure 8. Detailed ranking information of qcGEM in individual ADMET tasks. a) In the scaffold split setting, qcGEM rankings are shown from top to bottom for absorption (A), distribution (D), metabolism (M), excretion (E), and toxicity (T). **b)** Results are shown for the random split setting following the same convention.

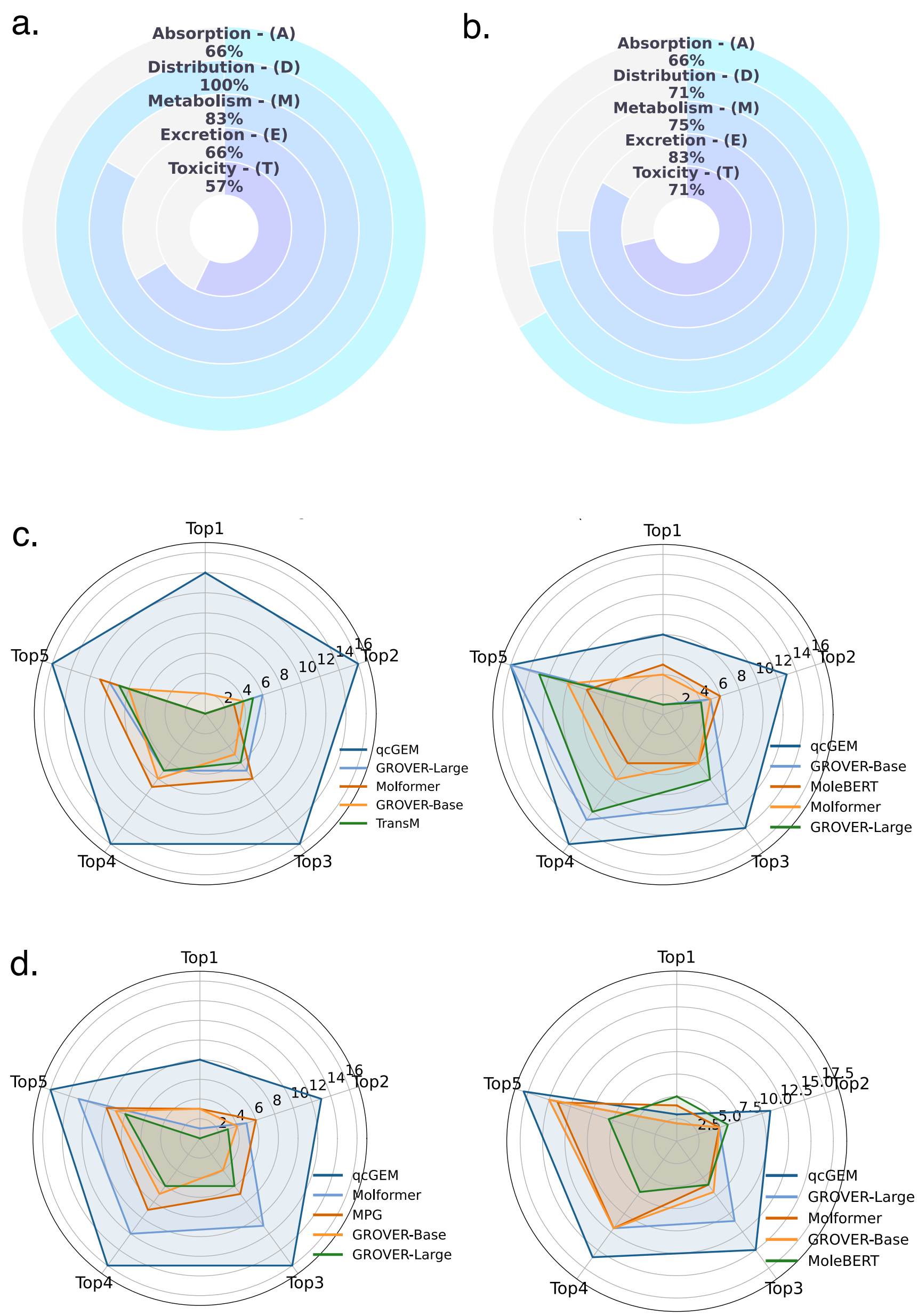

Supplementary Figure 9. Evaluation of qcGEM across the 5 ADMET task categories and 2 data split settings. **a)** Proportion of tasks in which qcGEM achieves the top 3 ranking across ADMET task categories for all molecules in the scaffold split setting. Here, ADMET tasks are classified into absorption (A), distribution (D), metabolism (M), excretion (E), and toxicity (T). **b)** Proportion of tasks in which qcGEM achieves the top 3 ranking across ADMET task categories for all molecules in the random split setting. **c-d)** Radar plots of the overall performance profiles for the top methods in the random split (**c**) and scaffold split (**d**) settings, with results of regression and classification tasks shown on the left and right, respectively. These plots supplement Figure 2d, providing the split-specific radar charts.

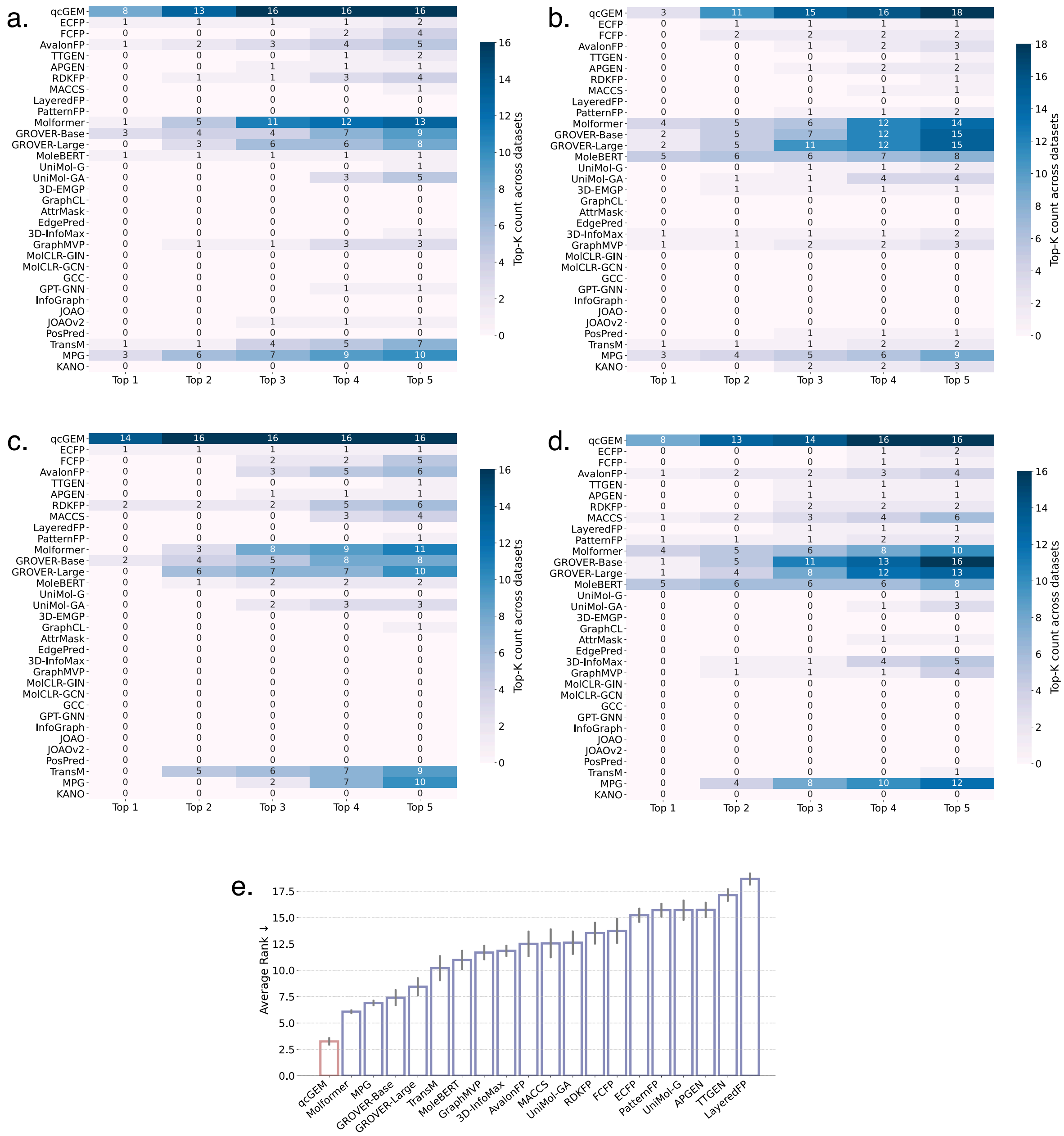

Supplementary Figure 10. Statistics of the top 1-5 rankings for all methods in ADMET tasks. **a**) Regression tasks in the scaffold split setting. **b**) Classification tasks in the scaffold split setting. **c**) Regression tasks in the random split setting. **d**) Classification tasks in the random split setting. **e**) The overall average rank of the top methods. Here, the average rank is calculated across data split settings and ADMET task categories, with the mean and standard errors presented in the bar plot.

Supplementary Table 10. Molecular property regression task (scaffold split) – Part I

Method	FreeSolv	ESOL	Lipophilicity	Caco2	Solubility AqSolDB
qcGEM	2.40639 ± 0.77910	0.79610 ± 0.04258	0.66334 ± 0.03645	0.48382 ± 0.04524	0.96784 ± 0.06764
ECFP	4.33168 ± 0.67484	1.81739 ± 0.27535	0.97741 ± 0.07489	0.83895 ± 0.16649	1.61814 ± 0.11770
FCFP	3.73943 ± 0.39733	1.64548 ± 0.24601	0.93437 ± 0.07242	0.72475 ± 0.17394	1.53320 ± 0.09067
AvalonFP	2.97680 ± 0.58118	1.42139 ± 0.21286	1.00543 ± 0.10069	0.91229 ± 0.25826	1.36693 ± 0.05424
TTGEN	4.57039 ± 0.71041	1.44532 ± 0.17098	1.06656 ± 0.12179	0.93071 ± 0.23914	1.42271 ± 0.08645
APGEN	3.98574 ± 0.49472	1.28746 ± 0.05621	1.00667 ± 0.08277	0.89184 ± 0.20918	1.30696 ± 0.04092
RDKFP	3.83381 ± 0.40907	1.67591 ± 0.26831	0.95674 ± 0.10695	0.65578 ± 0.07952	1.54048 ± 0.10102
MACCS	2.62950 ± 0.14820	1.30693 ± 0.25565	0.93140 ± 0.08849	0.76264 ± 0.19702	1.31032 ± 0.01082
LayeredFP	3.48682 ± 0.42621	1.30095 ± 0.14239	1.16168 ± 0.10768	0.97985 ± 0.26536	1.45059 ± 0.07127
PatternFP	3.91618 ± 0.60044	1.34797 ± 0.10101	1.02549 ± 0.07958	0.79646 ± 0.19077	1.38711 ± 0.06809
Molformer	2.88781 ± 0.53860	0.94180 ± 0.09944	0.74328 ± 0.04133	0.52618 ± 0.04886	1.03667 ± 0.06063
GROVER-Base	2.62741 ± 0.90487	1.00498 ± 0.01899	0.78457 ± 0.03829	0.67852 ± 0.05450	1.12080 ± 0.06993
GROVER-Large	2.99455 ± 0.88852	0.96307 ± 0.09002	0.70419 ± 0.08423	0.84747 ± 0.52992	1.00908 ± 0.08091
MoleBERT	3.03401 ± 0.78475	1.37038 ± 0.13472	0.84224 ± 0.08639	0.61098 ± 0.04402	1.43424 ± 0.04284
UniMol-G	3.06279 ± 0.47857	1.09605 ± 0.08057	1.02488 ± 0.09944	0.54225 ± 0.06269	1.10144 ± 0.08515
UniMol-GA	2.72047 ± 0.30748	0.99114 ± 0.11321	0.96680 ± 0.10296	0.53292 ± 0.06812	1.05127 ± 0.05818
3D-EMGP	4.56136 ± 0.69964	2.51409 ± 0.31857	1.19498 ± 0.12046	0.88328 ± 0.15113	2.38923 ± 0.26883
GraphCL	4.63226 ± 0.63226	1.62132 ± 0.15215	0.96410 ± 0.08436	0.70889 ± 0.08815	1.45094 ± 0.05207
AttrMask	3.87336 ± 0.74288	1.86333 ± 0.14844	1.09813 ± 0.11488	0.67464 ± 0.07548	1.82350 ± 0.10609
EdgePred	4.44407 ± 1.42198	2.02109 ± 0.18368	1.11928 ± 0.11723	0.71649 ± 0.08405	2.02125 ± 0.12135
3D-InfoMax	2.76988 ± 0.57364	1.08493 ± 0.11330	0.90792 ± 0.08228	0.54559 ± 0.06125	1.13324 ± 0.07979
GraphMVP	2.46996 ± 0.44680	1.07502 ± 0.12541	0.87104 ± 0.07406	0.52853 ± 0.01450	1.17682 ± 0.11729
MolCLR-GIN	4.00074 ± 0.69285	1.65176 ± 0.13098	1.13328 ± 0.13771	0.69744 ± 0.06378	1.62026 ± 0.09239
MolCLR-GCN	3.82983 ± 0.52814	1.60059 ± 0.09852	1.12832 ± 0.10901	0.68286 ± 0.07697	1.57266 ± 0.10219
GCC	3.36896 ± 0.49802	1.24257 ± 0.13185	1.08588 ± 0.11917	0.63597 ± 0.07780	1.35442 ± 0.06794
GPT-GNN	3.58078 ± 0.62244	1.32426 ± 0.10724	1.04372 ± 0.10857	0.96272 ± 0.07138	1.33579 ± 0.09010
InfoGraph	4.33888 ± 0.51741	1.70161 ± 0.18832	1.15448 ± 0.11228	0.67749 ± 0.08246	1.56965 ± 0.09420
JOAO	3.56206 ± 1.02112	1.30513 ± 0.15620	1.12390 ± 0.11785	0.90992 ± 0.16347	1.42521 ± 0.09042
JOAOv2	3.62090 ± 0.96602	1.22360 ± 0.13152	1.10060 ± 0.12204	0.70422 ± 0.09591	1.27820 ± 0.08150
PosPred	3.56029 ± 0.59955	1.45817 ± 0.29940	1.13007 ± 0.13411	1.18714 ± 0.38752	1.69584 ± 0.25858
TransM	2.53054 ± 0.77159	1.12285 ± 0.09963	0.94437 ± 0.09011	0.68513 ± 0.17669	1.16482 ± 0.06416
MPG	2.92944 ± 0.47057	1.00826 ± 0.12348	0.80254 ± 0.03943	0.51949 ± 0.05755	1.12709 ± 0.07022
KANO	3.36221 ± 0.74825	1.16311 ± 0.11978	1.09621 ± 0.10812	0.54085 ± 0.05533	1.30763 ± 0.07955

Prediction is evaluated by RMSE. For each method, the mean and standard deviation are reported for the evaluation metrics.

Supplementary Table 11. Molecular property regression task (scaffold split) – Part II

Method	VDss	Clearance Hepatocyte	Clearance Microsome	Half Life	LD50
qcGEM	0.01573 ± 0.01984	0.29785 ± 0.01327	0.25089 ± 0.02205	0.02753 ± 0.02182	0.70783 ± 0.02724
ECFP	0.01805 ± 0.02354	0.29554 ± 0.03085	0.24146 ± 0.02832	0.02791 ± 0.02229	0.76627 ± 0.02376
FCFP	0.01741 ± 0.02274	0.29297 ± 0.01661	0.25311 ± 0.02379	0.02786 ± 0.02221	0.76644 ± 0.02944
AvalonFP	0.01680 ± 0.02139	0.30290 ± 0.02701	0.25572 ± 0.02676	0.02781 ± 0.02209	0.69322 ± 0.02814
TTGEN	0.01824 ± 0.02356	0.29888 ± 0.02065	0.24454 ± 0.02732	0.02785 ± 0.02197	0.78283 ± 0.03724
APGEN	0.01707 ± 0.02163	0.31426 ± 0.01784	0.25894 ± 0.01969	0.02771 ± 0.02204	0.76191 ± 0.03110
RDKFP	0.01685 ± 0.02154	0.30179 ± 0.02181	0.24909 ± 0.02314	0.02775 ± 0.02230	0.71384 ± 0.03260
MACCS	0.01701 ± 0.02176	0.30210 ± 0.02986	0.25277 ± 0.02503	0.02877 ± 0.02146	0.73484 ± 0.03591
LayeredFP	0.01856 ± 0.02448	0.29815 ± 0.02258	0.25071 ± 0.02348	0.02793 ± 0.02223	0.75438 ± 0.01819
PatternFP	0.01813 ± 0.02350	0.30375 ± 0.01802	0.25268 ± 0.02390	0.02801 ± 0.02224	0.73390 ± 0.03062
Molformer	0.01614 ± 0.01992	0.29234 ± 0.02092	0.24440 ± 0.02496	0.02788 ± 0.02232	0.70473 ± 0.02518
GROVER-Base	0.01569 ± 0.02000	0.28999 ± 0.02781	0.25121 ± 0.02960	0.02734 ± 0.02266	0.77780 ± 0.03940
GROVER-Large	0.01568 ± 0.01923	0.29425 ± 0.02678	0.25059 ± 0.03324	0.02812 ± 0.02307	0.71392 ± 0.03029
MoleBERT	0.01835 ± 0.02440	0.29555 ± 0.03237	0.25594 ± 0.02021	0.02801 ± 0.02229	0.79174 ± 0.03426
UniMol-G	0.01737 ± 0.02193	0.31386 ± 0.01914	0.25003 ± 0.03120	0.02828 ± 0.02234	0.75851 ± 0.02930
UniMol-GA	0.01716 ± 0.02168	0.31193 ± 0.01743	0.24798 ± 0.03235	0.02807 ± 0.02232	0.73875 ± 0.03200
3D-EMGP	0.01926 ± 0.02472	0.32572 ± 0.02425	0.29169 ± 0.06121	0.02904 ± 0.02242	0.87365 ± 0.03511
GraphCL	0.01776 ± 0.02308	0.29880 ± 0.00880	0.26173 ± 0.03469	0.02829 ± 0.02225	0.78185 ± 0.02900
AttrMask	0.01884 ± 0.02458	0.32525 ± 0.02356	0.27000 ± 0.02298	0.02827 ± 0.02232	0.85022 ± 0.03076
EdgePred	0.01889 ± 0.02458	0.31961 ± 0.02169	0.26444 ± 0.02154	0.02832 ± 0.02217	0.82289 ± 0.02404
3D-InfoMax	0.01738 ± 0.02215	0.30154 ± 0.01844	0.25774 ± 0.02809	0.02808 ± 0.02190	0.73420 ± 0.02970
GraphMVP	0.01769 ± 0.02318	0.30046 ± 0.02339	0.25361 ± 0.02932	0.02802 ± 0.02221	0.74075 ± 0.02731
MolCLR-GIN	0.01894 ± 0.02446	0.32583 ± 0.02419	0.26895 ± 0.02113	0.02837 ± 0.02237	0.80495 ± 0.02294
MolCLR-GCN	0.01902 ± 0.02437	0.32539 ± 0.02423	0.26657 ± 0.02152	0.02809 ± 0.02247	0.82576 ± 0.02560
GCC	0.01865 ± 0.02462	0.32726 ± 0.01892	0.26629 ± 0.02175	0.02832 ± 0.02232	0.76973 ± 0.02347
GPT-GNN	0.01979 ± 0.02397	0.32044 ± 0.02523	0.26513 ± 0.03331	0.03054 ± 0.02224	0.79540 ± 0.01608
InfoGraph	0.01912 ± 0.02440	0.32035 ± 0.02757	0.26032 ± 0.02356	0.02884 ± 0.02221	0.85155 ± 0.02556
JOAO	0.02322 ± 0.02273	0.33791 ± 0.04999	0.26280 ± 0.02277	0.05729 ± 0.03298	0.82328 ± 0.01905
JOAOv2	0.01948 ± 0.02365	0.32008 ± 0.03076	0.26464 ± 0.02105	0.02849 ± 0.02269	0.80504 ± 0.01288
PosPred	0.10371 ± 0.08882	0.32676 ± 0.02533	0.47704 ± 0.41781	0.18738 ± 0.19028	1.02727 ± 0.29040
TransM	0.01661 ± 0.02129	0.30347 ± 0.02420	0.25194 ± 0.02548	0.02804 ± 0.02172	0.75302 ± 0.01711
MPG	0.01720 ± 0.02186	0.28631 ± 0.02421	0.24261 ± 0.02676	0.02845 ± 0.02205	0.71205 ± 0.03213
KANO	0.01901 ± 0.02478	0.32638 ± 0.02486	0.26851 ± 0.02195	0.02827 ± 0.02234	0.79594 ± 0.01951

Prediction is evaluated by RMSE. For each method, the mean and standard deviation are reported for the evaluation metrics.

Supplementary Table 12. Molecular property regression task (scaffold split) – Part III

Method	BACEreg	CYP 2C9	CYP 2D6	CYP 3A4	LogD
qcGEM	0.84200 ± 0.09400	13.87273 ± 2.57187	16.39443 ± 1.00552	15.75368 ± 2.43405	0.73572 ± 0.03211
ECFP	0.85555 ± 0.08242	14.80181 ± 2.38350	15.95698 ± 1.00695	16.62031 ± 2.96193	1.04023 ± 0.03631
FCFP	0.78076 ± 0.05189	14.66210 ± 2.59678	15.94621 ± 0.70920	16.13795 ± 2.90944	0.99681 ± 0.04723
AvalonFP	0.85959 ± 0.03960	14.63053 ± 2.74949	15.54995 ± 0.71351	16.78990 ± 2.77810	0.99233 ± 0.04855
TTGEN	0.84013 ± 0.03954	15.16429 ± 2.81407	16.47013 ± 0.68513	16.68259 ± 2.81081	1.08354 ± 0.05389
APGEN	0.83566 ± 0.06783	14.27041 ± 2.80854	16.87029 ± 0.82450	16.08243 ± 3.25528	1.09159 ± 0.05515
RDKFP	0.89194 ± 0.06587	14.53371 ± 2.64917	16.06851 ± 1.63369	16.64615 ± 3.15537	1.05447 ± 0.06231
MACCS	0.96188 ± 0.05593	14.53137 ± 2.28572	16.58992 ± 1.02456	16.52305 ± 3.20641	1.07435 ± 0.05067
LayeredFP	0.94594 ± 0.09521	14.97568 ± 2.45307	16.49205 ± 0.62454	16.92413 ± 2.81403	1.22786 ± 0.04763
PatternFP	0.86081 ± 0.07444	14.58776 ± 1.93515	16.32873 ± 1.37855	16.70895 ± 2.93053	1.13333 ± 0.05139
Molformer	0.83865 ± 0.03478	14.22016 ± 2.93274	16.43912 ± 1.58177	16.65601 ± 2.43440	0.86865 ± 0.02712
GROVER-Base	1.19377 ± 0.08717	14.50199 ± 3.12085	16.01343 ± 1.40306	15.48293 ± 2.53891	0.95262 ± 0.05345
GROVER-Large	1.40678 ± 0.71251	14.58141 ± 2.79295	16.59023 ± 2.07677	15.83687 ± 2.56484	0.80127 ± 0.02760
MoleBERT	0.92653 ± 0.04388	13.80503 ± 2.27607	16.49574 ± 0.76193	16.14848 ± 2.69709	0.99327 ± 0.05051
UniMol-G	0.98481 ± 0.11967	14.36104 ± 2.42867	16.17966 ± 1.10299	16.35234 ± 3.18591	1.12382 ± 0.04705
UniMol-GA	0.94057 ± 0.06946	14.25567 ± 2.53449	15.97846 ± 1.15563	16.19921 ± 3.05365	1.01884 ± 0.05959
3D-EMGP	1.37647 ± 0.08174	14.23313 ± 2.43253	16.73389 ± 1.62128	16.86962 ± 3.10379	1.35896 ± 0.03440
GraphCL	1.01371 ± 0.06520	15.22490 ± 2.75609	16.85431 ± 1.11047	16.76882 ± 2.83045	1.05820 ± 0.04692
AttrMask	1.16254 ± 0.09881	14.33355 ± 2.38728	16.28519 ± 1.02284	16.64471 ± 3.06525	1.28210 ± 0.05903
EdgePred	1.07597 ± 0.05897	14.42734 ± 2.40656	16.23059 ± 1.09242	16.92126 ± 2.55709	1.25486 ± 0.06415
3D-InfoMax	0.95121 ± 0.12371	14.41532 ± 2.39521	16.19805 ± 1.13500	15.92224 ± 3.14093	0.97784 ± 0.05533
GraphMVP	0.96266 ± 0.08580	14.54744 ± 2.76917	16.65955 ± 1.19727	15.82143 ± 2.23476	0.98693 ± 0.05838
MolCLR-GIN	1.31499 ± 0.11811	14.27227 ± 2.45869	16.78834 ± 1.57825	16.89185 ± 3.11141	1.30042 ± 0.05795
MolCLR-GCN	1.26764 ± 0.12216	14.25190 ± 2.49647	16.38077 ± 1.18079	16.87882 ± 3.11776	1.30806 ± 0.05690
GCC	1.16135 ± 0.13208	15.01874 ± 2.77289	16.58461 ± 1.09760	16.66746 ± 2.96159	1.25228 ± 0.06219
GPT-GNN	1.07726 ± 0.09286	14.06433 ± 2.20562	16.47101 ± 1.20045	16.63800 ± 2.79274	1.29428 ± 0.03562
InfoGraph	1.17776 ± 0.11841	14.48885 ± 2.17678	16.72613 ± 1.29798	16.68015 ± 2.89200	1.26522 ± 0.04035
JOAO	1.11307 ± 0.06506	14.20463 ± 2.31880	16.75709 ± 1.60248	16.60634 ± 3.00653	1.33829 ± 0.04989
JOAOv2	1.03960 ± 0.08815	13.92172 ± 2.29257	16.49752 ± 1.27982	16.39207 ± 2.90956	1.26579 ± 0.04655
PosPred	1.18144 ± 0.19714	14.29238 ± 2.49850	16.82893 ± 1.68439	16.76764 ± 3.07537	1.35191 ± 0.05944
TransM	0.85205 ± 0.02900	14.15299 ± 2.17877	15.24504 ± 0.72072	15.75456 ± 2.55389	1.02103 ± 0.04470
MPG	0.88410 ± 0.08777	14.31766 ± 2.33380	15.39469 ± 1.10505	15.88426 ± 3.33296	0.93512 ± 0.04942
KANO	1.05719 ± 0.07707	14.23719 ± 2.51959	16.02461 ± 1.34385	16.55026 ± 2.86485	1.19965 ± 0.04550

Prediction is evaluated by RMSE. For each method, the mean and standard deviation are reported for the evaluation metrics. BACEreg stands for the BACE dataset with all categorical labels restored to their original numerical values; it is only reported for analysis but excluded from final summary.

Supplementary Table 13. Molecular property regression task (scaffold split) – Part IV

Method	MLMC	HLMC	PPB	RLMC	Water Solubility
qcGEM	0.79385 ± 0.07050	0.56288 ± 0.02761	0.18062 ± 0.02218	0.66889 ± 0.04957	0.87887 ± 0.06600
ECFP	0.89859 ± 0.11287	0.59334 ± 0.03039	0.20612 ± 0.02046	0.86545 ± 0.06883	1.46256 ± 0.13408
FCFP	0.81096 ± 0.07977	0.58461 ± 0.04509	0.21478 ± 0.01914	0.79909 ± 0.01819	1.39479 ± 0.12399
AvalonFP	0.83578 ± 0.11744	0.58984 ± 0.03768	0.17982 ± 0.01881	0.72004 ± 0.03902	1.36660 ± 0.25707
TTGEN	0.86669 ± 0.11045	0.58850 ± 0.06081	0.20385 ± 0.03211	0.75774 ± 0.11032	1.38547 ± 0.14196
APGEN	0.87330 ± 0.13489	0.70001 ± 0.02904	0.21492 ± 0.02946	0.92868 ± 0.08820	1.29125 ± 0.14818
RDKFP	0.79685 ± 0.09097	0.58601 ± 0.05138	0.18855 ± 0.01819	0.70310 ± 0.06385	1.50042 ± 0.21229
MACCS	0.88435 ± 0.08422	0.67800 ± 0.03653	0.23382 ± 0.03981	0.80460 ± 0.05499	1.58106 ± 0.52573
LayeredFP	0.83834 ± 0.12034	0.67054 ± 0.06976	0.21576 ± 0.04186	0.88011 ± 0.05869	1.44559 ± 0.14498
PatternFP	0.94026 ± 0.12788	0.66661 ± 0.03392	0.21868 ± 0.04342	0.86972 ± 0.11204	1.30549 ± 0.10944
Molformer	0.79230 ± 0.09995	0.57993 ± 0.04021	0.17265 ± 0.01335	0.82343 ± 0.08180	1.00503 ± 0.11143
GROVER-Base	0.85423 ± 0.10454	0.62657 ± 0.04804	0.18186 ± 0.02758	0.82744 ± 0.11698	1.36313 ± 0.14250
GROVER-Large	0.82613 ± 0.09148	0.60478 ± 0.03239	0.19758 ± 0.03099	0.84694 ± 0.08486	1.03157 ± 0.17842
MoleBERT	0.88630 ± 0.12269	0.60976 ± 0.02806	0.21639 ± 0.02980	0.80409 ± 0.08085	1.34689 ± 0.05950
UniMol-G	0.92536 ± 0.13933	0.69343 ± 0.04428	0.23117 ± 0.05256	0.89165 ± 0.07273	1.11694 ± 0.07799
UniMol-GA	0.89652 ± 0.11113	0.68875 ± 0.04601	0.22421 ± 0.05341	0.88272 ± 0.08031	1.04559 ± 0.06087
3D-EMGP	1.01065 ± 0.12853	0.71800 ± 0.05374	0.25479 ± 0.06556	0.91573 ± 0.05026	2.01158 ± 0.23184
GraphCL	0.88481 ± 0.12411	0.60774 ± 0.02672	0.20937 ± 0.03236	0.81412 ± 0.07992	1.50097 ± 0.16649
AttrMask	0.99578 ± 0.12813	0.71910 ± 0.06993	0.24309 ± 0.05695	0.90046 ± 0.05727	1.72805 ± 0.11521
EdgePred	0.97173 ± 0.13099	0.65824 ± 0.05043	0.23808 ± 0.04835	0.83790 ± 0.05411	2.08547 ± 0.70618
3D-InfoMax	0.91081 ± 0.12124	0.64598 ± 0.03904	0.22004 ± 0.05307	0.91563 ± 0.08740	1.08551 ± 0.07227
GraphMVP	1.00037 ± 0.10052	0.68581 ± 0.03497	0.22518 ± 0.05507	0.90694 ± 0.10613	1.14628 ± 0.06986
MolCLR-GIN	1.00870 ± 0.13119	0.72887 ± 0.06160	0.24845 ± 0.05980	0.91283 ± 0.06045	1.59395 ± 0.14739
MolCLR-GCN	1.01286 ± 0.12667	0.73160 ± 0.05980	0.24346 ± 0.05596	0.92168 ± 0.05311	1.57183 ± 0.17829
GCC	0.98561 ± 0.10990	0.72650 ± 0.06266	0.23013 ± 0.04927	0.92769 ± 0.03666	1.46827 ± 0.09057
GPT-GNN	0.97651 ± 0.12348	0.68533 ± 0.04866	0.24335 ± 0.05624	0.93505 ± 0.06692	1.63867 ± 0.25180
InfoGraph	1.03008 ± 0.12528	0.71361 ± 0.05580	0.24510 ± 0.05590	0.91152 ± 0.09142	1.52563 ± 0.21329
JOAO	1.01352 ± 0.11827	0.70899 ± 0.05798	0.24642 ± 0.05658	0.91029 ± 0.06876	1.87549 ± 0.23495
JOAOv2	0.96879 ± 0.14553	0.70760 ± 0.04742	0.23395 ± 0.05130	0.90060 ± 0.03041	1.49950 ± 0.16940
PosPred	1.07864 ± 0.21598	0.73008 ± 0.06008	0.24685 ± 0.05898	0.91267 ± 0.04933	2.00038 ± 0.23081
TransM	0.83627 ± 0.08629	0.57262 ± 0.04071	0.18770 ± 0.02247	0.80528 ± 0.03255	1.22759 ± 0.17774
MPG	0.77303 ± 0.10590	0.56281 ± 0.05203	0.20561 ± 0.04549	0.71961 ± 0.08563	1.10324 ± 0.07913
KANO	0.98228 ± 0.10589	0.72146 ± 0.05961	0.23929 ± 0.05828	0.89006 ± 0.05169	1.26692 ± 0.11949

Prediction is evaluated by RMSE. For each method, the mean and standard deviation are reported for the evaluation metrics.

Supplementary Table 14. Molecular property classification task (scaffold split) – Part I

Method	BBBP	BACE	Bioavailability	HIA	PAMPA
qcGEM	0.88797 ± 0.03213	0.82897 ± 0.04812	0.60690 ± 0.08815	0.96273 ± 0.04819	0.62418 ± 0.03628
ECFP	0.74191 ± 0.09176	0.82891 ± 0.04346	0.66352 ± 0.07510	0.92256 ± 0.03717	0.61287 ± 0.06790
FCFP	0.72945 ± 0.06865	0.84900 ± 0.04437	0.57114 ± 0.11002	0.83743 ± 0.11245	0.62276 ± 0.03712
AvalonFP	0.82441 ± 0.06154	0.82441 ± 0.05129	0.56550 ± 0.05678	0.89898 ± 0.06780	0.58291 ± 0.01335
TTGEN	0.73312 ± 0.07375	0.80652 ± 0.04990	0.57889 ± 0.11409	0.88474 ± 0.06855	0.63547 ± 0.00559
APGEN	0.80766 ± 0.04311	0.84516 ± 0.03961	0.59924 ± 0.14453	0.87945 ± 0.06494	0.63166 ± 0.07503
RDKFP	0.81478 ± 0.05773	0.81956 ± 0.04949	0.55845 ± 0.03922	0.85954 ± 0.08578	0.60912 ± 0.03009
MACCS	0.82598 ± 0.07293	0.82710 ± 0.03367	0.57394 ± 0.07819	0.92571 ± 0.11738	0.64346 ± 0.03027
LayeredFP	0.83433 ± 0.04538	0.78281 ± 0.04968	0.55148 ± 0.08988	0.92313 ± 0.02584	0.57680 ± 0.04246
PatternFP	0.77649 ± 0.03834	0.81541 ± 0.03633	0.53076 ± 0.12688	0.89380 ± 0.03456	0.61630 ± 0.06132
Molformer	0.84943 ± 0.02962	0.81439 ± 0.04583	0.71499 ± 0.07651	0.96504 ± 0.03317	0.66692 ± 0.04788
GROVER-Base	0.86314 ± 0.06612	0.85026 ± 0.02596	0.64629 ± 0.09951	0.93181 ± 0.11720	0.67266 ± 0.04658
GROVER-Large	0.83025 ± 0.06314	0.83224 ± 0.03201	0.63800 ± 0.09865	0.93862 ± 0.10496	0.66654 ± 0.05078
MoleBERT	0.82606 ± 0.06401	0.81044 ± 0.05718	0.62591 ± 0.04466	0.86756 ± 0.05241	0.67347 ± 0.04477
UniMol-G	0.81723 ± 0.05816	0.79549 ± 0.04996	0.65719 ± 0.09561	0.89893 ± 0.09941	0.63799 ± 0.05559
UniMol-GA	0.83366 ± 0.05990	0.80894 ± 0.04456	0.60855 ± 0.09943	0.91820 ± 0.07629	0.67223 ± 0.04715
3D-EMGP	0.65383 ± 0.04932	0.61361 ± 0.05359	0.55474 ± 0.02643	0.74715 ± 0.10155	0.52293 ± 0.05286
GraphCL	0.76794 ± 0.06258	0.80663 ± 0.03038	0.57588 ± 0.08861	0.87376 ± 0.04769	0.63952 ± 0.05301
AttrMask	0.78565 ± 0.04846	0.77370 ± 0.03481	0.57673 ± 0.07581	0.87666 ± 0.06411	0.60333 ± 0.04452
EdgePred	0.77052 ± 0.04762	0.79134 ± 0.03830	0.52771 ± 0.10443	0.80307 ± 0.10035	0.58889 ± 0.06919
3D-InfoMax	0.82499 ± 0.03145	0.80423 ± 0.06403	0.61582 ± 0.05578	0.97136 ± 0.01636	0.66424 ± 0.03440
GraphMVP	0.85109 ± 0.06095	0.82837 ± 0.05707	0.60709 ± 0.14884	0.83859 ± 0.08687	0.63614 ± 0.03482
MolCLR-GIN	0.78907 ± 0.03093	0.74444 ± 0.05160	0.62049 ± 0.09191	0.82890 ± 0.11728	0.64408 ± 0.06818
MolCLR-GCN	0.76892 ± 0.07394	0.66145 ± 0.01589	0.57537 ± 0.12133	0.81104 ± 0.13331	0.61481 ± 0.05043
GCC	0.77096 ± 0.03805	0.69364 ± 0.09042	0.55672 ± 0.06094	0.85504 ± 0.04170	0.59982 ± 0.02506
GPT-GNN	0.77562 ± 0.06261	0.69168 ± 0.05263	0.59499 ± 0.06396	0.80902 ± 0.09864	0.55200 ± 0.05137
InfoGraph	0.67235 ± 0.04208	0.65124 ± 0.03019	0.58612 ± 0.11222	0.68137 ± 0.12859	0.52430 ± 0.07886
JOAO	0.76361 ± 0.05730	0.67366 ± 0.03466	0.58968 ± 0.04058	0.70911 ± 0.11508	0.54662 ± 0.12497
JOAOv2	0.77296 ± 0.06301	0.70677 ± 0.03725	0.53494 ± 0.10656	0.79690 ± 0.10671	0.56027 ± 0.09179
PosPred	0.73879 ± 0.05948	0.73030 ± 0.03576	0.57978 ± 0.02365	0.82347 ± 0.09168	0.60344 ± 0.10727
TransM	0.84880 ± 0.05527	0.81461 ± 0.04333	0.59226 ± 0.07759	0.83642 ± 0.12800	0.58986 ± 0.05158
MPG	0.89422 ± 0.05054	0.82894 ± 0.05817	0.63462 ± 0.06836	0.96633 ± 0.02951	0.68509 ± 0.04133
KANO	0.86689 ± 0.03215	0.75946 ± 0.03417	0.61716 ± 0.06465	0.82595 ± 0.10927	0.61286 ± 0.04029

Prediction is evaluated by ROC-AUC. For each method, the mean and standard deviation are reported for the evaluation metrics.

Supplementary Table 15. Molecular property classification task (scaffold split) – Part II

Method	Pgp	BBB	CYP1A2 Veith	CYP2C19 Veith	CYP2C9 Substrate
qcGEM	0.92759 ± 0.02316	0.85057 ± 0.03163	0.90959 ± 0.00286	0.88140 ± 0.01004	0.66033 ± 0.04257
ECFP	0.89251 ± 0.02750	0.76757 ± 0.05367	0.86861 ± 0.00668	0.82606 ± 0.00792	0.58222 ± 0.08871
FCFP	0.90254 ± 0.01770	0.78705 ± 0.02423	0.87533 ± 0.00802	0.83822 ± 0.00732	0.54211 ± 0.05946
AvalonFP	0.87805 ± 0.04459	0.84961 ± 0.03205	0.88148 ± 0.01227	0.84460 ± 0.01358	0.51436 ± 0.06149
TTGEN	0.88694 ± 0.03945	0.72880 ± 0.07359	0.88162 ± 0.00868	0.81851 ± 0.02381	0.58607 ± 0.12364
APGEN	0.88856 ± 0.02658	0.80253 ± 0.03971	0.88390 ± 0.00979	0.81412 ± 0.00681	0.52787 ± 0.08497
RDKFP	0.83172 ± 0.08347	0.82691 ± 0.03845	0.88424 ± 0.00390	0.82952 ± 0.01789	0.59360 ± 0.09207
MACCS	0.85366 ± 0.08365	0.81794 ± 0.02862	0.88721 ± 0.00927	0.85065 ± 0.01324	0.55956 ± 0.12046
LayeredFP	0.88035 ± 0.01896	0.77324 ± 0.06767	0.85861 ± 0.01244	0.79137 ± 0.02629	0.59571 ± 0.06656
PatternFP	0.87325 ± 0.05426	0.82070 ± 0.04156	0.88878 ± 0.00717	0.82804 ± 0.00997	0.57129 ± 0.08464
Molformer	0.90752 ± 0.01844	0.85861 ± 0.02919	0.90923 ± 0.00538	0.87204 ± 0.01140	0.59545 ± 0.03538
GROVER-Base	0.91796 ± 0.02331	0.83492 ± 0.04430	0.90939 ± 0.00627	0.87461 ± 0.01302	0.62794 ± 0.06158
GROVER-Large	0.91190 ± 0.01371	0.85541 ± 0.01973	0.91004 ± 0.00701	0.87457 ± 0.01343	0.66318 ± 0.02904
MoleBERT	0.89063 ± 0.03055	0.79273 ± 0.04029	0.95053 ± 0.00399	0.93226 ± 0.00477	0.64383 ± 0.06400
UniMol-G	0.89455 ± 0.02235	0.79563 ± 0.05178	0.89326 ± 0.00885	0.84372 ± 0.01086	0.51212 ± 0.07734
UniMol-GA	0.89522 ± 0.02747	0.80029 ± 0.05100	0.90186 ± 0.00635	0.85789 ± 0.01152	0.54336 ± 0.10471
3D-EMGP	0.81754 ± 0.02479	0.63653 ± 0.09101	0.66610 ± 0.02228	0.50514 ± 0.02519	0.53514 ± 0.08812
GraphCL	0.84372 ± 0.03679	0.77261 ± 0.03373	0.84620 ± 0.01060	0.83507 ± 0.01150	0.56984 ± 0.09795
AttrMask	0.79295 ± 0.04389	0.73631 ± 0.05298	0.83606 ± 0.01103	0.78110 ± 0.01435	0.55336 ± 0.08229
EdgePred	0.83410 ± 0.08013	0.71456 ± 0.05768	0.83397 ± 0.01370	0.79210 ± 0.01353	0.51386 ± 0.07280
3D-InfoMax	0.89181 ± 0.03777	0.77520 ± 0.06730	0.89655 ± 0.00973	0.86739 ± 0.01060	0.59753 ± 0.05585
GraphMVP	0.86811 ± 0.03752	0.80947 ± 0.02670	0.89733 ± 0.01009	0.86820 ± 0.01478	0.65531 ± 0.04199
MolCLR-GIN	0.81402 ± 0.04259	0.75775 ± 0.04757	0.86265 ± 0.01010	0.82767 ± 0.01743	0.60732 ± 0.07948
MolCLR-GCN	0.83795 ± 0.04910	0.76439 ± 0.01884	0.85450 ± 0.00782	0.81538 ± 0.02163	0.60799 ± 0.06881
GCC	0.88986 ± 0.02391	0.75641 ± 0.05232	0.85650 ± 0.00838	0.79980 ± 0.01810	0.55541 ± 0.06548
GPT-GNN	0.87932 ± 0.01880	0.77682 ± 0.02338	0.85299 ± 0.01915	0.78168 ± 0.01914	0.51860 ± 0.10284
InfoGraph	0.88244 ± 0.01751	0.68800 ± 0.05401	0.80805 ± 0.01373	0.71848 ± 0.02746	0.53295 ± 0.06204
JOAO	0.85782 ± 0.02332	0.74678 ± 0.06826	0.85769 ± 0.00948	0.76936 ± 0.00995	0.50688 ± 0.07282
JOAOv2	0.86430 ± 0.01879	0.77971 ± 0.06387	0.87434 ± 0.01220	0.80522 ± 0.01129	0.55487 ± 0.09470
PosPred	0.85348 ± 0.03477	0.72966 ± 0.06052	0.87785 ± 0.00774	0.79315 ± 0.01839	0.58916 ± 0.11063
TransM	0.90204 ± 0.02765	0.82356 ± 0.05837	0.89218 ± 0.00462	0.83373 ± 0.01067	0.57343 ± 0.06992
MPG	0.90609 ± 0.01905	0.84482 ± 0.04074	0.89889 ± 0.00620	0.87637 ± 0.01458	0.58268 ± 0.06218
KANO	0.89907 ± 0.01657	0.80207 ± 0.07421	0.85759 ± 0.01183	0.80274 ± 0.01858	0.58158 ± 0.06350

Prediction is evaluated by ROC-AUC. For each method, the mean and standard deviation are reported for the evaluation metrics.

Supplementary Table 16. Molecular property classification task (scaffold split) – Part III

Method	CYP2C9 Veith	CYP2D6 Substrate	CYP2D6 Veith	CYP3A4 Substrate	CYP3A4 Veith
qcGEM	0.89583 ± 0.00845	0.79886 ± 0.03798	0.85365 ± 0.00795	0.66774 ± 0.02014	0.87388 ± 0.01592
ECFP	0.85053 ± 0.01707	0.69287 ± 0.10515	0.82375 ± 0.01314	0.63751 ± 0.04671	0.85008 ± 0.01381
FCFP	0.86574 ± 0.01725	0.75052 ± 0.02783	0.81553 ± 0.02444	0.66795 ± 0.07290	0.85463 ± 0.01310
AvalonFP	0.85294 ± 0.01340	0.75533 ± 0.05970	0.80487 ± 0.02054	0.63898 ± 0.07887	0.83470 ± 0.01857
TTGEN	0.86822 ± 0.01143	0.73946 ± 0.06920	0.80183 ± 0.01307	0.64093 ± 0.07011	0.84509 ± 0.00750
APGEN	0.84172 ± 0.01974	0.73308 ± 0.07788	0.79705 ± 0.02615	0.65992 ± 0.06360	0.82667 ± 0.02225
RDKFP	0.85376 ± 0.01725	0.73801 ± 0.05915	0.81991 ± 0.00829	0.64171 ± 0.02173	0.83586 ± 0.01096
MACCS	0.86419 ± 0.01200	0.75270 ± 0.03452	0.82529 ± 0.01466	0.60377 ± 0.04584	0.83453 ± 0.01304
LayeredFP	0.82257 ± 0.01754	0.68835 ± 0.05999	0.77975 ± 0.02371	0.59191 ± 0.03112	0.82111 ± 0.01440
PatternFP	0.85638 ± 0.01865	0.68916 ± 0.05810	0.80202 ± 0.01406	0.63342 ± 0.02452	0.84239 ± 0.00765
Molformer	0.88802 ± 0.01033	0.76850 ± 0.06497	0.85171 ± 0.00478	0.65568 ± 0.07185	0.87194 ± 0.01207
GROVER-Base	0.88778 ± 0.00786	0.73491 ± 0.07212	0.84864 ± 0.00693	0.61341 ± 0.04403	0.87036 ± 0.00942
GROVER-Large	0.88865 ± 0.01425	0.74479 ± 0.06931	0.85310 ± 0.01405	0.63616 ± 0.08518	0.87275 ± 0.01224
MoleBERT	0.94193 ± 0.00446	0.71541 ± 0.05978	0.92215 ± 0.01369	0.65854 ± 0.03643	0.92933 ± 0.00515
UniMol-G	0.86341 ± 0.01448	0.72005 ± 0.04484	0.81264 ± 0.01388	0.63531 ± 0.05324	0.84330 ± 0.01272
UniMol-GA	0.87785 ± 0.01454	0.74969 ± 0.06159	0.83595 ± 0.01648	0.63514 ± 0.04516	0.86130 ± 0.00945
3D-EMGP	0.63277 ± 0.03221	0.47598 ± 0.05048	0.53433 ± 0.02163	0.51511 ± 0.08732	0.68239 ± 0.02064
GraphCL	0.85055 ± 0.01401	0.63732 ± 0.08470	0.80990 ± 0.01126	0.58385 ± 0.02624	0.82782 ± 0.01086
AttrMask	0.81535 ± 0.01760	0.73438 ± 0.06225	0.73812 ± 0.01067	0.58385 ± 0.02378	0.74508 ± 0.00951
EdgePred	0.81953 ± 0.01641	0.63199 ± 0.06137	0.77421 ± 0.00824	0.56181 ± 0.06201	0.79813 ± 0.00882
3D-InfoMax	0.87432 ± 0.01328	0.75290 ± 0.05393	0.83842 ± 0.01002	0.63269 ± 0.06375	0.86838 ± 0.01174
GraphMVP	0.88240 ± 0.01197	0.75216 ± 0.04102	0.83839 ± 0.00960	0.63780 ± 0.06982	0.86339 ± 0.01211
MolCLR-GIN	0.85007 ± 0.02097	0.73448 ± 0.03941	0.79174 ± 0.01058	0.60630 ± 0.03616	0.80263 ± 0.01106
MolCLR-GCN	0.84398 ± 0.01740	0.67650 ± 0.08523	0.78053 ± 0.01520	0.57887 ± 0.06014	0.78101 ± 0.00707
GCC	0.83087 ± 0.01723	0.72970 ± 0.09057	0.76826 ± 0.01231	0.60935 ± 0.05028	0.79930 ± 0.01989
GPT-GNN	0.83036 ± 0.01794	0.61990 ± 0.03260	0.76781 ± 0.01678	0.64056 ± 0.03927	0.78206 ± 0.02167
InfoGraph	0.74043 ± 0.02256	0.65648 ± 0.04722	0.70753 ± 0.01420	0.62774 ± 0.01976	0.73829 ± 0.02111
JOAO	0.81353 ± 0.01806	0.64392 ± 0.13095	0.73059 ± 0.01026	0.63855 ± 0.03111	0.77457 ± 0.01532
JOAOv2	0.83980 ± 0.01520	0.70002 ± 0.03141	0.76341 ± 0.01307	0.64279 ± 0.05603	0.80977 ± 0.01274
PosPred	0.83493 ± 0.01825	0.67720 ± 0.05887	0.76356 ± 0.01279	0.63608 ± 0.01616	0.80037 ± 0.01787
TransM	0.87235 ± 0.00682	0.75371 ± 0.08270	0.82241 ± 0.01656	0.68416 ± 0.07017	0.83857 ± 0.01238
MPG	0.88601 ± 0.00755	0.74549 ± 0.05816	0.84733 ± 0.00732	0.65357 ± 0.06033	0.87040 ± 0.00923
KANO	0.85148 ± 0.01531	0.74787 ± 0.07223	0.79029 ± 0.01082	0.62300 ± 0.02610	0.80444 ± 0.01120

Prediction is evaluated by ROC-AUC. For each method, the mean and standard deviation are reported for the evaluation metrics.

Supplementary Table 17. Molecular property classification task (scaffold split) – Part IV

Method	Ames	Carcinogens	DILI	hERG Blockers	SkinReac
qcGEM	0.86069 ± 0.01285	0.82976 ± 0.12825	0.83007 ± 0.09565	0.83286 ± 0.05640	0.67427 ± 0.10432
ECFP	0.79260 ± 0.03559	0.74105 ± 0.11801	0.81769 ± 0.09804	0.81329 ± 0.03514	0.50480 ± 0.03563
FCFP	0.81113 ± 0.02093	0.74057 ± 0.19200	0.79377 ± 0.08718	0.79233 ± 0.05155	0.58705 ± 0.09123
AvalonFP	0.82494 ± 0.01741	0.75552 ± 0.17210	0.79648 ± 0.06769	0.76050 ± 0.04851	0.63176 ± 0.03049
TTGEN	0.78850 ± 0.01068	0.70119 ± 0.11174	0.83172 ± 0.03962	0.77264 ± 0.05115	0.63935 ± 0.06975
APGEN	0.79811 ± 0.01587	0.79090 ± 0.11641	0.77390 ± 0.09322	0.79002 ± 0.03896	0.64449 ± 0.05576
RDKFP	0.80588 ± 0.01838	0.78679 ± 0.15615	0.79115 ± 0.05905	0.72976 ± 0.06940	0.61687 ± 0.05580
MACCS	0.83346 ± 0.02491	0.77638 ± 0.14017	0.84043 ± 0.10607	0.82661 ± 0.03360	0.65974 ± 0.03021
LayeredFP	0.81694 ± 0.01925	0.72148 ± 0.24300	0.82291 ± 0.08214	0.70854 ± 0.08305	0.70190 ± 0.07341
PatternFP	0.83669 ± 0.01995	0.80286 ± 0.16227	0.78856 ± 0.09847	0.79427 ± 0.05192	0.70048 ± 0.03369
Molformer	0.83857 ± 0.01346	0.76862 ± 0.09890	0.80130 ± 0.05559	0.86756 ± 0.04562	0.76498 ± 0.05649
GROVER-Base	0.85786 ± 0.02629	0.80238 ± 0.16411	0.82485 ± 0.07102	0.85128 ± 0.02389	0.62729 ± 0.06413
GROVER-Large	0.85554 ± 0.02038	0.62086 ± 0.15287	0.81276 ± 0.07519	0.85783 ± 0.03515	0.58322 ± 0.07434
MoleBERT	0.82518 ± 0.01610	0.77614 ± 0.05121	0.81051 ± 0.06640	0.82828 ± 0.06036	0.66951 ± 0.05728
UniMol-G	0.81468 ± 0.02351	0.72619 ± 0.18685	0.83089 ± 0.09044	0.82680 ± 0.05551	0.71731 ± 0.07446
UniMol-GA	0.82479 ± 0.03076	0.68971 ± 0.18703	0.85025 ± 0.08696	0.84754 ± 0.04933	0.72923 ± 0.07385
3D-EMGP	0.56512 ± 0.02311	0.50224 ± 0.23743	0.44392 ± 0.07304	0.63109 ± 0.11817	0.73581 ± 0.03757
GraphCL	0.77868 ± 0.02433	0.74067 ± 0.10027	0.79741 ± 0.07662	0.75697 ± 0.08453	0.69558 ± 0.08320
AttrMask	0.72658 ± 0.02075	0.69429 ± 0.14329	0.78531 ± 0.07184	0.77129 ± 0.04404	0.43482 ± 0.04752
EdgePred	0.67203 ± 0.02880	0.64790 ± 0.23063	0.72747 ± 0.05410	0.65802 ± 0.06557	0.61357 ± 0.10344
3D-InfoMax	0.83300 ± 0.01685	0.73619 ± 0.22387	0.79495 ± 0.07501	0.81123 ± 0.03317	0.60886 ± 0.11537
GraphMVP	0.83040 ± 0.02360	0.75071 ± 0.18293	0.85684 ± 0.07587	0.82463 ± 0.02913	0.56355 ± 0.06820
MolCLR-GIN	0.77888 ± 0.02566	0.54886 ± 0.24927	0.82497 ± 0.08356	0.76300 ± 0.08156	0.62052 ± 0.07654
MolCLR-GCN	0.79625 ± 0.02826	0.78414 ± 0.10567	0.81228 ± 0.09761	0.75681 ± 0.08813	0.54219 ± 0.08852
GCC	0.75962 ± 0.01862	0.71852 ± 0.14455	0.78965 ± 0.05059	0.75180 ± 0.03454	0.67602 ± 0.05199
GPT-GNN	0.71630 ± 0.01871	0.49805 ± 0.16916	0.66950 ± 0.07894	0.76778 ± 0.05646	0.69751 ± 0.10306
InfoGraph	0.66035 ± 0.02421	0.55795 ± 0.05821	0.65341 ± 0.04909	0.74399 ± 0.05889	0.57809 ± 0.11113
JOAO	0.74587 ± 0.02992	0.69248 ± 0.14032	0.77546 ± 0.08176	0.74846 ± 0.08570	0.68598 ± 0.03351
JOAOv2	0.74033 ± 0.04022	0.70557 ± 0.07727	0.82583 ± 0.07655	0.75780 ± 0.06903	0.68068 ± 0.04352
PosPred	0.75474 ± 0.02882	0.62714 ± 0.12057	0.77978 ± 0.10555	0.77200 ± 0.07563	0.73269 ± 0.07483
TransM	0.82353 ± 0.01158	0.77148 ± 0.15387	0.82965 ± 0.05957	0.74152 ± 0.07619	0.66892 ± 0.09595
MPG	0.82445 ± 0.02760	0.84167 ± 0.10515	0.82643 ± 0.10095	0.78287 ± 0.02760	0.64641 ± 0.05218
KANO	0.76621 ± 0.02851	0.79738 ± 0.16226	0.84482 ± 0.04752	0.80449 ± 0.04226	0.62886 ± 0.07813

Prediction is evaluated by ROC-AUC. For each method, the mean and standard deviation are reported for the evaluation metrics.

Supplementary Table 18. Molecular property classification task (scaffold split) – Part V

Method	AMES(PharmaBench)	BBB(PharmaBench)
qcGEM	0.87140 ± 0.01376	0.89536 ± 0.03248
ECFP	0.83676 ± 0.01714	0.84394 ± 0.05320
FCFP	0.82105 ± 0.02410	0.87085 ± 0.02873
AvalonFP	0.85096 ± 0.00810	0.88725 ± 0.03275
TTGEN	0.81888 ± 0.02392	0.85748 ± 0.04777
APGEN	0.81275 ± 0.00812	0.85664 ± 0.04965
RDKFP	0.85603 ± 0.01613	0.87472 ± 0.03582
MACCS	0.85211 ± 0.00948	0.88147 ± 0.03750
LayeredFP	0.82138 ± 0.01515	0.83811 ± 0.06593
PatternFP	0.85014 ± 0.00173	0.86906 ± 0.04097
Molformer	0.84175 ± 0.01081	0.89348 ± 0.02256
GROVER-Base	0.87559 ± 0.01557	0.89556 ± 0.03323
GROVER-Large	0.87057 ± 0.00878	0.89766 ± 0.03948
MoleBERT	0.83349 ± 0.00853	0.85718 ± 0.04777
UniMol-G	0.84370 ± 0.01210	0.86073 ± 0.03984
UniMol-GA	0.85457 ± 0.01189	0.86740 ± 0.03830
3D-EMGP	0.63168 ± 0.02165	0.63191 ± 0.02974
GraphCL	0.79406 ± 0.00712	0.84796 ± 0.03485
AttrMask	0.75731 ± 0.01960	0.80938 ± 0.03172
EdgePred	0.72675 ± 0.03292	0.80938 ± 0.06652
3D-InfoMax	0.85350 ± 0.01384	0.86484 ± 0.04962
GraphMVP	0.84287 ± 0.01427	0.86697 ± 0.04676
MolCLR-GIN	0.80996 ± 0.01969	0.84792 ± 0.02573
MolCLR-GCN	0.80292 ± 0.02414	0.84055 ± 0.03645
GCC	0.77015 ± 0.02252	0.83200 ± 0.04133
GPT-GNN	0.73190 ± 0.02405	0.79186 ± 0.05304
InfoGraph	0.70143 ± 0.03158	0.79417 ± 0.04824
JOAO	0.65300 ± 0.04777	0.67479 ± 0.05268
JOAOv2	0.74871 ± 0.02792	0.79230 ± 0.04651
PosPred	0.66137 ± 0.03240	0.59414 ± 0.02879
TransM	0.83880 ± 0.00874	0.88110 ± 0.04420
MPG	0.85719 ± 0.01000	0.88196 ± 0.04560
KANO	0.78541 ± 0.01458	0.86377 ± 0.03959

Prediction is evaluated by ROC-AUC. For each method, the mean and standard deviation are reported for the evaluation metrics.

Supplementary Table 19. Molecular property regression task (random split) – Part I

Method	FreeSolv	ESOL	Lipophilicity	Caco2	Solubility AqSolDB
qcGEM	0.78718 ± 0.09563	0.58089 ± 0.06436	0.62173 ± 0.03459	0.40253 ± 0.01285	0.85471 ± 0.03503
ECFP	2.36869 ± 0.35691	1.45311 ± 0.24738	0.92559 ± 0.03047	0.57906 ± 0.04601	1.32836 ± 0.02024
FCFP	1.90083 ± 0.38249	1.34008 ± 0.25042	0.86026 ± 0.01096	0.49239 ± 0.02758	1.25412 ± 0.03153
AvalonFP	1.27456 ± 0.08631	1.08382 ± 0.06474	0.91070 ± 0.01735	0.50378 ± 0.02852	1.15840 ± 0.03683
TTGEN	2.98176 ± 0.35794	1.29109 ± 0.19365	0.98088 ± 0.03489	0.57542 ± 0.03087	1.30146 ± 0.03579
APGEN	2.39174 ± 0.43178	1.07674 ± 0.12051	0.95102 ± 0.01752	0.55896 ± 0.03030	1.19167 ± 0.02729
RDKFP	1.47780 ± 0.22079	1.24394 ± 0.17633	0.91309 ± 0.02450	0.53023 ± 0.05902	1.40740 ± 0.04507
MACCS	1.04916 ± 0.12566	1.06645 ± 0.15462	0.89326 ± 0.02377	0.47378 ± 0.02430	1.20947 ± 0.03036
LayeredFP	1.49811 ± 0.16076	0.96846 ± 0.10805	1.07219 ± 0.02018	0.59094 ± 0.02824	1.21310 ± 0.03562
PatternFP	1.50138 ± 0.21262	0.85725 ± 0.08611	0.99659 ± 0.01719	0.55638 ± 0.03597	1.16370 ± 0.05192
Molformer	1.47969 ± 0.08330	0.83695 ± 0.06252	0.70899 ± 0.01873	0.43652 ± 0.03371	0.93503 ± 0.02774
GROVER-Base	0.96533 ± 0.07892	0.79499 ± 0.11912	0.72415 ± 0.04390	0.65451 ± 0.05626	1.02653 ± 0.01874
GROVER-Large	1.17186 ± 0.16833	0.71944 ± 0.12297	0.65099 ± 0.04271	0.85010 ± 0.63415	0.91848 ± 0.01683
MoleBERT	1.86938 ± 0.49738	1.03651 ± 0.05397	0.79185 ± 0.02047	0.57447 ± 0.05401	1.20439 ± 0.03851
UniMol-G	1.64737 ± 0.13133	0.84864 ± 0.11981	0.99857 ± 0.01292	0.54870 ± 0.06500	1.02952 ± 0.02827
UniMol-GA	1.36139 ± 0.13317	0.74521 ± 0.09003	0.93649 ± 0.02523	0.58749 ± 0.07565	0.95160 ± 0.02775
3D-EMGP	3.51922 ± 0.55748	2.10998 ± 0.22642	1.18422 ± 0.01645	1.06128 ± 0.07722	2.27124 ± 0.08669
GraphCL	2.82872 ± 0.09572	1.20677 ± 0.17755	0.92866 ± 0.01451	0.58179 ± 0.05575	1.25706 ± 0.04751
AttrMask	2.90616 ± 0.36996	1.67838 ± 0.14574	1.10442 ± 0.01733	0.68598 ± 0.05105	1.68637 ± 0.04243
EdgePred	2.59230 ± 0.33980	1.63323 ± 0.16773	1.07283 ± 0.03111	0.65763 ± 0.03050	1.77874 ± 0.05874
3D-InfoMax	1.32417 ± 0.14903	0.86840 ± 0.09383	0.86611 ± 0.00892	0.50132 ± 0.04371	1.05595 ± 0.06003
GraphMVP	1.21258 ± 0.07180	0.85712 ± 0.12313	0.82116 ± 0.02441	0.49912 ± 0.03650	1.09651 ± 0.03553
MolCLR-GIN	1.97780 ± 0.30714	1.28791 ± 0.17466	1.11815 ± 0.02211	0.69972 ± 0.05957	1.40107 ± 0.02429
MolCLR-GCN	2.51501 ± 0.09334	1.37550 ± 0.14020	1.11865 ± 0.01494	0.69350 ± 0.04551	1.43219 ± 0.03956
GCC	1.98263 ± 0.13328	1.10148 ± 0.16717	1.05874 ± 0.01349	0.56573 ± 0.06420	1.26884 ± 0.02092
GPT-GNN	2.11439 ± 0.34910	1.07815 ± 0.17125	1.02573 ± 0.02200	0.85808 ± 0.01037	1.24042 ± 0.06641
InfoGraph	3.11819 ± 0.52885	1.41469 ± 0.12333	1.13866 ± 0.01920	0.69179 ± 0.05223	1.47627 ± 0.03643
JOAO	2.14021 ± 0.26118	1.15846 ± 0.12114	1.11536 ± 0.01601	0.87319 ± 0.09433	1.35219 ± 0.00576
JOAOv2	1.86046 ± 0.16855	1.03716 ± 0.13959	1.08737 ± 0.01079	0.68120 ± 0.04617	1.21096 ± 0.03785
PosPred	2.57304 ± 0.40634	1.26034 ± 0.15305	1.11610 ± 0.02406	0.92363 ± 0.06716	1.42016 ± 0.10777
TransM	1.04679 ± 0.10646	0.77901 ± 0.11125	0.89229 ± 0.01118	0.48656 ± 0.05391	1.00454 ± 0.02796
MPG	1.20850 ± 0.09798	0.77476 ± 0.09514	0.75643 ± 0.03197	0.46909 ± 0.01861	0.99578 ± 0.02862
KANO	1.98737 ± 0.28048	0.99825 ± 0.10339	1.08147 ± 0.01624	0.53161 ± 0.05411	1.24392 ± 0.02353

Prediction is evaluated by RMSE. For each method, the mean and standard deviation are reported for the evaluation metrics.

Supplementary Table 20. Molecular property regression task (random split) – Part II

Method	VDss	Clearance Hepatocyte	Clearance Microsome	Half Life	LD50
qcGEM	0.00700 ± 0.00183	0.27156 ± 0.01237	0.25278 ± 0.02112	0.06500 ± 0.06613	0.56211 ± 0.01627
ECFP	0.00723 ± 0.00205	0.29609 ± 0.01111	0.25430 ± 0.02430	0.06493 ± 0.06620	0.67167 ± 0.01577
FCFP	0.00707 ± 0.00188	0.30123 ± 0.01375	0.24843 ± 0.02739	0.06538 ± 0.06625	0.67005 ± 0.01521
AvalonFP	0.00717 ± 0.00200	0.30334 ± 0.01333	0.25799 ± 0.01535	0.06548 ± 0.06657	0.59123 ± 0.00444
TTGEN	0.00744 ± 0.00180	0.30805 ± 0.01084	0.25971 ± 0.01079	0.06550 ± 0.06638	0.72229 ± 0.02263
APGEN	0.00722 ± 0.00192	0.30505 ± 0.01283	0.26376 ± 0.01465	0.06559 ± 0.06691	0.66244 ± 0.01683
RDKFP	0.00748 ± 0.00173	0.30661 ± 0.00301	0.25536 ± 0.01314	0.06549 ± 0.06665	0.61590 ± 0.01921
MACCS	0.00718 ± 0.00197	0.30113 ± 0.01432	0.25277 ± 0.01442	0.06554 ± 0.06650	0.64974 ± 0.01336
LayeredFP	0.00771 ± 0.00195	0.30638 ± 0.02017	0.26132 ± 0.01276	0.06566 ± 0.06657	0.63494 ± 0.01683
PatternFP	0.00770 ± 0.00189	0.30310 ± 0.00532	0.26237 ± 0.01626	0.06540 ± 0.06672	0.65280 ± 0.01617
Molformer	0.00719 ± 0.00184	0.28094 ± 0.01459	0.23862 ± 0.01348	0.06546 ± 0.06653	0.61664 ± 0.00405
GROVER-Base	0.00672 ± 0.00186	0.27606 ± 0.02753	0.23300 ± 0.01944	0.06510 ± 0.06472	0.65442 ± 0.05419
GROVER-Large	0.00737 ± 0.00182	0.27903 ± 0.02402	0.23702 ± 0.01815	0.06563 ± 0.06582	0.59060 ± 0.02766
MoleBERT	0.00723 ± 0.00196	0.29203 ± 0.00913	0.26719 ± 0.02237	0.06520 ± 0.06687	0.71915 ± 0.02306
UniMol-G	0.00952 ± 0.00402	0.30639 ± 0.01448	0.25579 ± 0.01445	0.07191 ± 0.06398	0.71297 ± 0.03265
UniMol-GA	0.00947 ± 0.00402	0.30311 ± 0.01316	0.25280 ± 0.01280	0.07186 ± 0.06405	0.68163 ± 0.03458
3D-EMGP	0.00813 ± 0.00212	0.32706 ± 0.01249	0.27418 ± 0.01361	0.06553 ± 0.06678	0.92055 ± 0.02702
GraphCL	0.00747 ± 0.00204	0.29094 ± 0.01103	0.26026 ± 0.01363	0.06555 ± 0.06651	0.67950 ± 0.01528
AttrMask	0.00772 ± 0.00208	0.32507 ± 0.01080	0.27366 ± 0.01447	0.06600 ± 0.06671	0.86203 ± 0.02951
EdgePred	0.00769 ± 0.00211	0.32476 ± 0.01049	0.26532 ± 0.00989	0.06599 ± 0.06648	0.76549 ± 0.02266
3D-InfoMax	0.00739 ± 0.00174	0.30908 ± 0.01006	0.25790 ± 0.01294	0.06587 ± 0.06667	0.65818 ± 0.02434
GraphMVP	0.00739 ± 0.00187	0.30129 ± 0.02252	0.25015 ± 0.01845	0.06558 ± 0.06653	0.65539 ± 0.01530
MolCLR-GIN	0.00783 ± 0.00200	0.32532 ± 0.01011	0.27393 ± 0.01366	0.06606 ± 0.06683	0.79513 ± 0.02159
MolCLR-GCN	0.00793 ± 0.00184	0.32596 ± 0.00983	0.27220 ± 0.01484	0.06587 ± 0.06679	0.79826 ± 0.02492
GCC	0.00773 ± 0.00201	0.31469 ± 0.00906	0.27254 ± 0.01397	0.06597 ± 0.06680	0.72897 ± 0.01589
GPT-GNN	0.00876 ± 0.00193	0.31800 ± 0.00490	0.25840 ± 0.01455	0.06831 ± 0.06561	0.70142 ± 0.01558
InfoGraph	0.00772 ± 0.00188	0.32847 ± 0.00899	0.26507 ± 0.01550	0.06662 ± 0.06660	0.86617 ± 0.03219
JOAO	0.01386 ± 0.00494	0.32206 ± 0.00577	0.26673 ± 0.01204	0.12246 ± 0.09414	0.77380 ± 0.03808
JOAOv2	0.00821 ± 0.00208	0.31866 ± 0.00569	0.27060 ± 0.01560	0.06844 ± 0.06729	0.75337 ± 0.02739
PosPred	0.13485 ± 0.17820	0.31996 ± 0.00913	0.32762 ± 0.11263	0.27596 ± 0.36060	0.81195 ± 0.05407
TransM	0.00742 ± 0.00180	0.29394 ± 0.01232	0.26796 ± 0.01655	0.06557 ± 0.06633	0.63233 ± 0.00992
MPG	0.00747 ± 0.00188	0.28130 ± 0.00675	0.24349 ± 0.02253	0.06587 ± 0.06686	0.62866 ± 0.00674
KANO	0.00776 ± 0.00206	0.32603 ± 0.01127	0.27506 ± 0.01277	0.06599 ± 0.06672	0.75723 ± 0.02014

Prediction is evaluated by RMSE. For each method, the mean and standard deviation are reported for the evaluation metrics.

Supplementary Table 21. Molecular property regression task (random split) – Part III

Method	BACEreg	CYP 2C9	CYP 2D6	CYP 3A4	LogD
qcGEM	0.71257 ± 0.04452	14.83750 ± 1.43474	17.69330 ± 1.98676	14.21860 ± 0.91084	0.67071 ± 0.01403
ECFP	0.73079 ± 0.03907	14.61976 ± 1.55340	17.85011 ± 2.60875	15.99082 ± 1.09453	0.96815 ± 0.02119
FCFP	0.77732 ± 0.05071	14.42796 ± 1.74095	17.50086 ± 1.89314	16.42795 ± 1.75598	0.90269 ± 0.01900
AvalonFP	0.81680 ± 0.08039	14.68276 ± 1.63452	17.75384 ± 2.17797	16.24482 ± 1.58217	0.92074 ± 0.00741
TTGEN	0.80553 ± 0.04991	14.54331 ± 1.53171	17.86757 ± 2.62471	16.00723 ± 1.62659	1.04731 ± 0.02393
APGEN	0.78379 ± 0.07228	14.81640 ± 1.65702	17.37268 ± 1.80104	15.86630 ± 1.09243	1.04374 ± 0.02445
RDKFP	0.84288 ± 0.09136	14.16698 ± 1.69427	17.28505 ± 2.48335	15.87120 ± 1.59206	0.99321 ± 0.02212
MACCS	0.87760 ± 0.05113	15.51700 ± 1.87764	17.38545 ± 1.78493	15.63653 ± 1.48517	1.04701 ± 0.02150
LayeredFP	0.87218 ± 0.05132	15.06366 ± 1.43501	17.92813 ± 2.11743	16.18895 ± 1.13668	1.20619 ± 0.01809
PatternFP	0.80840 ± 0.06892	14.70275 ± 1.16792	17.96723 ± 1.64554	16.60171 ± 1.30429	1.12294 ± 0.02123
Molformer	0.75725 ± 0.02544	16.06817 ± 1.72088	17.33430 ± 1.92469	15.69511 ± 1.26846	0.80082 ± 0.01177
GROVER-Base	1.22887 ± 0.12925	15.31119 ± 1.61604	18.15392 ± 2.29439	14.98178 ± 1.45306	0.87907 ± 0.02132
GROVER-Large	1.37919 ± 0.73167	15.26130 ± 2.17512	18.35010 ± 2.27352	15.64180 ± 1.86439	0.74034 ± 0.01926
MoleBERT	0.85503 ± 0.02314	14.27381 ± 1.43136	17.77907 ± 1.95192	15.32739 ± 1.54730	0.99697 ± 0.02406
UniMol-G	0.94319 ± 0.07438	15.15529 ± 1.44013	17.77668 ± 2.35476	15.95409 ± 1.74707	1.13230 ± 0.02100
UniMol-GA	0.95164 ± 0.08595	15.10990 ± 1.46335	17.69371 ± 2.20867	15.44629 ± 1.62115	1.01723 ± 0.02644
3D-EMGP	1.40522 ± 0.07280	15.00173 ± 1.58618	18.63347 ± 2.44283	16.69108 ± 1.40154	1.38581 ± 0.02332
GraphCL	0.86651 ± 0.07508	15.02949 ± 0.92262	18.41746 ± 2.91818	15.30826 ± 0.81408	1.02343 ± 0.01336
AttrMask	1.12562 ± 0.04351	14.95124 ± 1.62601	18.31073 ± 2.01544	16.59988 ± 1.40897	1.31138 ± 0.02125
EdgePred	0.94037 ± 0.07278	14.56378 ± 1.36989	17.67169 ± 2.30494	16.00597 ± 1.58911	1.26118 ± 0.02245
3D-InfoMax	0.88781 ± 0.03793	14.96705 ± 1.74704	17.83941 ± 2.24205	15.45961 ± 1.45435	0.97635 ± 0.02183
GraphMVP	0.90266 ± 0.05881	14.84490 ± 1.57222	17.90984 ± 1.97455	15.45968 ± 1.14948	0.99256 ± 0.02708
MolCLR-GIN	1.30366 ± 0.09059	15.01775 ± 1.56376	18.67602 ± 2.37742	16.73035 ± 1.39745	1.34825 ± 0.03577
MolCLR-GCN	1.25245 ± 0.08377	15.02078 ± 1.57909	18.37002 ± 2.15875	16.75063 ± 1.38263	1.34402 ± 0.02381
GCC	1.08573 ± 0.04608	14.96104 ± 1.91764	18.23215 ± 2.04569	16.46209 ± 1.30227	1.26713 ± 0.01971
GPT-GNN	1.07185 ± 0.07801	14.96281 ± 1.48392	18.23398 ± 2.47632	16.58132 ± 1.30569	1.31552 ± 0.02466
InfoGraph	1.18441 ± 0.07359	15.01489 ± 1.45267	18.25152 ± 2.48212	16.91359 ± 1.19945	1.29704 ± 0.01252
JOAO	1.14185 ± 0.08752	14.84363 ± 1.52752	18.57170 ± 2.40914	16.74065 ± 1.26905	1.37313 ± 0.02427
JOAOv2	1.03725 ± 0.09822	15.17086 ± 1.35234	18.23501 ± 1.80147	16.30801 ± 1.33812	1.29446 ± 0.02711
PosPred	1.00713 ± 0.06494	15.00194 ± 1.58051	18.67077 ± 2.39546	16.67629 ± 1.35412	1.39745 ± 0.01467
TransM	0.75991 ± 0.03203	14.49028 ± 1.38306	18.02306 ± 2.05820	14.97800 ± 0.87497	0.95851 ± 0.01548
MPG	0.78889 ± 0.03126	14.87695 ± 1.66393	17.87871 ± 2.56221	14.98437 ± 1.14710	0.90962 ± 0.02155
KANO	0.99304 ± 0.06070	15.05822 ± 1.62667	17.64842 ± 2.11348	16.24698 ± 1.58406	1.22634 ± 0.01767

Prediction is evaluated by RMSE. For each method, the mean and standard deviation are reported for the evaluation metrics. BACEreg stands for the BACE dataset with all categorical labels restored to their original numerical values; it is only reported for analysis but excluded from final summary.

Supplementary Table 22. Molecular property regression task (random split) – Part IV

Method	MLMC	HLMC	PPB	RLMC	Water Solubility
qcGEM	0.63277 ± 0.06069	0.49524 ± 0.02240	0.15481 ± 0.01578	0.56906 ± 0.02014	0.78888 ± 0.02196
ECFP	0.73929 ± 0.05612	0.56678 ± 0.02187	0.18604 ± 0.01438	0.73657 ± 0.06412	1.25920 ± 0.01985
FCFP	0.73309 ± 0.05530	0.53393 ± 0.04239	0.18643 ± 0.01462	0.70565 ± 0.02762	1.22403 ± 0.02715
AvalonFP	0.70840 ± 0.04904	0.52898 ± 0.02765	0.16810 ± 0.01374	0.63289 ± 0.02972	1.14042 ± 0.02960
TTGEN	0.76727 ± 0.04239	0.53775 ± 0.03143	0.18546 ± 0.01051	0.67489 ± 0.02941	1.17395 ± 0.02776
APGEN	0.84471 ± 0.05565	0.65930 ± 0.03051	0.18672 ± 0.01384	0.76780 ± 0.04080	1.11549 ± 0.02474
RDKFP	0.69665 ± 0.04293	0.52952 ± 0.03246	0.18228 ± 0.01373	0.65569 ± 0.03147	1.31943 ± 0.02643
MACCS	0.84033 ± 0.07321	0.67208 ± 0.03388	0.22543 ± 0.02650	0.78858 ± 0.06063	1.24698 ± 0.03671
LayeredFP	0.79618 ± 0.02014	0.69666 ± 0.04391	0.21652 ± 0.02052	0.81021 ± 0.04379	1.28305 ± 0.02406
PatternFP	0.84650 ± 0.04521	0.63119 ± 0.04672	0.19821 ± 0.02122	0.79099 ± 0.02738	1.18206 ± 0.02434
Molformer	0.71088 ± 0.06892	0.53107 ± 0.02528	0.16776 ± 0.00956	0.67940 ± 0.06161	0.90627 ± 0.02573
GROVER-Base	0.81341 ± 0.09692	0.53214 ± 0.03444	0.17475 ± 0.00769	0.71716 ± 0.08289	1.36188 ± 0.05373
GROVER-Large	0.81141 ± 0.11296	0.61275 ± 0.06130	0.17109 ± 0.00899	0.79257 ± 0.05564	1.04408 ± 0.10563
MoleBERT	0.83595 ± 0.04644	0.59545 ± 0.02888	0.21272 ± 0.01762	0.73960 ± 0.05278	1.28030 ± 0.05046
UniMol-G	0.95366 ± 0.04640	0.75306 ± 0.05215	0.23091 ± 0.03357	0.88502 ± 0.04139	1.06987 ± 0.02712
UniMol-GA	0.91242 ± 0.04961	0.74500 ± 0.04946	0.22510 ± 0.03379	0.85519 ± 0.03544	1.02477 ± 0.05321
3D-EMGP	1.09766 ± 0.04082	0.80808 ± 0.05358	0.26888 ± 0.03856	0.97292 ± 0.05438	1.95593 ± 0.05365
GraphCL	0.77202 ± 0.04577	0.62361 ± 0.03136	0.19672 ± 0.01718	0.70777 ± 0.02966	1.29410 ± 0.04341
AttrMask	1.08593 ± 0.05073	0.79020 ± 0.04817	0.25079 ± 0.03910	0.98342 ± 0.07095	1.69788 ± 0.04653
EdgePred	0.89283 ± 0.07223	0.66685 ± 0.03350	0.23341 ± 0.03062	0.76278 ± 0.03416	1.71171 ± 0.06118
3D-InfoMax	0.83684 ± 0.08374	0.69356 ± 0.04176	0.22006 ± 0.02867	0.78050 ± 0.06845	1.04696 ± 0.02923
GraphMVP	0.93801 ± 0.05791	0.71565 ± 0.03509	0.23241 ± 0.02926	0.84994 ± 0.04789	1.12955 ± 0.02584
MolCLR-GIN	1.09740 ± 0.04146	0.80318 ± 0.05330	0.25573 ± 0.03597	0.99252 ± 0.06524	1.53070 ± 0.03310
MolCLR-GCN	1.10318 ± 0.04447	0.80413 ± 0.05415	0.25548 ± 0.03743	0.97851 ± 0.07320	1.52785 ± 0.04455
GCC	1.04019 ± 0.06286	0.78156 ± 0.04473	0.23890 ± 0.02759	0.93093 ± 0.05604	1.38370 ± 0.04528
GPT-GNN	1.03660 ± 0.05533	0.78698 ± 0.04662	0.24924 ± 0.02945	0.92406 ± 0.05475	1.53158 ± 0.06710
InfoGraph	1.10839 ± 0.03496	0.79645 ± 0.05143	0.25185 ± 0.03170	0.98700 ± 0.05138	1.47351 ± 0.05248
JOAO	1.09418 ± 0.04747	0.80435 ± 0.03991	0.25577 ± 0.03535	0.95825 ± 0.05390	1.83374 ± 0.05315
JOAOv2	0.98589 ± 0.04929	0.79236 ± 0.04202	0.24217 ± 0.03191	0.91225 ± 0.06448	1.43043 ± 0.04733
PosPred	1.09816 ± 0.04958	0.80642 ± 0.05375	0.25789 ± 0.03641	0.96812 ± 0.05194	1.96673 ± 0.05532
TransM	0.66218 ± 0.06123	0.51168 ± 0.02077	0.16620 ± 0.01875	0.60335 ± 0.04125	1.06834 ± 0.02621
MPG	0.68776 ± 0.04082	0.55202 ± 0.02416	0.19988 ± 0.02262	0.64792 ± 0.01945	1.02692 ± 0.03244
KANO	1.03743 ± 0.05210	0.79787 ± 0.05182	0.25073 ± 0.03635	0.93621 ± 0.05951	1.24239 ± 0.02949

Prediction is evaluated by RMSE. For each method, the mean and standard deviation are reported for the evaluation metrics.

Supplementary Table 23. Molecular property classification task (random split) – Part I

Method	BBBP	BACE	Bioavailability	HIA	PAMPA
qcGEM	0.91287 ± 0.00879	0.86240 ± 0.02731	0.73592 ± 0.07812	0.93782 ± 0.04531	0.71808 ± 0.05061
ECFP	0.83857 ± 0.02213	0.86541 ± 0.02735	0.66671 ± 0.07287	0.80459 ± 0.08204	0.71049 ± 0.02333
FCFP	0.86815 ± 0.02622	0.87067 ± 0.02953	0.62522 ± 0.07333	0.80686 ± 0.04488	0.69549 ± 0.03028
AvalonFP	0.87345 ± 0.02510	0.86024 ± 0.01300	0.69629 ± 0.06802	0.82153 ± 0.07131	0.70774 ± 0.02404
TTGEN	0.83974 ± 0.01933	0.85550 ± 0.02025	0.64977 ± 0.05006	0.75294 ± 0.06638	0.66696 ± 0.04262
APGEN	0.86726 ± 0.01928	0.86334 ± 0.01915	0.68493 ± 0.04035	0.84069 ± 0.08372	0.67537 ± 0.01915
RDKFP	0.87658 ± 0.03036	0.86345 ± 0.02799	0.64617 ± 0.07665	0.80599 ± 0.13707	0.67289 ± 0.05949
MACCS	0.89735 ± 0.02534	0.86041 ± 0.03742	0.73881 ± 0.04554	0.92587 ± 0.04465	0.68989 ± 0.05623
LayeredFP	0.87420 ± 0.02800	0.86070 ± 0.01545	0.66910 ± 0.08325	0.82294 ± 0.05198	0.65299 ± 0.02026
PatternFP	0.88393 ± 0.01264	0.85246 ± 0.01995	0.63833 ± 0.06395	0.73479 ± 0.16300	0.67124 ± 0.04523
Molformer	0.90906 ± 0.02264	0.86284 ± 0.02271	0.83193 ± 0.04935	0.96416 ± 0.02294	0.73774 ± 0.03103
GROVER-Base	0.89720 ± 0.03266	0.89395 ± 0.01771	0.74038 ± 0.04658	0.94164 ± 0.04762	0.73527 ± 0.03033
GROVER-Large	0.88974 ± 0.01524	0.88544 ± 0.02552	0.78234 ± 0.04836	0.92840 ± 0.07513	0.71503 ± 0.03365
MoleBERT	0.87261 ± 0.01722	0.85781 ± 0.04014	0.68396 ± 0.03871	0.91391 ± 0.05362	0.72083 ± 0.02261
UniMol-G	0.85685 ± 0.02691	0.81418 ± 0.02455	0.64713 ± 0.12414	0.94554 ± 0.02949	0.67178 ± 0.04107
UniMol-GA	0.86719 ± 0.01808	0.84035 ± 0.02355	0.66990 ± 0.07438	0.94654 ± 0.04035	0.68454 ± 0.04366
3D-EMGP	0.63414 ± 0.02869	0.60810 ± 0.04667	0.65211 ± 0.07977	0.62725 ± 0.19084	0.53058 ± 0.07156
GraphCL	0.84365 ± 0.02214	0.85667 ± 0.03435	0.66168 ± 0.09282	0.88452 ± 0.05912	0.68549 ± 0.03402
AttrMask	0.79270 ± 0.04602	0.75923 ± 0.04253	0.66740 ± 0.07382	0.77554 ± 0.06069	0.57280 ± 0.04643
EdgePred	0.80540 ± 0.03729	0.84311 ± 0.02268	0.52183 ± 0.06837	0.76211 ± 0.09944	0.64343 ± 0.03269
3D-InfoMax	0.86429 ± 0.02117	0.84784 ± 0.04550	0.73786 ± 0.05449	0.95287 ± 0.03245	0.72674 ± 0.03527
GraphMVP	0.89266 ± 0.01793	0.84393 ± 0.03335	0.70871 ± 0.09621	0.85443 ± 0.09936	0.72244 ± 0.04464
MolCLR-GIN	0.80928 ± 0.03771	0.76488 ± 0.05190	0.67051 ± 0.07296	0.84067 ± 0.05639	0.65875 ± 0.05171
MolCLR-GCN	0.80959 ± 0.02423	0.71093 ± 0.02907	0.60054 ± 0.04116	0.79088 ± 0.07192	0.59081 ± 0.04505
GCC	0.81776 ± 0.02789	0.76025 ± 0.03248	0.64666 ± 0.08172	0.81471 ± 0.01875	0.61126 ± 0.05150
GPT-GNN	0.82313 ± 0.03331	0.74369 ± 0.00969	0.61092 ± 0.07835	0.68818 ± 0.11327	0.59126 ± 0.04808
InfoGraph	0.74994 ± 0.02344	0.64072 ± 0.03404	0.61278 ± 0.09309	0.65361 ± 0.17712	0.55392 ± 0.02738
JOAO	0.82093 ± 0.02649	0.73460 ± 0.04613	0.62356 ± 0.06018	0.69088 ± 0.18888	0.55508 ± 0.07295
JOAOv2	0.83378 ± 0.01333	0.76005 ± 0.04110	0.67037 ± 0.05863	0.81605 ± 0.10417	0.59945 ± 0.06486
PosPred	0.81588 ± 0.02780	0.77963 ± 0.02785	0.64703 ± 0.07205	0.84639 ± 0.05662	0.59737 ± 0.02881
TransM	0.88635 ± 0.02315	0.86182 ± 0.04111	0.68802 ± 0.06348	0.86296 ± 0.02623	0.62168 ± 0.05757
MPG	0.89088 ± 0.01615	0.88938 ± 0.02994	0.70285 ± 0.04788	0.95057 ± 0.02789	0.72888 ± 0.01678
KANO	0.88800 ± 0.01798	0.83099 ± 0.03786	0.67394 ± 0.08316	0.78674 ± 0.06856	0.66976 ± 0.05434

Prediction is evaluated by ROC-AUC. For each method, the mean and standard deviation are reported for the evaluation metrics.

Supplementary Table 24. Molecular property classification task (random split) – Part II

Method	Pgp	BBB	CYP1A2 Veith	CYP2C19 Veith	CYP2C9 Substrate
qcGEM	0.94426 ± 0.01035	0.89914 ± 0.00751	0.92951 ± 0.00659	0.89208 ± 0.00470	0.76030 ± 0.09498
ECFP	0.91424 ± 0.02455	0.84811 ± 0.02589	0.89805 ± 0.00616	0.85869 ± 0.00985	0.67369 ± 0.09622
FCFP	0.91380 ± 0.04051	0.86491 ± 0.01792	0.90252 ± 0.01172	0.86001 ± 0.01414	0.61315 ± 0.03780
AvalonFP	0.91587 ± 0.02578	0.88941 ± 0.01573	0.91096 ± 0.00471	0.85649 ± 0.00918	0.66698 ± 0.09015
TTGEN	0.90330 ± 0.01458	0.82515 ± 0.02016	0.89438 ± 0.00960	0.84748 ± 0.00971	0.71407 ± 0.06974
APGEN	0.92687 ± 0.02365	0.87023 ± 0.00847	0.89383 ± 0.00502	0.83694 ± 0.00814	0.64979 ± 0.10017
RDKFP	0.89897 ± 0.03032	0.89956 ± 0.01479	0.90695 ± 0.00466	0.85127 ± 0.00887	0.67941 ± 0.09652
MACCS	0.90039 ± 0.02627	0.90181 ± 0.01329	0.90998 ± 0.00831	0.86680 ± 0.00597	0.69481 ± 0.08372
LayeredFP	0.90208 ± 0.02080	0.86900 ± 0.01507	0.88067 ± 0.01019	0.82270 ± 0.00809	0.65175 ± 0.07213
PatternFP	0.90510 ± 0.02139	0.87925 ± 0.01326	0.90799 ± 0.00858	0.85517 ± 0.00911	0.68441 ± 0.09139
Molformer	0.92951 ± 0.02570	0.89510 ± 0.01168	0.92520 ± 0.01048	0.88559 ± 0.00529	0.70849 ± 0.06089
GROVER-Base	0.93893 ± 0.01893	0.88287 ± 0.02756	0.93051 ± 0.00548	0.89030 ± 0.00403	0.69560 ± 0.09696
GROVER-Large	0.93813 ± 0.01900	0.88287 ± 0.02608	0.92870 ± 0.00699	0.89156 ± 0.00727	0.74080 ± 0.07440
MoleBERT	0.90931 ± 0.03927	0.85879 ± 0.01028	0.95790 ± 0.00243	0.93645 ± 0.00533	0.68864 ± 0.12179
UniMol-G	0.91972 ± 0.01097	0.84219 ± 0.01672	0.90706 ± 0.00388	0.84970 ± 0.00593	0.59299 ± 0.09376
UniMol-GA	0.93458 ± 0.01274	0.85263 ± 0.01334	0.91944 ± 0.00302	0.86138 ± 0.00613	0.58065 ± 0.07304
3D-EMGP	0.85513 ± 0.01556	0.61568 ± 0.06089	0.61974 ± 0.01405	0.51492 ± 0.01494	0.49266 ± 0.09093
GraphCL	0.87514 ± 0.01404	0.84205 ± 0.02117	0.87677 ± 0.00683	0.85877 ± 0.00322	0.69534 ± 0.07610
AttrMask	0.81122 ± 0.03740	0.79065 ± 0.02965	0.84900 ± 0.00975	0.79535 ± 0.01040	0.59891 ± 0.07031
EdgePred	0.86518 ± 0.04177	0.80851 ± 0.03065	0.85451 ± 0.00580	0.80600 ± 0.00655	0.60922 ± 0.05153
3D-InfoMax	0.93754 ± 0.01680	0.87087 ± 0.01347	0.91340 ± 0.00862	0.87670 ± 0.00675	0.63880 ± 0.05365
GraphMVP	0.91524 ± 0.02583	0.87478 ± 0.01847	0.91470 ± 0.00528	0.87935 ± 0.00728	0.69527 ± 0.10367
MolCLR-GIN	0.81975 ± 0.02777	0.81233 ± 0.03635	0.87727 ± 0.00983	0.84602 ± 0.00535	0.68226 ± 0.05770
MolCLR-GCN	0.86234 ± 0.04175	0.80873 ± 0.02662	0.86720 ± 0.00726	0.82838 ± 0.00643	0.59281 ± 0.11254
GCC	0.88355 ± 0.02129	0.81980 ± 0.01837	0.87104 ± 0.00472	0.81894 ± 0.00564	0.63313 ± 0.04470
GPT-GNN	0.89644 ± 0.02176	0.78967 ± 0.01507	0.86321 ± 0.00544	0.79579 ± 0.00599	0.66400 ± 0.11494
InfoGraph	0.87963 ± 0.02454	0.74102 ± 0.04177	0.82001 ± 0.00658	0.72314 ± 0.00957	0.55027 ± 0.06831
JOAO	0.87009 ± 0.02125	0.77099 ± 0.01505	0.86602 ± 0.00668	0.78166 ± 0.00698	0.62766 ± 0.09265
JOAOv2	0.88968 ± 0.01232	0.81628 ± 0.04060	0.88088 ± 0.00487	0.81155 ± 0.00889	0.60864 ± 0.09358
PosPred	0.89537 ± 0.01748	0.76218 ± 0.02510	0.88784 ± 0.00699	0.80104 ± 0.00859	0.63322 ± 0.10962
TransM	0.93448 ± 0.02263	0.87425 ± 0.01198	0.91712 ± 0.00491	0.86438 ± 0.00688	0.67405 ± 0.06516
MPG	0.93185 ± 0.02695	0.89962 ± 0.01058	0.91822 ± 0.00573	0.89142 ± 0.00642	0.70518 ± 0.05887
KANO	0.92068 ± 0.02736	0.86132 ± 0.04093	0.87641 ± 0.01130	0.81717 ± 0.00693	0.63945 ± 0.06915

Prediction is evaluated by ROC-AUC. For each method, the mean and standard deviation are reported for the evaluation metrics.

Supplementary Table 25. Molecular property classification task (random split) – Part III

Method	CYP2C9 Veith	CYP2D6 Substrate	CYP2D6 Veith	CYP3A4 Substrate	CYP3A4 Veith
qcGEM	0.88708 ± 0.00357	0.79447 ± 0.03874	0.86694 ± 0.01189	0.66324 ± 0.07009	0.88745 ± 0.00455
ECFP	0.86288 ± 0.00820	0.74665 ± 0.05353	0.83128 ± 0.00963	0.66103 ± 0.06554	0.86307 ± 0.00698
FCFP	0.86254 ± 0.01096	0.74284 ± 0.03554	0.83639 ± 0.01544	0.61654 ± 0.06696	0.86561 ± 0.00470
AvalonFP	0.86345 ± 0.00511	0.73339 ± 0.06803	0.83294 ± 0.02116	0.60327 ± 0.08013	0.86347 ± 0.00766
TTGEN	0.86313 ± 0.00787	0.74331 ± 0.03761	0.81810 ± 0.01664	0.60934 ± 0.07903	0.86117 ± 0.00594
APGEN	0.83977 ± 0.01199	0.73749 ± 0.05759	0.80893 ± 0.01447	0.66242 ± 0.07896	0.84103 ± 0.01386
RDKFP	0.86033 ± 0.00502	0.78884 ± 0.03882	0.83627 ± 0.00452	0.61822 ± 0.03695	0.85757 ± 0.00869
MACCS	0.86170 ± 0.00559	0.76839 ± 0.04350	0.84663 ± 0.01341	0.64738 ± 0.07787	0.85983 ± 0.00946
LayeredFP	0.82849 ± 0.00694	0.72631 ± 0.06011	0.80426 ± 0.01261	0.58468 ± 0.05604	0.82988 ± 0.00615
PatternFP	0.85123 ± 0.00685	0.73923 ± 0.05386	0.83479 ± 0.01026	0.66587 ± 0.07908	0.85648 ± 0.00491
Molformer	0.88095 ± 0.00610	0.78422 ± 0.03612	0.85208 ± 0.01642	0.65973 ± 0.06335	0.88060 ± 0.00528
GROVER-Base	0.88605 ± 0.00369	0.73865 ± 0.14698	0.86679 ± 0.00715	0.59201 ± 0.06816	0.88234 ± 0.00520
GROVER-Large	0.88129 ± 0.00603	0.71352 ± 0.14100	0.86519 ± 0.01646	0.59457 ± 0.07257	0.88471 ± 0.00829
MoleBERT	0.93399 ± 0.00401	0.77824 ± 0.05194	0.92082 ± 0.01148	0.66080 ± 0.07667	0.93269 ± 0.00879
UniMol-G	0.85856 ± 0.00399	0.75682 ± 0.02323	0.82208 ± 0.01210	0.64319 ± 0.07155	0.85726 ± 0.00935
UniMol-GA	0.87338 ± 0.00418	0.73906 ± 0.09421	0.84236 ± 0.01350	0.64275 ± 0.07184	0.86856 ± 0.00598
3D-EMGP	0.61522 ± 0.01277	0.48403 ± 0.06009	0.54031 ± 0.02502	0.54556 ± 0.09048	0.68439 ± 0.01596
GraphCL	0.84758 ± 0.00548	0.68373 ± 0.08209	0.81870 ± 0.01041	0.57247 ± 0.02162	0.84892 ± 0.00660
AttrMask	0.79597 ± 0.00534	0.73926 ± 0.05293	0.74837 ± 0.02384	0.57903 ± 0.07403	0.76392 ± 0.01294
EdgePred	0.82237 ± 0.01494	0.70223 ± 0.04119	0.77479 ± 0.01431	0.62209 ± 0.05421	0.81076 ± 0.00470
3D-InfoMax	0.87035 ± 0.00654	0.73631 ± 0.03223	0.85255 ± 0.01097	0.61218 ± 0.09602	0.87979 ± 0.00718
GraphMVP	0.87565 ± 0.00279	0.78144 ± 0.05635	0.85543 ± 0.01192	0.66069 ± 0.09177	0.87884 ± 0.00219
MolCLR-GIN	0.83247 ± 0.00707	0.74044 ± 0.10108	0.79929 ± 0.01822	0.58815 ± 0.05671	0.80678 ± 0.01750
MolCLR-GCN	0.83002 ± 0.00912	0.71434 ± 0.03092	0.78426 ± 0.01468	0.60855 ± 0.06888	0.78784 ± 0.01824
GCC	0.82025 ± 0.01161	0.71089 ± 0.04490	0.78597 ± 0.01307	0.58268 ± 0.06779	0.81187 ± 0.00725
GPT-GNN	0.80318 ± 0.00884	0.65753 ± 0.02888	0.75407 ± 0.00965	0.64686 ± 0.06508	0.79216 ± 0.01386
InfoGraph	0.72654 ± 0.01123	0.68847 ± 0.08472	0.70063 ± 0.02230	0.59319 ± 0.06402	0.74937 ± 0.00972
JOAO	0.79364 ± 0.00811	0.72586 ± 0.08993	0.74079 ± 0.01531	0.61935 ± 0.08860	0.78092 ± 0.00848
JOAOv2	0.81608 ± 0.01265	0.71623 ± 0.02913	0.76859 ± 0.01502	0.61083 ± 0.07450	0.81188 ± 0.01025
PosPred	0.81533 ± 0.00810	0.71888 ± 0.04541	0.76975 ± 0.00950	0.60997 ± 0.08831	0.80584 ± 0.00992
TransM	0.86553 ± 0.00688	0.76704 ± 0.02161	0.83316 ± 0.01369	0.64620 ± 0.04361	0.85440 ± 0.00731
MPG	0.88526 ± 0.00212	0.79259 ± 0.04665	0.86219 ± 0.01596	0.60952 ± 0.07876	0.88492 ± 0.00790
KANO	0.83440 ± 0.00451	0.76570 ± 0.04573	0.79097 ± 0.01989	0.62508 ± 0.07355	0.81499 ± 0.01259

Prediction is evaluated by ROC-AUC. For each method, the mean and standard deviation are reported for the evaluation metrics.

Supplementary Table 26. Molecular property classification task (random split) – Part IV

Method	Ames	Carcinogens	DILI	hERG Blockers	SkinReac
qcGEM	0.89796 ± 0.01966	0.80704 ± 0.14627	0.93957 ± 0.00620	0.86849 ± 0.07201	0.73921 ± 0.04738
ECFP	0.86396 ± 0.00918	0.70229 ± 0.22481	0.86088 ± 0.05118	0.82787 ± 0.05795	0.71463 ± 0.05263
FCFP	0.86678 ± 0.01783	0.74574 ± 0.06457	0.85816 ± 0.04497	0.82462 ± 0.05422	0.63935 ± 0.12288
AvalonFP	0.88725 ± 0.01384	0.72827 ± 0.11220	0.91626 ± 0.01521	0.78318 ± 0.05968	0.68901 ± 0.11340
TTGEN	0.84048 ± 0.01125	0.73277 ± 0.11558	0.85636 ± 0.01544	0.78930 ± 0.06832	0.64242 ± 0.10494
APGEN	0.84975 ± 0.01126	0.74707 ± 0.18948	0.86868 ± 0.02205	0.83618 ± 0.04540	0.72088 ± 0.11242
RDKFP	0.87645 ± 0.01601	0.69198 ± 0.07617	0.89680 ± 0.03083	0.79252 ± 0.07076	0.74857 ± 0.16493
MACCS	0.87920 ± 0.01255	0.77838 ± 0.08905	0.92789 ± 0.01686	0.83145 ± 0.05147	0.70327 ± 0.05465
LayeredFP	0.87223 ± 0.01395	0.70951 ± 0.17662	0.89331 ± 0.04004	0.82392 ± 0.04912	0.80695 ± 0.01533
PatternFP	0.87814 ± 0.01540	0.72355 ± 0.14295	0.86724 ± 0.02693	0.79149 ± 0.06378	0.80360 ± 0.07048
Molformer	0.86982 ± 0.01649	0.72213 ± 0.16159	0.90792 ± 0.01239	0.86209 ± 0.06817	0.83618 ± 0.07236
GROVER-Base	0.88703 ± 0.00924	0.83357 ± 0.12821	0.92174 ± 0.01021	0.84369 ± 0.08548	0.71119 ± 0.04778
GROVER-Large	0.88666 ± 0.01500	0.85374 ± 0.12123	0.90980 ± 0.00520	0.83736 ± 0.08514	0.73219 ± 0.04318
MoleBERT	0.85506 ± 0.01000	0.84264 ± 0.07985	0.87726 ± 0.04336	0.83150 ± 0.09133	0.80275 ± 0.07529
UniMol-G	0.85306 ± 0.01462	0.78971 ± 0.12358	0.86635 ± 0.05076	0.81858 ± 0.05024	0.73237 ± 0.09836
UniMol-GA	0.86174 ± 0.01811	0.81580 ± 0.11310	0.89095 ± 0.05743	0.83054 ± 0.04183	0.71249 ± 0.08617
3D-EMGP	0.56491 ± 0.02852	0.27726 ± 0.02949	0.45749 ± 0.05462	0.73660 ± 0.07400	0.54084 ± 0.10784
GraphCL	0.82854 ± 0.01522	0.71761 ± 0.15979	0.79302 ± 0.04395	0.78158 ± 0.06948	0.64525 ± 0.10687
AttrMask	0.76187 ± 0.01231	0.82917 ± 0.05930	0.81999 ± 0.02636	0.80976 ± 0.07557	0.51835 ± 0.06892
EdgePred	0.75005 ± 0.01903	0.71852 ± 0.10802	0.83184 ± 0.06916	0.74653 ± 0.08377	0.70952 ± 0.05425
3D-InfoMax	0.87823 ± 0.01394	0.78684 ± 0.08126	0.91050 ± 0.01187	0.85827 ± 0.05831	0.71209 ± 0.10760
GraphMVP	0.86579 ± 0.01945	0.72316 ± 0.11064	0.90088 ± 0.04086	0.86256 ± 0.05613	0.68886 ± 0.05388
MolCLR-GIN	0.81219 ± 0.01996	0.78505 ± 0.12202	0.89592 ± 0.02283	0.78990 ± 0.10033	0.68670 ± 0.05024
MolCLR-GCN	0.81616 ± 0.01833	0.80655 ± 0.10888	0.89545 ± 0.01028	0.78619 ± 0.07183	0.62674 ± 0.06655
GCC	0.79705 ± 0.01279	0.78591 ± 0.10842	0.84897 ± 0.06070	0.76999 ± 0.05219	0.62503 ± 0.10013
GPT-GNN	0.80641 ± 0.00633	0.74413 ± 0.12926	0.74359 ± 0.11771	0.81640 ± 0.03734	0.57520 ± 0.09959
InfoGraph	0.67952 ± 0.01025	0.51433 ± 0.08826	0.64526 ± 0.08458	0.80907 ± 0.03934	0.66681 ± 0.08827
JOAO	0.79560 ± 0.01569	0.67197 ± 0.20318	0.77540 ± 0.12059	0.78284 ± 0.09488	0.66472 ± 0.08813
JOAOv2	0.80737 ± 0.01626	0.67369 ± 0.16287	0.91094 ± 0.05012	0.83429 ± 0.06782	0.66644 ± 0.07133
PosPred	0.79768 ± 0.02143	0.69808 ± 0.15952	0.83855 ± 0.03696	0.83759 ± 0.06202	0.69387 ± 0.09499
TransM	0.87983 ± 0.01803	0.77668 ± 0.15068	0.87976 ± 0.02252	0.80645 ± 0.04814	0.72923 ± 0.06683
MPG	0.87133 ± 0.01484	0.77636 ± 0.08112	0.92235 ± 0.02237	0.82405 ± 0.08711	0.80899 ± 0.02571
KANO	0.78385 ± 0.01749	0.73911 ± 0.15443	0.89443 ± 0.03333	0.83459 ± 0.09836	0.68132 ± 0.07689

Prediction is evaluated by ROC-AUC. For each method, the mean and standard deviation are reported for the evaluation metrics.

Supplementary Table 27. Molecular property classification task (random split) – Part V

Method	AMES(PharmaBench)	BBB(PharmaBench)
qcGEM	0.91342 ± 0.00801	0.92243 ± 0.00930
ECFP	0.88246 ± 0.00595	0.91447 ± 0.01054
FCFP	0.87904 ± 0.00616	0.91918 ± 0.00883
AvalonFP	0.89869 ± 0.00403	0.92878 ± 0.01112
TTGEN	0.85472 ± 0.00699	0.91526 ± 0.00579
APGEN	0.86179 ± 0.00845	0.90413 ± 0.00913
RDKFP	0.89152 ± 0.00624	0.90783 ± 0.01621
MACCS	0.89477 ± 0.00520	0.92030 ± 0.01008
LayeredFP	0.87548 ± 0.01097	0.90554 ± 0.00518
PatternFP	0.89251 ± 0.00856	0.91395 ± 0.01250
Molformer	0.88491 ± 0.01064	0.91929 ± 0.01289
GROVER-Base	0.89957 ± 0.01109	0.92646 ± 0.01061
GROVER-Large	0.90425 ± 0.00705	0.92633 ± 0.01159
MoleBERT	0.86789 ± 0.00793	0.89656 ± 0.00837
UniMol-G	0.86078 ± 0.01247	0.89125 ± 0.01560
UniMol-GA	0.87663 ± 0.00756	0.89990 ± 0.01727
3D-EMGP	0.59504 ± 0.01137	0.62552 ± 0.02361
GraphCL	0.83937 ± 0.00880	0.88029 ± 0.01691
AttrMask	0.77293 ± 0.01350	0.83526 ± 0.01421
EdgePred	0.76048 ± 0.01635	0.86306 ± 0.01720
3D-InfoMax	0.88778 ± 0.00355	0.91298 ± 0.01278
GraphMVP	0.87898 ± 0.00407	0.91423 ± 0.00838
MolCLR-GIN	0.83493 ± 0.00201	0.87413 ± 0.01068
MolCLR-GCN	0.83300 ± 0.00888	0.86046 ± 0.01630
GCC	0.79328 ± 0.00753	0.86231 ± 0.02490
GPT-GNN	0.71478 ± 0.01678	0.82585 ± 0.01885
InfoGraph	0.69747 ± 0.02397	0.82220 ± 0.01555
JOAO	0.65894 ± 0.01793	0.68790 ± 0.02282
JOAOv2	0.74938 ± 0.00769	0.83351 ± 0.02101
PosPred	0.60378 ± 0.01693	0.58037 ± 0.01867
TransM	0.88943 ± 0.00373	0.91862 ± 0.01113
MPG	0.88670 ± 0.00862	0.91771 ± 0.01050
KANO	0.79763 ± 0.01132	0.89442 ± 0.01679

Prediction is evaluated by ROC-AUC. For each method, the mean and standard deviation are reported for the evaluation metrics.

3.3.2 Activity cliff prediction

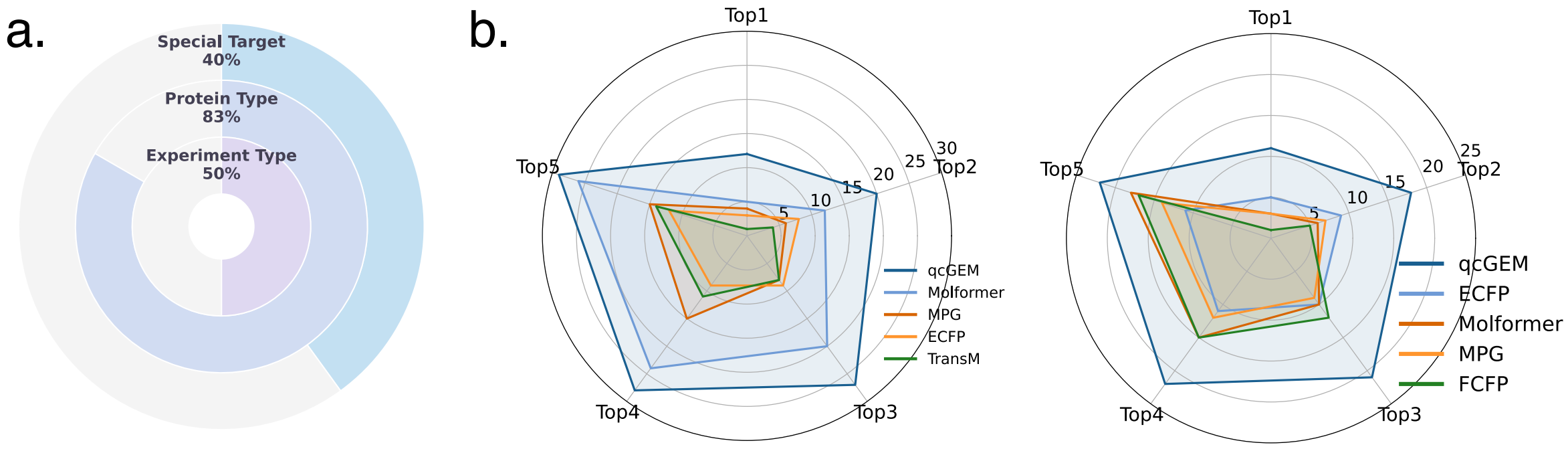

Supplementary Figure 11. Ranking evaluation of qcGEM in activity cliff tasks. a) This figure supplements Figure 3d, with the focus shifted to activity cliff molecules instead of all molecules. Specifically, the multi-layer pie chart illustrates proportions of qcGEM achieving the best ranking for activity cliff molecules in 30 tasks under three task classification criteria: individual proteins targets (outer), protein classes (middle), and experimental categories (inner). **b)** The radar plot on the left shows the frequency with which each method appears in the top 1-5 rankings for all molecules, while the plot on the right shows the results for activity cliff molecules.

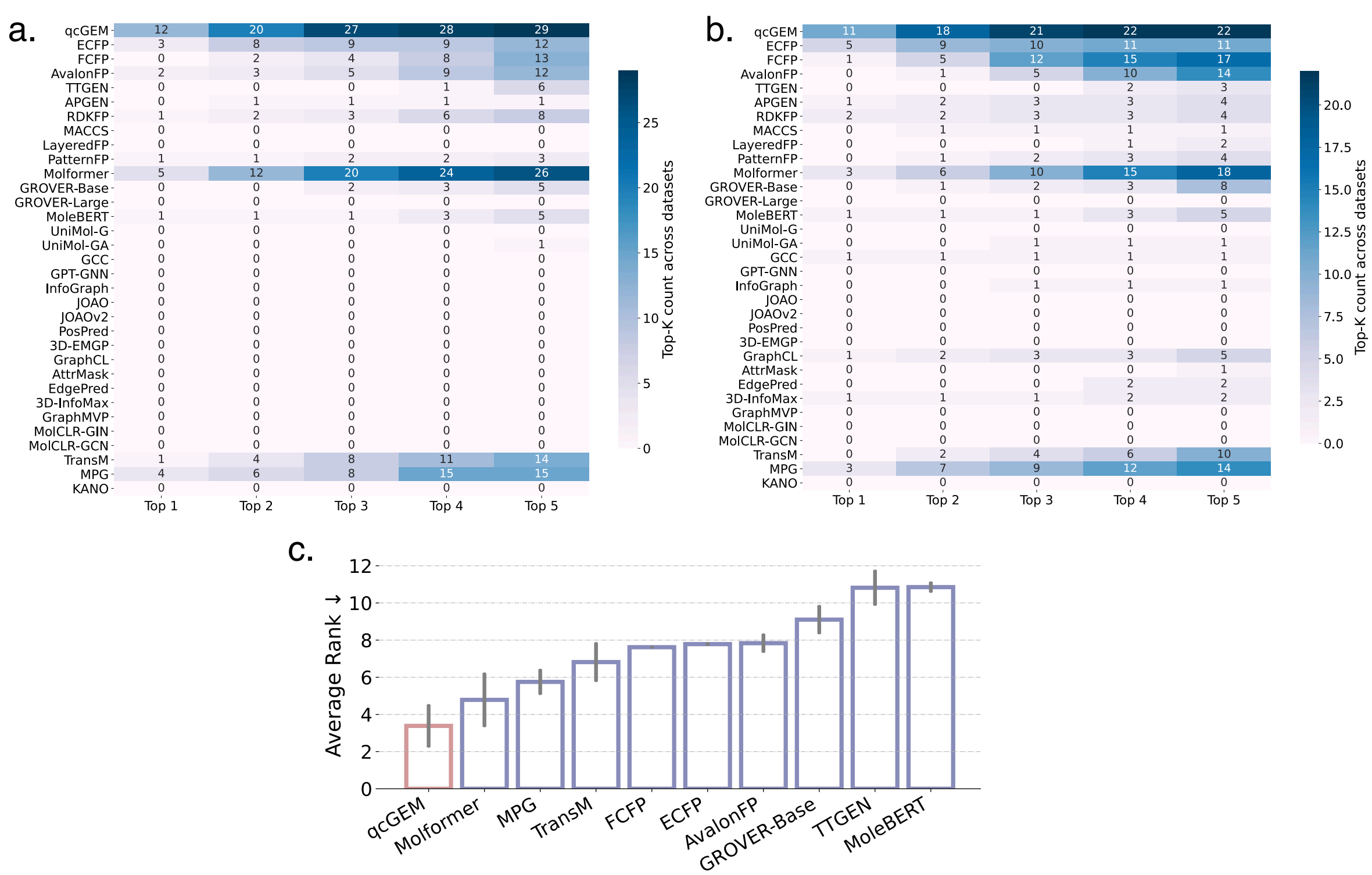

Supplementary Figure 12. Statistics of the top 1-5 rankings for all methods in activity cliff tasks. a) Evaluation results of qcGEM and baseline methods on all molecules. **b)** Evaluation results of qcGEM and baseline methods on activity cliff molecules. **c)** The overall average rank of the top methods.

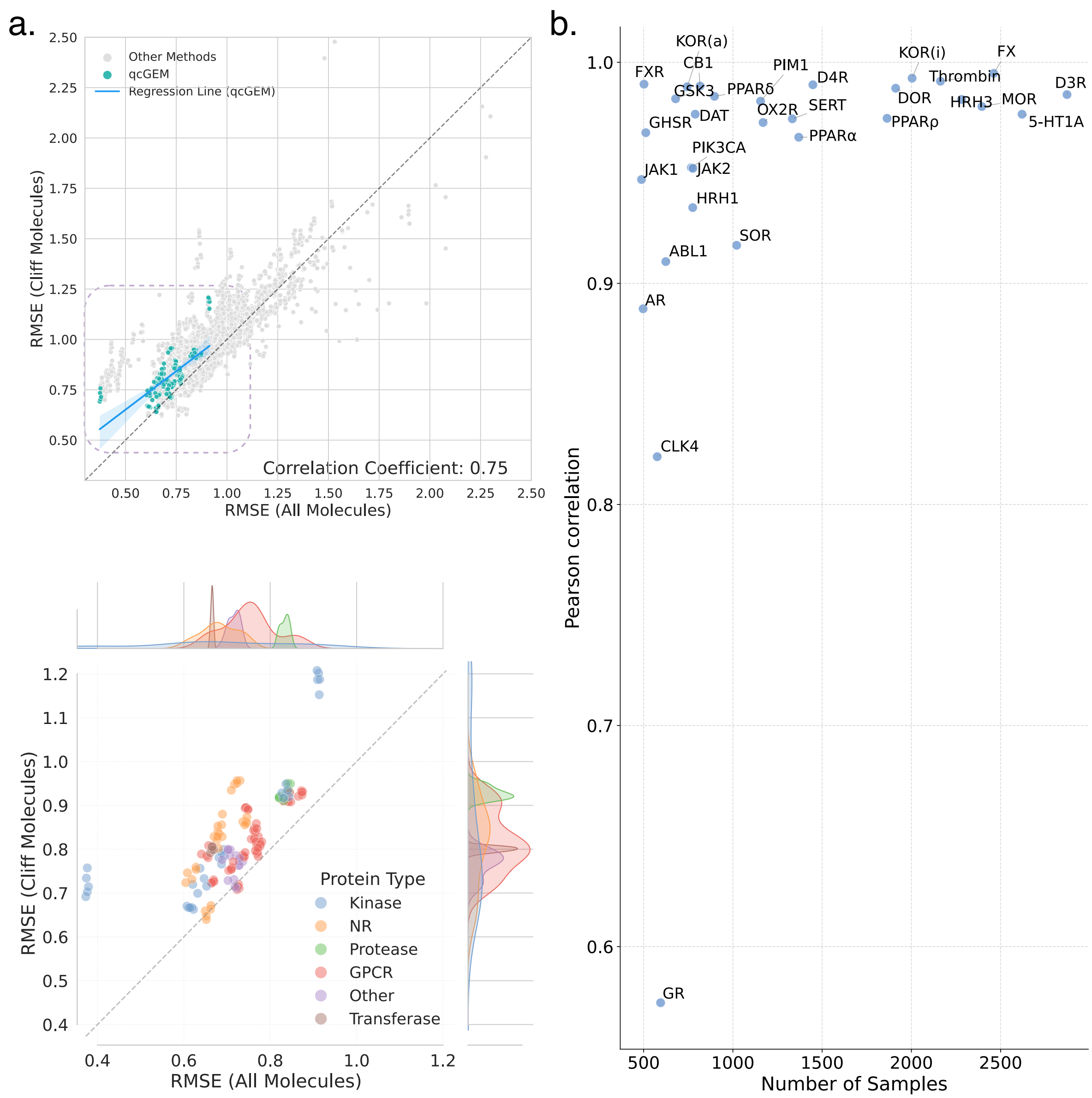

Supplementary Figure 13. Additional results of qcGEM in activity cliff tasks. a) The upper scatter plot compares the prediction error (in RMSE) on all molecules (horizontal axis) vs. activity cliff molecules (vertical axis). Each point refers to a protein target. qcGEM results are highlighted in teal, while the other methods are shown in gray, with the dashed diagonal indicating equal performance between the two subsets for ideal methods with perfect generalizability. A fitted regression line for qcGEM (blue) demonstrates the positive correlation (Pearson correlation coefficient r = 0.75) between the two groups of molecules. The lower scatter plot (corresponding to the dash-line enclosed region in the upper plot) provides a detailed view of qcGEM results. Here, protein targets are colored by their protein classes: kinase, NR, protease, GPCR, transferase, and other. Kernel density estimates are shown along the margins to highlight the distribution of RMSE values. **b)** Correlation between inference values of models optimized on all and activity cliff molecules. Each point represents one protein target, with the horizontal and vertical axes showing the number of available samples and the Pearson correlation coefficient, respectively.

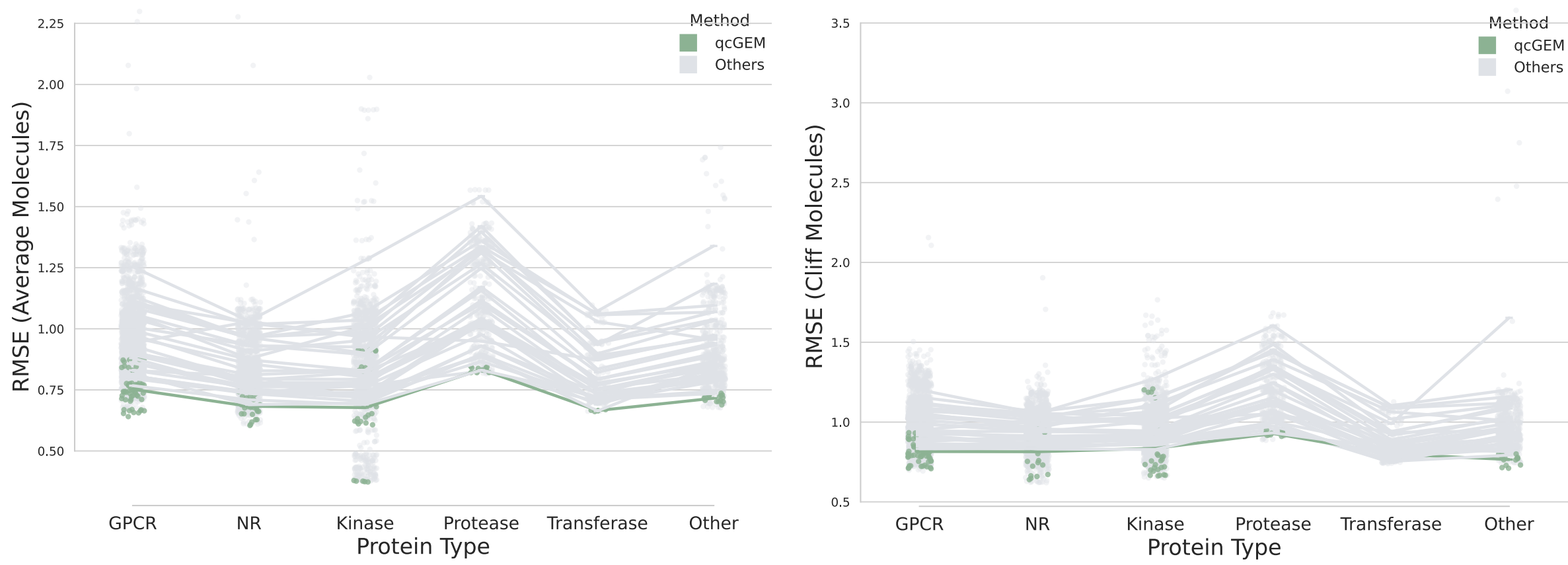

Supplementary Figure 14. Evaluation of qcGEM vs. other methods among the 6 protein types in the activity cliff prediction. The prediction error (in RMSE) is compared for qcGEM (colored in green) and other methods (colored in gray) for all molecules and activity cliff molecules in the left and right panels, respectively. For each protein type, results on individual protein targets are presented as dots, whereas the mean results are connected by solid lines across protein types.

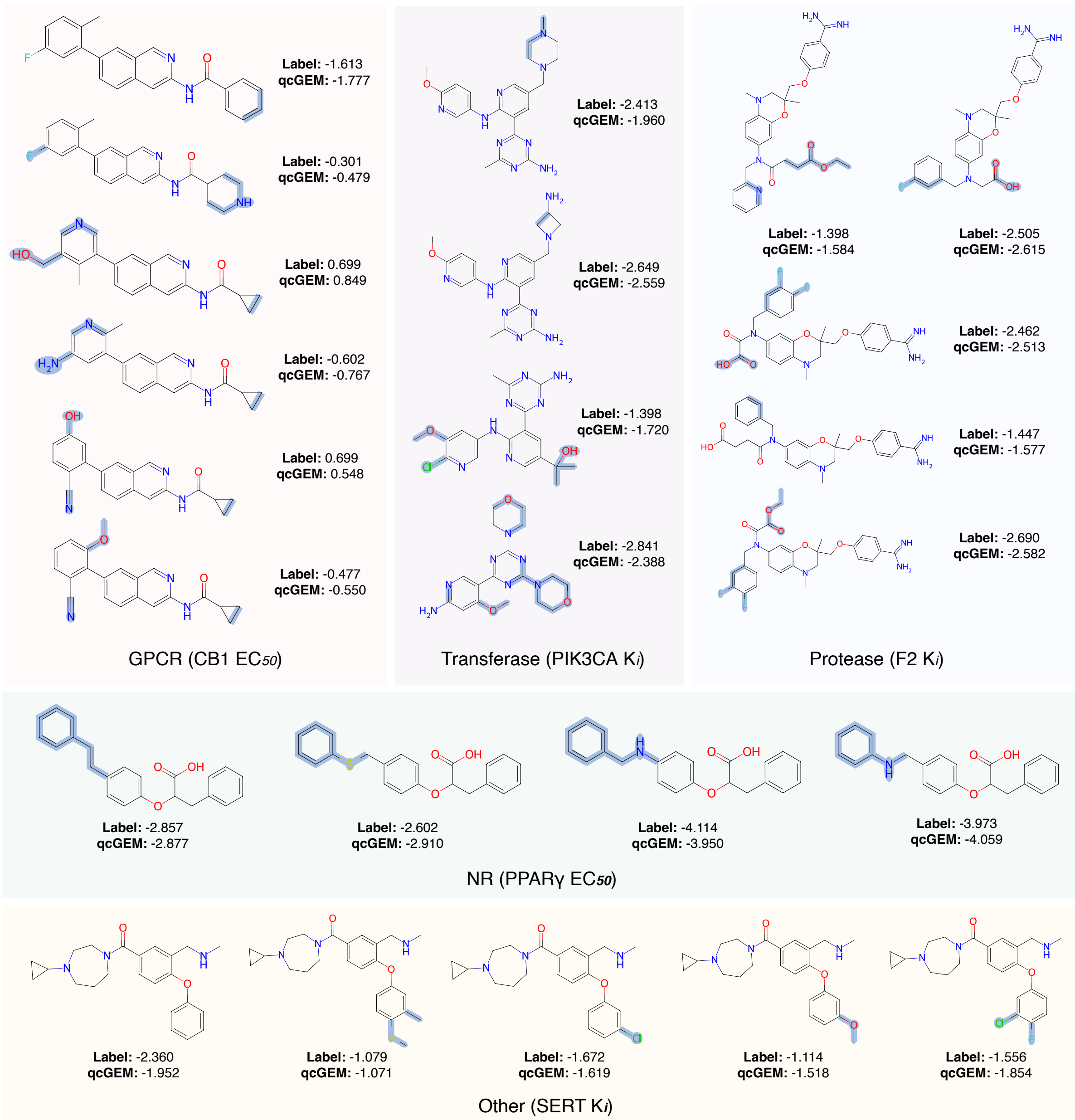

Supplementary Figure 15. Examples of the identification of activity cliff molecules for different kinds of protein targets. As a supplement to the kinase target in Figure 3e, this figure presents activity cliff molecules for five protein targets falling in the other protein classes (GPCR, protease, NR, transferase, and other). Among ligands targeted to the same protein, difference in chemical structure is highlighted in blue shadow for each ligand, while the ground-truth binding affinity and prediction based on qcGEM are listed aside.

Supplementary Table 28. Activity cliff prediction (all molecules) – Part I

Method	CHEMBL1862 K_i	CHEMBL1871 K_i	CHEMBL2034 K_i	CHEMBL2047 EC_{50}	CHEMBL204 K_i
qcGEM	0.68837 $\pm$ 0.00473	0.68083 $\pm$ 0.00776	0.72052 $\pm$ 0.00753	0.65562 $\pm$ 0.00612	0.84185 $\pm$ 0.00343
ECFP	0.88849 $\pm$ 0.00337	0.74889 $\pm$ 0.00497	0.77455 $\pm$ 0.00600	0.68553 $\pm$ 0.00316	0.82639 $\pm$ 0.00975
FCFP	0.86827 $\pm$ 0.01063	0.75576 $\pm$ 0.00319	0.74120 $\pm$ 0.00556	0.69238 $\pm$ 0.00454	0.89283 $\pm$ 0.00454
AvalonFP	0.78049 $\pm$ 0.00636	0.76487 $\pm$ 0.00173	0.74495 $\pm$ 0.00282	0.75196 $\pm$ 0.00279	0.88351 $\pm$ 0.00397
TTGEN	0.81539 $\pm$ 0.00988	0.74161 $\pm$ 0.00295	0.82475 $\pm$ 0.00612	0.68361 $\pm$ 0.00370	0.95722 $\pm$ 0.00342
APGEN	0.82297 $\pm$ 0.01898	0.67537 $\pm$ 0.00779	0.77883 $\pm$ 0.00365	0.69447 $\pm$ 0.00248	1.07413 $\pm$ 0.00467
RDKFP	0.78331 $\pm$ 0.00067	0.75317 $\pm$ 0.00116	0.78361 $\pm$ 0.00225	0.77218 $\pm$ 0.00273	0.92334 $\pm$ 0.00236
MACCS	0.89220 $\pm$ 0.00635	0.76879 $\pm$ 0.00326	0.75045 $\pm$ 0.00609	0.73933 $\pm$ 0.00516	1.04168 $\pm$ 0.01058
LayeredFP	0.80692 $\pm$ 0.00641	0.73840 $\pm$ 0.00194	0.75335 $\pm$ 0.00668	0.77042 $\pm$ 0.00344	1.00646 $\pm$ 0.00273
PatternFP	0.64121 $\pm$ 0.00233	0.78583 $\pm$ 0.00354	0.82129 $\pm$ 0.00404	0.72117 $\pm$ 0.00574	1.02974 $\pm$ 0.00098
Molformer	0.72662 $\pm$ 0.01024	0.73434 $\pm$ 0.00231	0.73806 $\pm$ 0.00280	0.66572 $\pm$ 0.00362	0.82375 $\pm$ 0.00395
GROVER-Base	0.69721 $\pm$ 0.01318	0.74083 $\pm$ 0.00873	0.75336 $\pm$ 0.01329	0.78317 $\pm$ 0.01684	0.95848 $\pm$ 0.05063
GROVER-Large	1.10110 $\pm$ 0.43252	1.05922 $\pm$ 0.43046	0.88320 $\pm$ 0.12424	1.33243 $\pm$ 0.77472	0.99466 $\pm$ 0.07448
MoleBERT	0.73701 $\pm$ 0.00223	0.68395 $\pm$ 0.00189	0.81688 $\pm$ 0.00349	0.67699 $\pm$ 0.00127	1.09724 $\pm$ 0.00090
UniMol-G	0.82023 $\pm$ 0.00404	0.75638 $\pm$ 0.00126	0.84075 $\pm$ 0.00242	0.84976 $\pm$ 0.00389	1.21497 $\pm$ 0.00235
UniMol-GA	0.78656 $\pm$ 0.00441	0.75998 $\pm$ 0.00294	0.82143 $\pm$ 0.00189	0.84057 $\pm$ 0.00607	1.16230 $\pm$ 0.00412
GCC	0.90042 $\pm$ 0.00551	0.80155 $\pm$ 0.00339	0.88180 $\pm$ 0.00258	0.81417 $\pm$ 0.00288	1.26442 $\pm$ 0.00186
GPT-GNN	1.00079 $\pm$ 0.02296	0.83967 $\pm$ 0.00945	0.83682 $\pm$ 0.00761	0.88350 $\pm$ 0.01736	1.25197 $\pm$ 0.01212
GraphCL	0.74777 $\pm$ 0.00502	0.73633 $\pm$ 0.00275	0.78863 $\pm$ 0.00253	0.71186 $\pm$ 0.00610	1.05354 $\pm$ 0.00337
InfoGraph	1.06380 $\pm$ 0.00703	0.90989 $\pm$ 0.00288	0.88276 $\pm$ 0.00332	0.91066 $\pm$ 0.00378	1.41360 $\pm$ 0.00353
JOAO	0.99353 $\pm$ 0.00462	0.99248 $\pm$ 0.01508	0.86776 $\pm$ 0.00310	0.87934 $\pm$ 0.00583	1.33611 $\pm$ 0.00184
JOAOv2	1.17222 $\pm$ 0.01736	0.82397 $\pm$ 0.00408	0.83188 $\pm$ 0.00248	0.86386 $\pm$ 0.00560	1.35854 $\pm$ 0.00556
PosPred	1.17485 $\pm$ 0.01624	0.98954 $\pm$ 0.01114	0.85229 $\pm$ 0.00465	1.28644 $\pm$ 0.29285	1.37893 $\pm$ 0.02275
3D-EMGP	1.52198 $\pm$ 0.00266	1.02511 $\pm$ 0.00093	0.98371 $\pm$ 0.00004	0.94654 $\pm$ 0.00076	1.51749 $\pm$ 0.00183
3D-InfoMax	0.86640 $\pm$ 0.00726	0.73443 $\pm$ 0.00189	0.75975 $\pm$ 0.00252	0.74698 $\pm$ 0.00392	1.06734 $\pm$ 0.00480
AttrMask	1.10877 $\pm$ 0.00183	0.83746 $\pm$ 0.00132	0.94840 $\pm$ 0.00092	0.88104 $\pm$ 0.00105	1.43098 $\pm$ 0.00166
EdgePred	0.85584 $\pm$ 0.00459	0.83753 $\pm$ 0.00101	0.75669 $\pm$ 0.00285	0.70930 $\pm$ 0.00244	1.13453 $\pm$ 0.00145
GraphMVP	0.88323 $\pm$ 0.00312	0.73353 $\pm$ 0.00106	0.87163 $\pm$ 0.00116	0.74174 $\pm$ 0.00136	1.18659 $\pm$ 0.00271
MolCLR-GIN	0.98850 $\pm$ 0.02059	0.97256 $\pm$ 0.00795	0.98297 $\pm$ 0.00030	0.93856 $\pm$ 0.00329	1.33312 $\pm$ 0.00333
MolCLR-GCN	1.16344 $\pm$ 0.00748	0.79125 $\pm$ 0.00290	0.95325 $\pm$ 0.00245	0.84951 $\pm$ 0.00123	1.36284 $\pm$ 0.00189
TransM	0.74483 $\pm$ 0.00471	0.70292 $\pm$ 0.00370	0.77907 $\pm$ 0.00210	0.70334 $\pm$ 0.00463	0.95125 $\pm$ 0.00183
MPG	0.73944 $\pm$ 0.00589	0.66422 $\pm$ 0.00130	0.71036 $\pm$ 0.00387	0.61277 $\pm$ 0.00234	0.92861 $\pm$ 0.00246
KANO	1.05894 $\pm$ 0.00209	0.80277 $\pm$ 0.00076	0.81089 $\pm$ 0.00147	0.81646 $\pm$ 0.00115	1.36052 $\pm$ 0.00365

Prediction is evaluated by RMSE. For each method, the mean and standard deviation are reported for the evaluation metrics.

Supplementary Table 29. Activity cliff prediction (all molecules) – Part II

Method	CHEMBL2147 K_i	CHEMBL214 K_i	CHEMBL218 EC_{50}	CHEMBL219 K_i	CHEMBL228 K_i
qcGEM	0.61466 $\pm$ 0.00602	0.74509 $\pm$ 0.00297	0.76617 $\pm$ 0.00773	0.77383 $\pm$ 0.00587	0.72839 $\pm$ 0.00518
ECFP	0.74361 $\pm$ 0.01004	0.73303 $\pm$ 0.00376	0.87448 $\pm$ 0.00304	0.87184 $\pm$ 0.00115	0.81211 $\pm$ 0.00265
FCFP	0.67646 $\pm$ 0.00556	0.75507 $\pm$ 0.00211	0.83645 $\pm$ 0.00279	0.86186 $\pm$ 0.00328	0.85877 $\pm$ 0.00727
AvalonFP	0.71053 $\pm$ 0.00149	0.71376 $\pm$ 0.00198	0.77854 $\pm$ 0.00435	0.85434 $\pm$ 0.00345	0.84081 $\pm$ 0.00330
TTGEN	0.71707 $\pm$ 0.00587	0.82505 $\pm$ 0.00441	0.80314 $\pm$ 0.00515	0.84774 $\pm$ 0.00514	0.89600 $\pm$ 0.00113
APGEN	0.83712 $\pm$ 0.00650	0.84332 $\pm$ 0.00519	0.87665 $\pm$ 0.00471	0.88960 $\pm$ 0.00255	0.91511 $\pm$ 0.00590
RDKFP	0.68290 $\pm$ 0.00390	0.80530 $\pm$ 0.00284	0.73228 $\pm$ 0.00491	0.83213 $\pm$ 0.00530	0.87462 $\pm$ 0.00298
MACCS	0.91308 $\pm$ 0.00596	0.88889 $\pm$ 0.00321	0.83423 $\pm$ 0.00548	0.95768 $\pm$ 0.00233	0.92128 $\pm$ 0.00625
LayeredFP	0.81274 $\pm$ 0.00444	0.87506 $\pm$ 0.00377	0.82660 $\pm$ 0.00060	0.91473 $\pm$ 0.00092	0.85459 $\pm$ 0.00244
PatternFP	0.77215 $\pm$ 0.00529	0.87333 $\pm$ 0.00262	0.78006 $\pm$ 0.00183	0.87309 $\pm$ 0.00073	0.90526 $\pm$ 0.00264
Molformer	0.68498 $\pm$ 0.00330	0.73497 $\pm$ 0.00390	0.78227 $\pm$ 0.00240	0.80302 $\pm$ 0.00478	0.77089 $\pm$ 0.00428
GROVER-Base	0.70684 $\pm$ 0.01599	0.77441 $\pm$ 0.01800	0.85078 $\pm$ 0.03485	0.87993 $\pm$ 0.02392	0.85901 $\pm$ 0.02764
GROVER-Large	0.87952 $\pm$ 0.16928	0.86388 $\pm$ 0.07701	1.05974 $\pm$ 0.30444	0.96672 $\pm$ 0.04780	0.93170 $\pm$ 0.05234
MoleBERT	0.86644 $\pm$ 0.00282	0.85920 $\pm$ 0.00045	0.84152 $\pm$ 0.00187	0.87557 $\pm$ 0.00146	0.87971 $\pm$ 0.00138
UniMol-G	0.96757 $\pm$ 0.00276	0.95054 $\pm$ 0.00195	0.92690 $\pm$ 0.00035	1.02533 $\pm$ 0.00090	1.01779 $\pm$ 0.00433
UniMol-GA	0.91121 $\pm$ 0.00616	0.92661 $\pm$ 0.00213	0.92282 $\pm$ 0.00342	1.01596 $\pm$ 0.00100	0.98009 $\pm$ 0.00278
GCC	1.12656 $\pm$ 0.00505	1.00348 $\pm$ 0.00099	1.05079 $\pm$ 0.00318	1.00837 $\pm$ 0.00171	1.06528 $\pm$ 0.00110
GPT-GNN	1.02804 $\pm$ 0.02250	0.99815 $\pm$ 0.00410	0.96832 $\pm$ 0.00790	1.03291 $\pm$ 0.01194	1.00920 $\pm$ 0.00923
GraphCL	0.76042 $\pm$ 0.00560	0.89396 $\pm$ 0.00422	0.89781 $\pm$ 0.00313	0.92458 $\pm$ 0.00349	0.88172 $\pm$ 0.00305
InfoGraph	1.36522 $\pm$ 0.00453	1.07934 $\pm$ 0.00067	1.00164 $\pm$ 0.00076	1.07458 $\pm$ 0.00067	1.14917 $\pm$ 0.00107
JOAO	1.08643 $\pm$ 0.01596	1.04259 $\pm$ 0.00257	0.95532 $\pm$ 0.00561	1.02494 $\pm$ 0.00383	1.13554 $\pm$ 0.00482
JOAOv2	1.07009 $\pm$ 0.00584	1.04244 $\pm$ 0.00147	0.97235 $\pm$ 0.00074	1.00342 $\pm$ 0.00187	1.07634 $\pm$ 0.00451
PosPred	1.21646 $\pm$ 0.01184	1.06323 $\pm$ 0.00488	0.99805 $\pm$ 0.00295	0.99238 $\pm$ 0.00255	1.57255 $\pm$ 0.08395
3D-EMGP	1.89667 $\pm$ 0.00229	1.09949 $\pm$ 0.00073	1.03128 $\pm$ 0.00025	1.07015 $\pm$ 0.00015	1.23063 $\pm$ 0.05512
3D-InfoMax	0.82634 $\pm$ 0.00937	0.90783 $\pm$ 0.00230	0.86426 $\pm$ 0.00290	0.93551 $\pm$ 0.00233	0.93310 $\pm$ 0.00372
AttrMask	1.28845 $\pm$ 0.00120	1.05570 $\pm$ 0.00073	1.01061 $\pm$ 0.00065	1.06427 $\pm$ 0.00046	1.10969 $\pm$ 0.00082
EdgePred	0.90890 $\pm$ 0.00272	0.93025 $\pm$ 0.00064	0.83842 $\pm$ 0.00144	0.94226 $\pm$ 0.00136	1.00639 $\pm$ 0.00221
GraphMVP	0.93749 $\pm$ 0.00451	0.94492 $\pm$ 0.00188	0.87613 $\pm$ 0.00189	0.96934 $\pm$ 0.00156	0.95057 $\pm$ 0.00077
MolCLR-GIN	1.23394 $\pm$ 0.00648	1.09580 $\pm$ 0.00030	1.02650 $\pm$ 0.00028	1.06918 $\pm$ 0.00017	1.17211 $\pm$ 0.00063
MolCLR-GCN	1.17534 $\pm$ 0.00233	1.08253 $\pm$ 0.00330	1.03530 $\pm$ 0.00089	1.06330 $\pm$ 0.00031	1.16692 $\pm$ 0.00054
TransM	0.62929 $\pm$ 0.00335	0.77439 $\pm$ 0.00366	0.71587 $\pm$ 0.00330	0.81164 $\pm$ 0.00454	0.78561 $\pm$ 0.00290
MPG	0.69768 $\pm$ 0.00769	0.77055 $\pm$ 0.00295	0.83245 $\pm$ 0.00185	0.87911 $\pm$ 0.00180	0.80515 $\pm$ 0.00136
KANO	1.09928 $\pm$ 0.00139	1.04304 $\pm$ 0.00156	0.98547 $\pm$ 0.00133	1.03895 $\pm$ 0.00142	1.10604 $\pm$ 0.00180

Prediction is evaluated by RMSE. For each method, the mean and standard deviation are reported for the evaluation metrics.

Supplementary Table 30. Activity cliff prediction (all molecules) – Part III

**Method**	**CHEMBL231** $\mathbf{K}_i$	**CHEMBL233** $\mathbf{K}_i$	**CHEMBL234** $\mathbf{K}_i$	**CHEMBL235** $\mathbf{EC}_{50}$	**CHEMBL236** $\mathbf{K}_i$
qcGEM	0.65440 ± 0.00873	0.87174 ± 0.00390	0.72563 ± 0.00194	0.68084 ± 0.00479	0.76550 ± 0.00297
ECFP	0.93158 ± 0.00910	0.89385 ± 0.00274	0.71508 ± 0.00498	0.76087 ± 0.00293	0.75841 ± 0.00330
FCFP	0.82003 ± 0.00521	0.93504 ± 0.00593	0.74603 ± 0.00288	0.76455 ± 0.00378	0.81321 ± 0.00325
AvalonFP	0.91507 ± 0.00846	0.88096 ± 0.00247	0.72732 ± 0.00328	0.75419 ± 0.00254	0.80195 ± 0.00111
TTGEN	0.81180 ± 0.00506	0.91007 ± 0.00357	0.73995 ± 0.00243	0.79620 ± 0.00311	0.84120 ± 0.00056
APGEN	0.81580 ± 0.00503	0.99313 ± 0.00323	0.80591 ± 0.00497	0.78003 ± 0.00914	0.92188 ± 0.00399
RDKFP	0.87746 ± 0.00370	0.97702 ± 0.00186	0.78239 ± 0.00195	0.72297 ± 0.00213	0.91632 ± 0.00305
MACCS	0.95576 ± 0.00426	1.06502 ± 0.00242	0.85781 ± 0.00755	0.87088 ± 0.00597	1.00499 ± 0.00630
LayeredFP	0.85043 ± 0.00513	1.03202 ± 0.00060	0.83281 ± 0.00268	0.80461 ± 0.00489	0.95897 ± 0.00367
PatternFP	0.83732 ± 0.00825	1.02169 ± 0.00132	0.86660 ± 0.00034	0.80034 ± 0.00247	0.93317 ± 0.00477
Molformer	0.77181 ± 0.00571	0.88199 ± 0.00155	0.71222 ± 0.00208	0.65417 ± 0.00383	0.81193 ± 0.00299
GROVER-Base	0.81907 ± 0.03944	0.91444 ± 0.02443	0.76671 ± 0.01195	0.82107 ± 0.03139	0.84554 ± 0.02096
GROVER-Large	1.11323 ± 0.38755	0.92147 ± 0.02575	0.77556 ± 0.00738	0.84075 ± 0.05237	0.90521 ± 0.08132
MoleBERT	0.85923 ± 0.00287	1.01749 ± 0.00076	0.84984 ± 0.00133	0.76103 ± 0.00084	0.89412 ± 0.00075
UniMol-G	0.94870 ± 0.00337	1.09289 ± 0.00160	0.92002 ± 0.00098	0.93448 ± 0.00225	1.05310 ± 0.00301
UniMol-GA	0.89326 ± 0.00384	1.06118 ± 0.00178	0.90049 ± 0.00131	0.90923 ± 0.00587	1.03121 ± 0.00243
GCC	1.01886 ± 0.00539	1.13241 ± 0.00184	0.95744 ± 0.00058	0.98453 ± 0.00339	1.11900 ± 0.00323
GPT-GNN	0.94968 ± 0.01025	1.06714 ± 0.01256	0.92257 ± 0.00273	0.94303 ± 0.00846	1.06309 ± 0.01109
GraphCL	0.88804 ± 0.00518	1.04440 ± 0.00207	0.81551 ± 0.00178	0.85450 ± 0.00270	0.95089 ± 0.00335
InfoGraph	1.14598 ± 0.00276	1.21415 ± 0.00128	1.00855 ± 0.00330	1.04762 ± 0.00050	1.27377 ± 0.00314
JOAO	1.09933 ± 0.00487	1.21209 ± 0.02237	0.95928 ± 0.00213	1.01021 ± 0.00176	1.23949 ± 0.00573
JOAOv2	1.10815 ± 0.00899	1.18313 ± 0.00314	0.92708 ± 0.00096	1.00419 ± 0.00161	1.19091 ± 0.00344
PosPred	1.18139 ± 0.01046	1.20692 ± 0.01695	0.96177 ± 0.00515	0.96178 ± 0.00455	1.25145 ± 0.02897
3D-EMGP	1.30526 ± 0.00202	1.28675 ± 0.00156	1.10141 ± 0.00008	1.08099 ± 0.00222	1.32813 ± 0.00072
3D-InfoMax	0.86074 ± 0.00381	1.01287 ± 0.00340	0.85254 ± 0.00227	0.83231 ± 0.00520	0.92660 ± 0.00428
AttrMask	1.25491 ± 0.00089	1.15777 ± 0.00028	1.02351 ± 0.00052	0.99922 ± 0.00086	1.20795 ± 0.00127
EdgePred	0.84213 ± 0.00223	0.98125 ± 0.00045	0.87958 ± 0.00145	0.83713 ± 0.00068	0.93892 ± 0.00053
GraphMVP	0.95253 ± 0.00288	1.09197 ± 0.00189	0.94544 ± 0.00139	0.87687 ± 0.00105	0.98539 ± 0.00124
MolCLR-GIN	1.32172 ± 0.00162	1.27205 ± 0.00080	1.09954 ± 0.00023	1.06086 ± 0.00070	1.26766 ± 0.00643
MolCLR-GCN	1.33118 ± 0.00070	1.19390 ± 0.00096	1.02998 ± 0.00116	1.04621 ± 0.00300	1.26128 ± 0.00144
TransM	0.79262 ± 0.00565	0.89184 ± 0.00345	0.74281 ± 0.00225	0.74342 ± 0.00183	0.87224 ± 0.00385
MPG	0.79708 ± 0.00278	0.93468 ± 0.00234	0.76332 ± 0.00187	0.74028 ± 0.00121	0.83339 ± 0.00248
KANO	1.09700 ± 0.00395	1.13296 ± 0.00035	0.97776 ± 0.00039	0.97575 ± 0.00085	1.06665 ± 0.00114

Prediction is evaluated by RMSE. For each method, the mean and standard deviation are reported for the evaluation metrics.

Supplementary Table 31. Activity cliff prediction (all molecules) – Part IV

Method	CHEMBL237 EC_{50}	CHEMBL237 K_i	CHEMBL238 K_i	CHEMBL239 EC_{50}	CHEMBL244 K_i
qcGEM	0.84118 ± 0.00660	0.73866 ± 0.00247	0.70150 ± 0.00748	0.74247 ± 0.00383	0.82510 ± 0.00596
ECFP	0.82259 ± 0.00636	0.79107 ± 0.00513	0.80513 ± 0.00400	0.85001 ± 0.00278	0.82708 ± 0.00506
FCFP	0.88028 ± 0.00263	0.78796 ± 0.00361	0.85280 ± 0.00391	0.78912 ± 0.00258	0.87704 ± 0.00398
AvalonFP	0.89985 ± 0.00466	0.81834 ± 0.00299	0.76474 ± 0.00493	0.76359 ± 0.00531	0.84888 ± 0.00603
TTGEN	0.97405 ± 0.00782	0.79851 ± 0.00187	0.76175 ± 0.00431	0.86440 ± 0.00321	0.99417 ± 0.00494
APGEN	0.96497 ± 0.00461	0.88436 ± 0.00706	0.81106 ± 0.00915	0.90922 ± 0.00208	1.10089 ± 0.00635
RDKFP	1.00436 ± 0.00221	0.95430 ± 0.00234	0.78897 ± 0.00972	0.82779 ± 0.00361	0.85908 ± 0.00606
MACCS	1.02421 ± 0.00674	0.97355 ± 0.00637	0.81729 ± 0.00247	0.88597 ± 0.00811	1.00487 ± 0.01248
LayeredFP	1.08796 ± 0.00271	0.90564 ± 0.00076	0.78298 ± 0.00617	0.85934 ± 0.00260	1.02208 ± 0.00483
PatternFP	1.11031 ± 0.00264	0.95846 ± 0.00024	0.81404 ± 0.00189	0.85246 ± 0.00318	1.01489 ± 0.00864
Molformer	0.83803 ± 0.00751	0.79754 ± 0.00374	0.73931 ± 0.00502	0.81765 ± 0.00109	0.83784 ± 0.00139
GROVER-Base	0.90496 ± 0.03623	0.82821 ± 0.02097	0.81301 ± 0.02020	0.91849 ± 0.02926	0.90087 ± 0.01185
GROVER-Large	1.19650 ± 0.22738	0.90472 ± 0.08559	1.15105 ± 0.39997	0.98785 ± 0.08787	0.90462 ± 0.02620
MoleBERT	0.98883 ± 0.00172	0.90912 ± 0.00135	0.75586 ± 0.00197	0.85056 ± 0.00104	0.97762 ± 0.00140
UniMol-G	1.23674 ± 0.00169	1.04999 ± 0.00391	0.96532 ± 0.00291	0.92972 ± 0.00075	1.12679 ± 0.00197
UniMol-GA	1.18759 ± 0.00347	1.00381 ± 0.00517	0.92726 ± 0.00204	0.91083 ± 0.00139	1.07019 ± 0.00355
GCC	1.23575 ± 0.00125	1.09359 ± 0.00169	0.99221 ± 0.00354	0.96781 ± 0.00095	1.26780 ± 0.00331
GPT-GNN	1.23772 ± 0.00513	0.99867 ± 0.00669	1.03210 ± 0.00658	0.99128 ± 0.00630	1.24413 ± 0.02276
GraphCL	1.03741 ± 0.00478	0.93526 ± 0.00214	0.84711 ± 0.00269	0.87626 ± 0.00332	1.01443 ± 0.00324
InfoGraph	1.31421 ± 0.00346	1.17125 ± 0.00141	1.16110 ± 0.00205	1.02350 ± 0.00126	1.37926 ± 0.00242
JOAO	1.31023 ± 0.00663	1.19453 ± 0.01122	1.08481 ± 0.00367	0.98579 ± 0.00213	1.33049 ± 0.00343
JOAOv2	1.23608 ± 0.00490	1.15050 ± 0.00443	1.01912 ± 0.00448	1.01104 ± 0.00091	1.26212 ± 0.00268
PosPred	1.45535 ± 0.02002	1.16914 ± 0.01138	1.14652 ± 0.02257	1.02944 ± 0.01849	1.28549 ± 0.00098
3D-EMGP	1.42974 ± 0.00789	1.31752 ± 0.00085	1.15810 ± 0.00042	1.03897 ± 0.00114	1.56807 ± 0.00085
3D-InfoMax	1.05207 ± 0.00412	0.89662 ± 0.00249	0.91265 ± 0.00200	0.90515 ± 0.00315	1.01652 ± 0.00329
AttrMask	1.30421 ± 0.00159	1.13603 ± 0.00049	1.13082 ± 0.00040	1.01898 ± 0.00077	1.40814 ± 0.00048
EdgePred	1.01786 ± 0.00140	0.91859 ± 0.00200	0.86095 ± 0.00296	0.95299 ± 0.00097	1.07041 ± 0.00095
GraphMVP	1.15585 ± 0.00200	1.07566 ± 0.00116	0.87245 ± 0.00235	0.93455 ± 0.00091	1.12634 ± 0.00490
MolCLR-GIN	1.44413 ± 0.00054	1.25829 ± 0.00416	1.14814 ± 0.00373	1.08056 ± 0.00027	1.34215 ± 0.01838
MolCLR-GCN	1.34036 ± 0.00859	1.12373 ± 0.00118	1.15603 ± 0.00313	1.07925 ± 0.00245	1.35700 ± 0.00169
TransM	0.86623 ± 0.00408	0.82442 ± 0.00160	0.72420 ± 0.00455	0.82048 ± 0.00623	0.90828 ± 0.00284
MPG	0.90884 ± 0.00386	0.80648 ± 0.00345	0.75168 ± 0.00371	0.75384 ± 0.00142	0.86321 ± 0.00252
KANO	1.25919 ± 0.00318	1.11950 ± 0.00049	0.96917 ± 0.00341	0.97049 ± 0.00182	1.27645 ± 0.00074

Prediction is evaluated by RMSE. For each method, the mean and standard deviation are reported for the evaluation metrics.

Supplementary Table 32. Activity cliff prediction (all molecules) – Part V

Method	CHEMBL262 K_i	CHEMBL264 K_i	CHEMBL2835 K_i	CHEMBL287 K_i	CHEMBL2971 K_i
qcGEM	0.83561 $\pm$ 0.00693	0.66724 $\pm$ 0.00250	0.37588 $\pm$ 0.00235	0.71752 $\pm$ 0.00986	0.63797 $\pm$ 0.01255
ECFP	0.86035 $\pm$ 0.00580	0.73272 $\pm$ 0.00350	0.40712 $\pm$ 0.00335	0.73287 $\pm$ 0.00702	0.71883 $\pm$ 0.00549
FCFP	0.78720 $\pm$ 0.00381	0.72785 $\pm$ 0.00211	0.37844 $\pm$ 0.00205	0.75285 $\pm$ 0.00412	0.75947 $\pm$ 0.00629
AvalonFP	0.80481 $\pm$ 0.00068	0.72486 $\pm$ 0.00351	0.43081 $\pm$ 0.00251	0.82536 $\pm$ 0.00671	0.63631 $\pm$ 0.00238
TTGEN	0.89269 $\pm$ 0.00333	0.77332 $\pm$ 0.00198	0.42959 $\pm$ 0.00353	0.76819 $\pm$ 0.00296	0.67478 $\pm$ 0.00673
APGEN	0.83197 $\pm$ 0.00362	0.76271 $\pm$ 0.00623	0.42976 $\pm$ 0.00221	0.79364 $\pm$ 0.00508	0.74055 $\pm$ 0.00513
RDKFP	0.81911 $\pm$ 0.00510	0.78569 $\pm$ 0.00387	0.38239 $\pm$ 0.00121	0.89183 $\pm$ 0.00280	0.68741 $\pm$ 0.00463
MACCS	0.92171 $\pm$ 0.00279	0.86649 $\pm$ 0.00777	0.45570 $\pm$ 0.00334	0.84284 $\pm$ 0.00462	0.72995 $\pm$ 0.00219
LayeredFP	0.84229 $\pm$ 0.00343	0.83684 $\pm$ 0.00465	0.44052 $\pm$ 0.00281	0.79868 $\pm$ 0.00366	0.77930 $\pm$ 0.00128
PatternFP	0.86082 $\pm$ 0.00475	0.80802 $\pm$ 0.00388	0.38199 $\pm$ 0.00172	0.78745 $\pm$ 0.00220	0.69226 $\pm$ 0.00366
Molformer	0.75650 $\pm$ 0.00900	0.67308 $\pm$ 0.00383	0.40334 $\pm$ 0.00230	0.69545 $\pm$ 0.00328	0.65083 $\pm$ 0.00604
GROVER-Base	0.85394 $\pm$ 0.02830	0.71055 $\pm$ 0.01160	0.38651 $\pm$ 0.00144	0.71275 $\pm$ 0.01716	0.66520 $\pm$ 0.01026
GROVER-Large	1.18886 $\pm$ 0.42976	0.73734 $\pm$ 0.01400	0.46278 $\pm$ 0.11488	0.83737 $\pm$ 0.09905	0.81706 $\pm$ 0.15570
MoleBERT	0.81559 $\pm$ 0.00109	0.82774 $\pm$ 0.00075	0.42258 $\pm$ 0.00176	0.72707 $\pm$ 0.00083	0.72979 $\pm$ 0.00299
UniMol-G	0.95057 $\pm$ 0.00064	0.88992 $\pm$ 0.00192	0.48653 $\pm$ 0.00253	0.81510 $\pm$ 0.00201	0.78272 $\pm$ 0.00227
UniMol-GA	0.93193 $\pm$ 0.00159	0.86031 $\pm$ 0.00231	0.48414 $\pm$ 0.00225	0.79031 $\pm$ 0.00184	0.75903 $\pm$ 0.00366
GCC	1.07609 $\pm$ 0.00379	0.96670 $\pm$ 0.00142	0.58035 $\pm$ 0.00362	0.82829 $\pm$ 0.00440	0.96508 $\pm$ 0.00781
GPT-GNN	0.90974 $\pm$ 0.01245	0.88114 $\pm$ 0.00337	0.47721 $\pm$ 0.00603	0.78402 $\pm$ 0.00661	0.85629 $\pm$ 0.00874
GraphCL	0.92422 $\pm$ 0.00324	0.77549 $\pm$ 0.00253	0.46845 $\pm$ 0.00443	0.81982 $\pm$ 0.00415	0.76440 $\pm$ 0.00684
InfoGraph	0.99478 $\pm$ 0.00426	1.02329 $\pm$ 0.00172	0.53743 $\pm$ 0.00285	0.89771 $\pm$ 0.00268	0.90513 $\pm$ 0.00337
JOAO	1.01182 $\pm$ 0.00524	0.98122 $\pm$ 0.00068	0.46146 $\pm$ 0.00215	0.87946 $\pm$ 0.00503	0.79513 $\pm$ 0.00557
JOAOv2	1.01717 $\pm$ 0.00507	0.97053 $\pm$ 0.00369	0.43312 $\pm$ 0.00344	0.80372 $\pm$ 0.00456	0.81133 $\pm$ 0.00398
PosPred	0.99698 $\pm$ 0.00185	1.00186 $\pm$ 0.00337	0.51900 $\pm$ 0.02838	0.83259 $\pm$ 0.00501	0.84912 $\pm$ 0.01012
3D-EMGP	1.12148 $\pm$ 0.00596	1.06637 $\pm$ 0.00069	0.83013 $\pm$ 0.01970	1.62800 $\pm$ 0.13060	1.19840 $\pm$ 0.00283
3D-InfoMax	0.89698 $\pm$ 0.00415	0.80796 $\pm$ 0.00248	0.40006 $\pm$ 0.00302	0.75378 $\pm$ 0.00238	0.75900 $\pm$ 0.00378
AttrMask	1.10127 $\pm$ 0.00185	1.03171 $\pm$ 0.00146	0.58606 $\pm$ 0.00047	0.87300 $\pm$ 0.00085	0.92657 $\pm$ 0.00190
EdgePred	0.90044 $\pm$ 0.00189	0.84142 $\pm$ 0.00066	0.46324 $\pm$ 0.00181	0.79754 $\pm$ 0.00365	0.88733 $\pm$ 0.00297
GraphMVP	0.93333 $\pm$ 0.00381	0.89160 $\pm$ 0.00205	0.47823 $\pm$ 0.00162	0.74506 $\pm$ 0.00156	0.77325 $\pm$ 0.00194
MolCLR-GIN	1.11441 $\pm$ 0.00104	1.06141 $\pm$ 0.00021	0.86888 $\pm$ 0.00387	0.96659 $\pm$ 0.00042	0.93877 $\pm$ 0.00206
MolCLR-GCN	1.08935 $\pm$ 0.00098	1.06182 $\pm$ 0.00124	0.85232 $\pm$ 0.00543	0.88144 $\pm$ 0.00565	1.05347 $\pm$ 0.00264
TransM	0.78487 $\pm$ 0.00460	0.71896 $\pm$ 0.00274	0.41310 $\pm$ 0.00225	0.69636 $\pm$ 0.00252	0.69113 $\pm$ 0.00224
MPG	0.78622 $\pm$ 0.00306	0.71296 $\pm$ 0.00296	0.39649 $\pm$ 0.00273	0.67930 $\pm$ 0.00196	0.65249 $\pm$ 0.00159
KANO	1.06832 $\pm$ 0.00194	1.02020 $\pm$ 0.00388	0.86282 $\pm$ 0.00016	0.79092 $\pm$ 0.00196	0.92437 $\pm$ 0.00162

Prediction is evaluated by RMSE. For each method, the mean and standard deviation are reported for the evaluation metrics.

Supplementary Table 33. Activity cliff prediction (all molecules) – Part VI

**Method**	**CHEMBL3979** $\mathbf{EC_{50}}$	**CHEMBL4005** $\mathbf{K}_i$	**CHEMBL4203** $\mathbf{K}_i$	**CHEMBL4616** $\mathbf{EC_{50}}$	**CHEMBL4792** $\mathbf{K}_i$
qcGEM	0.61750 ± 0.01103	0.66537 ± 0.00283	0.91154 ± 0.00258	0.70984 ± 0.00385	0.76991 ± 0.00268
ECFP	0.61958 ± 0.00550	0.70054 ± 0.00476	0.98782 ± 0.00868	0.67271 ± 0.00760	0.88131 ± 0.00266
FCFP	0.66562 ± 0.00832	0.69028 ± 0.00408	0.97847 ± 0.00680	0.72468 ± 0.00570	0.80995 ± 0.00480
AvalonFP	0.67116 ± 0.00985	0.71417 ± 0.00346	0.95362 ± 0.00592	0.74354 ± 0.00136	0.82965 ± 0.00355
TTGEN	0.66008 ± 0.01764	0.70013 ± 0.00317	1.06014 ± 0.00855	0.79175 ± 0.00303	0.84797 ± 0.00274
APGEN	0.74385 ± 0.00988	0.74928 ± 0.00101	0.95806 ± 0.00624	0.76263 ± 0.00403	0.83734 ± 0.00410
RDKFP	0.65923 ± 0.01219	0.66379 ± 0.00135	1.02744 ± 0.00405	0.75696 ± 0.00141	0.89692 ± 0.00182
MACCS	0.71890 ± 0.01629	0.73376 ± 0.00186	1.02464 ± 0.00296	0.76200 ± 0.00134	0.97723 ± 0.00464
LayeredFP	0.69644 ± 0.00260	0.75588 ± 0.00286	1.01443 ± 0.00254	0.78587 ± 0.00124	0.99435 ± 0.00247
PatternFP	0.71659 ± 0.00456	0.70192 ± 0.00144	0.98467 ± 0.00154	0.74636 ± 0.00168	0.91965 ± 0.00425
Molformer	0.64612 ± 0.00559	0.72152 ± 0.00183	0.91917 ± 0.00372	0.68603 ± 0.00587	0.71412 ± 0.00445
GROVER-Base	0.82505 ± 0.02265	0.73605 ± 0.02139	0.97250 ± 0.00543	0.76898 ± 0.00459	0.88750 ± 0.02796
GROVER-Large	1.07350 ± 0.30200	0.87205 ± 0.16790	1.35627 ± 0.54014	0.87174 ± 0.11836	0.98112 ± 0.08643
MoleBERT	0.70976 ± 0.00864	0.73042 ± 0.00124	0.81864 ± 0.00148	0.78853 ± 0.00153	0.84837 ± 0.00311
UniMol-G	0.92668 ± 0.00599	0.82215 ± 0.00067	0.95735 ± 0.00202	0.82827 ± 0.00216	1.00311 ± 0.00224
UniMol-GA	0.89927 ± 0.00634	0.79434 ± 0.00241	0.94477 ± 0.00286	0.81734 ± 0.00121	0.96821 ± 0.00369
GCC	1.00188 ± 0.00745	0.89009 ± 0.00240	1.07396 ± 0.00326	0.87125 ± 0.00174	1.06610 ± 0.00185
GPT-GNN	1.10928 ± 0.00790	0.83516 ± 0.01420	1.09314 ± 0.00867	0.94039 ± 0.00477	1.02330 ± 0.00616
GraphCL	0.71387 ± 0.00971	0.75217 ± 0.00255	1.01713 ± 0.00361	0.81149 ± 0.00238	0.83494 ± 0.00383
InfoGraph	1.02534 ± 0.00313	0.94612 ± 0.00356	1.03964 ± 0.00208	0.91551 ± 0.00236	1.15726 ± 0.00135
JOAO	1.05432 ± 0.00429	0.89808 ± 0.00397	1.05487 ± 0.00211	0.90987 ± 0.00617	1.12457 ± 0.00195
JOAOv2	1.04754 ± 0.00322	0.87862 ± 0.00563	1.04977 ± 0.00139	0.89652 ± 0.00186	1.05364 ± 0.00270
PosPred	1.02401 ± 0.01119	0.93496 ± 0.00761	1.03782 ± 0.00217	0.92094 ± 0.00494	1.05715 ± 0.00484
3D-EMGP	1.11505 ± 0.00688	1.07238 ± 0.00175	1.11360 ± 0.01554	0.92705 ± 0.00091	2.08299 ± 0.20476
3D-InfoMax	0.78771 ± 0.00792	0.76935 ± 0.00369	1.01150 ± 0.00240	0.81179 ± 0.00263	0.90973 ± 0.00298
AttrMask	1.08250 ± 0.00214	0.94196 ± 0.00282	1.07546 ± 0.00044	0.92360 ± 0.00049	1.06421 ± 0.00087
EdgePred	0.77567 ± 0.00774	0.79311 ± 0.00131	0.95855 ± 0.00219	0.83237 ± 0.00313	0.86506 ± 0.00243
GraphMVP	0.80773 ± 0.01101	0.77758 ± 0.00216	0.97786 ± 0.00087	0.82065 ± 0.00271	1.00366 ± 0.00118
MolCLR-GIN	1.07126 ± 0.00248	1.06040 ± 0.00109	1.07055 ± 0.00051	0.92857 ± 0.00047	1.18004 ± 0.00129
MolCLR-GCN	1.05715 ± 0.00938	1.05724 ± 0.00316	1.07295 ± 0.00036	0.93496 ± 0.00070	1.14787 ± 0.00196
TransM	0.66290 ± 0.00375	0.71083 ± 0.00208	0.95361 ± 0.00161	0.74541 ± 0.00222	0.79952 ± 0.00323
MPG	0.65448 ± 0.00682	0.71177 ± 0.00291	0.90298 ± 0.00244	0.75390 ± 0.00282	0.79482 ± 0.00249
KANO	0.88936 ± 0.00376	1.02902 ± 0.00516	1.03991 ± 0.00069	0.90715 ± 0.00355	1.06745 ± 0.00184

Prediction is evaluated by RMSE. For each method, the mean and standard deviation are reported for the evaluation metrics.

Supplementary Table 34. Activity cliff prediction (cliff molecules) – Part I

Method	CHEMBL1862 K_i	CHEMBL1871 K_i	CHEMBL2034 K_i	CHEMBL2047 EC_{50}	CHEMBL204 K_i
qcGEM	0.78454 $\pm$ 0.01263	0.85137 $\pm$ 0.01929	0.94914 $\pm$ 0.00888	0.65620 $\pm$ 0.01270	0.93956 $\pm$ 0.00975
ECFP	0.83430 $\pm$ 0.02353	0.88172 $\pm$ 0.01064	0.87067 $\pm$ 0.00232	0.72209 $\pm$ 0.00497	0.97275 $\pm$ 0.01004
FCFP	0.80484 $\pm$ 0.00792	0.88891 $\pm$ 0.01031	0.85456 $\pm$ 0.01174	0.74101 $\pm$ 0.00778	1.01742 $\pm$ 0.00755
AvalonFP	0.82919 $\pm$ 0.01076	1.06238 $\pm$ 0.01803	0.86549 $\pm$ 0.01113	0.79843 $\pm$ 0.00471	1.02395 $\pm$ 0.00368
TTGEN	0.87787 $\pm$ 0.00282	0.92752 $\pm$ 0.01338	1.01772 $\pm$ 0.01132	0.74474 $\pm$ 0.00626	1.10662 $\pm$ 0.00378
APGEN	0.80238 $\pm$ 0.01655	0.90565 $\pm$ 0.01498	0.96859 $\pm$ 0.01163	0.68805 $\pm$ 0.00987	1.21172 $\pm$ 0.00751
RDKFP	0.83071 $\pm$ 0.00374	0.95258 $\pm$ 0.00933	0.91186 $\pm$ 0.00364	0.75374 $\pm$ 0.01983	1.05361 $\pm$ 0.00675
MACCS	0.89946 $\pm$ 0.01249	0.96972 $\pm$ 0.00392	0.85935 $\pm$ 0.00189	0.72246 $\pm$ 0.01303	1.20470 $\pm$ 0.01602
LayeredFP	0.87613 $\pm$ 0.00749	0.97204 $\pm$ 0.01214	0.93397 $\pm$ 0.00637	0.80021 $\pm$ 0.00818	1.18767 $\pm$ 0.00459
PatternFP	0.83071 $\pm$ 0.00220	0.98523 $\pm$ 0.00408	0.96983 $\pm$ 0.00739	0.70298 $\pm$ 0.00738	1.20718 $\pm$ 0.00608
Molformer	0.80902 $\pm$ 0.00857	0.94843 $\pm$ 0.02268	0.92575 $\pm$ 0.00403	0.70045 $\pm$ 0.01398	0.97356 $\pm$ 0.00810
GROVER-Base	0.83272 $\pm$ 0.01319	0.93154 $\pm$ 0.03551	0.89952 $\pm$ 0.00965	0.64642 $\pm$ 0.01375	1.04710 $\pm$ 0.03864
GROVER-Large	0.97480 $\pm$ 0.13113	1.11486 $\pm$ 0.17533	0.96808 $\pm$ 0.06977	1.11416 $\pm$ 0.63494	1.12981 $\pm$ 0.07407
MoleBERT	0.90203 $\pm$ 0.00228	0.97596 $\pm$ 0.00400	0.94670 $\pm$ 0.00599	0.62355 $\pm$ 0.00336	1.19726 $\pm$ 0.00269
UniMol-G	0.95152 $\pm$ 0.01535	0.99902 $\pm$ 0.00643	0.86433 $\pm$ 0.01112	0.80484 $\pm$ 0.02456	1.36996 $\pm$ 0.00797
UniMol-GA	0.94235 $\pm$ 0.01115	1.03305 $\pm$ 0.00793	0.84206 $\pm$ 0.01232	0.79584 $\pm$ 0.01571	1.30928 $\pm$ 0.00659
GCC	0.94809 $\pm$ 0.01357	1.06086 $\pm$ 0.00630	0.93303 $\pm$ 0.00328	0.78554 $\pm$ 0.00499	1.40323 $\pm$ 0.00964
GPT-GNN	1.02696 $\pm$ 0.03253	1.08751 $\pm$ 0.00689	0.91480 $\pm$ 0.01686	0.77862 $\pm$ 0.02456	1.42885 $\pm$ 0.01572
GraphCL	0.81438 $\pm$ 0.00297	0.88224 $\pm$ 0.00467	0.83573 $\pm$ 0.00858	0.67451 $\pm$ 0.01276	1.21286 $\pm$ 0.00541
InfoGraph	1.31784 $\pm$ 0.02375	1.18629 $\pm$ 0.00951	0.89739 $\pm$ 0.00490	0.85832 $\pm$ 0.01668	1.55463 $\pm$ 0.01386
JOAO	1.02844 $\pm$ 0.03776	1.28621 $\pm$ 0.02264	0.91150 $\pm$ 0.00516	0.81132 $\pm$ 0.01915	1.44554 $\pm$ 0.01620
JOAOv2	0.95052 $\pm$ 0.02834	1.19775 $\pm$ 0.01388	0.91808 $\pm$ 0.00181	0.84342 $\pm$ 0.01471	1.47952 $\pm$ 0.00658
PosPred	1.07338 $\pm$ 0.02034	1.23686 $\pm$ 0.01996	0.92779 $\pm$ 0.00483	0.94871 $\pm$ 0.05651	1.48184 $\pm$ 0.03036
3D-EMGP	1.45138 $\pm$ 0.00740	1.18469 $\pm$ 0.00148	0.93750 $\pm$ 0.00039	0.87121 $\pm$ 0.00512	1.66990 $\pm$ 0.00964
3D-InfoMax	0.89331 $\pm$ 0.01146	0.93996 $\pm$ 0.01135	0.81695 $\pm$ 0.00480	0.77179 $\pm$ 0.01292	1.19371 $\pm$ 0.01258
AttrMask	1.05330 $\pm$ 0.00536	1.06090 $\pm$ 0.00470	0.90959 $\pm$ 0.00279	0.81373 $\pm$ 0.00659	1.59042 $\pm$ 0.01045
EdgePred	0.93752 $\pm$ 0.00436	1.11094 $\pm$ 0.00198	0.85427 $\pm$ 0.00810	0.78540 $\pm$ 0.00712	1.28451 $\pm$ 0.00668
GraphMVP	0.98704 $\pm$ 0.00521	1.00193 $\pm$ 0.00325	0.91814 $\pm$ 0.00406	0.73013 $\pm$ 0.00534	1.33209 $\pm$ 0.00430
MolCLR-GIN	1.09368 $\pm$ 0.03827	1.18675 $\pm$ 0.01306	0.93669 $\pm$ 0.00048	0.86301 $\pm$ 0.01034	1.51943 $\pm$ 0.02022
MolCLR-GCN	1.17818 $\pm$ 0.02330	1.06788 $\pm$ 0.01092	0.87663 $\pm$ 0.00292	0.80343 $\pm$ 0.00445	1.51585 $\pm$ 0.01760
TransM	0.82974 $\pm$ 0.00999	0.95840 $\pm$ 0.01240	0.90644 $\pm$ 0.01125	0.71915 $\pm$ 0.00735	1.13930 $\pm$ 0.00751
MPG	0.80550 $\pm$ 0.00861	0.95879 $\pm$ 0.01284	0.88822 $\pm$ 0.00894	0.62959 $\pm$ 0.00429	1.06979 $\pm$ 0.00221
KANO	1.03620 $\pm$ 0.00416	1.08738 $\pm$ 0.00741	0.86137 $\pm$ 0.00197	0.74080 $\pm$ 0.00437	1.49179 $\pm$ 0.00572

Prediction is evaluated by RMSE. For each method, the mean and standard deviation are reported for the evaluation metrics.

Supplementary Table 35. Activity cliff prediction (cliff molecules) – Part II

Method	CHEMBL2147 K_i	CHEMBL214 K_i	CHEMBL218 EC_{50}	CHEMBL219 K_i	CHEMBL228 K_i
qcGEM	0.66654 ± 0.00263	0.89286 ± 0.00218	0.82349 ± 0.00459	0.81123 ± 0.00572	0.77128 ± 0.00907
ECFP	0.77127 ± 0.01352	0.78791 ± 0.00182	0.87531 ± 0.00342	0.89344 ± 0.00508	0.87223 ± 0.00404
FCFP	0.69259 ± 0.00913	0.84218 ± 0.00450	0.78929 ± 0.00281	0.89758 ± 0.00691	0.88796 ± 0.00543
AvalonFP	0.78322 ± 0.00477	0.79586 ± 0.00335	0.80560 ± 0.01006	0.86736 ± 0.00393	0.83551 ± 0.00389
TTGEN	0.80440 ± 0.00726	0.87387 ± 0.00265	0.81389 ± 0.00876	0.85717 ± 0.00689	0.92002 ± 0.00399
APGEN	0.78560 ± 0.00470	0.93383 ± 0.00681	0.85826 ± 0.00888	0.88448 ± 0.00514	0.92592 ± 0.00417
RDKFP	0.73152 ± 0.00611	0.89728 ± 0.00248	0.75743 ± 0.01331	0.90247 ± 0.00437	0.89773 ± 0.00495
MACCS	0.92693 ± 0.00787	0.96277 ± 0.00270	0.86529 ± 0.00739	0.94422 ± 0.00118	0.99194 ± 0.00600
LayeredFP	0.80055 ± 0.00568	0.95704 ± 0.00666	0.88277 ± 0.00322	0.92044 ± 0.00234	0.88886 ± 0.00322
PatternFP	0.85054 ± 0.00448	0.93463 ± 0.00559	0.87933 ± 0.00378	0.88703 ± 0.00175	0.97826 ± 0.00278
Molformer	0.79114 ± 0.00522	0.85188 ± 0.00245	0.80029 ± 0.00916	0.85330 ± 0.00632	0.83775 ± 0.00808
GROVER-Base	0.78126 ± 0.01088	0.88629 ± 0.01320	0.84542 ± 0.03335	0.86506 ± 0.02409	0.85814 ± 0.01595
GROVER-Large	0.85611 ± 0.02360	0.97482 ± 0.05950	0.99128 ± 0.20295	0.94949 ± 0.04509	0.93766 ± 0.02508
MoleBERT	0.85095 ± 0.00179	0.90087 ± 0.00143	0.85553 ± 0.00129	0.89810 ± 0.00251	0.92186 ± 0.00229
UniMol-G	0.88173 ± 0.00610	1.02354 ± 0.00120	0.93210 ± 0.00477	1.03802 ± 0.00187	1.04872 ± 0.01065
UniMol-GA	0.86502 ± 0.01673	1.00795 ± 0.00280	0.93753 ± 0.00656	1.02734 ± 0.00115	1.02084 ± 0.00518
GCC	1.08948 ± 0.01179	1.08846 ± 0.00297	0.99263 ± 0.02319	1.04327 ± 0.00591	1.11480 ± 0.00476
GPT-GNN	0.99844 ± 0.04421	1.07041 ± 0.00471	0.97442 ± 0.02277	1.00635 ± 0.01086	1.06025 ± 0.01497
GraphCL	0.80679 ± 0.00961	0.95202 ± 0.00344	0.85519 ± 0.00702	0.93019 ± 0.00286	0.91766 ± 0.00540
InfoGraph	1.27223 ± 0.02065	1.14752 ± 0.00285	0.96789 ± 0.00464	1.04965 ± 0.00178	1.13490 ± 0.00342
JOAO	0.98905 ± 0.01532	1.11880 ± 0.00283	0.96509 ± 0.01134	1.00606 ± 0.00832	1.13530 ± 0.00686
JOAOv2	1.03371 ± 0.01119	1.13156 ± 0.00516	0.97031 ± 0.00879	1.00378 ± 0.00168	1.06666 ± 0.01057
PosPred	1.13722 ± 0.01261	1.13124 ± 0.00542	1.02547 ± 0.00576	0.97277 ± 0.00499	2.85493 ± 0.48378
3D-EMGP	1.61439 ± 0.03917	1.15415 ± 0.00159	1.01867 ± 0.00290	1.06410 ± 0.00059	1.13444 ± 0.00504
3D-InfoMax	0.86664 ± 0.00428	0.97351 ± 0.00472	0.91051 ± 0.00830	0.95146 ± 0.00248	1.00648 ± 0.00752
AttrMask	1.17462 ± 0.01072	1.12235 ± 0.00236	0.99850 ± 0.00205	1.05576 ± 0.00089	1.09945 ± 0.00341
EdgePred	0.91800 ± 0.00438	0.98045 ± 0.00285	0.88439 ± 0.00607	0.98964 ± 0.00131	1.00061 ± 0.00156
GraphMVP	1.02344 ± 0.00238	1.01007 ± 0.00171	0.93137 ± 0.00637	0.98132 ± 0.00169	0.99519 ± 0.00352
MolCLR-GIN	1.14514 ± 0.01081	1.15289 ± 0.00075	1.02760 ± 0.00060	1.06386 ± 0.00094	1.14165 ± 0.00202
MolCLR-GCN	1.14527 ± 0.01088	1.13224 ± 0.00860	1.05113 ± 0.00172	1.05787 ± 0.00097	1.12826 ± 0.01125
TransM	0.70501 ± 0.00482	0.89344 ± 0.00248	0.78493 ± 0.00628	0.85099 ± 0.00666	0.86028 ± 0.00645
MPG	0.70970 ± 0.00961	0.85194 ± 0.00216	0.84313 ± 0.00314	0.89190 ± 0.00537	0.80676 ± 0.00733
KANO	1.04278 ± 0.00890	1.09597 ± 0.00203	1.01726 ± 0.00149	1.02603 ± 0.00186	1.11223 ± 0.00050

Prediction is evaluated by RMSE. For each method, the mean and standard deviation are reported for the evaluation metrics.

Supplementary Table 36. Activity cliff prediction (cliff molecules) – Part III

Method	CHEMBL231 K_i	CHEMBL233 K_i	CHEMBL234 K_i	CHEMBL235 EC_{50}	CHEMBL236 K_i
qcGEM	0.79382 $\pm$ 0.01287	0.92786 $\pm$ 0.00537	0.71355 $\pm$ 0.00451	0.82323 $\pm$ 0.01289	0.84708 $\pm$ 0.00730
ECFP	0.98269 $\pm$ 0.02814	0.95531 $\pm$ 0.00414	0.70269 $\pm$ 0.00696	0.86321 $\pm$ 0.00738	0.81280 $\pm$ 0.00454
FCFP	0.88422 $\pm$ 0.00416	1.01232 $\pm$ 0.00716	0.73028 $\pm$ 0.00481	0.84442 $\pm$ 0.00660	0.85789 $\pm$ 0.00557
AvalonFP	0.99747 $\pm$ 0.01002	0.97059 $\pm$ 0.00380	0.73104 $\pm$ 0.00699	0.85328 $\pm$ 0.00275	0.87753 $\pm$ 0.00257
TTGEN	0.96446 $\pm$ 0.01071	1.02663 $\pm$ 0.00617	0.73632 $\pm$ 0.00473	0.93364 $\pm$ 0.00352	0.87991 $\pm$ 0.00351
APGEN	0.79009 $\pm$ 0.01299	1.03060 $\pm$ 0.00543	0.79308 $\pm$ 0.00825	0.86936 $\pm$ 0.00857	0.95212 $\pm$ 0.00597
RDKFP	0.82506 $\pm$ 0.00363	1.04514 $\pm$ 0.00295	0.78765 $\pm$ 0.00322	0.92458 $\pm$ 0.00365	0.98504 $\pm$ 0.00275
MACCS	0.89523 $\pm$ 0.00830	1.09993 $\pm$ 0.00303	0.82154 $\pm$ 0.00661	1.00879 $\pm$ 0.00372	1.05609 $\pm$ 0.01072
LayeredFP	0.88584 $\pm$ 0.00685	1.12274 $\pm$ 0.00457	0.84413 $\pm$ 0.00258	0.97145 $\pm$ 0.00290	1.02346 $\pm$ 0.00501
PatternFP	0.84755 $\pm$ 0.00976	1.07609 $\pm$ 0.00453	0.83658 $\pm$ 0.00281	0.95147 $\pm$ 0.00763	1.03456 $\pm$ 0.00582
Molformer	0.94433 $\pm$ 0.01302	0.97185 $\pm$ 0.00553	0.73739 $\pm$ 0.00514	0.74964 $\pm$ 0.00390	0.85933 $\pm$ 0.00578
GROVER-Base	0.92140 $\pm$ 0.05702	0.98054 $\pm$ 0.01577	0.75294 $\pm$ 0.00809	0.87031 $\pm$ 0.01071	0.88418 $\pm$ 0.00957
GROVER-Large	1.09552 $\pm$ 0.29462	0.99110 $\pm$ 0.01505	0.77159 $\pm$ 0.00758	0.92835 $\pm$ 0.05523	0.94650 $\pm$ 0.08697
MoleBERT	0.88387 $\pm$ 0.00417	1.03976 $\pm$ 0.00285	0.79124 $\pm$ 0.00207	0.87428 $\pm$ 0.00370	0.95618 $\pm$ 0.00335
UniMol-G	0.93460 $\pm$ 0.00971	1.10880 $\pm$ 0.00615	0.90192 $\pm$ 0.00237	1.07343 $\pm$ 0.00979	1.12195 $\pm$ 0.00587
UniMol-GA	0.88480 $\pm$ 0.01261	1.08140 $\pm$ 0.00386	0.88502 $\pm$ 0.00145	1.04327 $\pm$ 0.00792	1.10534 $\pm$ 0.00752
GCC	0.96470 $\pm$ 0.00487	1.13822 $\pm$ 0.00421	0.95552 $\pm$ 0.00239	1.09859 $\pm$ 0.00910	1.15886 $\pm$ 0.00361
GPT-GNN	1.05323 $\pm$ 0.01528	1.09714 $\pm$ 0.02046	0.90983 $\pm$ 0.00474	1.09011 $\pm$ 0.03188	1.18808 $\pm$ 0.01747
GraphCL	0.89684 $\pm$ 0.01263	1.09484 $\pm$ 0.00716	0.79521 $\pm$ 0.00216	0.95411 $\pm$ 0.00938	1.02648 $\pm$ 0.01053
InfoGraph	1.09292 $\pm$ 0.00315	1.25916 $\pm$ 0.00574	0.98605 $\pm$ 0.00326	1.14473 $\pm$ 0.00717	1.32831 $\pm$ 0.01363
JOAO	1.06075 $\pm$ 0.00947	1.23705 $\pm$ 0.02139	0.91022 $\pm$ 0.00294	1.13585 $\pm$ 0.01089	1.26433 $\pm$ 0.00691
JOAOv2	1.04998 $\pm$ 0.01713	1.22983 $\pm$ 0.00900	0.89030 $\pm$ 0.00233	1.10747 $\pm$ 0.01428	1.23800 $\pm$ 0.00947
PosPred	1.12234 $\pm$ 0.01448	1.23499 $\pm$ 0.02168	0.91710 $\pm$ 0.00244	1.08630 $\pm$ 0.01859	1.34040 $\pm$ 0.02130
3D-EMGP	1.20368 $\pm$ 0.00371	1.32170 $\pm$ 0.00275	1.04626 $\pm$ 0.00171	1.17730 $\pm$ 0.00811	1.30726 $\pm$ 0.00251
3D-InfoMax	0.90555 $\pm$ 0.00808	1.07543 $\pm$ 0.00515	0.83883 $\pm$ 0.00280	1.00572 $\pm$ 0.01615	1.03149 $\pm$ 0.00516
AttrMask	1.06427 $\pm$ 0.00263	1.19696 $\pm$ 0.00185	0.98715 $\pm$ 0.00292	1.10511 $\pm$ 0.00252	1.25652 $\pm$ 0.00565
EdgePred	1.01109 $\pm$ 0.00690	1.02412 $\pm$ 0.00082	0.85640 $\pm$ 0.00258	0.95793 $\pm$ 0.00187	0.98524 $\pm$ 0.00481
GraphMVP	0.92980 $\pm$ 0.00662	1.12339 $\pm$ 0.00314	0.89856 $\pm$ 0.00357	1.02156 $\pm$ 0.00635	1.08377 $\pm$ 0.00312
MolCLR-GIN	1.22732 $\pm$ 0.00188	1.32612 $\pm$ 0.00138	1.04361 $\pm$ 0.00135	1.16075 $\pm$ 0.00259	1.28268 $\pm$ 0.00987
MolCLR-GCN	1.23100 $\pm$ 0.01283	1.24741 $\pm$ 0.00067	0.98601 $\pm$ 0.00346	1.16293 $\pm$ 0.00931	1.30239 $\pm$ 0.00647
TransM	0.89233 $\pm$ 0.00667	0.95857 $\pm$ 0.00991	0.74511 $\pm$ 0.00358	0.84574 $\pm$ 0.00498	0.90675 $\pm$ 0.00927
MPG	0.95467 $\pm$ 0.01026	1.02368 $\pm$ 0.00430	0.79168 $\pm$ 0.00180	0.86310 $\pm$ 0.00600	0.90114 $\pm$ 0.00356
KANO	1.05811 $\pm$ 0.00299	1.15715 $\pm$ 0.00142	0.93051 $\pm$ 0.00167	1.07806 $\pm$ 0.00805	1.15938 $\pm$ 0.00330

Prediction is evaluated by RMSE. For each method, the mean and standard deviation are reported for the evaluation metrics.

Supplementary Table 37. Activity cliff prediction (cliff molecules) – Part IV

Method	CHEMBL237 EC_{50}	CHEMBL237 K_i	CHEMBL238 K_i	CHEMBL239 EC_{50}	CHEMBL244 K_i
qcGEM	0.91305 ± 0.00897	0.78570 ± 0.00411	0.79172 ± 0.01071	0.86289 ± 0.00662	0.91683 ± 0.00366
ECFP	0.86450 ± 0.00770	0.81297 ± 0.00593	0.86284 ± 0.01151	0.96973 ± 0.00283	0.93422 ± 0.00513
FCFP	0.99089 ± 0.00795	0.81106 ± 0.00439	0.81762 ± 0.00727	0.89730 ± 0.00474	0.93284 ± 0.00119
AvalonFP	0.92521 ± 0.00558	0.90149 ± 0.00294	0.75737 ± 0.00538	0.88798 ± 0.00919	0.92218 ± 0.00700
TTGEN	1.07088 ± 0.01098	0.87983 ± 0.00115	0.77028 ± 0.00287	1.06024 ± 0.00553	1.01041 ± 0.00693
APGEN	1.00555 ± 0.00949	0.90024 ± 0.00455	0.84135 ± 0.01361	1.02930 ± 0.00437	1.05222 ± 0.00712
RDKFP	0.99485 ± 0.00549	1.02230 ± 0.00541	0.82424 ± 0.01384	1.00646 ± 0.00347	0.94334 ± 0.00334
MACCS	0.97926 ± 0.00558	1.00367 ± 0.00563	0.82215 ± 0.00544	1.01983 ± 0.00810	1.01731 ± 0.00749
LayeredFP	1.12702 ± 0.00314	1.00089 ± 0.00175	0.81767 ± 0.01159	0.95071 ± 0.00477	1.04555 ± 0.00588
PatternFP	1.15476 ± 0.00263	1.03527 ± 0.00423	0.88942 ± 0.00866	1.00067 ± 0.00399	1.05214 ± 0.00510
Molformer	0.88770 ± 0.00576	0.84492 ± 0.00231	0.75258 ± 0.00600	0.93818 ± 0.00807	0.89122 ± 0.00408
GROVER-Base	0.95409 ± 0.03583	0.86956 ± 0.01749	0.83579 ± 0.01477	0.96034 ± 0.01939	0.95721 ± 0.01598
GROVER-Large	1.16119 ± 0.14953	0.95885 ± 0.12494	0.98765 ± 0.20335	0.99131 ± 0.02628	0.96997 ± 0.02898
MoleBERT	1.02355 ± 0.00164	0.94937 ± 0.00093	0.81708 ± 0.01021	0.99355 ± 0.00172	1.02361 ± 0.00151
UniMol-G	1.29465 ± 0.00237	1.07916 ± 0.00975	1.01530 ± 0.00915	1.04010 ± 0.00331	1.12382 ± 0.00225
UniMol-GA	1.25773 ± 0.00235	1.03099 ± 0.00598	0.98680 ± 0.01949	1.01868 ± 0.00849	1.08574 ± 0.00194
GCC	1.23073 ± 0.00489	1.09346 ± 0.00629	0.95191 ± 0.02096	1.06395 ± 0.00485	1.28203 ± 0.00409
GPT-GNN	1.28034 ± 0.00721	1.00602 ± 0.02174	0.93557 ± 0.01588	1.04268 ± 0.00947	1.17052 ± 0.01320
GraphCL	1.09355 ± 0.00534	0.96850 ± 0.00430	0.83551 ± 0.00876	0.98311 ± 0.00330	1.01300 ± 0.00399
InfoGraph	1.30526 ± 0.00558	1.26183 ± 0.00355	1.16603 ± 0.01347	1.12158 ± 0.00385	1.35134 ± 0.00428
JOAO	1.26596 ± 0.01239	1.25208 ± 0.01535	1.12327 ± 0.02976	1.09892 ± 0.00727	1.25978 ± 0.00778
JOAOv2	1.22472 ± 0.00854	1.22041 ± 0.01465	1.07728 ± 0.01721	1.10698 ± 0.00724	1.18362 ± 0.00577
PosPred	1.43195 ± 0.05355	1.21515 ± 0.01635	1.11738 ± 0.02909	1.09229 ± 0.01420	1.19951 ± 0.00476
3D-EMGP	1.39135 ± 0.01865	1.42260 ± 0.00601	1.19947 ± 0.00354	1.12224 ± 0.00959	1.53754 ± 0.00315
3D-InfoMax	1.08927 ± 0.00304	0.94724 ± 0.00369	0.94370 ± 0.01703	1.02483 ± 0.01066	1.02696 ± 0.01056
AttrMask	1.23014 ± 0.00240	1.15408 ± 0.00334	1.10797 ± 0.00428	1.13617 ± 0.00705	1.38227 ± 0.00187
EdgePred	1.06612 ± 0.00303	0.95416 ± 0.00508	0.90435 ± 0.00565	1.09418 ± 0.00702	1.02152 ± 0.00150
GraphMVP	1.16091 ± 0.00363	1.09234 ± 0.00504	0.84218 ± 0.01067	1.03855 ± 0.00375	1.15526 ± 0.00288
MolCLR-GIN	1.35261 ± 0.00055	1.33284 ± 0.01329	1.18554 ± 0.00164	1.17551 ± 0.00216	1.35088 ± 0.02075
MolCLR-GCN	1.28721 ± 0.00787	1.12527 ± 0.00372	1.17531 ± 0.00621	1.16936 ± 0.00873	1.36498 ± 0.00164
TransM	0.96616 ± 0.00651	0.87034 ± 0.00217	0.78938 ± 0.00884	0.92809 ± 0.00505	1.00628 ± 0.00327
MPG	0.96759 ± 0.00639	0.87463 ± 0.00483	0.74682 ± 0.00692	0.87864 ± 0.00389	0.92414 ± 0.00779
KANO	1.21768 ± 0.00161	1.13270 ± 0.00126	0.98554 ± 0.00234	1.08318 ± 0.00554	1.28873 ± 0.00224

Prediction is evaluated by RMSE. For each method, the mean and standard deviation are reported for the evaluation metrics.

Supplementary Table 38. Activity cliff prediction (cliff molecules) – Part V

Method	CHEMBL262 K_i	CHEMBL264 K_i	CHEMBL2835 K_i	CHEMBL287 K_i	CHEMBL2971 K_i
qcGEM	0.92953 $\pm$ 0.01290	0.72567 $\pm$ 0.00293	0.72002 $\pm$ 0.02610	0.73294 $\pm$ 0.02753	0.72481 $\pm$ 0.02142
ECFP	0.96082 $\pm$ 0.01975	0.81008 $\pm$ 0.00409	0.83094 $\pm$ 0.01197	0.95715 $\pm$ 0.01054	0.80524 $\pm$ 0.02073
FCFP	0.86413 $\pm$ 0.00738	0.75346 $\pm$ 0.00236	0.77092 $\pm$ 0.00596	0.92119 $\pm$ 0.00841	0.87272 $\pm$ 0.01902
AvalonFP	0.88962 $\pm$ 0.00568	0.77095 $\pm$ 0.00301	0.85026 $\pm$ 0.02006	0.89819 $\pm$ 0.01244	0.87783 $\pm$ 0.00687
TTGEN	0.95224 $\pm$ 0.00708	0.84433 $\pm$ 0.00393	0.82819 $\pm$ 0.00915	0.90255 $\pm$ 0.01006	0.81504 $\pm$ 0.01035
APGEN	0.87116 $\pm$ 0.00750	0.82966 $\pm$ 0.00807	0.96320 $\pm$ 0.01882	0.92671 $\pm$ 0.00922	0.87211 $\pm$ 0.01650
RDKFP	0.99241 $\pm$ 0.00439	0.88399 $\pm$ 0.00443	0.78090 $\pm$ 0.02426	0.98549 $\pm$ 0.00223	0.77165 $\pm$ 0.01043
MACCS	0.99307 $\pm$ 0.00717	0.91096 $\pm$ 0.00901	0.79652 $\pm$ 0.01109	0.93186 $\pm$ 0.01354	0.67825 $\pm$ 0.00748
LayeredFP	0.87493 $\pm$ 0.00260	0.83125 $\pm$ 0.00580	0.83848 $\pm$ 0.01078	0.98822 $\pm$ 0.00474	0.84001 $\pm$ 0.00319
PatternFP	0.96325 $\pm$ 0.00734	0.86140 $\pm$ 0.00442	0.72035 $\pm$ 0.01089	0.89617 $\pm$ 0.00589	0.73103 $\pm$ 0.00477
Molformer	0.94818 $\pm$ 0.01229	0.75080 $\pm$ 0.00447	0.74416 $\pm$ 0.00859	0.85435 $\pm$ 0.00635	0.81124 $\pm$ 0.01457
GROVER-Base	0.94198 $\pm$ 0.02672	0.78524 $\pm$ 0.01457	0.76169 $\pm$ 0.01347	0.80525 $\pm$ 0.01104	0.72940 $\pm$ 0.01882
GROVER-Large	1.18362 $\pm$ 0.39219	0.78679 $\pm$ 0.01937	0.90206 $\pm$ 0.18254	0.91288 $\pm$ 0.11405	0.90281 $\pm$ 0.20094
MoleBERT	0.94832 $\pm$ 0.00832	0.89147 $\pm$ 0.00185	0.75554 $\pm$ 0.01234	0.85098 $\pm$ 0.00202	0.80893 $\pm$ 0.00509
UniMol-G	0.97248 $\pm$ 0.00380	0.96889 $\pm$ 0.00320	0.90639 $\pm$ 0.01320	0.91550 $\pm$ 0.00826	0.85996 $\pm$ 0.00476
UniMol-GA	0.95350 $\pm$ 0.00663	0.93334 $\pm$ 0.00526	0.94360 $\pm$ 0.00630	0.86738 $\pm$ 0.00787	0.85138 $\pm$ 0.00709
GCC	1.07654 $\pm$ 0.00504	0.97800 $\pm$ 0.00203	1.04977 $\pm$ 0.01996	0.99118 $\pm$ 0.00660	1.05050 $\pm$ 0.01351
GPT-GNN	0.98757 $\pm$ 0.02195	0.91254 $\pm$ 0.01076	0.88184 $\pm$ 0.02547	0.91131 $\pm$ 0.01758	1.01372 $\pm$ 0.03913
GraphCL	1.03274 $\pm$ 0.00842	0.83174 $\pm$ 0.00264	0.83023 $\pm$ 0.02291	0.93518 $\pm$ 0.00463	0.65421 $\pm$ 0.00383
InfoGraph	1.04330 $\pm$ 0.00536	1.02443 $\pm$ 0.00618	1.00731 $\pm$ 0.01833	1.02650 $\pm$ 0.01019	0.89018 $\pm$ 0.00603
JOAO	1.04395 $\pm$ 0.00277	1.01412 $\pm$ 0.00476	0.75572 $\pm$ 0.01238	1.04274 $\pm$ 0.01056	0.87345 $\pm$ 0.01849
JOAOv2	1.01769 $\pm$ 0.00561	0.99899 $\pm$ 0.00501	0.86270 $\pm$ 0.02191	0.95085 $\pm$ 0.00812	0.87565 $\pm$ 0.01579
PosPred	0.99708 $\pm$ 0.00690	1.04892 $\pm$ 0.00586	0.88774 $\pm$ 0.01228	0.98717 $\pm$ 0.01499	0.96261 $\pm$ 0.05718
3D-EMGP	1.14354 $\pm$ 0.00175	1.03618 $\pm$ 0.00257	1.27548 $\pm$ 0.13180	1.27382 $\pm$ 0.20975	1.06902 $\pm$ 0.00077
3D-InfoMax	0.96522 $\pm$ 0.00710	0.88613 $\pm$ 0.01151	0.73941 $\pm$ 0.01478	0.91834 $\pm$ 0.00526	0.91153 $\pm$ 0.00279
AttrMask	1.11617 $\pm$ 0.00348	1.01784 $\pm$ 0.00353	0.99342 $\pm$ 0.00155	1.04727 $\pm$ 0.00026	0.98481 $\pm$ 0.00393
EdgePred	0.98158 $\pm$ 0.00449	0.89525 $\pm$ 0.00132	0.77558 $\pm$ 0.00504	0.96050 $\pm$ 0.00199	0.94167 $\pm$ 0.00243
GraphMVP	0.94403 $\pm$ 0.00910	0.96807 $\pm$ 0.00210	0.93462 $\pm$ 0.00914	0.89168 $\pm$ 0.00187	0.98969 $\pm$ 0.01139
MolCLR-GIN	1.14332 $\pm$ 0.00269	1.02001 $\pm$ 0.00095	1.44651 $\pm$ 0.04675	1.15001 $\pm$ 0.00386	0.99783 $\pm$ 0.00408
MolCLR-GCN	1.02764 $\pm$ 0.00231	1.02349 $\pm$ 0.00272	1.37003 $\pm$ 0.01767	1.05355 $\pm$ 0.01147	1.10671 $\pm$ 0.00922
TransM	0.88254 $\pm$ 0.00496	0.78433 $\pm$ 0.00525	0.79186 $\pm$ 0.01549	0.85245 $\pm$ 0.00891	0.84466 $\pm$ 0.01345
MPG	0.80981 $\pm$ 0.00610	0.77777 $\pm$ 0.00609	0.71087 $\pm$ 0.02035	0.82515 $\pm$ 0.00226	0.80242 $\pm$ 0.00850
KANO	1.05999 $\pm$ 0.00284	1.00151 $\pm$ 0.00330	1.53726 $\pm$ 0.00505	0.92664 $\pm$ 0.00118	0.89664 $\pm$ 0.00513

Prediction is evaluated by RMSE. For each method, the mean and standard deviation are reported for the evaluation metrics.

Supplementary Table 39. Activity cliff prediction (cliff molecules) – Part VI

Method	CHEMBL3979 EC_{50}	CHEMBL4005 K_i	CHEMBL4203 K_i	CHEMBL4616 EC_{50}	CHEMBL4792 K_i
qcGEM	0.74273 $\pm$ 0.01492	0.80003 $\pm$ 0.00579	1.18727 $\pm$ 0.02147	0.75790 $\pm$ 0.00794	0.79306 $\pm$ 0.00734
ECFP	0.73070 $\pm$ 0.00551	0.76469 $\pm$ 0.00716	1.03542 $\pm$ 0.01318	0.78546 $\pm$ 0.00648	0.83952 $\pm$ 0.00932
FCFP	0.79905 $\pm$ 0.00648	0.74651 $\pm$ 0.00509	1.25876 $\pm$ 0.01646	0.89926 $\pm$ 0.00391	0.72211 $\pm$ 0.00239
AvalonFP	0.78202 $\pm$ 0.00469	0.78306 $\pm$ 0.00396	1.10813 $\pm$ 0.01877	0.77831 $\pm$ 0.00322	0.74788 $\pm$ 0.00349
TTGEN	0.77917 $\pm$ 0.01176	0.77298 $\pm$ 0.00391	1.26314 $\pm$ 0.00694	0.79405 $\pm$ 0.00400	0.77618 $\pm$ 0.00500
APGEN	0.87927 $\pm$ 0.01568	0.84010 $\pm$ 0.01212	1.23681 $\pm$ 0.00913	0.83043 $\pm$ 0.00679	0.87659 $\pm$ 0.00314
RDKFP	0.78833 $\pm$ 0.00662	0.74584 $\pm$ 0.00379	1.18752 $\pm$ 0.00793	0.80127 $\pm$ 0.00527	0.86715 $\pm$ 0.00216
MACCS	0.83389 $\pm$ 0.01112	0.79188 $\pm$ 0.00310	1.09084 $\pm$ 0.01213	0.79210 $\pm$ 0.00352	0.93091 $\pm$ 0.00399
LayeredFP	0.80831 $\pm$ 0.01407	0.77145 $\pm$ 0.00337	1.20955 $\pm$ 0.00802	0.82465 $\pm$ 0.00194	0.86507 $\pm$ 0.00381
PatternFP	0.87090 $\pm$ 0.01102	0.78293 $\pm$ 0.00212	1.13293 $\pm$ 0.02102	0.74664 $\pm$ 0.00383	0.82514 $\pm$ 0.00457
Molformer	0.75433 $\pm$ 0.01550	0.83333 $\pm$ 0.00644	1.09629 $\pm$ 0.00964	0.74517 $\pm$ 0.00610	0.74065 $\pm$ 0.00195
GROVER-Base	0.83585 $\pm$ 0.01751	0.85206 $\pm$ 0.01227	1.09928 $\pm$ 0.01532	0.77668 $\pm$ 0.00562	0.84881 $\pm$ 0.02057
GROVER-Large	0.96130 $\pm$ 0.13885	0.93553 $\pm$ 0.14487	1.33756 $\pm$ 0.32377	0.83930 $\pm$ 0.07668	0.93473 $\pm$ 0.08795
MoleBERT	0.75658 $\pm$ 0.01165	0.80683 $\pm$ 0.00509	1.09251 $\pm$ 0.00540	0.77656 $\pm$ 0.00109	0.82057 $\pm$ 0.00282
UniMol-G	0.95453 $\pm$ 0.02205	0.87230 $\pm$ 0.00274	1.09412 $\pm$ 0.00812	0.79006 $\pm$ 0.00199	0.96507 $\pm$ 0.00695
UniMol-GA	0.92458 $\pm$ 0.02660	0.85548 $\pm$ 0.00542	1.08510 $\pm$ 0.01127	0.78057 $\pm$ 0.00336	0.92424 $\pm$ 0.00709
GCC	0.99883 $\pm$ 0.01632	0.91840 $\pm$ 0.00960	1.00860 $\pm$ 0.00613	0.90126 $\pm$ 0.00220	1.01019 $\pm$ 0.00243
GPT-GNN	1.01544 $\pm$ 0.04273	0.85842 $\pm$ 0.01217	1.19190 $\pm$ 0.03161	0.91258 $\pm$ 0.00901	0.95462 $\pm$ 0.01473
GraphCL	0.84477 $\pm$ 0.01533	0.84921 $\pm$ 0.00637	1.42574 $\pm$ 0.01666	0.78724 $\pm$ 0.00356	0.76994 $\pm$ 0.00537
InfoGraph	1.02910 $\pm$ 0.01190	1.01878 $\pm$ 0.00201	1.04262 $\pm$ 0.01208	0.85831 $\pm$ 0.00788	1.17169 $\pm$ 0.00438
JOAO	1.01994 $\pm$ 0.02800	0.93554 $\pm$ 0.00603	1.23149 $\pm$ 0.01690	0.83842 $\pm$ 0.01691	1.12748 $\pm$ 0.00542
JOAOv2	1.04168 $\pm$ 0.00767	0.88827 $\pm$ 0.00642	1.15554 $\pm$ 0.00577	0.83585 $\pm$ 0.00623	1.03579 $\pm$ 0.01130
PosPred	1.03485 $\pm$ 0.01901	0.98975 $\pm$ 0.00991	1.18663 $\pm$ 0.00322	0.87250 $\pm$ 0.00874	1.01349 $\pm$ 0.01639
3D-EMGP	1.02868 $\pm$ 0.01297	1.10301 $\pm$ 0.00208	1.08570 $\pm$ 0.00829	0.85025 $\pm$ 0.00209	1.61445 $\pm$ 0.48517
3D-InfoMax	0.79110 $\pm$ 0.02021	0.80932 $\pm$ 0.00267	1.17406 $\pm$ 0.01914	0.82136 $\pm$ 0.00174	0.85300 $\pm$ 0.00184
AttrMask	1.02907 $\pm$ 0.00961	0.94099 $\pm$ 0.00289	1.07105 $\pm$ 0.00265	0.84772 $\pm$ 0.00077	1.02412 $\pm$ 0.00363
EdgePred	0.83529 $\pm$ 0.00554	0.82941 $\pm$ 0.00258	1.06354 $\pm$ 0.00744	0.83297 $\pm$ 0.00205	0.84968 $\pm$ 0.00184
GraphMVP	0.91514 $\pm$ 0.01244	0.81326 $\pm$ 0.00436	1.17219 $\pm$ 0.03496	0.80697 $\pm$ 0.00337	0.92666 $\pm$ 0.00268
MolCLR-GIN	1.06835 $\pm$ 0.01024	1.08879 $\pm$ 0.00130	1.07823 $\pm$ 0.00117	0.84904 $\pm$ 0.00125	1.19249 $\pm$ 0.00175
MolCLR-GCN	1.05838 $\pm$ 0.01452	1.08741 $\pm$ 0.00374	1.09202 $\pm$ 0.00115	0.86082 $\pm$ 0.00121	1.09334 $\pm$ 0.00301
TransM	0.75475 $\pm$ 0.00405	0.80286 $\pm$ 0.00297	1.10887 $\pm$ 0.02851	0.81968 $\pm$ 0.00238	0.80783 $\pm$ 0.00707
MPG	0.77107 $\pm$ 0.01214	0.75406 $\pm$ 0.00688	1.13191 $\pm$ 0.01112	0.78330 $\pm$ 0.00576	0.73250 $\pm$ 0.00792
KANO	0.92128 $\pm$ 0.00404	1.06406 $\pm$ 0.00483	1.10935 $\pm$ 0.00466	0.83837 $\pm$ 0.00254	1.04204 $\pm$ 0.00242

Prediction is evaluated by RMSE. For each method, the mean and standard deviation are reported for the evaluation metrics.

3.3.3 Protein-ligand interaction prediction

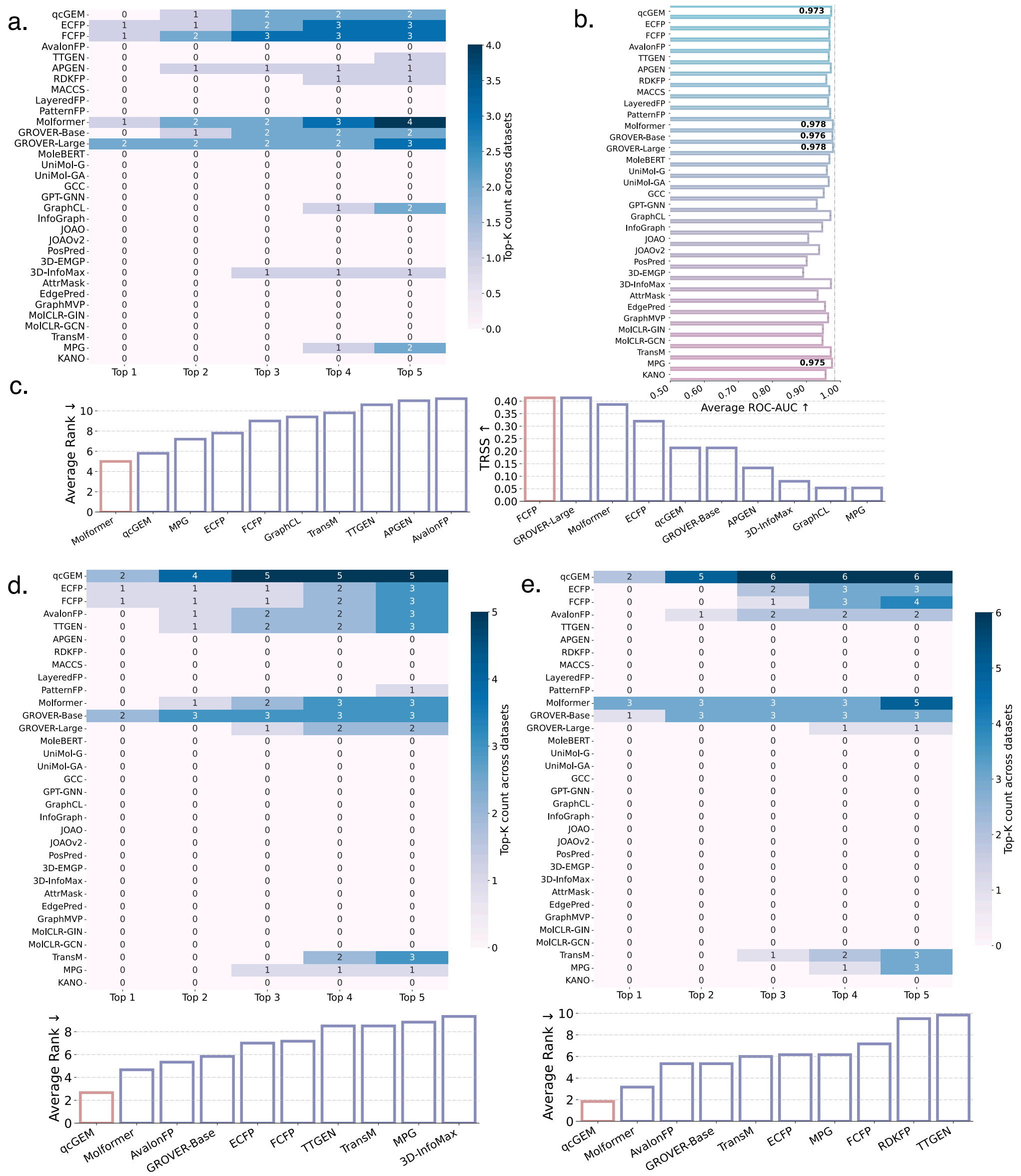

Supplementary Figure 16. Supplementary information of the protein–ligand interaction prediction tasks. a) Heatmap of Top-K occupancy across the five common protein–ligand interaction tasks (Human, *C. elegans*, BindingDB, DAVIS and KIBA). **b)** Mean ROC-AUC bar plot of the three classification tasks among them (Human, *C. elegans* and BindingDB). **c)** Average rank (left; lower is better) and TRSS (right; higher is better) of the same five common tasks. **d)** Heatmap (upper) and average rank (lower) of the six opioid protein–ligand interaction tasks under scaffold split. **e)** Heatmap (upper) and average rank (lower) of the six opioid protein–ligand interaction tasks under random split.

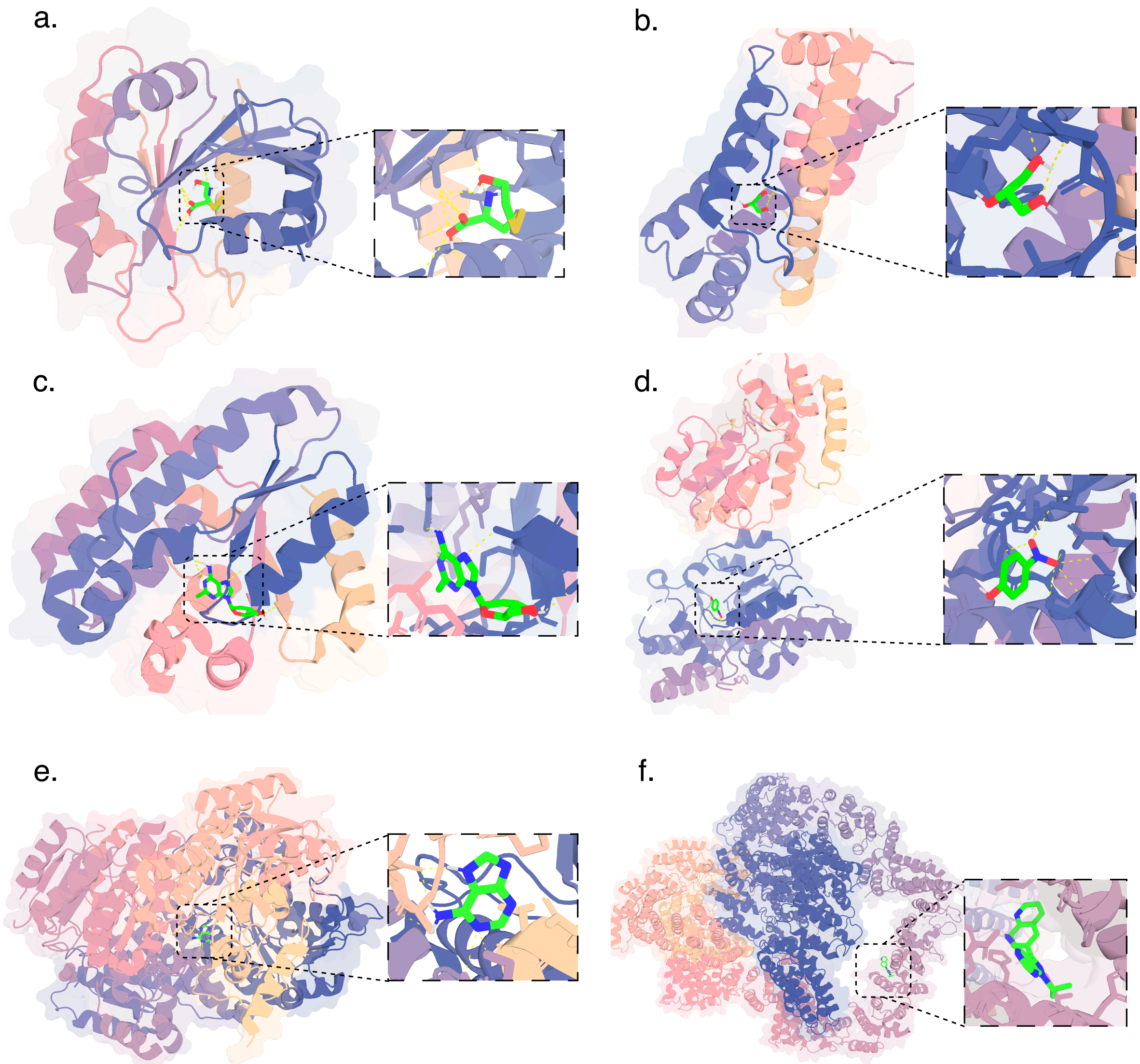

Supplementary Figure 17. Additional docking results. This figure supplements Figure 4e, showing more examples for the atomic level interaction between the predicted positive ligand and target protein. The presented proteins include **a)** 4ILE; **b)** 1SWX; **c)** 1P5Z; **d)** 1ZD1; **e)** 1YXM; **f)** 5W1R.

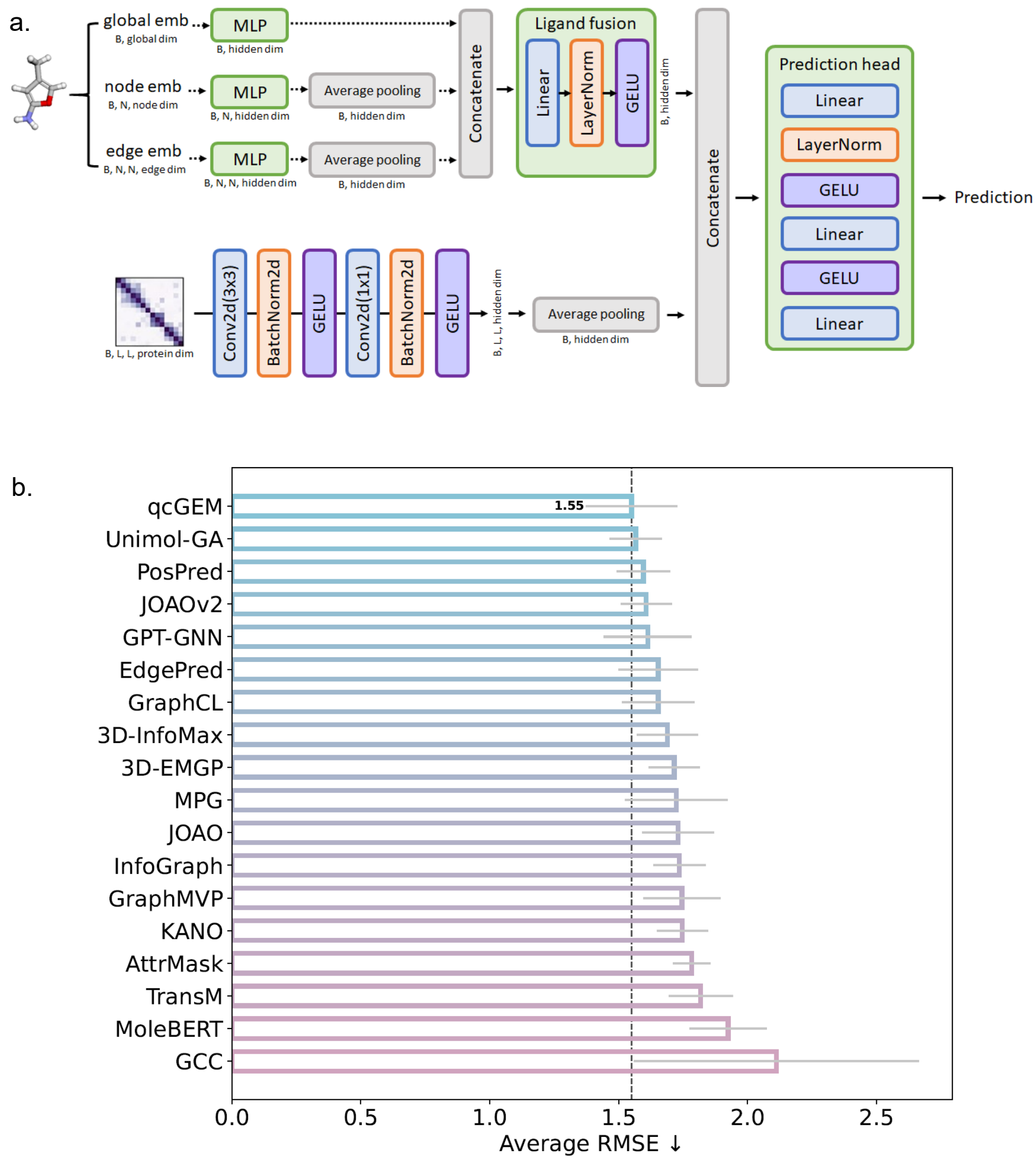

Supplementary Figure 18. Performance comparison of qcGEM against state-of-the-art graph-based models on the CASF-2016 benchmark. a) Architecture of the probe network for protein-ligand interaction prediction. The ligand representation is derived from three embedding sources: (i) global embedding (size B × global dim), processed by an MLP to yield a global feature vector; (ii) node embeddings (B × N × node dim), transformed by an MLP and pooled via average pooling to obtain a node-level global representation; and (iii) edge embeddings (B × N × N × edge dim), similarly projected and pooled. All three branches (global, node, and edge) are depicted with dashed arrows to signify their optional nature: each branch is included only if the ligand representation method supplies the corresponding embedding level, and omitted otherwise. The remaining available features are concatenated and fused through a ligand-specific module (Linear → LayerNorm → GELU) to produce a unified ligand representation (B × hidden dim). Concurrently, the protein 2D pair representation (B × L × L × protein dim) is processed by a lightweight 2D CNN (Conv2d–BatchNorm2d–GELU ×2) followed by average pooling over valid residue pairs, yielding a protein global feature (B × hidden dim). The ligand and protein representations are concatenated (B × 2 hidden dim) and fed into the prediction head—a multi-layer MLP (Linear → LayerNorm → GELU → Linear → GELU → Linear)—to output the final binding affinity. **b)** Average RMSE (↓ indicates lower is better) for qcGEM and baseline models. qcGEM achieves the best performance. The vertical dashed line is aligned with the average RMSE of qcGEM to highlight its comparative advantage. Error bars indicate standard deviation from five independent runs.

Supplementary Table 40. Protein–ligand interaction

Method	Classification			Regression	
	Human	*C. elegans*	BindingDB	DAVIS	KIBA
qcGEM	0.97054 ± 0.00176	0.98747 ± 0.00106	0.95972 ± 0.00435	0.59304 ± 0.00231	0.45545 ± 0.00333
ECFP	0.96192 ± 0.00158	0.97796 ± 0.00070	0.96494 ± 0.00152	0.59325 ± 0.00564	0.45124 ± 0.00387
FCFP	0.95860 ± 0.00093	0.97620 ± 0.00119	0.96583 ± 0.00150	0.59211 ± 0.00270	0.45216 ± 0.00384
AvalonFP	0.95955 ± 0.00273	0.98377 ± 0.00172	0.96103 ± 0.00213	0.60549 ± 0.00249	0.46771 ± 0.00276
TTGEN	0.97084 ± 0.00203	0.96475 ± 0.00066	0.96107 ± 0.00142	0.60205 ± 0.00507	0.46652 ± 0.00439
APGEN	0.97929 ± 0.00040	0.98082 ± 0.00195	0.95429 ± 0.00148	0.61182 ± 0.00143	0.47185 ± 0.00446
RDKFP	0.95193 ± 0.00092	0.96178 ± 0.00078	0.96126 ± 0.00156	0.59424 ± 0.00527	0.47321 ± 0.00320
MACCS	0.96923 ± 0.00081	0.97295 ± 0.00109	0.95937 ± 0.00171	0.60231 ± 0.00330	0.48871 ± 0.00119
LayeredFP	0.95808 ± 0.00111	0.98071 ± 0.00142	0.95210 ± 0.00091	0.62502 ± 0.00357	0.50139 ± 0.00427
PatternFP	0.96423 ± 0.00139	0.98142 ± 0.00142	0.96371 ± 0.00093	0.61022 ± 0.00461	0.48900 ± 0.00125
Molformer	0.98046 ± 0.00161	0.99371 ± 0.00064	0.95863 ± 0.00119	0.59428 ± 0.00542	0.46355 ± 0.00374
GROVER-Base	0.97049 ± 0.00285	0.99263 ± 0.00273	0.96596 ± 0.00174	0.78004 ± 0.02173	0.59794 ± 0.01976
GROVER-Large	0.97308 ± 0.00347	0.99424 ± 0.00125	0.96645 ± 0.00097	0.82653 ± 0.02379	0.62969 ± 0.03116
MoleBERT	0.96717 ± 0.00285	0.97627 ± 0.00080	0.95866 ± 0.00080	0.60966 ± 0.00165	0.49339 ± 0.00127
UniMol-G	0.96190 ± 0.00173	0.97754 ± 0.00190	0.94040 ± 0.00106	0.60465 ± 0.00329	0.51170 ± 0.00384
UniMol-GA	0.96825 ± 0.00066	0.98105 ± 0.00073	0.94671 ± 0.00075	0.61317 ± 0.00668	0.52615 ± 0.00332
GCC	0.95959 ± 0.00113	0.96567 ± 0.00142	0.92687 ± 0.00118	0.61623 ± 0.00128	0.54093 ± 0.00610
GPT-GNN	0.92559 ± 0.00131	0.93235 ± 0.00063	0.93294 ± 0.00223	0.63679 ± 0.00342	0.60068 ± 0.00700
GraphCL	0.97533 ± 0.00128	0.98858 ± 0.00034	0.94713 ± 0.00316	0.60548 ± 0.00273	0.47136 ± 0.00251
InfoGraph	0.94729 ± 0.00074	0.96655 ± 0.00177	0.92464 ± 0.00153	0.60889 ± 0.00283	0.53806 ± 0.00617
JOAO	0.90644 ± 0.00174	0.89290 ± 0.00178	0.91628 ± 0.00167	0.66701 ± 0.00223	0.66450 ± 0.00327
JOAOv2	0.94906 ± 0.00191	0.93398 ± 0.00206	0.92811 ± 0.00152	0.63505 ± 0.00270	0.61312 ± 0.00847
PosPred	0.89299 ± 0.00115	0.89249 ± 0.00137	0.91652 ± 0.00149	0.65790 ± 0.00185	0.70035 ± 0.00148
3D-EMGP	0.89205 ± 0.00250	0.88058 ± 0.00136	0.89808 ± 0.00255	0.75138 ± 0.00223	0.71894 ± 0.00130
3D-InfoMax	0.97550 ± 0.00080	0.98171 ± 0.00101	0.95826 ± 0.00116	0.61263 ± 0.00392	0.49375 ± 0.00253
AttrMask	0.93825 ± 0.00131	0.94365 ± 0.00099	0.91652 ± 0.00134	0.64440 ± 0.00993	0.64319 ± 0.00294
EdgePred	0.95116 ± 0.00159	0.97024 ± 0.00034	0.94195 ± 0.00215	0.61419 ± 0.00112	0.52818 ± 0.00458
GraphMVP	0.96761 ± 0.00102	0.97513 ± 0.00177	0.94780 ± 0.00267	0.59551 ± 0.00135	0.48850 ± 0.00173
MolCLR-GIN	0.95527 ± 0.00056	0.95565 ± 0.00065	0.93376 ± 0.00054	0.63494 ± 0.00126	0.62248 ± 0.00497
MolCLR-GCN	0.94638 ± 0.00163	0.97621 ± 0.00068	0.91871 ± 0.00173	0.65388 ± 0.00192	0.63639 ± 0.00924
TransM	0.96881 ± 0.00110	0.98720 ± 0.00038	0.95820 ± 0.00144	0.59516 ± 0.00215	0.47381 ± 0.00589
MPG	0.97174 ± 0.00071	0.98901 ± 0.00113	0.96400 ± 0.00102	0.60317 ± 0.00351	0.47772 ± 0.00314
KANO	0.96782 ± 0.00068	0.96875 ± 0.00030	0.93219 ± 0.00122	0.63408 ± 0.00209	0.61726 ± 0.00233

Prediction on the protein-ligand binding affinity is evaluated by RMSE. For each method, the mean and standard deviation are reported for the evaluation metrics.

Supplementary Table 41. Opioid-type receptors (scaffold split) - Part I

Method	CYP2D6	CYP3A4	DOR
qcGEM	1.67359 ± 0.11977	1.54310 ± 0.08609	1.86060 ± 0.18759
ECFP	1.69106 ± 0.15489	1.68475 ± 0.10026	2.01433 ± 0.12572
FCFP	1.71851 ± 0.12169	1.71609 ± 0.07582	1.98483 ± 0.23219
AvalonFP	1.71808 ± 0.12356	1.65212 ± 0.08906	1.92614 ± 0.16346
TTGEN	1.77929 ± 0.06985	1.81053 ± 0.07974	1.91847 ± 0.19714
APGEN	1.74078 ± 0.06465	1.76221 ± 0.14448	2.08468 ± 0.16354
RDKFP	1.78772 ± 0.09054	1.69711 ± 0.10709	2.08825 ± 0.23066
MACCS	1.71905 ± 0.07901	1.82158 ± 0.05916	2.15502 ± 0.15034
LayeredFP	1.81976 ± 0.09233	1.90096 ± 0.08395	2.19604 ± 0.17069
PatternFP	1.76391 ± 0.05947	1.76459 ± 0.03919	2.15975 ± 0.17630
Molformer	1.66859 ± 0.13922	1.59040 ± 0.10298	1.99551 ± 0.10435
GROVER-Base	1.63458 ± 0.10250	1.51508 ± 0.11255	2.15838 ± 0.20634
GROVER-Large	1.67574 ± 0.06625	1.57615 ± 0.04018	2.30733 ± 0.48658
MoleBERT	1.74504 ± 0.08589	1.91366 ± 0.13602	2.24863 ± 0.18310
UniMol-G	1.73736 ± 0.08990	1.84587 ± 0.07890	2.36668 ± 0.15503
UniMol-GA	1.72894 ± 0.08552	1.79267 ± 0.08590	2.29491 ± 0.15380
GCC	1.83755 ± 0.09922	1.98214 ± 0.15243	2.50533 ± 0.17012
GPT-GNN	1.83198 ± 0.12952	1.99387 ± 0.10118	2.61106 ± 0.20850
GraphCL	1.80711 ± 0.08996	1.84421 ± 0.12988	2.17693 ± 0.13616
InfoGraph	1.85303 ± 0.08961	2.01219 ± 0.10418	2.77379 ± 0.25718
JOAO	1.87284 ± 0.11800	2.00169 ± 0.10479	2.77685 ± 0.23952
JOAOv2	1.78593 ± 0.11412	1.93993 ± 0.12068	2.63164 ± 0.26156
PosPred	1.87588 ± 0.11756	2.05967 ± 0.09032	3.01632 ± 0.22282
3D-EMGP	1.87621 ± 0.11790	2.06653 ± 0.11222	2.98050 ± 0.24458
3D-InfoMax	1.70790 ± 0.09501	1.69538 ± 0.14087	2.13547 ± 0.09130
AttrMask	1.83366 ± 0.07507	2.03451 ± 0.10122	2.71342 ± 0.15976
EdgePred	1.84842 ± 0.10486	1.91545 ± 0.08998	2.23064 ± 0.22329
GraphMVP	1.70911 ± 0.08202	1.84129 ± 0.15802	2.22659 ± 0.13662
MolCLR-GIN	1.87240 ± 0.10495	2.02067 ± 0.12918	2.84559 ± 0.25217
MolCLR-GCN	1.82016 ± 0.08630	2.01802 ± 0.12688	2.78910 ± 0.18057
TransM	1.78070 ± 0.09916	1.70806 ± 0.10341	1.94293 ± 0.14883
MPG	1.74749 ± 0.13674	1.71297 ± 0.09763	2.06031 ± 0.20078
KANO	1.86954 ± 0.11762	2.05179 ± 0.09234	2.37762 ± 0.23589

Prediction on the protein-ligand binding affinity is evaluated by RMSE. For each method, the mean and standard deviation are reported for the evaluation metrics.

Supplementary Table 42. Opioid-type receptors (scaffold split) - Part II

Method	KOR	MDR1	MOR
qcGEM	1.95855 ± 0.19164	1.66537 ± 0.35216	2.09829 ± 0.11459
ECFP	1.94909 ± 0.19421	2.16968 ± 0.28119	2.05343 ± 0.17022
FCFP	2.04387 ± 0.06819	2.04542 ± 0.23901	2.01275 ± 0.15564
AvalonFP	2.14167 ± 0.13942	1.94063 ± 0.24241	2.03082 ± 0.24114
TTGEN	2.08665 ± 0.13802	1.95604 ± 0.31682	2.04218 ± 0.13232
APGEN	2.16463 ± 0.12676	2.12845 ± 0.27590	2.27244 ± 0.14260
RDKFP	2.18927 ± 0.13730	1.99739 ± 0.15830	2.17727 ± 0.17876
MACCS	2.26126 ± 0.15003	2.08024 ± 0.23815	2.31491 ± 0.18888
LayeredFP	2.36224 ± 0.13342	2.02661 ± 0.21232	2.35929 ± 0.17014
PatternFP	2.29680 ± 0.12863	1.83716 ± 0.35446	2.22122 ± 0.20869
Molformer	2.01795 ± 0.13037	1.90938 ± 0.39293	2.07425 ± 0.17608
GROVER-Base	2.16311 ± 0.06569	1.68883 ± 0.26138	2.25422 ± 0.17425
GROVER-Large	2.34572 ± 0.58508	2.08024 ± 0.44289	2.40249 ± 0.56257
MoleBERT	2.29311 ± 0.14489	2.26366 ± 0.45920	2.53324 ± 0.21588
UniMol-G	2.38390 ± 0.15759	2.06330 ± 0.33585	2.49280 ± 0.21667
UniMol-GA	2.32851 ± 0.15757	1.98359 ± 0.34358	2.41420 ± 0.23654
GCC	2.51514 ± 0.19380	2.73948 ± 0.76927	2.62633 ± 0.10930
GPT-GNN	2.56537 ± 0.06476	2.31906 ± 0.42965	2.56267 ± 0.14855
GraphCL	2.22754 ± 0.24232	2.06637 ± 0.28129	2.31526 ± 0.26149
InfoGraph	2.73390 ± 0.15523	3.09887 ± 0.95758	2.84301 ± 0.17002
JOAO	2.80746 ± 0.14649	3.07591 ± 1.17377	2.93562 ± 0.14796
JOAOv2	2.75910 ± 0.14269	2.63582 ± 0.75612	2.77544 ± 0.14689
PosPred	2.91260 ± 0.14692	3.62402 ± 1.17016	2.94447 ± 0.16441
3D-EMGP	3.02375 ± 0.17745	3.64009 ± 1.18306	2.99495 ± 0.17386
3D-InfoMax	2.23106 ± 0.17606	1.87371 ± 0.21954	2.27568 ± 0.20227
AttrMask	2.63554 ± 0.15730	3.02676 ± 0.41207	2.76701 ± 0.14810
EdgePred	2.36780 ± 0.15334	2.39910 ± 0.18501	2.37519 ± 0.28146
GraphMVP	2.42388 ± 0.20286	2.24329 ± 0.45723	2.46471 ± 0.21806
MolCLR-GIN	2.68266 ± 0.19451	3.60040 ± 1.13232	2.97040 ± 0.15844
MolCLR-GCN	2.66297 ± 0.19978	3.18366 ± 0.50652	2.85047 ± 0.13691
TransM	2.22238 ± 0.15557	1.80775 ± 0.26562	2.06706 ± 0.24870
MPG	2.16573 ± 0.15749	1.76174 ± 0.20012	2.11407 ± 0.13534
KANO	2.49777 ± 0.18206	2.42616 ± 0.50015	2.58118 ± 0.19883

Prediction on the protein-ligand binding affinity is evaluated by RMSE. For each method, the mean and standard deviation are reported for the evaluation metrics.

Supplementary Table 43. Opioid-type receptors (random split) - Part I

Method	CYP2D6	CYP3A4	DOR
qcGEM	1.61892 ± 0.08486	1.47263 ± 0.12433	1.64093 ± 0.11190
ECFP	1.73104 ± 0.10477	1.63858 ± 0.05992	1.66679 ± 0.06605
FCFP	1.79525 ± 0.08218	1.60317 ± 0.12399	1.70336 ± 0.08328
AvalonFP	1.69100 ± 0.10825	1.55593 ± 0.11602	1.71076 ± 0.03559
TTGEN	1.79450 ± 0.09267	1.70398 ± 0.08797	1.73681 ± 0.07568
APGEN	1.78668 ± 0.08500	1.74860 ± 0.06257	1.87442 ± 0.06480
RDKFP	1.73858 ± 0.05815	1.63919 ± 0.09440	1.92569 ± 0.07575
MACCS	1.74612 ± 0.07261	1.80009 ± 0.07681	1.99573 ± 0.08871
LayeredFP	1.77813 ± 0.08600	1.85961 ± 0.05551	1.98199 ± 0.15622
PatternFP	1.76920 ± 0.06591	1.78570 ± 0.07695	1.97789 ± 0.05040
Molformer	1.69044 ± 0.12193	1.61780 ± 0.09818	1.63233 ± 0.06917
GROVER-Base	1.60051 ± 0.07923	1.50273 ± 0.11543	1.82665 ± 0.10432
GROVER-Large	1.66427 ± 0.06848	1.66577 ± 0.13320	2.09568 ± 0.51646
MoleBERT	1.80728 ± 0.09608	1.93501 ± 0.07264	2.21111 ± 0.08803
UniMol-G	1.80105 ± 0.02892	1.94334 ± 0.08269	2.27197 ± 0.13854
UniMol-GA	1.76808 ± 0.03281	1.88817 ± 0.06731	2.15971 ± 0.11956
GCC	1.90309 ± 0.07237	1.97441 ± 0.07132	2.51562 ± 0.14934
GPT-GNN	1.88837 ± 0.05550	2.05321 ± 0.07283	2.54642 ± 0.13939
GraphCL	1.86844 ± 0.06387	1.78944 ± 0.09131	1.95776 ± 0.07906
InfoGraph	1.90691 ± 0.05288	2.06908 ± 0.06504	2.74681 ± 0.07551
JOAO	1.95795 ± 0.05532	2.06198 ± 0.06102	2.79344 ± 0.09492
JOAOv2	1.83868 ± 0.04877	2.02499 ± 0.06667	2.64909 ± 0.16905
PosPred	1.95621 ± 0.05354	2.13291 ± 0.06584	2.99397 ± 0.12013
3D-EMGP	1.95957 ± 0.05018	2.13120 ± 0.06914	2.99067 ± 0.10114
3D-InfoMax	1.78503 ± 0.06331	1.76512 ± 0.09849	1.96599 ± 0.07058
AttrMask	1.89751 ± 0.05789	2.09581 ± 0.07230	2.74872 ± 0.13656
EdgePred	1.84999 ± 0.05643	1.91286 ± 0.09580	2.12724 ± 0.13393
GraphMVP	1.80825 ± 0.06683	1.89139 ± 0.06599	2.25027 ± 0.06827
MolCLR-GIN	1.95979 ± 0.05328	2.13439 ± 0.06576	2.79591 ± 0.10122
MolCLR-GCN	1.89289 ± 0.06093	2.12974 ± 0.06910	2.80292 ± 0.11082
TransM	1.66110 ± 0.05943	1.65649 ± 0.09417	1.68489 ± 0.05997
MPG	1.70449 ± 0.08798	1.61295 ± 0.08810	1.74019 ± 0.08130
KANO	1.95574 ± 0.05166	2.12806 ± 0.06791	2.29583 ± 0.13069

Prediction on the protein-ligand binding affinity is evaluated by RMSE. For each method, the mean and standard deviation are reported for the evaluation metrics.

Supplementary Table 44. Opioid-type receptors (random split) - Part II

Method	KOR	MDR1	MOR
qcGEM	1.77381 $\pm$ 0.09356	1.36312 $\pm$ 0.15562	1.80140 $\pm$ 0.09625
ECFP	1.81664 $\pm$ 0.07743	1.67713 $\pm$ 0.14921	1.83036 $\pm$ 0.08603
FCFP	1.82177 $\pm$ 0.07242	1.58091 $\pm$ 0.19329	1.82528 $\pm$ 0.06955
AvalonFP	1.87313 $\pm$ 0.07111	1.56699 $\pm$ 0.07899	1.81168 $\pm$ 0.06525
TTGEN	1.85372 $\pm$ 0.07339	1.69102 $\pm$ 0.16351	1.87980 $\pm$ 0.07497
APGEN	2.00931 $\pm$ 0.09342	1.76538 $\pm$ 0.25888	2.06481 $\pm$ 0.11648
RDKFP	2.05408 $\pm$ 0.13368	1.51874 $\pm$ 0.21219	2.06720 $\pm$ 0.08113
MACCS	2.11098 $\pm$ 0.06949	1.74353 $\pm$ 0.13507	2.25653 $\pm$ 0.06435
LayeredFP	2.18557 $\pm$ 0.07223	1.65031 $\pm$ 0.26543	2.23743 $\pm$ 0.07053
PatternFP	2.22936 $\pm$ 0.05924	1.55637 $\pm$ 0.03706	2.16621 $\pm$ 0.04887
Molformer	1.73794 $\pm$ 0.06682	1.32395 $\pm$ 0.10254	1.83825 $\pm$ 0.06720
GROVER-Base	1.94738 $\pm$ 0.06492	1.33393 $\pm$ 0.03648	2.03754 $\pm$ 0.13479
GROVER-Large	2.27158 $\pm$ 0.66542	1.81746 $\pm$ 0.56697	2.24957 $\pm$ 0.41886
MoleBERT	2.28352 $\pm$ 0.09247	1.94603 $\pm$ 0.11012	2.37607 $\pm$ 0.11007
UniMol-G	2.37098 $\pm$ 0.07230	2.03134 $\pm$ 0.16336	2.48163 $\pm$ 0.09188
UniMol-GA	2.31433 $\pm$ 0.04529	1.91697 $\pm$ 0.12029	2.38328 $\pm$ 0.07441
GCC	2.49869 $\pm$ 0.16450	2.36949 $\pm$ 0.17789	2.56473 $\pm$ 0.04827
GPT-GNN	2.52218 $\pm$ 0.06844	2.26482 $\pm$ 0.19890	2.59408 $\pm$ 0.09592
GraphCL	2.10435 $\pm$ 0.04978	1.83307 $\pm$ 0.21821	2.08813 $\pm$ 0.10809
InfoGraph	2.74528 $\pm$ 0.10940	2.86780 $\pm$ 0.23060	2.80497 $\pm$ 0.11395
JOAO	2.76895 $\pm$ 0.11751	2.93665 $\pm$ 0.30239	2.89746 $\pm$ 0.12518
JOAOv2	2.64804 $\pm$ 0.06814	2.46767 $\pm$ 0.20933	2.75885 $\pm$ 0.14469
PosPred	2.96307 $\pm$ 0.10391	3.34463 $\pm$ 0.29906	2.93188 $\pm$ 0.12318
3D-EMGP	3.11414 $\pm$ 0.12334	3.33832 $\pm$ 0.30986	2.98117 $\pm$ 0.13408
3D-InfoMax	2.04257 $\pm$ 0.06392	1.57192 $\pm$ 0.16074	2.14088 $\pm$ 0.05096
AttrMask	2.64030 $\pm$ 0.11036	2.67400 $\pm$ 0.22394	2.72027 $\pm$ 0.12055
EdgePred	2.19706 $\pm$ 0.07817	1.92715 $\pm$ 0.11060	2.24353 $\pm$ 0.02822
GraphMVP	2.36952 $\pm$ 0.05424	1.86659 $\pm$ 0.20072	2.37417 $\pm$ 0.06838
MolCLR-GIN	2.74350 $\pm$ 0.09036	3.13041 $\pm$ 0.18733	2.97446 $\pm$ 0.14384
MolCLR-GCN	2.63993 $\pm$ 0.08984	2.82132 $\pm$ 0.14880	2.82667 $\pm$ 0.10254
TransM	1.90571 $\pm$ 0.04151	1.45780 $\pm$ 0.10797	1.91016 $\pm$ 0.07885
MPG	1.83908 $\pm$ 0.06585	1.42265 $\pm$ 0.10518	1.93376 $\pm$ 0.06688
KANO	2.41916 $\pm$ 0.05825	2.07631 $\pm$ 0.23379	2.56316 $\pm$ 0.09684

Prediction on the protein-ligand binding affinity is evaluated by RMSE. For each method, the mean and standard deviation are reported for the evaluation metrics.

Supplementary Table 45. Evaluation on CASF-2016

Method	RMSE
qcGEM	1.55016 ± 0.17246
MoleBERT	1.92483 ± 0.14676
UniMol-GA	1.56645 ± 0.09671
GCC	2.11232 ± 0.54897
GPT-GNN	1.61280 ± 0.16703
GraphCL	1.65353 ± 0.13728
InfoGraph	1.73584 ± 0.09710
JOAO	1.72986 ± 0.13502
JOAOv2	1.60695 ± 0.09533
PosPred	1.59625 ± 0.10016
3D-EMGP	1.71557 ± 0.09577
3D-InfoMax	1.68871 ± 0.11491
AttrMask	1.78388 ± 0.06863
EdgePred	1.65343 ± 0.14987
GraphMVP	1.74563 ± 0.14480
MPG	1.72329 ± 0.19490
TransM	1.81790 ± 0.12050
KANO	1.74740 ± 0.09504

Comparison is limited to baseline methods that explicitly include node-level embeddings and do not elicit GPU memory overflow. Due to the inherent limitations of some baseline graph models in handling certain ligand inputs, a consistent subset of CASF-2016, including a total of 265 complexes, is adopted for a fair head-to-head comparison.

3.4 Model fine-tuning

3.4.1 Experimental setting

Supplementary Table 46. Hyper-parameter settings of fine-tuning experiments

Method	Learning rate	Description
qcGEM (Frozen)	/	Fixed parameters
qcGEM	1×10^{-6}	With learning rate warm up
qcGEM-Hybrid (Frozen)	/	Fixed parameters
qcGEM-Hybrid	1×10^{-6}	With learning rate warm up
Molformer	1×10^{-5}, 1×10^{-6}, 1×10^{-7}	Select the Best-Performing Result
UniMol-G	1×10^{-5}, 1×10^{-6}, 1×10^{-7}	Select the Best-Performing Result
UniMol-GA	1×10^{-5}, 1×10^{-6}, 1×10^{-7}	Select the Best-Performing Result
UniMol-Doc	/	Strictly follow the document
UniMol-Doc woKfold	/	Follow the document but omit the K-fold setting

3.4.2 Experimental results

Supplementary Table 47. Evaluation results of fine-tuning experiments

Method	MoleculeACE (30 subsets)
qcGEM (Frozen)	0.8106 $\pm$ 0.1106
qcGEM	0.8021 $\pm$ 0.1158
qcGEM-Hybrid (Frozen)	0.8110 $\pm$ 0.1160
qcGEM-Hybrid	0.8053 $\pm$ 0.1172
Molformer	0.9253 $\pm$ 0.1066
UniMol-G	1.0513 $\pm$ 0.1725
UniMol-GA	1.0304 $\pm$ 0.1646
UniMol-Doc	0.8281 $\pm$ 0.1149
UniMol-Doc woKfold	0.9805 $\pm$ 0.1176

Fine-tuning performance is evaluated by RMSE. For each method, the mean and standard deviation over the 30 datasets from MoleculeACE are reported for the evaluation metric.

4 Supplementary Discussion

4.1 Ablation studies

4.1.1 The essential role of quantum features

To evaluate the contribution of quantum descriptors to qcGEM, we performed a fully retrained and capacity-matched ablation experiment, referred to as *Retrained-200-Epochs*.

We trained QM-informed and QM-ablated models for 200 epochs using identical architectures, parameter counts, input dimensions, training data, and optimization protocols. The QM-ablated model retained molecular topology, three-dimensional geometry, and all RDKit-derived features, while its node-level and edge-level QM channels and corresponding reconstruction targets were set to zero. The training and validation loss curves are shown in Supplementary Figure 19.

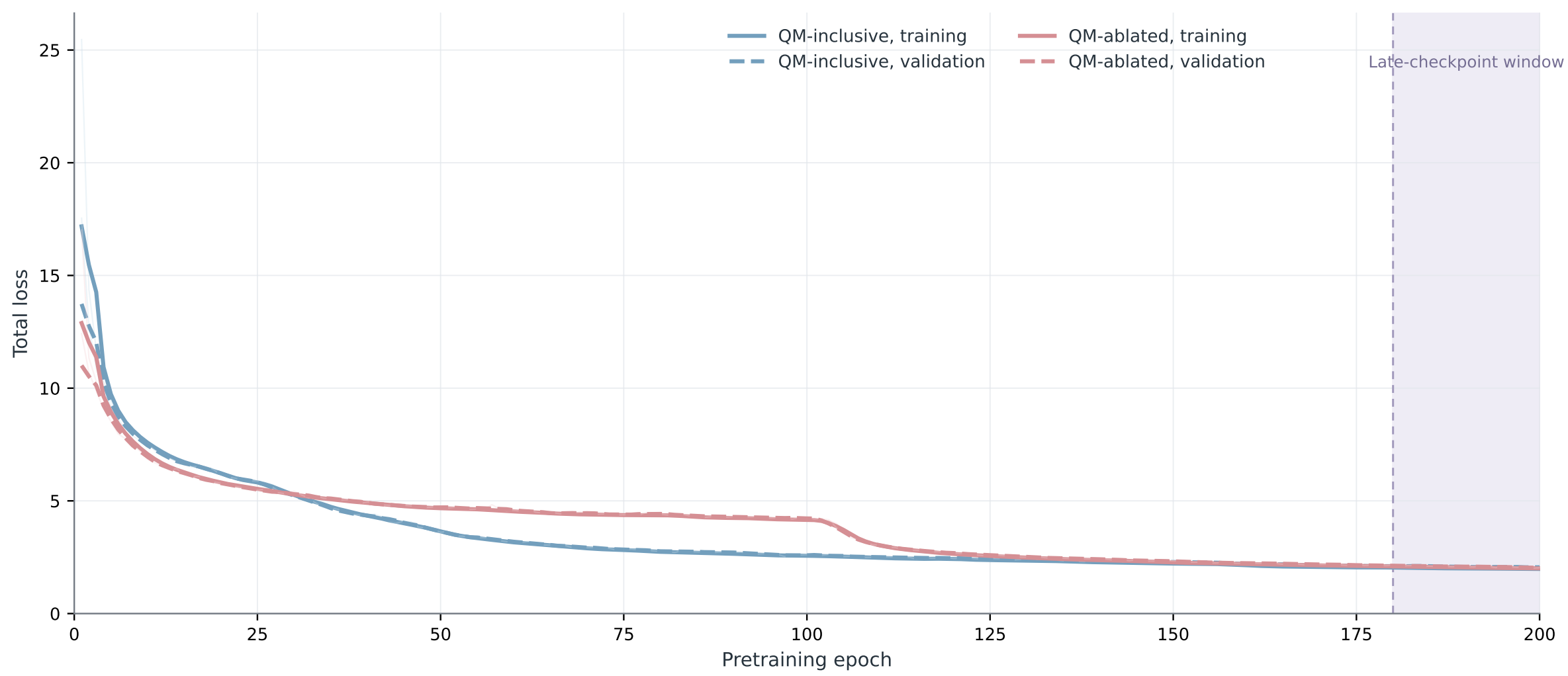

Supplementary Figure 19. Fully converged, capacity-matched pretraining ablation. Training and validation total loss per sample for the QM-informed and QM-ablated models over 200 epochs under the same protocol. The full linear vertical range is shown. Blue and pink distinguish the QM-informed and QM-ablated models, respectively; solid and dashed curves distinguish training and validation. Thin curves denote individual epochs and thick curves denote centered five-epoch means. The shaded region marks the prespecified epoch-180–200 late-checkpoint window.

We evaluated the checkpoints from epochs 180, 190, and 200, together with the best validation checkpoint, on 30 ADMET datasets under random and scaffold splits using five random seeds. Across 240 dataset–checkpoint–split comparisons, the QM-informed model performs better in 190 cases (79.2%). Detailed results are provided in Supplementary Figures 20–23.

The datasets were collected from MoleculeNet[25] and Therapeutics Data Commons[26]. These results show that, within the evaluated model and tasks, QM descriptors provide a consistent incremental contribution beyond molecular topology, three-dimensional geometry, and RDKit-derived features.

Overall, these experiments consistently highlight the essential role of quantum features in improving the downstream predictive power of qcGEM embeddings. Nevertheless, this conclusion is scope-limited and should not be overgeneralized. The benefits of QM descriptors are valid only under our tested qcGEM architecture and frozen-representation pipeline, and do not render QM features universally indispensable. Low-cost structural or approximate-quantum alternatives can still achieve competitive performance for certain tasks, reflecting the inherent accuracy-cost trade-off.

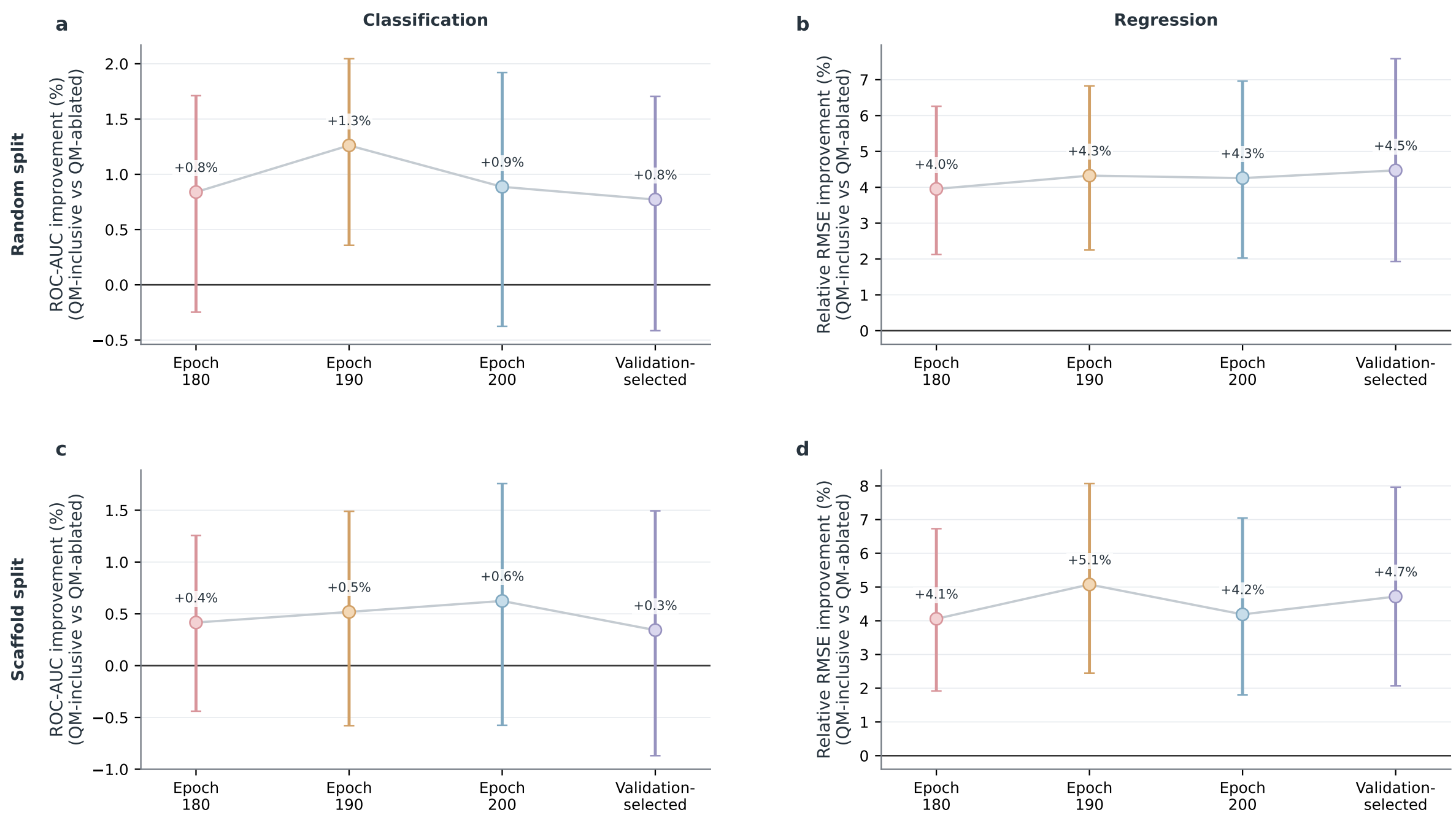

Supplementary Figure 20. Robustness of the QM contribution across late checkpoints and data splits. Panels **a)** and **b)** show classification and regression under the Random split; panels **c)** and **d** show the corresponding Scaffold-split results. ROC-AUC differences are multiplied by 100 and displayed as percentage-point improvements, whereas regression uses relative RMSE improvement (%). Positive values favor the QM-informed model. Points and numeric labels report cross-dataset means, error bars are dataset-bootstrap 95% confidence intervals, and the four pastel colors identify epochs 180, 190, and 200 and the validation-selected checkpoint.

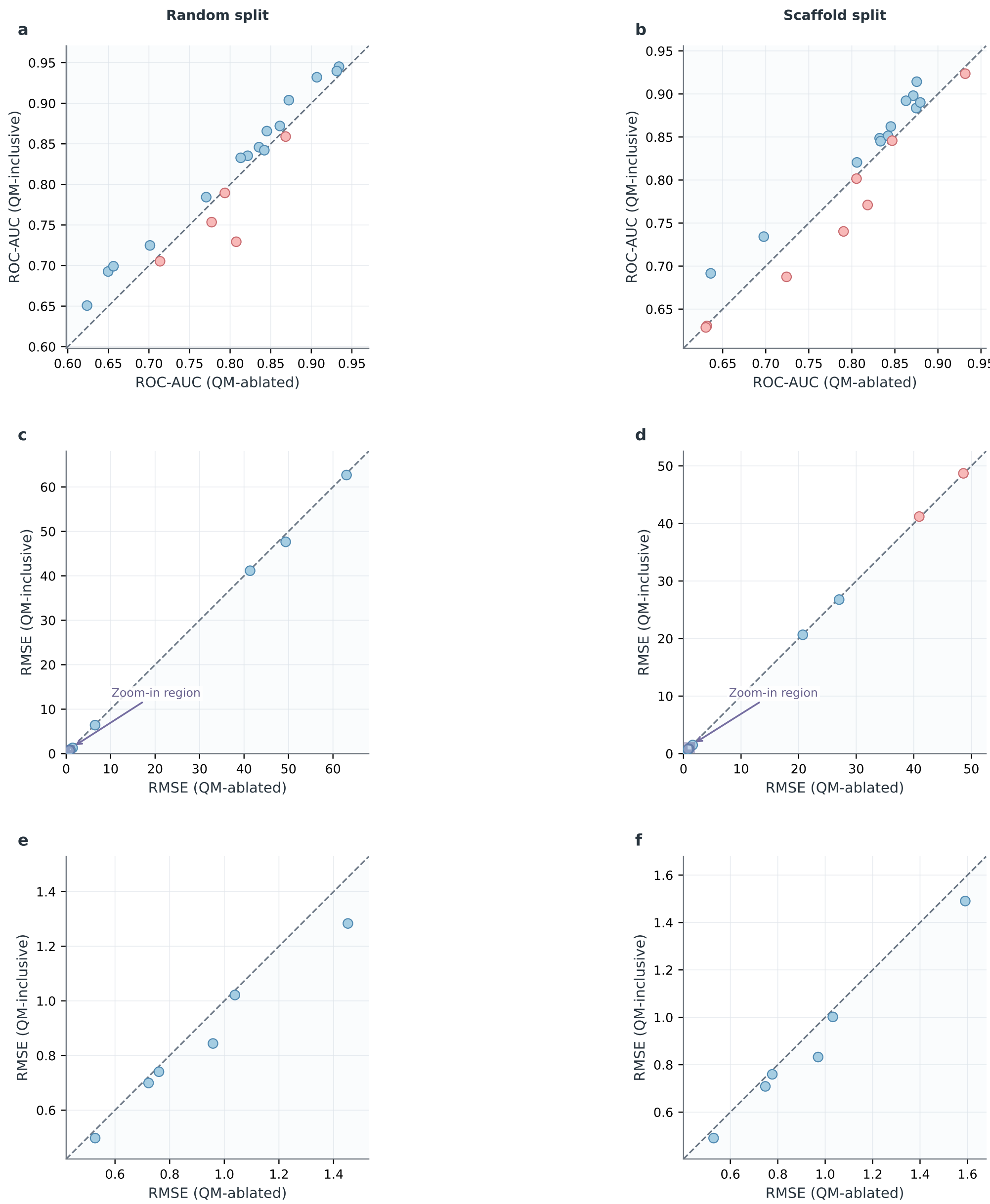

Supplementary Figure 21. Dataset-level paired comparison at epoch 200. Each point compares the five-seed mean performance of the QM-informed and QM-ablated models for one downstream dataset. Panels **a)** and **b)** show Random- and Scaffold-split classification ROC-AUC; panels **c)** and **d)** show the corresponding full-scale regression RMSE; panels **e)** and **f)** enlarge the low-RMSE regions marked by the lavender boxes and arrows in panels **c)** and **d)**. Blue points favor the QM-informed model, pink points favor the QM-ablated model, and the dashed identity line denotes equal performance.

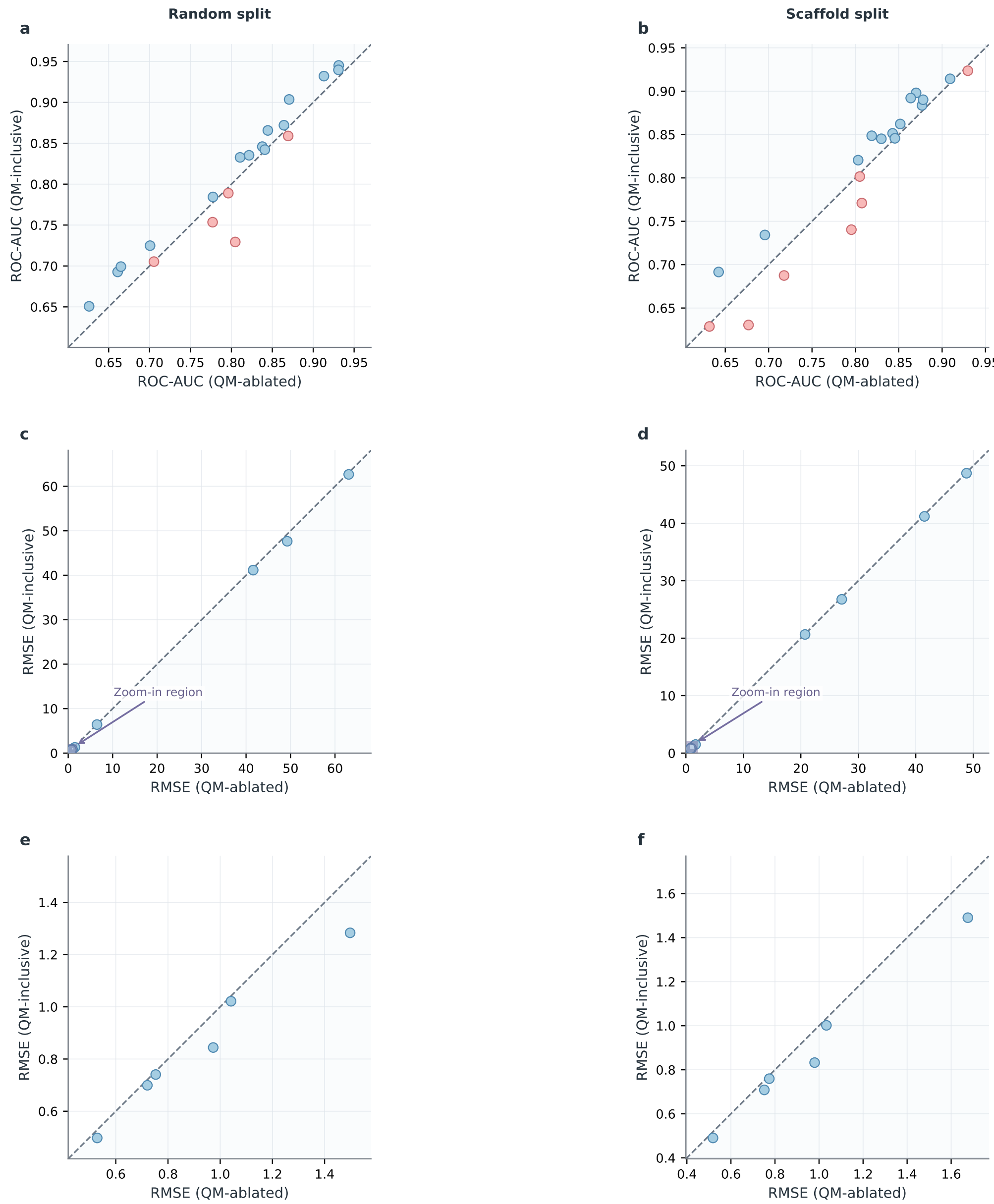

Supplementary Figure 22. Dataset-level paired comparison at the validation-selected checkpoint. Each point compares the five-seed mean performance of the QM-informed and QM-ablated models for one downstream dataset. Panels **a)** and **b)** show Random- and Scaffold-split classification ROC-AUC; panels **c)** and **d)** show the corresponding full-scale regression RMSE; panels **e)** and **f)** enlarge the low-RMSE regions marked by the lavender boxes and arrows above. Blue and pink points indicate datasets favoring the QM-informed and QM-ablated models, respectively, and the dashed line denotes equal performance. The checkpoint was selected using pretraining validation loss rather than downstream test performance.

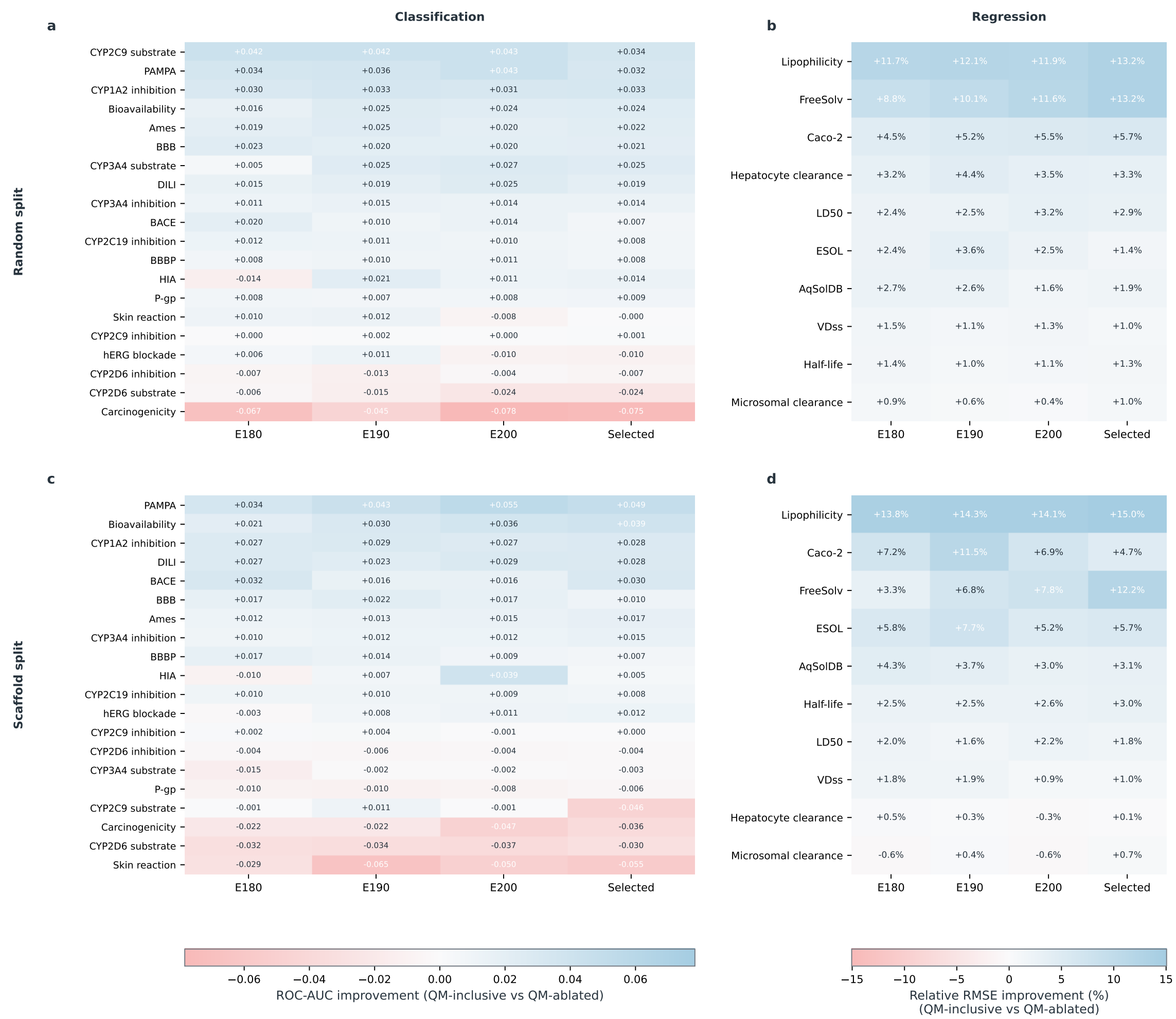

Supplementary Figure 23. Dataset-level consistency across late checkpoints. Panels **a)** and **b)** show Random-split classification and regression, and panels **c)** and **d)** show the corresponding Scaffold-split results. Cells report per-dataset ROC-AUC improvement for classification or relative RMSE improvement (%) for regression across epochs 180, 190, and 200 and the validation-selected checkpoint. Blue indicates positive improvement favoring the QM-informed model, pink indicates negative improvement, and near-white indicates little change. The heat map exposes task heterogeneity rather than reducing the evidence to a single aggregate score.

4.1.2 Analysis on the contribution of individual quantum descriptors

We examined the contribution of individual quantum descriptors on 30 activity cliff tasks (in the MoleculeACE dataset[27]). Specifically, we applied in-distribution perturbation upon each input feature of qcGEM, performed model prediction using the perturbed qcGEM embedding, and then quantified the importance of this feature as the loss of prediction power (*i.e.* ΔRMSE) across all 30 activity cliff tasks. Results are summarized in Supplementary Figure 24. Clearly, masking quantum mechanical (QM) features (*e.g.*, DI, ELF, NBO), either at the node or edge level, significantly undermines the prediction performance, demonstrating the primary contributions of these quantum descriptors in ensuring the downstream prediction power of qcGEM embeddings. Intriguingly, the top-contributing features are dominated by edge-level quantum descriptors, which encode chemical bonds and topological relationships between atoms and are therefore highly sensitive to the subtle electronic changes that determine the activity cliff. Notably, these features have been chronically missed in the previous molecular representation learning.

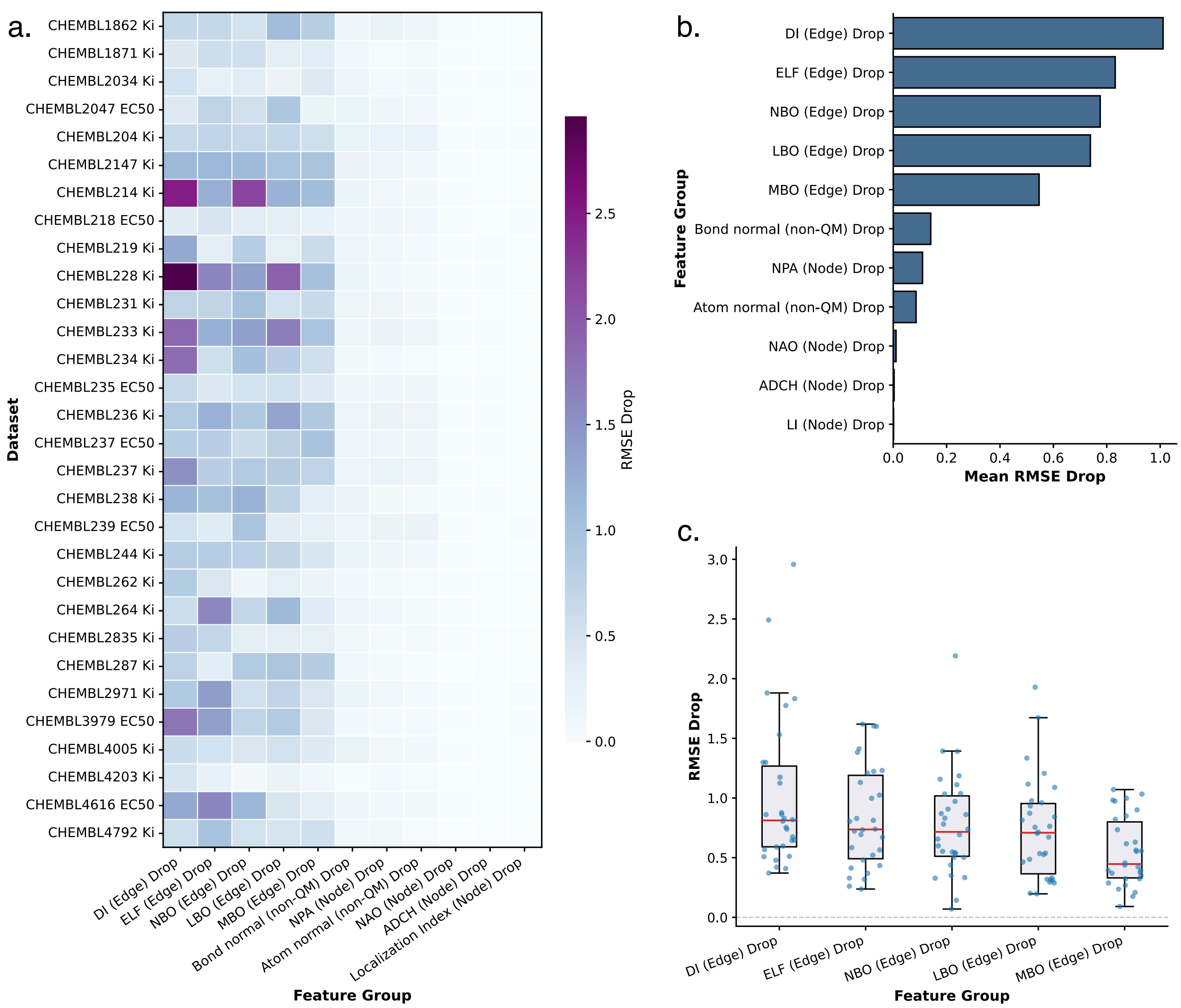

Supplementary Figure 24. Evaluation of qcGEM's feature importance in activity cliff tasks. a) Dataset-by-feature heatmap. Rows correspond to datasets and columns to features (or feature groups); color intensity encodes feature importance or effect strength within each dataset. **b)** Feature-importance bar plot. The horizontal axis quantifies the importance of features across the full dataset (by the increase in RMSE after excluding the corresponding feature), while the vertical axis lists top features (or feature groups) in descending order based on the numeric value of importance. **c)** Boxplots of the top-5 key features. This panel shows the distribution of the five most important global features across 30 sub-datasets.

Supplementary Figures 25 and 26 present case studies on specific activity cliff molecule pairs. Here, structural modifications are uniformly highlighted in pink in both 2D and 3D views, whereas atoms and bonds within the maximum common substructure (MCS) that have the most significant quantum feature discrepancies are marked in orange and green, respectively, to facilitate visualization. We find that the most pronounced electronic perturbations generally occur in close spatial proximity to the structural modification sites, from the viewpoints of both 2D topology and 3D conformation. Hence, qcGEM resolves divergent activities that arise from local chemical modifications, by successfully capturing these highly sensitive, spatially dependent electronic ripples.

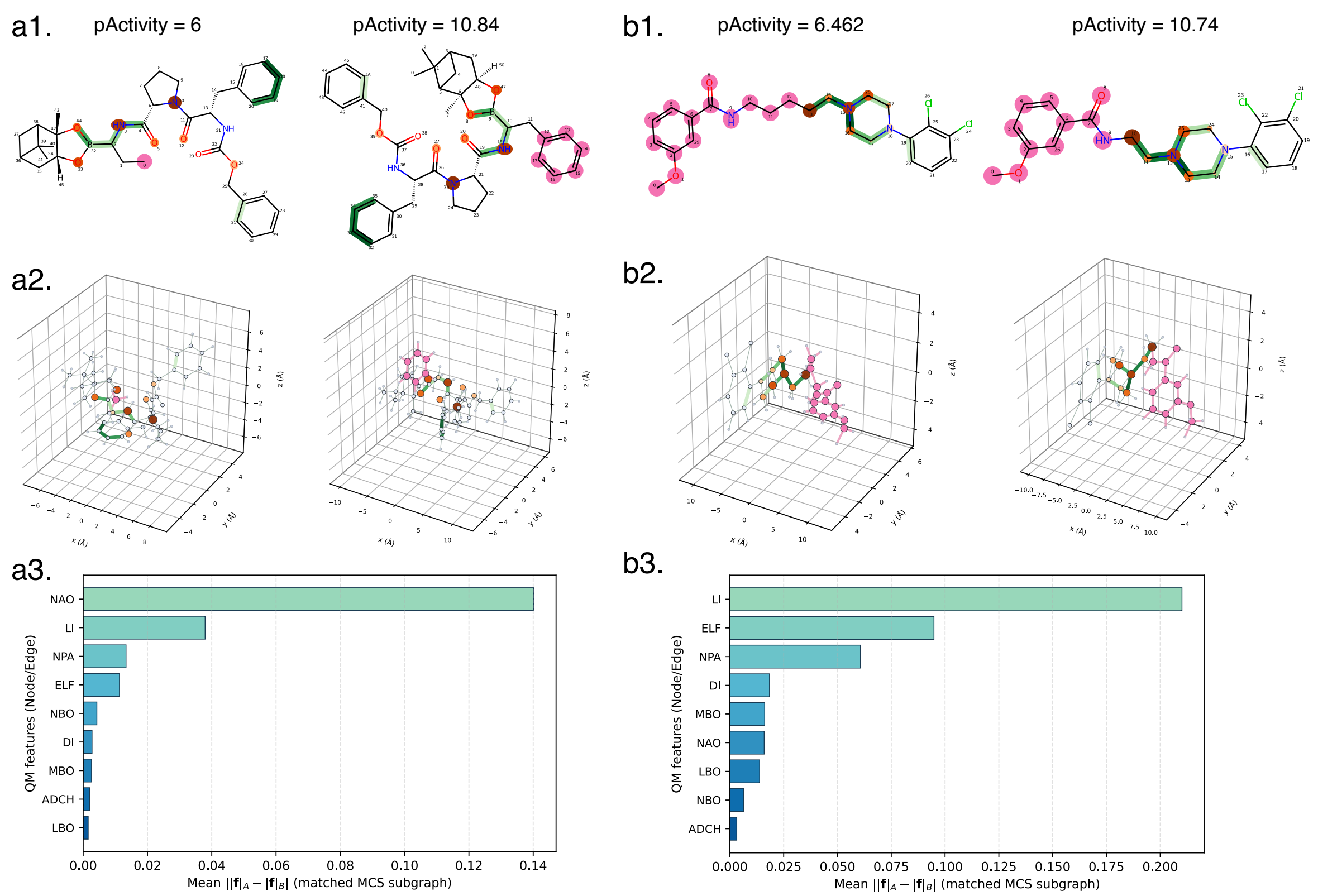

Supplementary Figure 25. Visualization of activity cliff molecules - Part I. Group-a and Group-b represent representative activity cliff molecule pairs targeting Thrombin (F2) and the Dopamine D4 receptor (D4), respectively. pActivity denotes the negative logarithm of the experimentally measured activity ($p\mathrm{K}_i$ or $p\mathrm{EC}_{50}$). **a1, b1)** 2D representations of two activity cliff molecule pairs. Structural differences between the paired molecules are highlighted in pink. Within the maximum common substructure (MCS), the top 8 atoms and bonds exhibiting the most significant quantum feature discrepancies are marked in orange and green, respectively, with color intensity corresponding to the magnitude of the difference. **a2, b2)** 3D spatial conformations of the paired molecules. The pink regions indicate local structural modifications, while the orange and green markers denote the atoms and bonds within the conserved substructure that undergo the largest feature variations. **a3, b3)** Quantitative comparison of feature deviations within the conserved MCS regions. The bar charts illustrate the mean absolute differences of various node/edge-level quantum mechanical (QM) descriptors between the paired molecules.

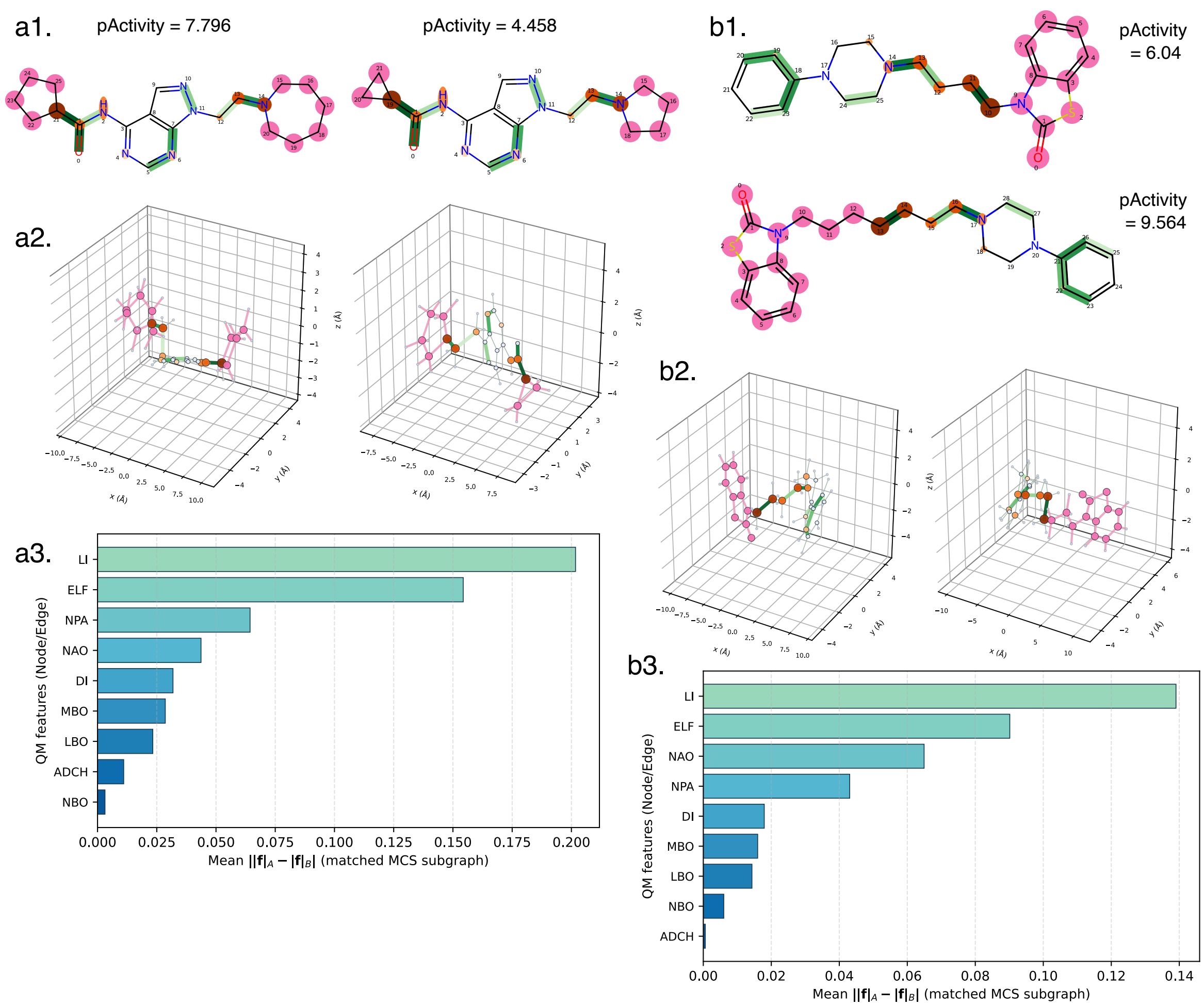

Supplementary Figure 26. Visualization of activity cliff molecules - Part II. Group-a and Group-b represent representative activity cliff molecule pairs targeting Sigma opioid receptor (SOR) and the Serotonin 1a receptor (5-HT1A), respectively. pActivity denotes the negative logarithm of the experimentally measured activity (pK_i or pEC_{50}). **a1, b1)** 2D representations of two activity cliff molecule pairs. Structural differences between the paired molecules are highlighted in pink. Within the maximum common substructure (MCS), the top 8 atoms and bonds exhibiting the most significant quantum feature discrepancies are marked in orange and green, respectively, with color intensity corresponding to the magnitude of the difference. **a2, b2)** 3D spatial conformations of the paired molecules. The pink regions indicate local structural modifications, while the orange and green markers denote the atoms and bonds within the conserved substructure that undergo the largest feature variations. **a3, b3)** Quantitative comparison of feature deviations within the conserved MCS regions. The bar charts illustrate the mean absolute differences of various node/edge-level quantum mechanical (QM) descriptors between the paired molecules.

4.1.3 About the performance difference between regression and classification tasks

In this section, we investigate the reason behind qcGEM's diminished advantage over other baselines in the classification tasks of molecular property prediction, compared to regression tasks (Figure 2b&c). For this aim, we systematically evaluated the sensitivity of these two types of tasks to the precision of quantum features. Specifically, we applied perturbations of varying degrees—precision truncation (5 levels, from 0 to 4) and Gaussian noise (five σ values, from 0 to 0.2)—to quantum features, and quantified the corresponding performance degradation as well as the slope of such degradation over perturbation level. This experiment was conducted across 3 regression (ESOL, FreeSolv and Lipophilicity) and 3 classification (BBBP, BACE and DILI) benchmarks in MoleculeNet[25], with each individual evaluation repeated over 5 random seeds across both random and scaffold splits. In total, this comprehensive analysis involved 600 independent experimental runs.

Here, we briefly introduce the evaluation metrics in this experiment. The "relative performance" is defined as $\text{RMSE}_{\text{perturbed}}/\text{RMSE}_{\text{baseline}}$ for regression and $\text{ROC-AUC}_{\text{baseline}}/\text{ROC-AUC}_{\text{perturbed}}$ for classification, respectively, with the value of > 1 indicating performance degradation. The "sensitivity slope" is the linear fit of relative performance against perturbation intensity. Cohen's d is used to quantify the significance of gap between regression and classification tasks.

All results are presented in Supplementary Figure 27. The sensitivity plots suggest that the model performance degrades more rapidly with increasing perturbation intensity in the regression tasks than in the classification tasks (Supplementary Figure 27a). The non-trivial Cohen's d values in all experimental settings confirm the statistical significance of the difference in sensitivity slope between regression and classification tasks (Supplementary Figure 27b). Moreover, the difference between the sensitivity slopes of regression and classification tasks is persistently positive across all settings (Supplementary Figure 27c), and the pattern is highly consistent across individual tasks (Supplementary Figure 27d).

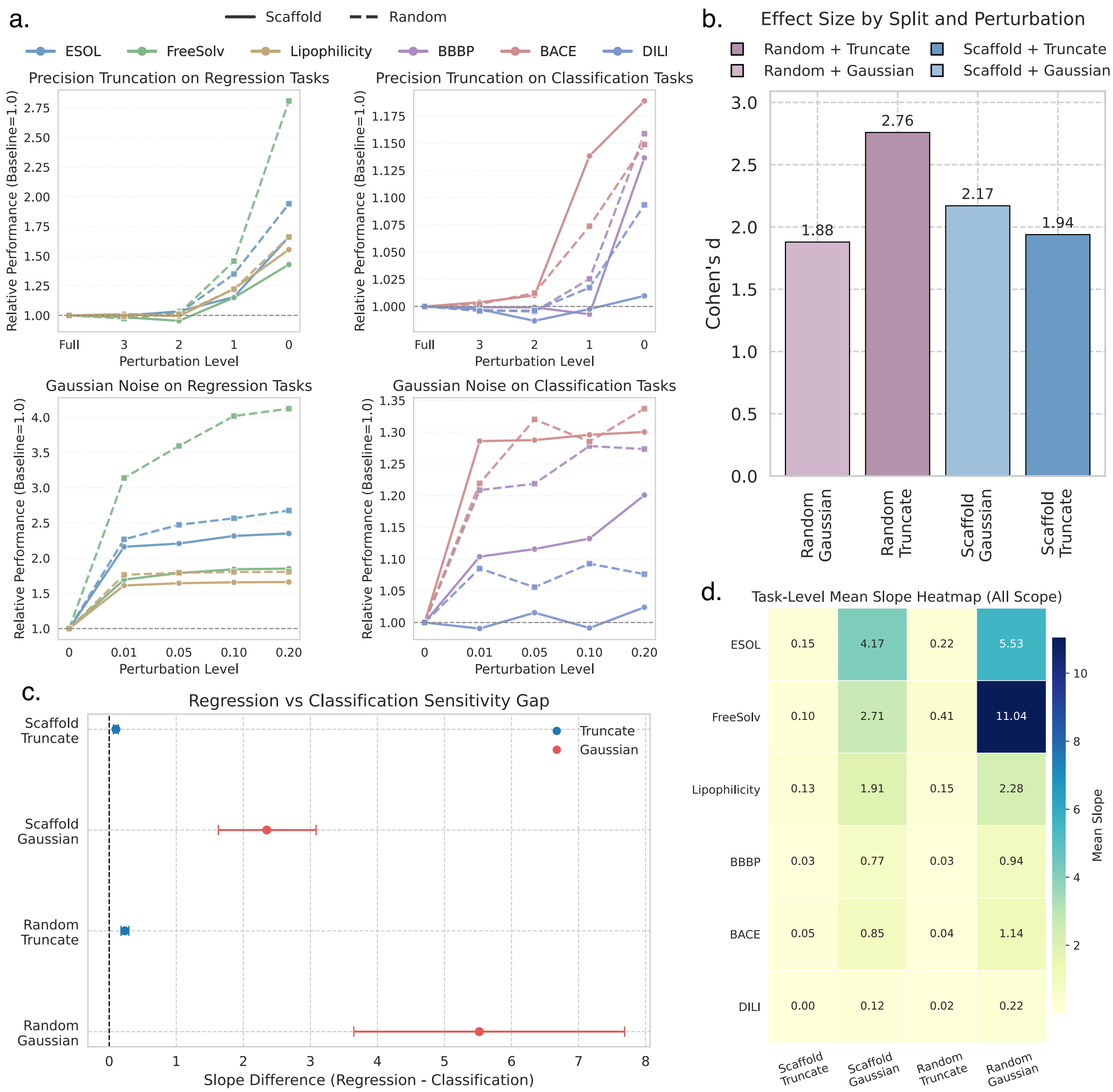

Supplementary Figure 27. Sensitivity analysis of qcGEM performance to perturbations on the quantum chemical features across regression and classification tasks. a) Relative performance degradation curves for regression (ESOL, FreeSolv and Lipophilicity) and classification (BBBP, BACE and DILI) tasks under varying levels of precision truncation (top) and Gaussian noise (bottom). A relative performance value of > 1.0 indicates degradation compared to the unperturbed baseline. Solid and dashed lines denote scaffold and random data splits, respectively. **b)** Standardized effect size (Cohen's d) of the regression–classification sensitivity gap across different perturbation types and split protocols, confirming the significance of differences between regression and classification tasks. **c)** Forest plot illustrating the difference in sensitivity slope between regression and classification tasks (Regression - Classification). Consistent positive values for the overall 95% confidence intervals (denoted by bars) demonstrate that regression tasks are significantly more sensitive to external perturbations. **d)** Heatmap of task-level mean sensitivity slopes. The consistently higher slope values for regression tasks confirm that the elevated sensitivity to feature precision is a robust task-type characteristic rather than a single-dataset artifact.

Subsequently, we specifically screened for a "BACEreg" benchmark (Supplementary Table 6), by inherently retaining the raw continuous activity values in the BACE dataset, which is originally designed for classification tasks and thus generally reports categorical information through data discretization. Under strictly identical data processing and splits, we conducted a rigorous head-to-head comparison of classification vs. regression in the same benchmark. Results in Supplementary Figure 28 reveal that qcGEM's comparative advantage significantly amplifies in the complex regression setting of the same task. Conversely, classification-strong baselines like GROVER suffer severe performance degradation in the harder regression task. This confirms that the current classification-dominant ADMET benchmarks objectively eclipse qcGEM's true potential, which lies inherently in high-precision regression scenarios.

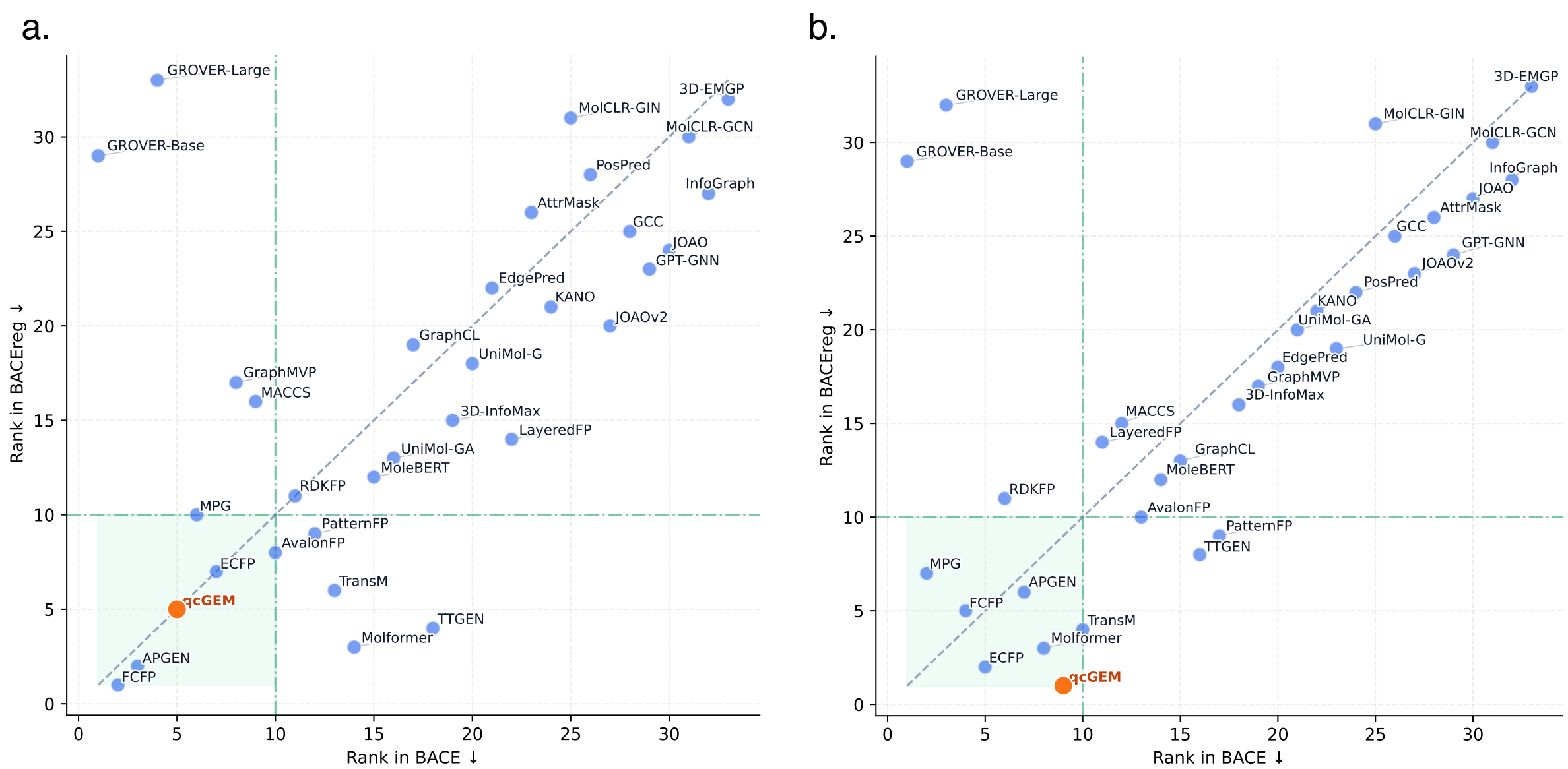

Supplementary Figure 28. Performance ranking comparison between classification and regression tasks for various models on the BACE dataset. a) Results under scaffold split. **b)** Results under random split. The horizontal and vertical axes represent performance ranks (lower is better) in the BACE classification task and regression task, respectively. Orange: qcGEM; Blue: baselines; Green shaded area: top-10 region. qcGEM consistently falls below the $y = x$ diagonal, confirming its strong comparative advantage in regression tasks.

4.2 Analysis of precision, time and performance

4.2.1 End-to-end runtime analysis

A key concern raised regarding the practicality of quantum-chemistry-based molecular property prediction is the computational overhead introduced by density functional theory (DFT) calculations relative to purely data-driven geometric models. To quantify this overhead transparently, we benchmarked the two production variants of the qcGEM pipeline: qcGEM and qcGEM-Hybrid. The two variants share three sequential stages: (i) semi-empirical geometry pre-optimization with GFN2-xTB; (ii) DFT calculation with ORCA at the B3LYP-D3/def2-SV(P) level; and (iii) wave-function analysis with NBO and Multiwfn. Their difference, however, only lies in stage (ii): the standard qcGEM pipeline (OPT) performs a full B3LYP-D3/def2-SV(P) geometry optimization, whereas the qcGEM-Hybrid pipeline (SP) replaces this with a B3LYP-D3/def2-SV(P)//GFN2-xTB single-point calculation on the GFN2-xTB-optimized geometry, bypassing the expensive iterative DFT relaxation.

We conducted a systematic end-to-end wall-clock timing analysis across $n = 378$ structurally diverse molecules (atom counts ranging from 5 to 348), benchmarking the two production variants of the qcGEM pipeline on a single CPU core.

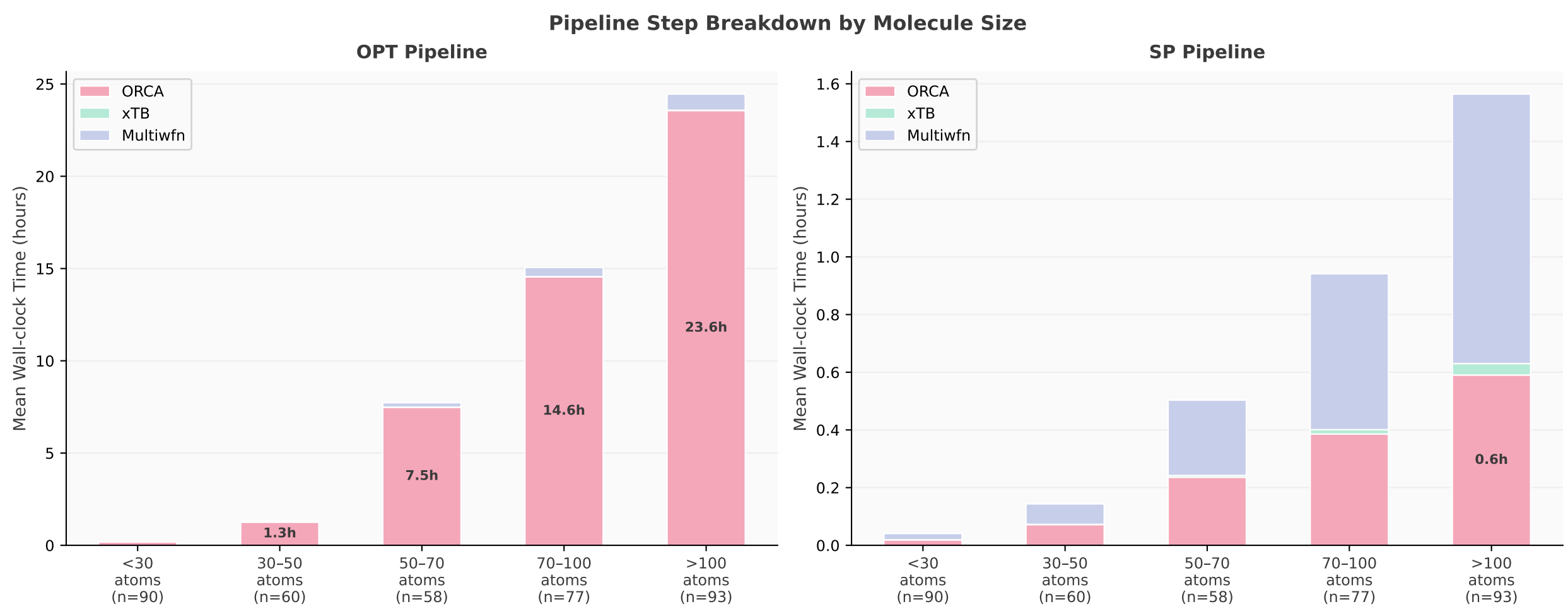

Supplementary Figure 29. Step-level wall-clock time breakdown by molecular size. Mean per-molecule computation time (hours) for the OPT (left) and SP (right) pipelines, decomposed into three steps (ORCA DFT, xTB semi-empirical, and Multiwfn feature extraction) and stratified by atom count bin. Sample sizes per bin are indicated below the horizontal axis. ORCA dominates the OPT pipeline ($\geq 95\%$ of total cost), whereas the SP pipeline distributes cost more evenly across all three steps.

Supplementary Figure 29 decomposes the mean per-molecule wall-clock time into its three constituent steps as a function of molecular size (atom-count bin). In the OPT pipeline, the ORCA B3LYP-D3/def2-SV(P) geometry optimization dominates overwhelmingly ($\geq 95\%$ of total time at median), growing from a mean of 0.20 h for molecules with fewer than 30 atoms to approximately 23.6 h for those with more than 100 atoms. The GFN2-xTB pre-optimization and Multiwfn/NBO feature extraction together contribute less than 5% of total OPT cost across all size classes. In the SP pipeline, the cost profile is markedly different: the ORCA single-point calculation accounts for a median of only 43.4% of the total runtime, while the GFN2-xTB and Multiwfn steps—both lightweight and largely size-insensitive—constitute the majority of the wall-clock time. As a result, mean SP total time grows much more slowly, from 0.041 h (<30 atoms) to 1.564 h (>100 atoms), representing a $10.8\times$ median reduction in per-molecule cost relative to the OPT pipeline (mean ratio $12.2\times$; maximum $55.9\times$ for the largest molecules). This is consistent with the efficiency gains reported for qcGEM-Hybrid in the main text (Figure 5a), where the power-law scaling exponent decreases from 2.58

(OPT) to 1.76 (SP) and the per-molecule DFT time for a 100-atom molecule drops from 18 hours to 28 minutes on a single CPU core.

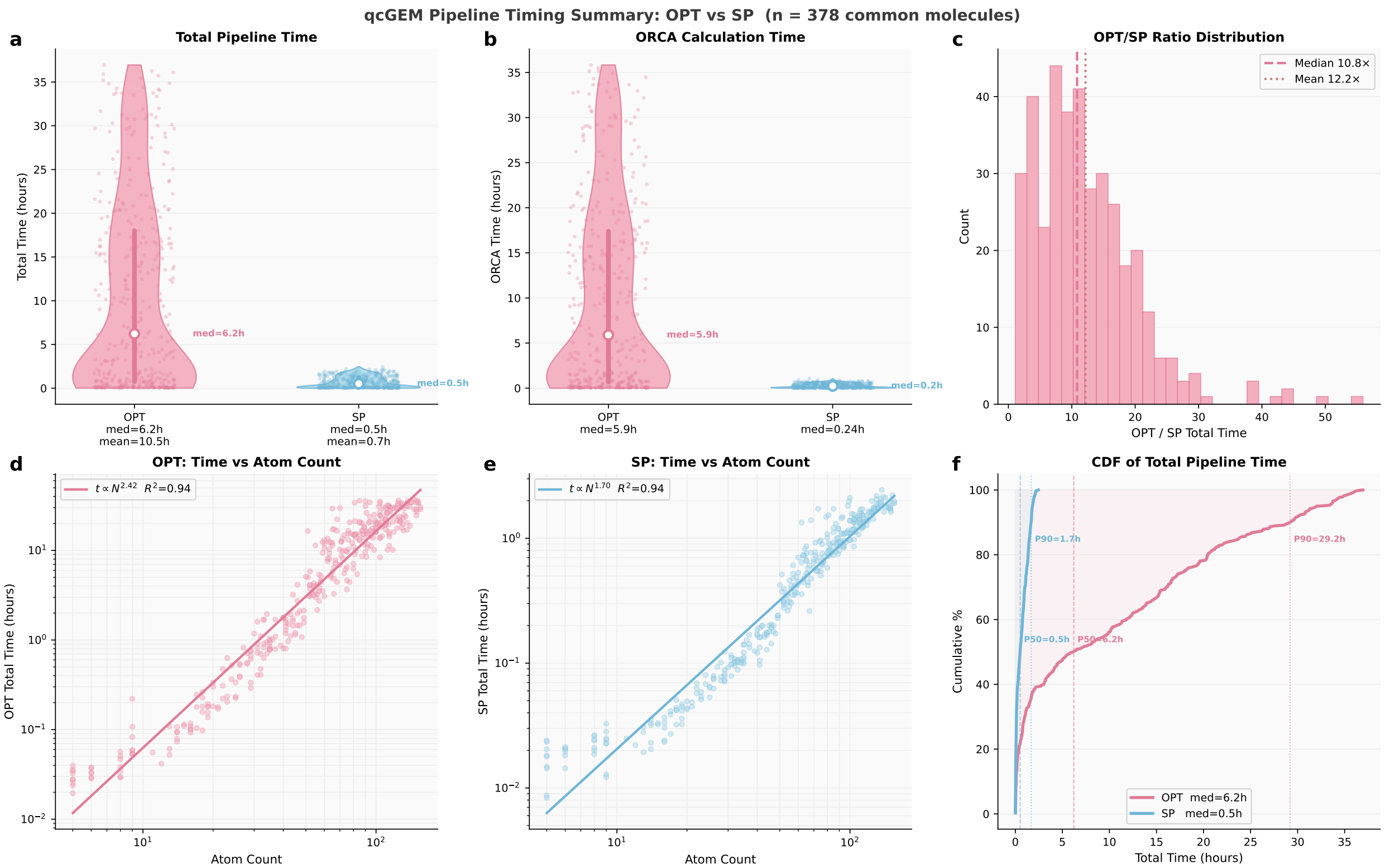

Supplementary Figure 30. Comprehensive timing summary for the qcGEM OPT and SP pipelines ($n = 378$ molecules). **(a)** Violin + strip plot of end-to-end total pipeline time. **(b)** Violin + strip plot of ORCA-only DFT calculation time. **(c)** Distribution of the OPT/SP total-time ratio. **(d, e)** Double logarithmic-scale scatter of total runtime vs. atom count for OPT and SP pipelines, respectively, with power-law fits. **(f)** Cumulative distribution of total pipeline time. Vertical dashed and dotted lines mark the 50th and 90th percentiles, respectively. Horizontal tick labels in panels (**a**) and (**b**) include median values for direct comparison.

Supplementary Figure 30 provides a comprehensive statistical characterization of the end-to-end per-molecule runtime. The violin plots (Supplementary Figure 30a) show that the OPT pipeline has a median total wall-clock time of 6.21 h (mean: 10.53 h), with a long right tail driven by large molecules (the 90th percentile: 29.20 h). By contrast, the SP pipeline achieves a median of $\mathbf{0.52}$ h (mean: 0.69 h), with 90% of molecules completed within $\mathbf{1.68}$ h and 95% within 1.83 h. The ORCA-only contribution (Supplementary Figure 30b) mirrors this pattern: full DFT geometry optimization requires a median of 5.94 h, whereas the B3LYP-D3/def2-SV(P)//GFN2-xTB single-point run requires only 0.24 h — an approximately 25-fold reduction attributable solely to eliminating the iterative DFT geometry relaxation. Acceleration by the SP pipeline is more clearly shown in the histogram of evaluation results on identical molecules (Supplementary Figure 30c). The cumulative distribution (Supplementary Figure 30f) further illustrates that virtually all SP computations complete within 2 h, making qcGEM-Hybrid practically feasible for medium-throughput screening campaigns involving thousands of molecules.

Admittedly, the per-molecule costs reported here (0.52 h median for SP, on a single CPU core) are not competitive with the millisecond-scale inference of end-to-end geometric deep-learning models such as SchNet, DimeNet++, or PaiNN. However, we emphasize that qcGEM targets a complementary use case: it is designed for focused chemical libraries where quantum chemical accuracy and physical interpretability are required, and where a dedicated DFT

campaign is already accepted as standard practice. Critically, the qcGEM-Hybrid variant achieves a favourable trade-off by replacing the full B3LYP-D3/def2-SV(P) geometry optimization with a single-point calculation on a GFN2-xTB geometry (B3LYP-D3/def2-SV(P)//GFN2-xTB), preserving predictive accuracy while reducing per-molecule DFT cost by more than an order of magnitude.

Furthermore, representations generated by qcGEM and qcGEM-Hybrid are reusable: once computed and stored, embeddings can be applied directly to any downstream task without repeating the quantum chemistry calculation, effectively amortising the one-time cost across all subsequent uses. Combined with the support for multi-core acceleration, which reduces the 100-atom SP calculation to within 2 minutes on 16 CPU cores (Figure 5b), this strategy enables practical, scalable deployment in large-scale screening scenarios.

4.2.2 Trade-off between performance and cost

We performed the performance–cost analysis for a number of molecular representations on the MoleculeACE dataset[27], including qcGEM/qcGEM-Hybrid as well as ECFP (1D fingerprint), MolCLR (2D graph) and UniMol (3D graph).

Supplementary Figure 31a compares the relative time consumption (normalized by ECFP) and the prediction error (in RMSE) of downstream probe network in activity cliff tasks. Clearly, qcGEM series achieve the best performance (*i.e.* the lowest RMSE). Benefiting from pre-training denoising and mask-replace tasks, our model robustly tolerates minor feature noise, yielding nearly negligible performance differences between qcGEM and qcGEM-Hybrid, in consistent with evaluation results in Figure 5c. Despite the substantial speedup with negligible performance loss, qcGEM-Hybrid is still computationally intensive, slower than the fastest method ECFP by about 7 orders of magnitude. However, through parallel computing on 64 CPU cores, qcGEM-Hybrid acquires an additional acceleration of nearly 2 orders of magnitude, reaching a comparable running time with the popular representation UniMol.

Supplementary Figure 31b presents the performance–cost analysis on specific quantum chemical features, where the acquisition time and the feature importance (*i.e.* performance degradation caused by injection of distribution-matched noise) are evaluated for each quantum descriptor. Our results show that DI, NBO, MBO and NAO reside on the Pareto frontier, representing an optimal balance between low computational cost and high importance to performance. In contrast, ELF significantly influences performance but accounts for the majority of the feature acquisition time.

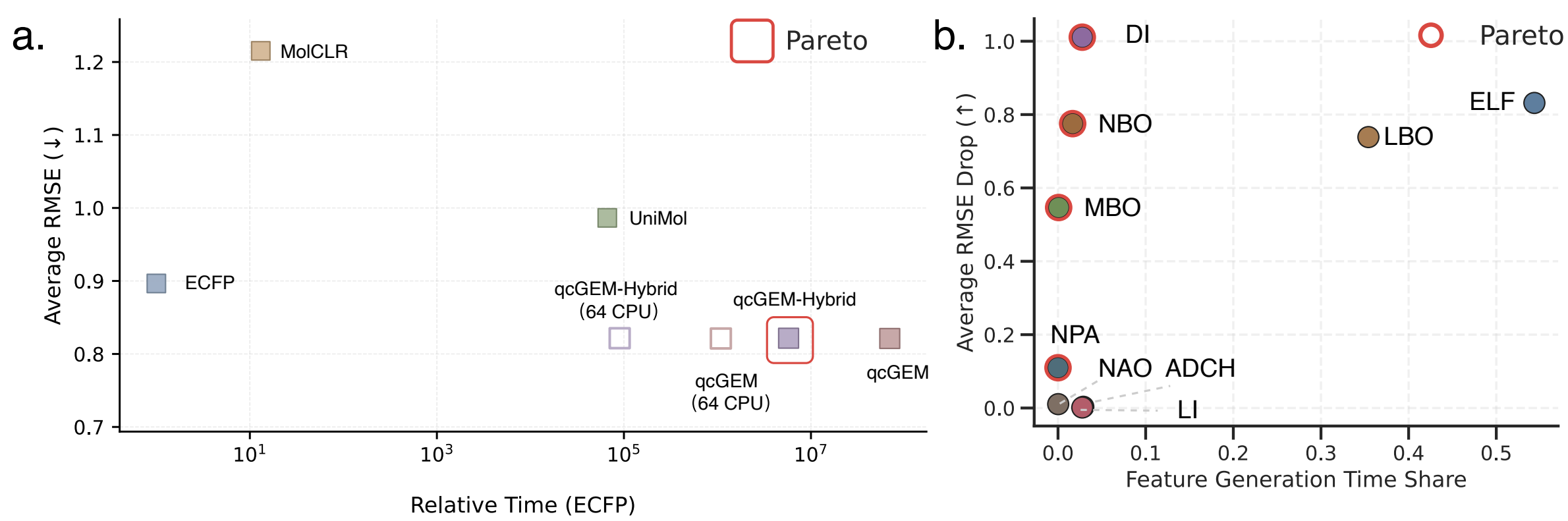

Supplementary Figure 31. Performance–cost analysis of qcGEM. a) The comparison of classical model performance vs. computational cost, where the vertical axis is the predictive error (RMSE; lower is better) and the horizontal axis reports relative representation-generation time (logarithmic scale, normalized to ECFP). Red circles indicate Pareto-optimal candidates under the dual objective of lower error and lower cost. **b)** The comparison of quantum chemical features performance vs. computational cost, where the vertical axis is the average increase in RMSE after permutation ablation (higher indicates stronger contribution to model performance) and the horizontal axis reports feature-generation time share in the post-analysis pipeline. Red circles denote Pareto-front features that provide the best performance–cost balance among all candidates.

Subsequently, we conducted a detailed comparison between qcGEM-Hybrid and qcGEM. In the corrrelation analysis shown in Supplementary Figure 32a, the molecule-wise prediction errors (in RMSE) of two pipelines present a strong correlation, with the Spearman correlation coefficient ρ reaching 0.962. Moreover, the two pipelines display nearly identical prediction errors in 16 out of the 20 hardest targets. We then performed a granular gap analysis to identify specific features where qcGEM-Hybrid significantly underperforms the original qcGEM. Supplementary Figure 32b indicates that the most robust negative signals are concentrated in charged molecules ($|\text{formal charge}| \geq 1$), where the Hybrid version exhibits a significantly higher prediction error. In contrast, highly conjugated compounds and systems with complex intramolecular hydrogen bonding do not show significant degradation after applying multiple testing

corrections. Regarding metal complexes, as the current benchmark contains no such samples ($n = 0$), this dimension remains untestable on the current dataset.

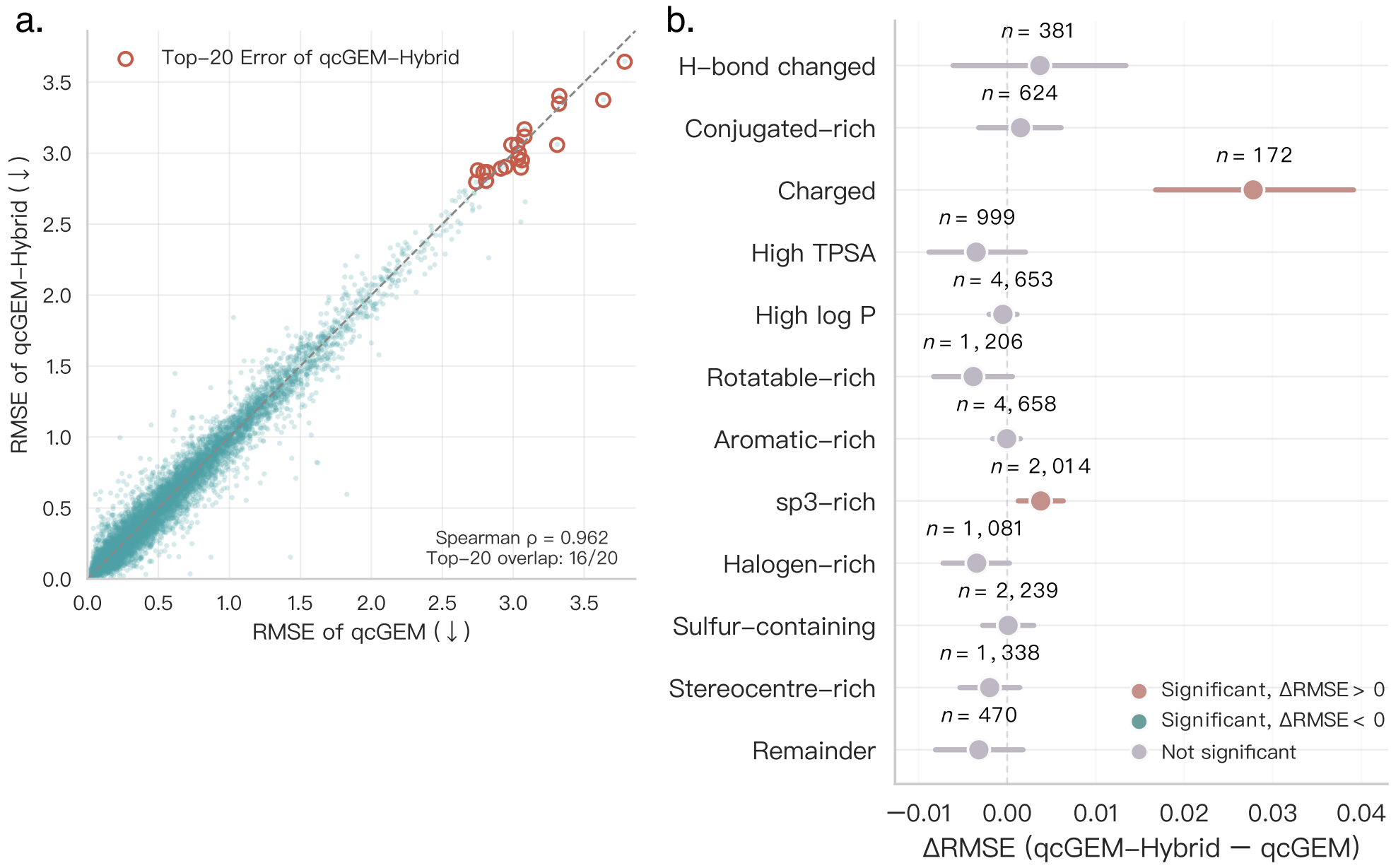

Supplementary Figure 32. Correlation and subgroup shifting between qcGEM and qcGEM-Hybrid. a) Molecule-wise RMSE correlation ($\rho = 0.962$), highlighting that the Hybrid version preserves the intrinsic error landscape and shares 16/20 of the hardest test cases (red circles). **b)** Statistical analysis of ΔRMSE (*i.e.* $\mathrm{RMSE}_{\mathrm{qcGEM\text{-}Hybrid}} - \mathrm{RMSE}_{\mathrm{qcGEM}}$) across molecular sub-categories. Significant performance degradation is primarily isolated to "Charged" molecules.

4.3 Effect of conformers

We designed a comprehensive multi-conformer evaluation protocol and conducted extensive experiments across 6 MoleculeNet[25] benchmark datasets: ESOL, FreeSolv, Lipophilicity, BBBP, BACE, and ClinTox.

For each molecule, we sourced five distinct conformers from the GEOM dataset[28], which provides CREST-generated low-energy conformer ensembles for a large collection of drug-like molecules. Specifically, we selected all molecules in the MoleculeNet benchmarks that had at least five conformers available in GEOM, retaining the five lowest-energy conformers per molecule (denoted Rank 0 through Rank 4 in the order of ascending energy values). All conformers were subsequently processed through the identical B3LYP-D3/def2-SV(P) geometry optimization and wave function post-analysis pipeline, ensuring that the multi-conformer geometries are fully consistent with the training data distribution of qcGEM. We then evaluated qcGEM's robustness from two complementary perspectives: embedding-level consistency and downstream prediction consistency.

4.3.1 Embedding-level consistency

We first examined whether different optimized conformers of the same molecule produce similar representations in qcGEM's learned embedding space. For each molecule, we extracted the 384-dimensional embedding vector (128D global + 128D node-mean + 128D edge-mean) from the frozen pre-trained encoder for all five conformers and computed pairwise cosine similarities between Rank-0 and Rank-1/2/3/4.

As shown in Supplementary Figure 33, the full 384D embeddings exhibit high cosine similarity across all six datasets, with the mean value ranging from 0.918 ± 0.045 (ESOL) to 0.955 ± 0.031 (BACE). Across all datasets, 70.1%–94.6% of conformer pairs achieve cosine similarity ≥ 0.90, with 26.3%–66.4% exceeding 0.95.

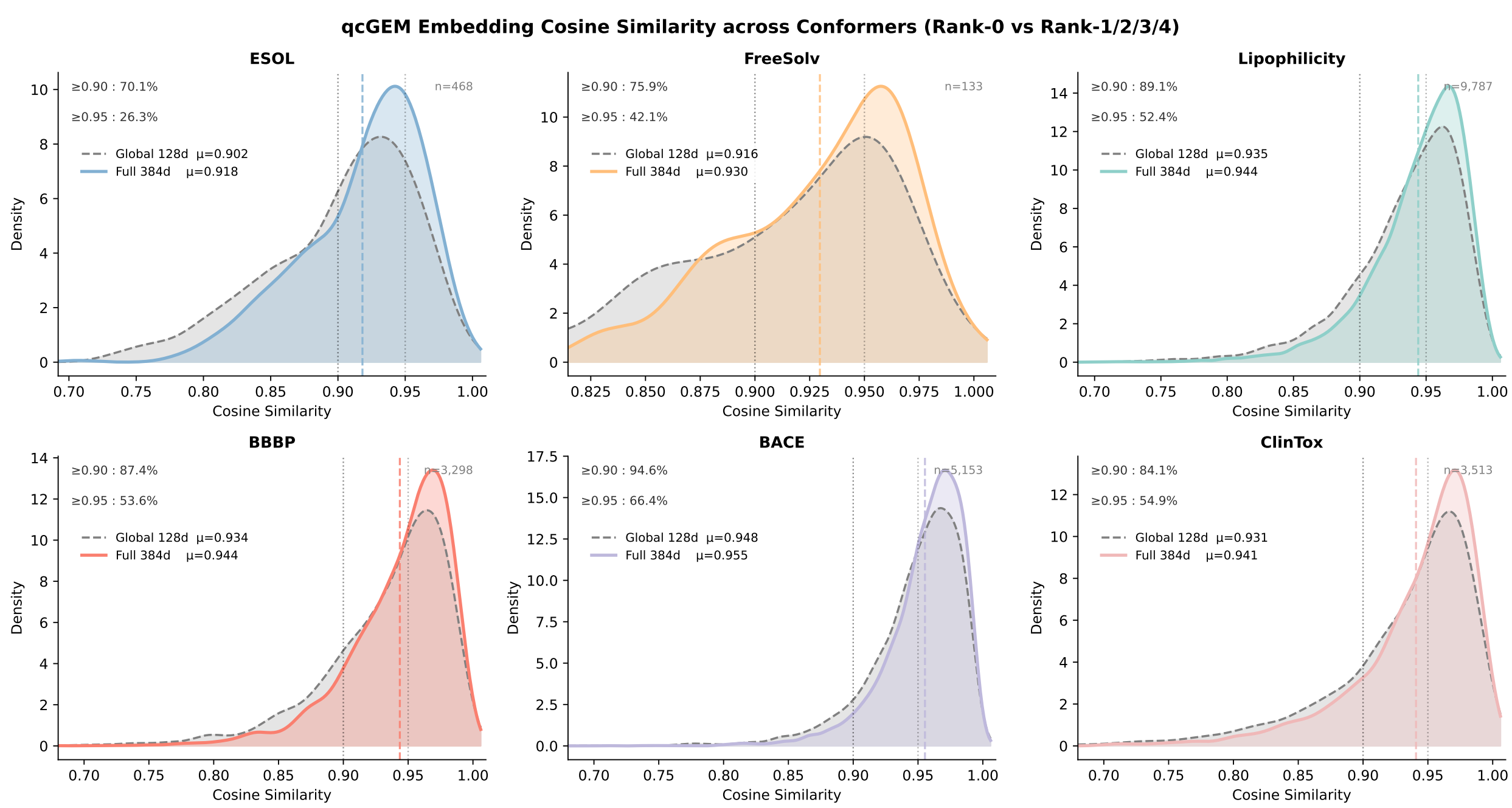

Supplementary Figure 33. Embedding cosine similarity across conformers. Kernel density estimation (KDE) of pairwise cosine similarity between Rank-0 and Rank-1/2/3/4 embeddings for each molecule, shown for the global 128D embedding (dashed) and the full 384D embedding (solid). The mean full-384D similarity ranges from 0.918 (ESOL) to 0.955 (BACE), with 70–95% of pairs exceeding 0.90.

To provide intuitive visualization, we performed t-SNE dimensionality reduction on the 384D embeddings. As shown in Supplementary Figure 34, conformers belonging to the same molecule (connected by lines to their centroids, with ★ indicating Rank-0) form extremely tight clusters, while different molecules are well separated. The cross-dataset t-SNE analysis (Supplementary Figure 35) further quantifies this observation: the average intra-molecule conformer spread is only 0.72 in the t-SNE space, whereas the average inter-molecule centroid distance is 41.27, yielding a separation ratio of 57×. Hence, the embedding variation caused by conformational differences is approximately two orders of magnitude smaller than the variation between distinct molecules, confirming that qcGEM captures molecular identity.

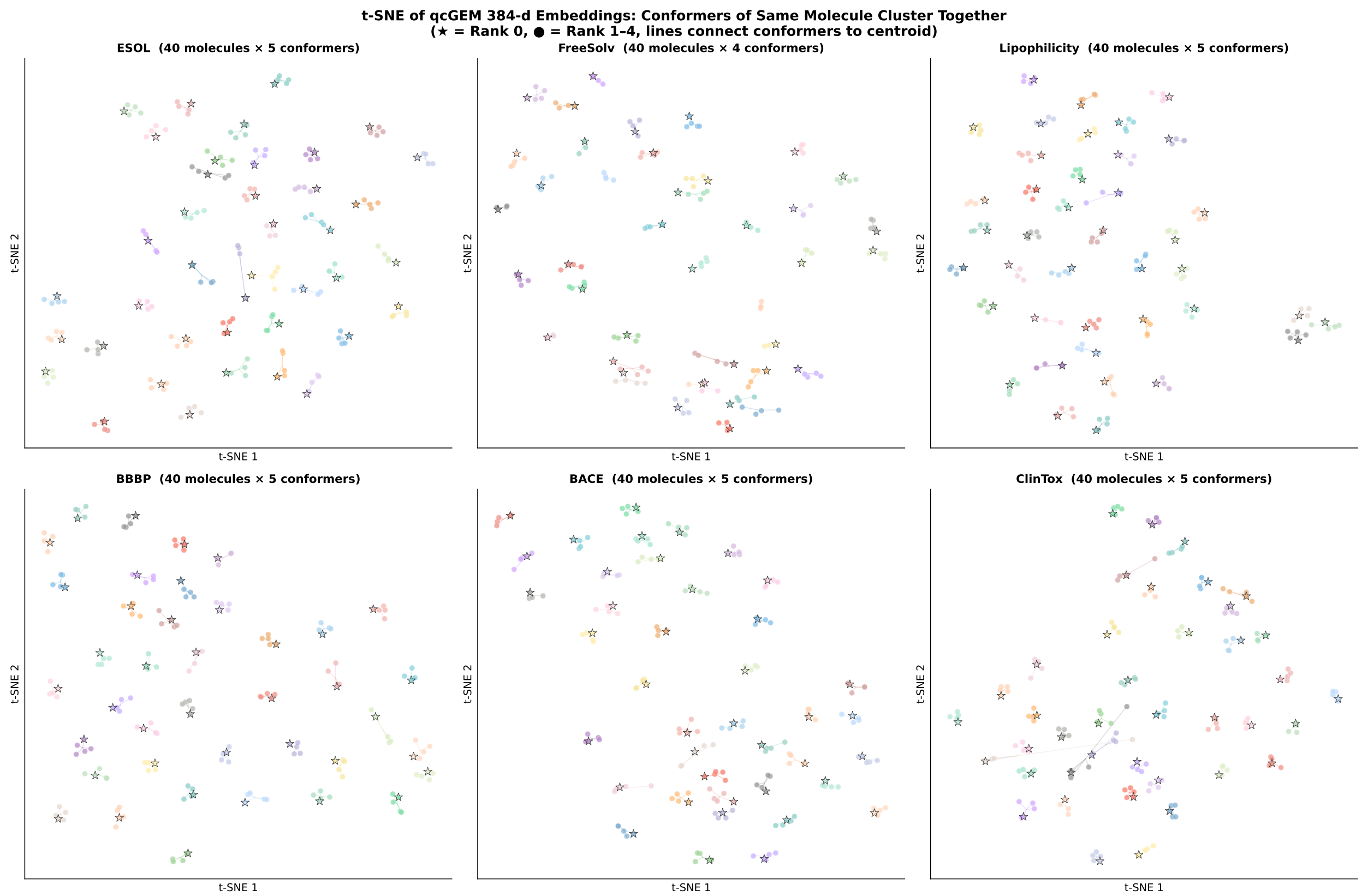

Supplementary Figure 34. t-SNE visualization of conformer embeddings per dataset. Each color represents a distinct molecule; ★ indicates the Rank-0 conformer and • indicates Rank-1–4. Lines connect conformers to their molecular centroid. Conformers of the same molecule cluster tightly, while different molecules are well separated.

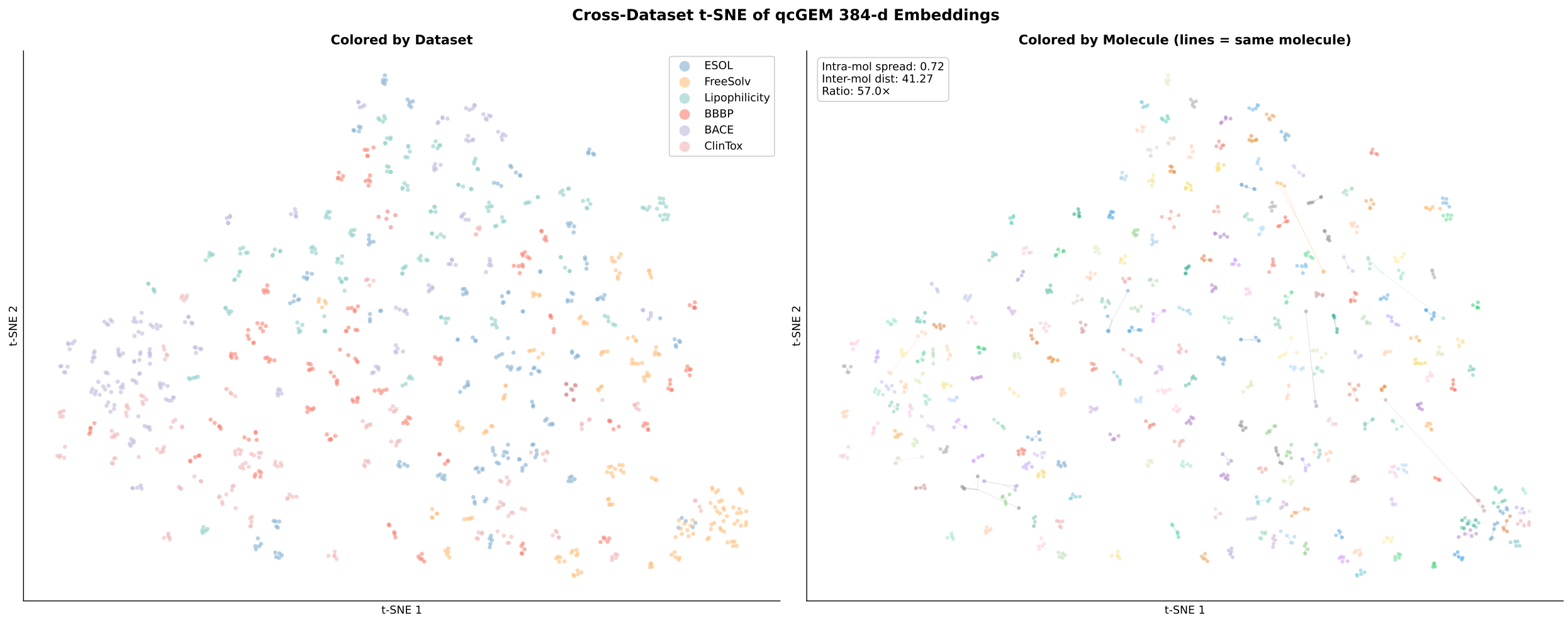

Supplementary Figure 35. Cross-dataset t-SNE of qcGEM embeddings. Left: colored by dataset origin, showing no dataset-specific bias. Right: colored by molecular identity, with lines connecting same-molecule conformers. The intra-molecule spread (0.72) is $57\times$ smaller than the inter-molecule distance (41.27).

4.3.2 Downstream prediction consistency

Beyond embedding-level analysis, we further evaluated whether conformational variations propagate into downstream molecular property predictions. We trained downstream the probe network (*i.e.* MLP heads) on Rank-0 conformers (10 random seeds, frozen encoder, "Hard" embedding mode) and then evaluated on all five conformer ranks for each test molecule.

Supplementary Figure 36 presents scatter plots of per-molecule predictions from Rank-0 versus Rank-1 through Rank-4. High Pearson correlation coefficients (r) are observed across all datasets and conformer ranks, indicating strong prediction consistency. For classification tasks (BBBP, BACE and ClinTox), the predicted probabilities from different conformers show tight agreement along the diagonal; for regression tasks (ESOL, FreeSolv and Lipophilicity), the predicted values remain highly correlated across conformers.

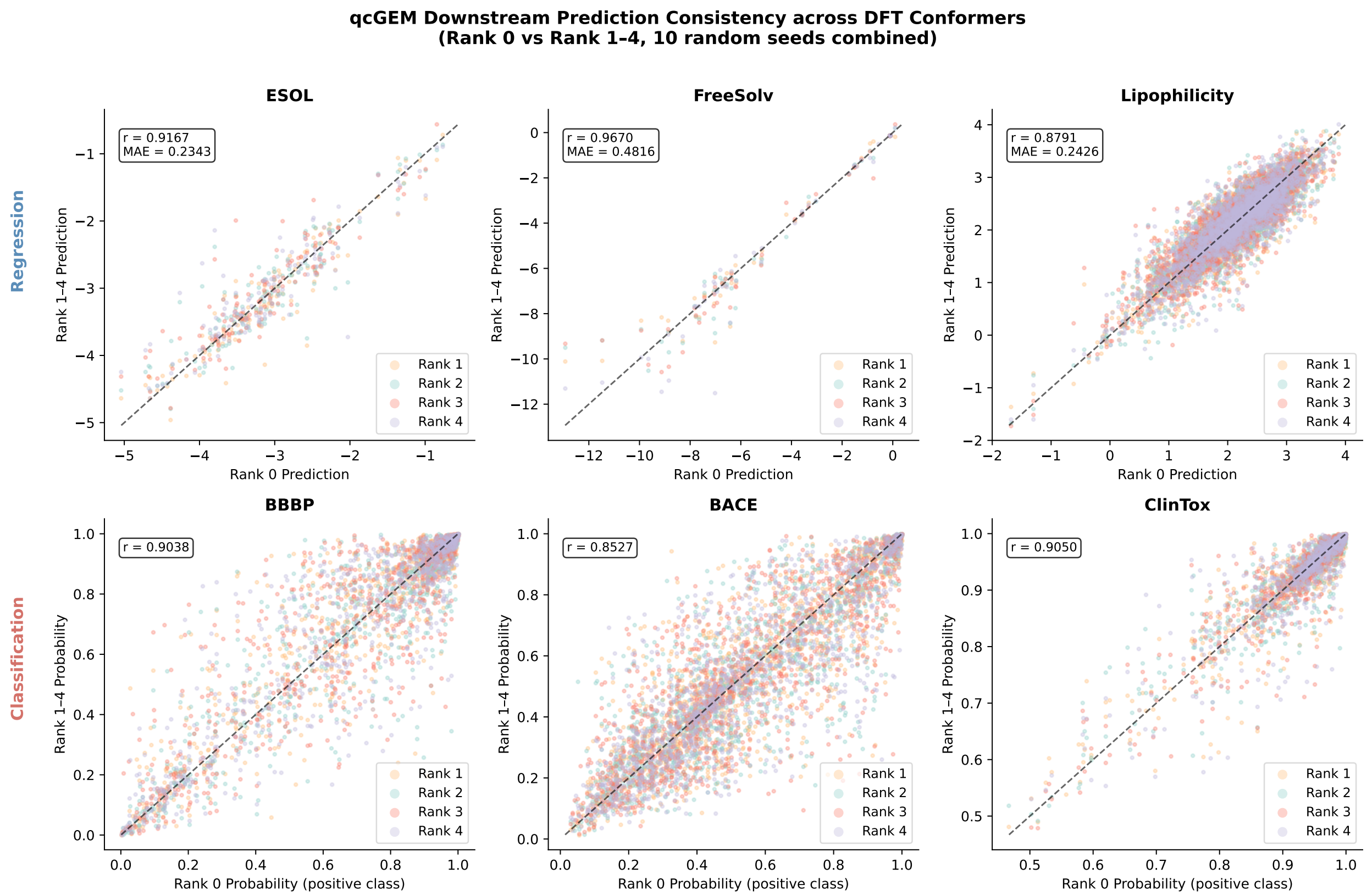

Supplementary Figure 36. Downstream prediction consistency across conformers. Scatter plots of per-molecule predictions from the Rank-0–trained model evaluated on Rank-0 (horizontal axis) vs. Rank-1 through Rank-4 (vertical axis). High Pearson r values across all datasets and conformer ranks confirm that predictions are highly consistent regardless of which optimized conformer is used.

Moreover, when starting from different conformers of the same molecule, the prediction values exhibit a low level of per-molecule standard deviation across all tested tasks (Supplementary Figure 37). Aggregated based on the evaluation metrics (RMSE for regression tasks and ROC-AUC for classification tasks), these predictions attain a steady performance across distinct conformers, with the inter-conformer difference falling below the intra-conformer variation induced by random seeds (Supplementary Figure 38). These results suggest that the qcGEM embedding captures the essential characteristics of each chemical molecule, without loosing the capacity to reflect the conformational variation.

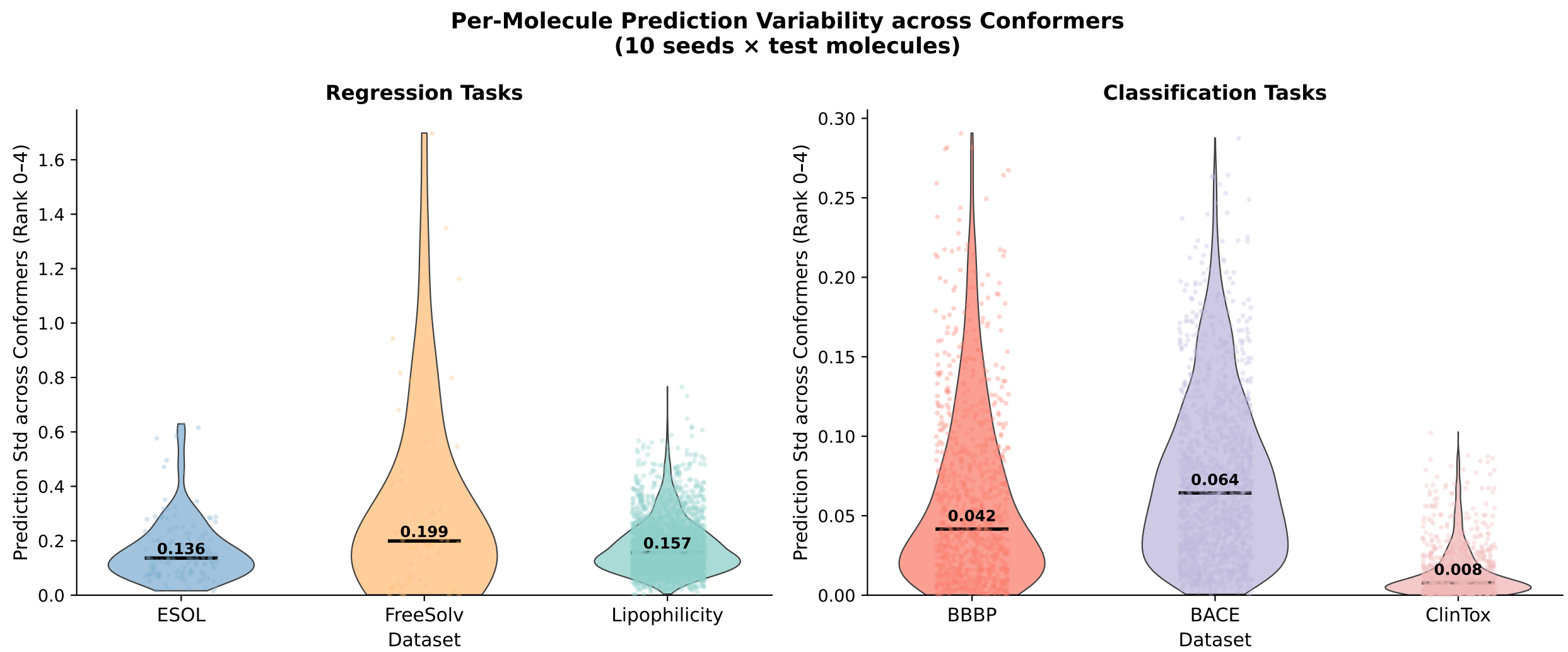

Supplementary Figure 37. Variation of per-molecule prediction across five conformers. Violin plots of the standard deviation of predictions across Rank-0–4 for each molecule. The distributions are concentrated near zero, indicating that conformational flexibility introduces low prediction variation for the vast majority of molecules.

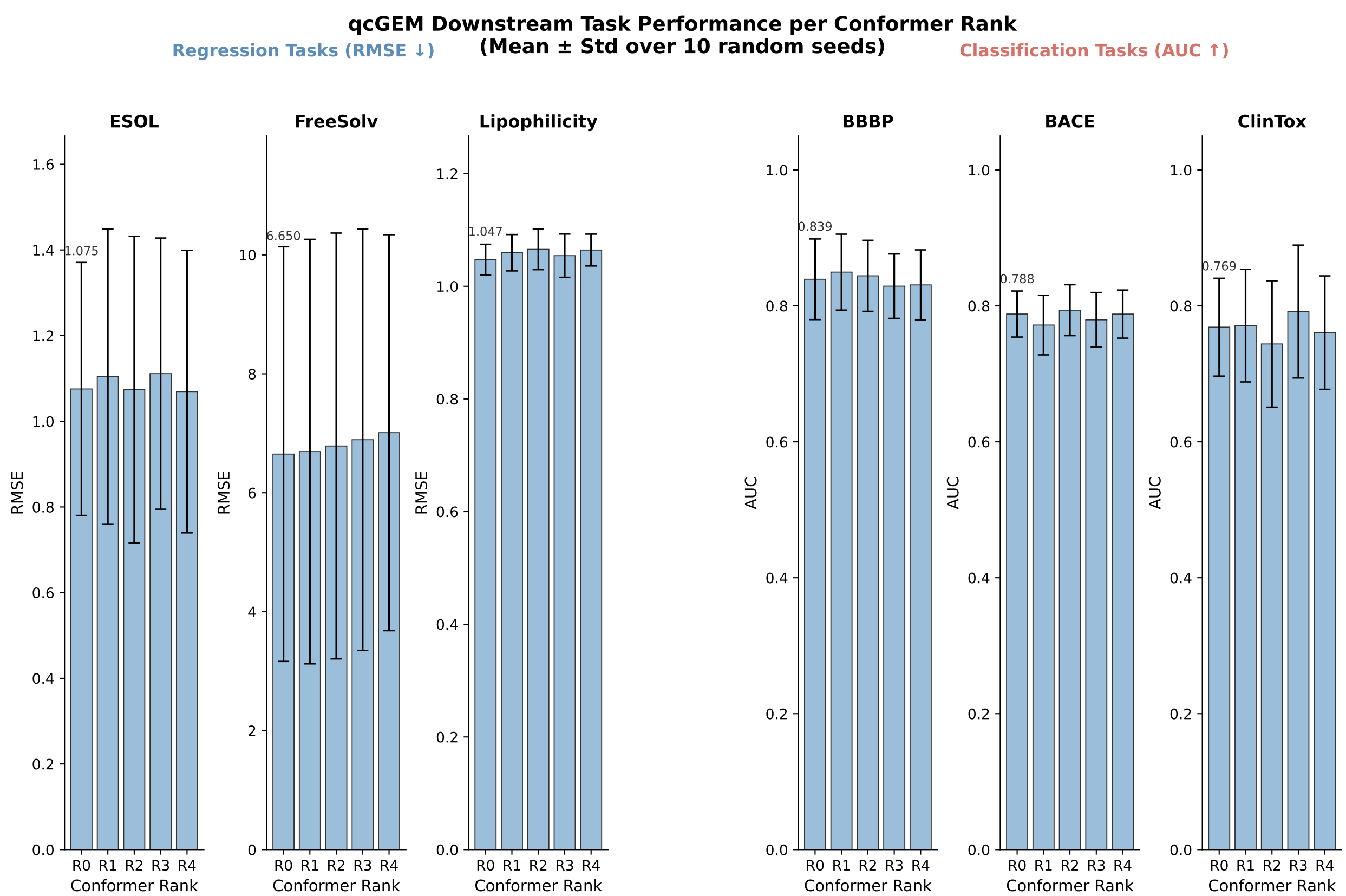

Supplementary Figure 38. Aggregate downstream performance by conformer rank. RMSE (regression, lower is better) and ROC-AUC (classification, higher is better) evaluated independently on each conformer rank (Rank-0 through Rank-4, 10 random seeds). Performance remains stable across all ranks, with inter-rank variation well below the seed-induced standard deviation.

Overall, the above results demonstrate that qcGEM yields highly consistent embeddings and downstream predictions regardless of which low-energy conformer is supplied, confirming that the model captures the chemical essence of molecules. This robustness is supposedly attributed to the GLI unit in the graph encoder (Figure 1c), which effectively aggregates multi-level features including the global molecular properties and local atomic/bond quantum chemical descriptors, rendering the embedding resistant to minor geometric perturbations arising from different local-optimum conformers of the same molecule.

4.4 Direct pair-level evaluation of activity cliffs

The original activity-cliff analysis follows the established MoleculeACE protocol and reports molecule-level $\mathrm{RMSE}_{\mathrm{All}}$ and $\mathrm{RMSE}_{\mathrm{Cliff}}$[27]. These benchmark endpoints, which have also been extensively used in subsequent studies[29,30], are retained in the main text. However, neither the difference nor the correlation reported by such conventional analysis directly tests whether a model is capable of recovering the activity change within a structurally similar molecular pair[31–33]. We therefore added the following pair-level analysis as a complementary evaluation.

4.4.1 Construction of within-target molecular pairs

We constructed the within-target molecule pairs using data of the 30 MoleculeACE tasks. Within each target, every unordered pair of test compounds was enumerated once, with self-pairs, train–test pairs and cross-target pairs excluded. Experimental pair differences were then calculated based on the raw $p\mathrm{Activity}$ values reported by the MoleculeACE dataset.

Following the released MoleculeACE implementation[27], three structural similarities were calculated for every pair (i, j): (i) Tanimoto similarity between radius-2, 1,024-bit Morgan fingerprints[1]; (ii) Tanimoto similarity between radius-2, 1,024-bit fingerprints generated after applying `MakeScaffoldGeneric`[34]; and (iii) normalized Levenshtein similarity between the supplied SMILES strings,

$$s_{ij}^{\mathrm{Lev}} = 1 - \frac{d_{\mathrm{Lev}}(\mathrm{SMILES}_i, \mathrm{SMILES}_j)}{\max(|\mathrm{SMILES}_i|, |\mathrm{SMILES}_j|)}. \tag{S20}$$

The MoleculeACE three-route criterion is a union, represented as

$$s_{ij} = \max\left(s_{ij}^{\mathrm{Morgan}}, s_{ij}^{\mathrm{framework}}, s_{ij}^{\mathrm{Lev}}\right). \tag{S21}$$

Hence, a pair will satisfy the similarity threshold when any of the three routes reaches that threshold. Notably, such similarity evaluations were used only for pair construction, rather than for predictive baselines.

We specified the primary activity-cliff definition as $s_{ij} \geq 0.90$ and $|\Delta p\mathrm{Activity}_{ij}| > 1.0$. This activity boundary was strictly set as greater than one $p\mathrm{Activity}$ unit, which corresponds to a potency difference greater than tenfold, with pairs exactly located on the boundary excluded. A tolerance of 10^{-12} was used only to protect this strict boundary from floating-point rounding.

In practice, we attempted various cumulative similarity thresholds of 0.80, 0.85, 0.90, and 0.95 as well as different strict activity-gap thresholds of 0.5, 1.0, and 1.5 $p\mathrm{Activity}$ units for the pair construction, but finally chose $s_{ij} \geq 0.90$ and $|\Delta p\mathrm{Activity}_{ij}| > 1.0$, for a manageable testing pair library (Supplementary Table 48). This primary set contains 1,043 activity-cliff pairs from 29 of the 30 MoleculeACE targets.

Supplementary Table 48. Numbers of within-target test–test pairs. Similarity rows are cumulative. The first numerical column contains all pairs satisfying the structural-similarity threshold; the remaining columns additionally require the indicated strict experimental activity gap. The primary definition is shown in bold.

Similarity threshold	All similar pairs	$\lvert\Delta p\mathrm{Activity}\rvert > 0.5$	$\lvert\Delta p\mathrm{Activity}\rvert > 1.0$	$\lvert\Delta p\mathrm{Activity}\rvert > 1.5$
$s_{ij} \geq 0.80$	15,736	8,362	4,285	2,159
$s_{ij} \geq 0.85$	9,301	4,784	2,309	1,129
$\mathbf{s_{ij} \geq 0.90}$	**4,874**	2,362	**1,043**	491
$s_{ij} \geq 0.95$	2,163	1,000	395	180

4.4.2 Pair-level endpoint and comparison protocol

We regenerated test-set predictions from archived checkpoints of qcGEM and other baselines (30 MoleculeACE tasks, five random seeds per task per model). For pair (i, j) and random seed k, the experimental and predicted signed differences are

$$\Delta y_{ij} = y_i - y_j, \qquad \Delta \hat{y}_{ij}^{(k)} = \hat{y}_i^{(k)} - \hat{y}_j^{(k)}, \tag{S22}$$

where y and $\hat{y}$ denote experimental value and model prediction, respectively.

We specified the signed pair-difference RMSE as the primary endpoint,

$$\mathrm{RMSE}_{\mathrm{pair}} = \sqrt{\frac{1}{N} \sum_{(i,j)} \left(\Delta \hat{y}_{ij} - \Delta y_{ij}\right)^2}, \tag{S23}$$

which penalizes errors in both the magnitude and direction of a predicted activity change. Additionally, we chose signed-difference MAE, activity-change direction accuracy, Pearson and Spearman correlations between experimental and predicted signed differences, and RMSE between the predicted and experimental absolute gaps, as supporting endpoints. Notably, the last endpoint discards direction and was therefore only treated as a sensitivity analysis.

Here, we evaluated qcGEM against all baseline methods reported in Figure 3a, which gives rise to 32 comparison methods in total (9 molecular fingerprints and 23 pre-trained molecular representations).

All methods were evaluated on the identical 1,043 within-target molecular pairs constructed in Supplementary Table 48. For the primary comparison, uncertainty was estimated using 10,000 paired target-cluster bootstrap replicates: targets were sampled with replacement, all pairs from each selected target were retained, while qcGEM and every comparator used exactly the same resampled targets and pairs. We report qcGEM-minus-baseline differences, for which negative values favor qcGEM.

4.4.3 Evaluation results

Primary comparison. qcGEM achieves the lowest signed pair-difference RMSE among all 33 methods (Supplementary Table 49). Its five-seed mean is 1.4055 ± 0.0044 $p\mathrm{Activity}$ units. The 32 baseline means range from 1.4706 (FCFP) to 1.7398 (GCC), corresponding to relative performance degradation of 4.4–19.2%.

As shown in Supplementary Figure 39a, the qcGEM point estimate is prominently lower than all baselines. The paired 95% bootstrap interval excludes zero for 31 of 32 comparisons (Supplementary Figure 39b), with FCFP (-0.0651, 95% confidence interval (CI) $[-0.1403, 0.0131]$) as the only inconclusive comparison.

Supplementary Table 49. Signed pair-difference RMSE on the primary activity-cliff set. Values are mean ± sample SD across five seeds. Individual-model and paired qcGEM-minus-baseline 95% confidence intervals were obtained from 10,000 target-cluster bootstrap replicates. Lower RMSE is better.

Method	Signed ΔRMSE	Bootstrap 95% CI	qcGEM − baseline (95% CI)
qcGEM	$\mathbf{1.4055 \pm 0.0044}$	$[1.3001, 1.4990]$	—
Molformer	1.4716 ± 0.0053	$[1.3631, 1.5726]$	-0.0661 $[-0.1197, -0.0089]$
ECFP	1.4863 ± 0.0087	$[1.3479, 1.6147]$	-0.0807 $[-0.1545, -0.0045]$
FCFP	1.4706 ± 0.0060	$[1.3416, 1.5915]$	-0.0651 $[-0.1403, 0.0131]$
AvalonFP	1.4916 ± 0.0022	$[1.3581, 1.6079]$	-0.0861 $[-0.1523, -0.0072]$
GROVER-Base	1.4956 ± 0.0070	$[1.3746, 1.6044]$	-0.0900 $[-0.1336, -0.0523]$
RDKFP	1.5291 ± 0.0025	$[1.4147, 1.6307]$	-0.1235 $[-0.1623, -0.0825]$
MoleBERT	1.5738 ± 0.0016	$[1.4465, 1.6940]$	-0.1682 $[-0.2226, -0.1129]$
TTGEN	1.5461 ± 0.0037	$[1.4183, 1.6603]$	-0.1406 $[-0.1857, -0.0913]$
APGEN	1.5928 ± 0.0026	$[1.4842, 1.6892]$	-0.1873 $[-0.2358, -0.1291]$
PatternFP	1.6204 ± 0.0016	$[1.4966, 1.7438]$	-0.2149 $[-0.2823, -0.1491]$
MACCS	1.6171 ± 0.0030	$[1.5018, 1.7315]$	-0.2115 $[-0.2699, -0.1549]$
LayeredFP	1.5798 ± 0.0024	$[1.4629, 1.6893]$	-0.1743 $[-0.2335, -0.1121]$
UniMol-GA	1.6848 ± 0.0005	$[1.5701, 1.7941]$	-0.2793 $[-0.3473, -0.2002]$
GROVER-Large	1.5802 ± 0.0151	$[1.4676, 1.6881]$	-0.1746 $[-0.2283, -0.1270]$
GraphMVP	1.6505 ± 0.0011	$[1.5427, 1.7533]$	-0.2450 $[-0.2962, -0.1905]$
UniMol-G	1.7179 ± 0.0010	$[1.6024, 1.8265]$	-0.3123 $[-0.3838, -0.2284]$
GPT-GNN	1.7311 ± 0.0008	$[1.6242, 1.8364]$	-0.3256 $[-0.4030, -0.2387]$
GCC	1.7398 ± 0.0013	$[1.6368, 1.8356]$	-0.3342 $[-0.4211, -0.2446]$
JOAOv2	1.7000 ± 0.0010	$[1.5943, 1.8035]$	-0.2945 $[-0.3606, -0.2165]$
AttrMask	1.6921 ± 0.0008	$[1.6026, 1.7802]$	-0.2866 $[-0.3448, -0.2235]$
JOAO	1.6877 ± 0.0009	$[1.5938, 1.7764]$	-0.2821 $[-0.3519, -0.2036]$
InfoGraph	1.7335 ± 0.0025	$[1.6286, 1.8375]$	-0.3280 $[-0.3966, -0.2504]$
MolCLR-GCN	1.7303 ± 0.0002	$[1.6455, 1.8103]$	-0.3247 $[-0.3878, -0.2577]$
PosPred	1.7156 ± 0.0019	$[1.6197, 1.8077]$	-0.3101 $[-0.3737, -0.2381]$
MolCLR-GIN	1.7349 ± 0.0005	$[1.6420, 1.8264]$	-0.3294 $[-0.3903, -0.2625]$
EdgePred	1.7311 ± 0.0011	$[1.6363, 1.8241]$	-0.3255 $[-0.3814, -0.2613]$
GraphCL	1.7148 ± 0.0012	$[1.6275, 1.7965]$	-0.3093 $[-0.3740, -0.2400]$
3D-InfoMax	1.7302 ± 0.0012	$[1.6376, 1.8199]$	-0.3247 $[-0.3858, -0.2581]$
3D-EMGP	1.7302 ± 0.0012	$[1.6376, 1.8199]$	-0.3247 $[-0.3858, -0.2581]$
TransM	1.5180 ± 0.0027	$[1.3839, 1.6568]$	-0.1124 $[-0.1920, -0.0497]$
MPG	1.5173 ± 0.0025	$[1.3918, 1.6409]$	-0.1117 $[-0.1848, -0.0401]$
KANO	1.6949 ± 0.0006	$[1.6095, 1.7752]$	-0.2893 $[-0.3482, -0.2268]$

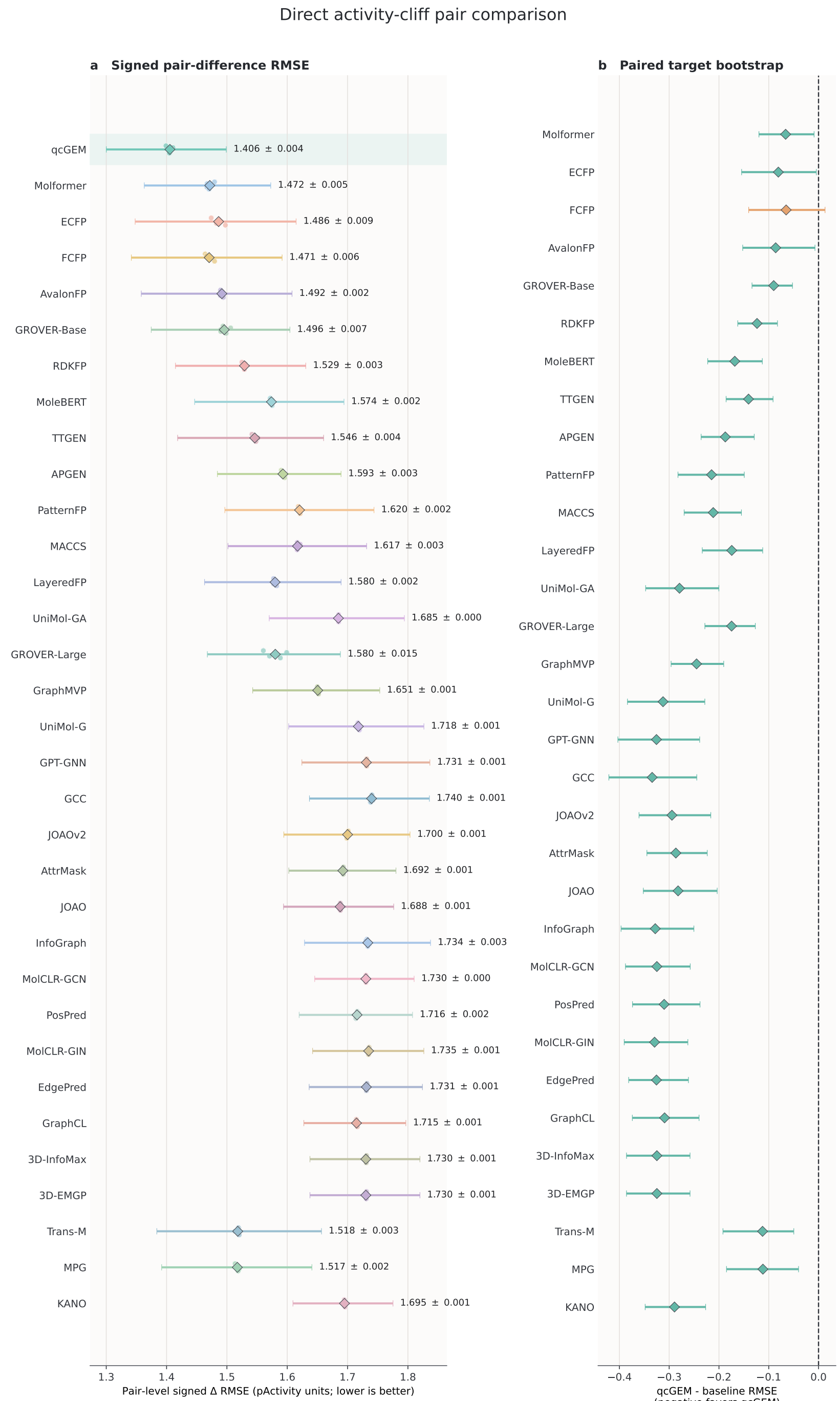

Supplementary Figure 39. Direct activity-cliff pair comparison. Signed pair-difference RMSE for qcGEM and all 32 non-qcGEM methods on the same 1,043 test–test activity-cliff pairs. **a)** Circles denote individual seeds, diamonds denote five-seed means, horizontal intervals denote target-cluster bootstrap 95% confidence intervals, and labels report mean ± sample SD. **b)** Paired qcGEM-minus-baseline RMSE differences with target-cluster bootstrap 95% confidence intervals; negative values favor qcGEM and the dashed line marks zero.

Supporting metrics. The supporting signed-difference endpoints agree with the primary result (Supplementary Figure 40). qcGEM has the lowest signed-difference MAE (1.2210 ± 0.0043 vs. 1.2696–1.6330 in baselines), the highest direction accuracy (0.7686 ± 0.0055 vs. 0.4422–0.7304 in baselines), the highest signed-difference Pearson correlation (0.6104 ± 0.0046 vs. 0.0222–0.5554 in baselines), and the highest signed-difference Spearman correlation (0.5998 ± 0.0044 vs. -0.0200–0.5486 in baselines).

Absolute-gap RMSE provides a less uniform ranking because this metric neglects the direction of activity change. Nevertheless, qcGEM ranks third (1.2451 ± 0.0048), behind ECFP and FCFP but ahead of the other 30 baselines. Supporting metric values for selected top-performing methods are listed in Supplementary Table 50.

Supplementary Table 50. Selected supporting metrics on the primary activity-cliff pair set. Values are mean ± sample SD across five seeds. Arrows indicate the favorable direction.

Method	Signed ΔMAE ↓	Direction accuracy ↑	Pearson r ↑	Spearman ρ ↑	Absolute-gap RMSE ↓
qcGEM	$\mathbf{1.2210 \pm 0.0043}$	$\mathbf{0.7686 \pm 0.0055}$	$\mathbf{0.6104 \pm 0.0046}$	$\mathbf{0.5998 \pm 0.0044}$	1.2451 ± 0.0048
ECFP	1.2759 ± 0.0106	0.7101 ± 0.0099	0.5151 ± 0.0089	0.5024 ± 0.0102	$\mathbf{1.2118 \pm 0.0089}$
FCFP	1.2696 ± 0.0052	0.7114 ± 0.0043	0.5358 ± 0.0066	0.5108 ± 0.0081	1.2354 ± 0.0039
AvalonFP	1.3205 ± 0.0034	0.7060 ± 0.0037	0.5252 ± 0.0014	0.5082 ± 0.0012	1.2937 ± 0.0048
TTGEN	1.3727 ± 0.0040	0.6706 ± 0.0025	0.4621 ± 0.0048	0.4320 ± 0.0057	1.3271 ± 0.0027
Molformer	1.3018 ± 0.0073	0.7304 ± 0.0034	0.5554 ± 0.0052	0.5486 ± 0.0054	1.3012 ± 0.0050
MPG	1.3254 ± 0.0021	0.7022 ± 0.0039	0.4892 ± 0.0034	0.4657 ± 0.0032	1.2855 ± 0.0025
TransM	1.3399 ± 0.0023	0.7241 ± 0.0046	0.4961 ± 0.0036	0.4944 ± 0.0037	1.3408 ± 0.0011
KANO	1.5843 ± 0.0007	0.6075 ± 0.0011	0.2090 ± 0.0022	0.2196 ± 0.0015	1.5913 ± 0.0015

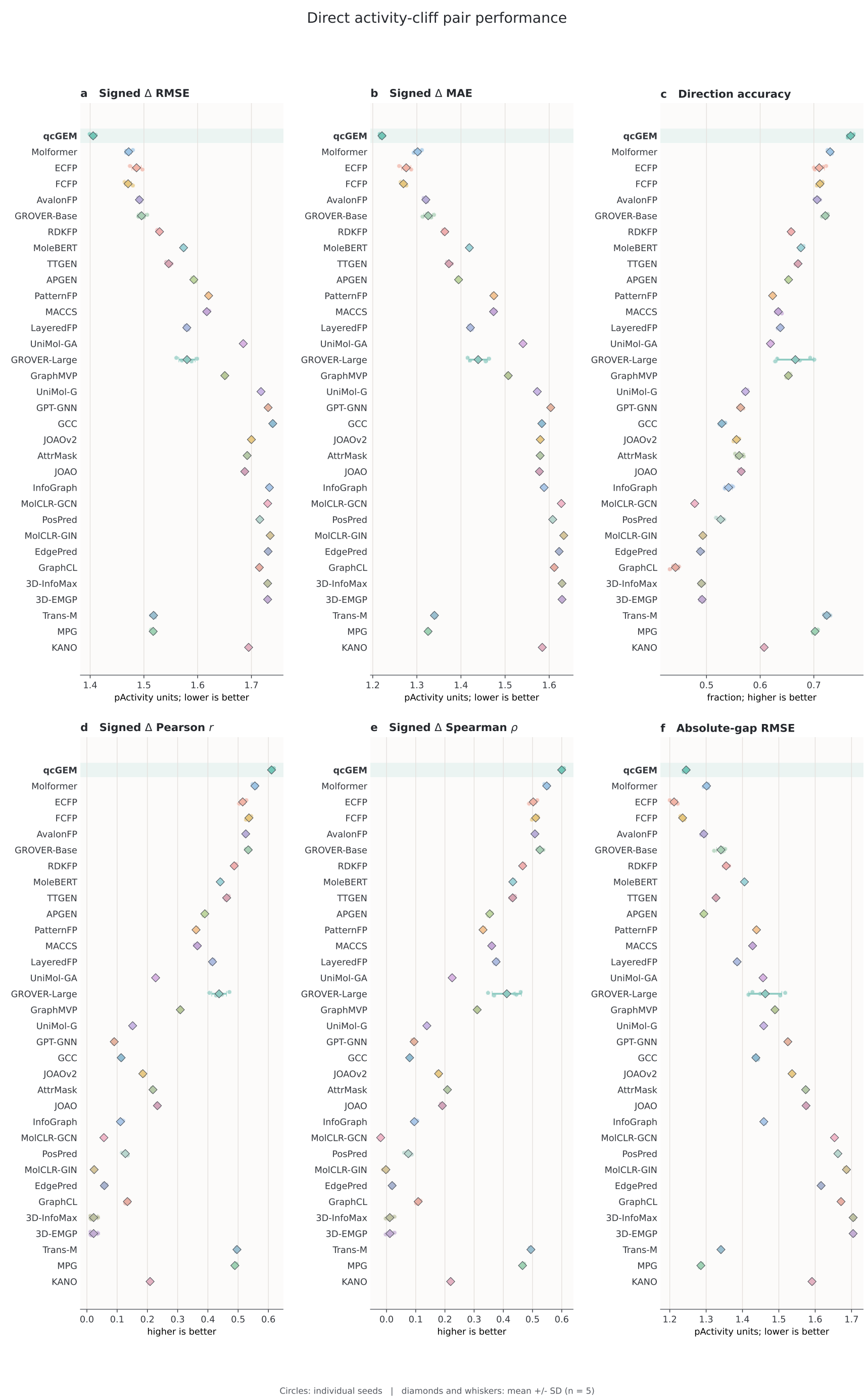

Supplementary Figure 40. Supporting pair-level activity-cliff metrics. All 33 methods were evaluated on the same 1,043 pairs. Panels show **a)** signed-difference RMSE, **b)** signed-difference MAE, **c)** direction accuracy, **d)** signed-difference Pearson correlation, **e)** signed-difference Spearman correlation, and **f)** absolute-gap RMSE. Circles denote individual seeds; diamonds and whiskers denote mean ± sample SD across five seeds.

For completeness, qcGEM-only gap and cliff-detection metrics are retained in Supplementary Table 51. Regression and direction metrics were evaluated on the 1,043 strict cliff pairs. To convert the continuous regression predictions into a cliff-detection evaluation, each of the 4,874 structurally similar pairs was assigned the experimental binary label $z_{ij} = \mathbf{1}\{|\Delta y_{ij}| > 1.0\}$: the 1,043 strict cliff pairs are positive and the remaining 3,831 pairs are negative. The continuous predicted absolute activity gap, $g_{ij} = |\Delta \hat{y}_{ij}| = |\hat{y}_i - \hat{y}_j|$, serves as the detection score, with a larger value indicating stronger model evidence for a cliff. Thus, no separate classifier was trained and no single threshold was imposed on the predicted gap. Instead, varying the threshold on g_{ij} will generate the receiver operating characteristic (ROC) and precision–recall (PR) curves[35–37]. The area under the ROC curve (AUROC) summarizes the true-positive rate vs. the false-positive rate across these thresholds; equivalently, it is the probability that a randomly selected cliff pair receives a higher score than a randomly selected non-cliff pair, with tied scores receiving half credit. Average precision (AP) is the non-interpolated summary of the PR curve, $\mathrm{AP} = \sum_n (R_n - R_{n-1}) P_n$, where P_n and R_n are the precision and recall at the nth distinct score threshold. Because the no-information reference for AP equals the positive prevalence ($1{,}043/4{,}874 = 0.2140$), we also report AP divided by prevalence as the fold enrichment over random ranking. These additional values do not replace the prespecified between-model endpoint.

Supplementary Table 51. Additional qcGEM pair-level metrics under the primary definition. Values are mean $\pm$ sample SD across five seeds; confidence intervals were obtained from 2,000 target-cluster bootstrap replicates.

Metric	Five-seed mean $\pm$ SD	Target-bootstrap 95% CI
Signed ΔRMSE	1.4055 ± 0.0044	$[1.3052, 1.4996]$
Signed ΔMAE	1.2210 ± 0.0043	$[1.1197, 1.3182]$
Absolute-gap RMSE	1.2451 ± 0.0048	$[1.1464, 1.3363]$
Absolute-gap MAE	1.0815 ± 0.0046	$[0.9913, 1.1685]$
Direction accuracy	0.7686 ± 0.0055	$[0.7280, 0.8020]$
Absolute-gap Pearson r	0.2365 ± 0.0105	$[0.1473, 0.3013]$
Absolute-gap Spearman ρ	0.1920 ± 0.0074	$[0.1153, 0.2619]$
Cliff-pair AUROC	0.6580 ± 0.0026	$[0.6205, 0.6923]$
Cliff-pair AP	0.3861 ± 0.0036	$[0.3291, 0.4403]$
AP/prevalence	1.8043 ± 0.0170	$[1.5533, 2.1056]$

Interpretation and limitations. The direct pair-level comparison addresses the defining activity-cliff question more directly than the original molecule-level RMSE difference or training-trajectory correlation. On the prespecified endpoint, qcGEM had the lowest signed pair-difference RMSE among all 33 methods. Nevertheless, the absolute-gap analysis limits the claim: the mean predicted absolute gap is 0.5716 ± 0.0049 pActivity units vs. an experimental mean of 1.6318, meaning that $96.09\% \pm 0.23\%$ of strict cliff-pair gaps are underestimated. Thus, the results support improved recovery of signed within-pair activity changes, but not uniformly superior calibration under every alternative metric.

5 Pseudo Codes and Algorithms

5.1 Algorithms of model

Algorithm 1: qcGEM

Input: Original Molecule

Output: Recovered Molecule

def qcGEM($\text{Molecule}_{\text{Original}}$)
    # Preprocessing
    $\text{Molecule}_{\text{Input}} \leftarrow \text{Preprocess}(\text{Molecule}_{\text{Original}})$
    # Encoding
    $\text{Embedding} \leftarrow \text{Encoder}(\text{Molecule}_{\text{Input}})$
    # Encoder to Decoder
    $\text{Embedding\_} \leftarrow \text{Encoder2Decoder}(\text{Embedding})$
    # Decoding
    $\text{Molecule}_{\text{Recovered}} \leftarrow \text{Decoder}(\text{Embedding\_})$
    return $\text{Molecule}_{\text{Recovered}}$

Algorithm 2: Preprocess

Input: Original Molecule

Output: Inited Molecule

def Preprocess($\text{Molecule}_{\text{Original}}$, $\text{Mask\_Ratio}_{\text{global}}$=1.0, $\text{Mask\_Ratio}_{\text{local}}$=0.3, $\text{Replace\_Ratio}_{\text{local}}$=0.3)

$\quad \text{Global}, \text{Node}, \text{Edge} \leftarrow \text{Molecule}_{\text{Original}}$

$\quad$ # Feature Normalization

$\quad \text{Node} \leftarrow \text{LayerNorm}(\text{Node}) \qquad \in \mathbb{R}^{N \times d_{\text{Node}}}$

$\quad \text{Edge} \leftarrow \text{LayerNorm}(\text{Edge}) \qquad \in \mathbb{R}^{E \times d_{\text{Edge}}}$

$\quad$ # Token initialize

$\quad \text{Token}_{\text{global}} \leftarrow \text{LearnableParameter}(0) \qquad \in \mathbb{R}^{1 \times 712}$

$\quad \text{Token}_{\text{node}} \leftarrow \text{LearnableParameter}(0) \qquad \in \mathbb{R}^{1 \times 80}$

$\quad \text{Token}_{\text{edge}} \leftarrow \text{LearnableParameter}(0) \qquad \in \mathbb{R}^{1 \times 53}$

$\quad \text{Token}_{\text{atom\_type}} \leftarrow \text{LearnableParameter}(0) \qquad \in \mathbb{R}^{1 \times 1}$

$\quad$ # Mask and replace

$\quad \text{Mask\_Index}_{\text{global}} \leftarrow \text{BernoulliSample}(\text{prob} = \text{Mask\_Ratio}_{\text{global}}) \qquad \in \{0, 1\}$

$\quad \text{Global}[\text{Mask\_Index}_{\text{global}}] \leftarrow \text{Token}_{\text{global}}$

$\quad \text{Mask\_Index}_{\text{node}} \leftarrow \text{BernoulliSample}(\text{prob} = \text{Mask\_Ratio}_{\text{local}}) \qquad \in \{0, 1\}$

$\quad \text{Node}[\text{Mask\_Index}_{\text{node}}] \leftarrow \text{Token}_{\text{node}}$

$\quad \text{Atom\_Type}[\text{Mask\_Index}_{\text{node}}] \leftarrow \text{Token}_{\text{atom\_type}}$

$\quad \text{Replace\_Index}_{\text{node}} \leftarrow \text{BernoulliSample}(\text{prob} = \text{Replace\_Ratio}_{\text{local}})$

$\quad \text{Node}[\text{Replace\_Index}_{\text{node}}] \leftarrow \text{RandomSampleFrom}(\text{Node})$

$\quad \text{Mask\_Index}_{\text{edge}} \leftarrow \text{BernoulliSample}(\text{prob} = \text{Mask\_Ratio}_{\text{local}}) \qquad \in \{0, 1\}$

$\quad \text{Edge}[\text{Mask\_Index}_{\text{edge}}] \leftarrow \text{Token}_{\text{edge}}$

$\quad \text{Replace\_Index}_{\text{edge}} \leftarrow \text{BernoulliSample}(\text{prob} = \text{Replace\_Ratio}_{\text{local}})$

$\quad \text{Edge}[\text{Replace\_Index}_{\text{edge}}] \leftarrow \text{RandomSampleFrom}(\text{Edge})$

$\quad$ # Add Noise to Coordinates

$\quad \text{XYZ}_{\text{noised}} \leftarrow \text{Add\_Noise}(\text{XYZ}, -1 \sim 1\,\text{Å})$

$\quad$ # Return

$\quad \text{Molecule}_{\text{Inited}} \leftarrow \text{Collect}\big([\text{XYZ}_{\text{noised}}, \text{Global}, \text{Node}, \text{Edge}]\big)$

$\quad$ **return** $\text{Molecule}_{\text{Inited}}$

Algorithm 3: Encoder

Input: Inited Molecule

Output: Embedding

def Encoder(*Inited Molecule*)

  $\mathrm{XYZ}, \mathrm{Global}, \mathrm{Node}, \mathrm{Edge} \leftarrow \mathrm{Molecule}_{\mathrm{Inited}}$

  # Positional Embedding

  $\mathrm{PE}_{\mathrm{node}} \leftarrow \mathrm{GeometryModule}_1(\mathrm{XYZ}, \mathrm{Atom\_Type}) \quad \in \mathbb{R}^{N \times d_{\mathrm{PE}}}$

  $\mathrm{PE}'_{\mathrm{node}} \leftarrow \mathrm{GeometryModule}_2(\mathrm{XYZ}, \mathrm{Atom\_Type}) \quad \in \mathbb{R}^{N \times d_{\mathrm{PE}}}$

  $\mathrm{PE}_{\mathrm{edge}} \leftarrow \mathrm{MLP}\left(\left\|\mathrm{r}_i - \mathrm{r}_j\right\|_2^2\right)$ for $(i,j)=\mathrm{Index}_{\mathrm{edge}} \quad \in \mathbb{R}^{E \times 1}$

  # Input Embedding

  $\mathrm{Global}_{\mathrm{emb}} \leftarrow \mathrm{MLP}(\mathrm{Global}) \quad \in \mathbb{R}^{B \times d_g}$

  $\mathrm{Node}_{\mathrm{emb}} \leftarrow \mathrm{MLP}\left([\mathrm{Node} \,\|\, \mathrm{PE}_{\mathrm{node}}]\right) \quad \in \mathbb{R}^{N \times H_n}$

  $\mathrm{Edge}_{\mathrm{emb}} \leftarrow \mathrm{MLP}(\mathrm{Edge}) \quad \in \mathbb{R}^{E \times H_e}$

  $\mathcal{L}=\{0, 3, 7, 11, 15\}$

  # Encoding

  for $\ell \leftarrow 0$ **to** 15 **do**

    $\mathrm{Node}_{\mathrm{res}} \leftarrow \mathrm{Node},\ \mathrm{Edge}_{\mathrm{res}} \leftarrow \mathrm{Edge}$

    $\mathrm{Node} \leftarrow \mathrm{qcGEM{-}Transformer}_{\ell}\left(\mathrm{Node},\ \mathrm{Index}_{\mathrm{edge}},\ \mathrm{Edge},\ \mathrm{PE}_{\mathrm{edge}}\right)$

    $\mathrm{Node} \leftarrow \mathrm{MLP}_{\ell}\left([\mathrm{Node}\ \|\ \mathrm{PE}_{\mathrm{node}}]\right)$

    # Global-Local Interaction

    $\mathrm{Local}_{\mathrm{emb}} \leftarrow \mathrm{MeanAgg}_{\ell}(\mathrm{Node},\ \mathrm{Index{=}batch})$

    $\mathrm{O} \leftarrow \mathrm{GLI}_{\ell}\left([\mathrm{Global}_{\mathrm{emb}}\ \|\ \mathrm{Local}_{\mathrm{emb}}]\right)$

    # Global-Local Update

    $\mathrm{Node} \leftarrow \mathrm{Local\_Update}_{\ell}\left([\mathrm{Node}\ \|\ \mathrm{O}[\mathrm{batch}]]\right)$

    $\mathrm{Edge} \leftarrow [\mathrm{Node_i}\ \|\ \mathrm{Edge}\ \|\ \mathrm{Node_j}]$ for $(i,j) \in \mathrm{Index}_{\mathrm{edge}}$

    $\mathrm{Graph}_{\mathrm{res}} \leftarrow \mathrm{O}$

    $\mathrm{Graph} \leftarrow \mathrm{Global\_Update}_{\ell}(\mathrm{O})$

    # Collect Embedding

    if $\ell \in \mathcal{L}$ **then**

      $\mathrm{G}_{\mathrm{emb}}$ `.append`(graph); $\mathrm{N}_{\mathrm{emb}}$ `.append`(node); $\mathrm{E}_{\mathrm{emb}}$ `.append`(edge);

    end

    # Add Residual

    $\mathrm{Graph} \leftarrow \mathrm{MLP}_{\ell}(\mathrm{Graph}) + \mathrm{Graph}_{\mathrm{res}}$

    $\mathrm{Node} \leftarrow \mathrm{MLP}_{\ell}(\mathrm{Node}) + \mathrm{Node}_{\mathrm{res}}$

    $\mathrm{Edge} \leftarrow \mathrm{MLP}_{\ell}(\mathrm{Edge}) + \mathrm{Edge}_{\mathrm{res}}$

  end

  $\mathrm{Embedding} \leftarrow [\,\mathrm{G}_{\mathrm{emb}},\ \mathrm{N}_{\mathrm{emb}},\ \mathrm{E}_{\mathrm{emb}}\,]$

  return Embedding

Algorithm 4: Encoder to Decoder

Input: Embedding

Output: Embedding_

def Encoder2Decoder(Embedding)

    # ReMask

    $\text{Embedding}_{\text{remasked}} \leftarrow$ $\text{Preprocess}(\text{Embedding}, \text{Mask\_Ratio}_{\text{global}}{=}0.0, \text{Mask\_Ratio}_{\text{local}}{=}0.0, \text{Replace\_Ratio}_{\text{local}}{=}0.0)$

    # Output

    $\text{Graph}_{\text{emb\_}} \leftarrow \text{MLP}\big(\text{Mean}(\text{G}_{\text{remasked}})\big)$

    $\text{Node}_{\text{emb\_}} \leftarrow \text{MLP}\big(\big[\text{Mean}(\text{N}_{\text{remasked}}) \,\|\, \text{PE}'_{\text{node}}\big]\big)$

    $\text{Edge}_{\text{emb\_}} \leftarrow \text{MLP}\big(\text{Mean}(\text{E}_{\text{remasked}})\big)$

    $\text{Embedding\_} \leftarrow [\,\text{Graph}_{\text{emb\_}},\ \text{Node}_{\text{emb\_}},\ \text{Edge}_{\text{emb\_}}\,]$

    return Embedding_

Algorithm 5: Decoder

Input: Embedding_

Output: Recovered Molecule

```
def Decoder(Embedding_)
    graph, node, edge ← Embedding_
    # Recover Global Info
    Global_Recovered ← MLP(graph)
    # Recover Local Info
    Node_Recovered ← MLP(node)
    Edge_Recovered ← MLP(edge)
    # Coordinates Denoising
    Node_xyz ← MLP(node)
    Edge_xyz ← MLP(edge)
    XYZ_1, XYZ_2, Node_xyz, Edge_xyz ← Folding_Optimizer_1(Node_xyz, Edge_xyz)
    XYZ_3, XYZ_4, Node_xyz, Edge_xyz ← Folding_Optimizer_2(Node_xyz, Edge_xyz)
    XYZ_5, XYZ_6, Node_xyz, Edge_xyz ← Folding_Optimizer_3(Node_xyz, Edge_xyz)
    XYZ_Denoised ← Stack([XYZ_1, XYZ_2, XYZ_3, XYZ_4, XYZ_5, XYZ_6])
    # Recover the Molecule
    Molecule_Recovered ← Collect([XYZ_Denoised, Global_Recovered, Node_Recovered, Edge_Recovered])
    return Molecule_Recovered
```

Algorithm 6: Geometry Module

Input: Coordinates, AtomType

Output: $\mathrm{PE_{node}}$

def GeometryModule(XYZ, Atom_Type)

  # Define the graph

  $\mathrm{Index_{edge}} \leftarrow \mathrm{RadiusGraph}(\mathrm{XYZ}, r{=}\mathrm{CutOff})$

  $(i, j) \leftarrow \mathrm{Index_{edge}}$

  $\{\mathrm{r}\} \leftarrow \mathrm{XYZ}$

  $\overrightarrow{\mathrm{v_{ji}}} \leftarrow \mathrm{r_j} - \mathrm{r_i}$

  # Atom Type Embedding

  $\mathrm{x} \leftarrow \mathrm{MLP}(\mathrm{Atom\_Type})$

  # Define the Neighborhood

  $\mathrm{n^i_0}, \mathrm{n^i_1} \leftarrow$ nearest and second-nearest neighbors of i

  $\mathrm{n^j_0}, \mathrm{n^j_1} \leftarrow$ nearest and second-nearest neighbors of j

  $\mathrm{i_{ref}} \leftarrow \begin{cases} \mathrm{n^i_1}, & \mathrm{n^i_0} = \mathrm{j} \\ \mathrm{n^i_0}, & \mathrm{otherwise} \end{cases}$

  $\mathrm{j_{ref}} \leftarrow \begin{cases} \mathrm{n^j_1}, & \mathrm{n^j_0} = \mathrm{i} \\ \mathrm{n^j_0}, & \mathrm{otherwise} \end{cases}$

  $\mathrm{v_{in0}} \leftarrow \mathrm{v}_{i\,n^i_0}, \;\; \mathrm{v_{in1}} \leftarrow \mathrm{v}_{i\,n^i_1}$

  $\mathrm{v_{i_{ref}}} \leftarrow \mathrm{v_{i\,i_{ref}}}, \;\; \mathrm{v_{j_{ref}}} \leftarrow \mathrm{v}_{j\,j_{ref}}$

  # r, θ, ϕ, τ

  $r \leftarrow \|\mathrm{v}_{ji}\|_2$

  $\theta \leftarrow \mathrm{atan2}(\|\mathrm{v}_{ji} \times \mathrm{v}_{in0}\|, \;\; -\mathrm{v}_{ji} \cdot \mathrm{v}_{in0})$

  $\phi \leftarrow \mathrm{atan2}\Big(\big((-\,\mathrm{v}_{ji} \times \mathrm{v}_{in0}) \times (-\,\mathrm{v}_{ji} \times \mathrm{v}_{in1})\big) \cdot \frac{\mathrm{v}_{ji}}{\|\mathrm{v}_{ji}\| + \varepsilon}, \; (-\,\mathrm{v}_{ji} \times \mathrm{v}_{in0}) \cdot (-\,\mathrm{v}_{ji} \times \mathrm{v}_{in1})\Big)$

  $\tau \leftarrow \mathrm{atan2}\Big(\big((\mathrm{v}_{ji} \times \mathrm{v_{j_{ref}}}) \times (\mathrm{v}_{ji} \times \mathrm{v_{i_{ref}}})\big) \cdot \frac{\mathrm{v}_{ji}}{\|\mathrm{v}_{ji}\| + \varepsilon}, \; (\mathrm{v}_{ji} \times \mathrm{v_{j_{ref}}}) \cdot (\mathrm{v}_{ji} \times \mathrm{v_{i_{ref}}})\Big)$

  # RBF $\times$ SH expansion

  $\mathrm{Feature_1} \leftarrow \mathrm{FeatureExpansion}(r, \; \theta, \; \phi)$

  $\mathrm{Feature_2} \leftarrow \mathrm{FeatureExpansion}(r, \; \tau)$

  # Fuse geometry via edge weights

  $\mathrm{x} \leftarrow \mathrm{InteractionBlock}_\ell\big(\mathrm{x}, \; \mathrm{Feature_1}, \; \mathrm{Feature_2}, \; \mathrm{Index_{edge}}\big)$

  # Output

  $\mathrm{PE_{node}} \leftarrow \mathrm{MLP}(\mathrm{x})$

  return $\mathrm{PE_{node}}$

Algorithm 7: qcGEM-Transformer

Input: $\mathrm{Node}, \mathrm{Edge}, \mathrm{PE}_{\mathrm{edge}}$

Output: $\mathrm{Updated\ Node}$

def qcGEM-Transformer$(\mathrm{Node}, \mathrm{Edge}, \mathrm{Index}_{\mathrm{edge}}, \mathrm{PE}_{\mathrm{edge}})$

  # Pre-process

  $\mathrm{Q} \leftarrow \mathrm{MLP}_{\mathrm{Q}}(\mathrm{Node})$

  $\mathrm{K} \leftarrow \mathrm{MLP}_{\mathrm{K}}(\mathrm{Node})$

  $\mathrm{V} \leftarrow \mathrm{MLP}_{\mathrm{V}}(\mathrm{Node})$

  $\mathrm{Edge} \leftarrow \mathrm{MLP}(\mathrm{Edge})$

  $\mathrm{K} \leftarrow \mathrm{K} + \mathrm{Edge}$

  # Scaled Dot-Product Attention (+PE)

  $\mathrm{A} \leftarrow \dfrac{\langle \mathrm{Q}, \mathrm{K} \rangle}{\sqrt{C}} + \mathrm{PE}_{\mathrm{edge}}$

  $\mathrm{A} \leftarrow \mathrm{SoftMax}(\mathrm{A})$

  $\mathrm{A} \leftarrow \mathrm{Dropout}(\mathrm{A})$

  # Message & Aggregate

  $\mathrm{V} \leftarrow \mathrm{V} + \mathrm{E}$

  $\mathrm{M} \leftarrow \mathrm{AV}$

  $\mathrm{Node} \leftarrow \mathrm{Agg}(\mathrm{M})$

  # Skip

  $\mathrm{Node}_{\mathrm{res}} \leftarrow \mathrm{MLP}(\mathrm{Node})$

  $\beta \leftarrow \mathrm{MLP}_{\beta}([\mathrm{Node} \,\|\, \mathrm{Node}_{\mathrm{res}} \,\|\, \mathrm{Node} - \mathrm{Node}_{\mathrm{res}}])$

  $\mathrm{Node} \leftarrow \beta \cdot \mathrm{Node}_{\mathrm{res}} + (1 - \beta) \cdot \mathrm{Node}$

  $\mathrm{Node} \leftarrow \mathrm{Node} + \mathrm{Node}_{\mathrm{res}}$

  return Node

Algorithm 8: GLI

Input: Global Embedding, Local Embedding

Output: Fusion Embedding

def GLI($\mathrm{Global_{emb}}$, $\mathrm{Local_{emb}}$)
    # Pre-process
    $\mathrm{O} \leftarrow \mathrm{MLP}([\mathrm{Global_{emb}} \| \mathrm{Local_{emb}}])$
    $\mathrm{O_{res}} \leftarrow \mathrm{O}$
    # QKV
    $Q \leftarrow \mathrm{MLP_Q}(\mathrm{O})$
    $K \leftarrow \mathrm{MLP_K}(\mathrm{O})$
    $V \leftarrow \mathrm{MLP_V}(\mathrm{O})$
    # Scaled Dot-Product Attention
    $\mathrm{A} \leftarrow (\mathrm{Q}\,\mathrm{K}^\top)/\sqrt{\mathrm{D_h}}$
    $\mathrm{W} \leftarrow \mathrm{SoftMax}(\mathrm{A})$
    $\mathrm{U} \leftarrow \mathrm{WV}$
    # Feed-Forward
    $\mathrm{U} \leftarrow \mathrm{MLP}(\mathrm{U})$
    $\mathrm{O} \leftarrow \mathrm{U} + \mathrm{O_{res}}$
    $\mathrm{O} \leftarrow \mathrm{U} + \mathrm{O_{res}}$
    $\mathrm{O} \leftarrow \mathrm{MLP}(\mathrm{O})$
    $\mathrm{O} \leftarrow \mathrm{O} + \mathrm{O_{res}}$
    return O

Algorithm 9: Global/Local Update

Input: Global Features or Local Features

Output: Updated Result

```
def Global_Update(features)
    features_updated ← MLP(features)
    return features_updated
def Local_Update(features)
    features_updated ← MLP(features)
    return features_updated
```

Algorithm 10: InteractionBlock

Input: Atom Embedding, Geometry Features

Output: Updated Atom Embedding

```
def InteractionBlock(x, Feature_1, Feature_2, Index_edge)
    # Pre-projection on nodes
    x ← MLP(x)
    # Transform RBF×SH edge features into edge weights
    e_1 ← MLP(Feature_1)
    e_2 ← MLP(Feature_2)
    # Edge-weighted GraphConv (two channels)
    h_1 ← EdgeGraphConv(x, edge_index, e_1)
    h_2 ← EdgeGraphConv(x, edge_index, e_2)
    # Fuse two channels
    h ← MLP([h_1 || h_2])
    # Output projection
    h ← h + x
    Output ← MLP(h)
    return Output
```

Algorithm 11: EdgeGraphConv

Input: Node Feature, Edge Weight

Output: Updated Node Feature

```
def EdgeGraphConv(x, e)
    # Per-edge message
    i, j ← Index_edge
    m_{j→i} ← e_{i,j} · x_j
    # Aggregate incoming messages
    s_i ← Σ_{j∈N(i)} m_{j→i}
    # Output
    Output_i ← MLP(x_i) + MLP(s_i)
    return Output
```

Algorithm 12: Folding_Optimizer

Input: $\mathrm{Node_{xyz}},\ \mathrm{Edge_{xyz}}$

Output: Denoised Geometry

def Fold_Optimizer(*xyz*)
  # Stage-1: Predict XYZ_1
  $\mathrm{Node^{res1}_{xyz}} \leftarrow \mathrm{Node_{xyz}}$
  $\mathrm{Edge^{res}_{xyz}} \leftarrow \mathrm{Edge_{xyz}}$
  $\mathrm{Node_{xyz}},\ \mathrm{Edge_{xyz}} \leftarrow \mathrm{NEE_1}(\mathrm{Node_{xyz}}, \mathrm{Edge_{xyz}}, \mathrm{Index_{edge}})$
  $\mathrm{Node_{xyz}} \leftarrow \mathrm{Node_{xyz}} + \mathrm{Node^{res1}_{xyz}}$
  $\mathrm{XYZ_1} \leftarrow \mathrm{PredGeometry_1}(\mathrm{Node_{xyz}}, \mathrm{Edge_{xyz}}, \mathrm{Index_{edge}})$
  $\overrightarrow{\mathrm{XYZ_1}} \leftarrow \mathrm{VectorDiff}(\mathrm{XYZ_1}, \mathrm{Index_{edge}})$
  $\mathrm{DistanceMap_1} \leftarrow \|\overrightarrow{\mathrm{XYZ_1}}\|_2^2$
  $\mathrm{ConcatMap_1} \leftarrow \mathrm{Distance2Concat}(\mathrm{DistanceMap_1})$
  $\mathrm{Edge_{xyz}} \leftarrow \mathrm{MLP}\left([\mathrm{Edge_{xyz}} \,\|\, \mathrm{DistanceMap_1} \,\|\, \mathrm{ConcatMap_1}]\right)$
  $\mathrm{Edge_{xyz}} \leftarrow \mathrm{Edge_{xyz}} + \mathrm{Edge^{res}_{xyz}}$
  # Stage-2: Predict $\Delta\mathrm{XYZ}$
  $\mathrm{Node^{res2}_{xyz}} \leftarrow \mathrm{Node_{xyz}}$
  $\mathrm{Edge^{res}_{xyz}} \leftarrow \mathrm{Edge_{xyz}}$
  $\mathrm{Node_{xyz}},\ \mathrm{Edge_{xyz}} \leftarrow \mathrm{NEE_2}(\mathrm{Node_{xyz}}, \mathrm{Edge_{xyz}}, \mathrm{Index_{edge}})$
  $\mathrm{Node_{xyz}} \leftarrow \mathrm{Node_{xyz}} + \mathrm{Node^{res1}_{xyz}} + \mathrm{Node^{res2}_{xyz}}$
  $\Delta\mathrm{XYZ} \leftarrow \mathrm{PredGeometry_2}(\mathrm{Node_{xyz}}, \mathrm{Edge_{xyz}}, \mathrm{Index_{edge}})$
  # Stage-3: Predict XYZ_2
  $\mathrm{XYZ_2} \leftarrow \mathrm{XYZ_1} + \Delta\mathrm{XYZ}$
  $\overrightarrow{\mathrm{XYZ_2}} \leftarrow \mathrm{VectorDiff}(\mathrm{XYZ_2}, \mathrm{Index_{edge}})$
  $\mathrm{DistanceMap_2} \leftarrow \|\overrightarrow{\mathrm{XZY_2}}\|_2^2$
  $\mathrm{ConcatMap_2} \leftarrow \mathrm{Distance2Concat}(\mathrm{DistanceMap_2})$
  $\mathrm{Edge_{xyz}} \leftarrow \mathrm{MLP}\left([\mathrm{Edge_{xyz}} \,\|\, \mathrm{DistanceMap_2} \,\|\, \mathrm{ConcatMap_2}]\right)$
  $\mathrm{Edge_{xyz}} \leftarrow \mathrm{Edge_{xyz}} + \mathrm{Edge^{res}_{xyz}}$
  # Output
  return $\mathrm{XYZ_1}, \mathrm{XYZ_2}, \mathrm{Node_{xyz}}, \mathrm{Edge_{xyz}}$

Algorithm 13: Distance2Concat

Input: DistanceMap

Output: ConcatMap

def Distance2Concat(*DistanceMap*)

$\quad \text{CUTOFF} \leftarrow [0.01, 0.05, 0.1, 0.12, 0.3, 0.5, 0.8, 1.0, 2.0, 3.0, 5.0, 8.0]$

$\quad \text{TileNumber} \leftarrow \text{Length}(\text{CUTOFF})$

$\quad \text{DistanceMap}_{\text{Array}} \leftarrow \text{Tile}(\text{DistanceMap}, \text{TileNumber})$

$\quad \text{ConcatMap} \leftarrow \text{TrueOrFalse}(\text{DistanceMap} < \text{CUTOFF})$

$\quad$ **return** ConcatMap

Algorithm 14: PredGeometry

Input: $\mathrm{Node_{xyz}}, \mathrm{Edge_{xyz}}, \mathrm{Index_{edge}}$

Output: XYZ

```
def PredGeometry(Node_xyz, Edge_xyz, Index_edge)
    # Projection
    node ← MLP(Node_xyz)
    edge ← MLP(Edge_xyz)
    # Message Passing
    Message_ij ← MLP(node_i + edge_ij) for (i, j)=Index_edge
    # Aggregation
    α_ij ← SoftMax(Message_ij)
    Z ← Σ_{j∈N(i)} α_ij Message_ij
    # Residual
    node ← node + Z
    # Output
    XYZ ← MLP(node)
    return XYZ
```

Algorithm 15: VectorDiff

Input: XYZ

Output: $\overrightarrow{\mathrm{XYZ}}$

```
def VectorDiff(XYZ, Index_edge)
    Index_source, Index_target ← Index_edge
    →XYZ ← XYZ[Index_target] − XYZ[Index_source]
    return →XYZ
```

Algorithm 16: NEE

Input: $\mathrm{Node}_{\mathrm{xyz}}$, $\mathrm{Edge}_{\mathrm{xyz}}$

Output: $\mathrm{Node}_{\mathrm{xyz}}$, $\mathrm{Edge}_{\mathrm{xyz}}$

def NEE($\mathrm{Node}_{\mathrm{xyz}}$, $\mathrm{Edge}_{\mathrm{xyz}}$, $\mathrm{Index}_{\mathrm{edge}}$)

  $\mathrm{Index}_{\mathrm{source}}, \mathrm{Index}_{\mathrm{target}} \leftarrow \mathrm{Index}_{\mathrm{edge}}$

  # Edge update

  $\mathrm{Diff\_Info} \leftarrow \mathrm{Node2Node\_Diff}(\mathrm{Node}, \mathrm{Index}_{\mathrm{edge}})$

  $\mathrm{Edge}_{\mathrm{ij}} \leftarrow \mathrm{MLP}([\mathrm{Node}_{\mathrm{i}} \,\|\, \mathrm{Node}_{\mathrm{j}} \,\|\, \mathrm{Diff\_Info} \,\|\, \mathrm{Edge}_{\mathrm{ij}}])$

  $\mathrm{Edge} \leftarrow \mathrm{MLP}(\mathrm{Edge}_{\mathrm{ij}})$

  # Node update

  $\mathrm{Edge}_{\mathrm{Agg}} \leftarrow \mathrm{scatter\_add\_}(\mathrm{Edge}_{\mathrm{ij}},\ \mathrm{Index}_{\mathrm{source}}, \mathrm{Length}_{\mathrm{Molecule}})$

  $\mathrm{Node} \leftarrow \mathrm{MLP}([\mathrm{Node} \,\|\, \mathrm{Edge}_{\mathrm{Agg}}])$

  $\mathrm{Node} \leftarrow \mathrm{MLP}(\mathrm{Node})$

  return $\mathrm{Node}, \mathrm{Edge}$

Algorithm 17: Node2Node_Diff

Input: $\mathrm{Node_{xyz}}$, $\mathrm{Edge_{xyz}}$

Output: Diff_Info

def Node2Node_Diff($\mathrm{Node_{xyz}}$, $\mathrm{Edge_{xyz}}$, $\mathrm{Index_{edge}}$)

  $\mathrm{Index_{source}}, \mathrm{Index_{target}} \leftarrow \mathrm{Index_{edge}}$

  # Vector

  $\Delta\mathrm{Vector} \leftarrow \mathrm{Node}[\mathrm{Index_{target}}] - \mathrm{Node}[\mathrm{Index_{source}}]$

  # Distance

  $\Delta\mathrm{Distance} \leftarrow \|\Delta\mathrm{Vector}\|_2$

  # Cosine

  $\Delta\mathrm{CosSim} \leftarrow \mathrm{CosineSimilarity}(\mathrm{Node}[\mathrm{Index_{target}}], \mathrm{Node}[\mathrm{Index_{source}}])$

  # Concat

  $\mathrm{Diff\_Info} \leftarrow [\Delta\mathrm{Vector} \| \, \Delta\mathrm{Distance} \| \, \Delta\mathrm{CosSim}]$

  return Diff_Info

5.2 Loss function for model training

Algorithm 18: Total Loss

Input: $\text{Molecule}_{\text{Predicted}}, \text{Molecule}_{\text{Original}}$

Output: Total Loss

def compute_Total_Loss($\text{Molecule}_{\text{Predicted}}, \text{Molecule}_{\text{Original}}$, Mask_Index, Replace_Index)

    # Prepare
    $\text{Mask\_Index}_{\text{global}}, \text{Mask\_Index}_{\text{node}}, \text{Mask\_Index}_{\text{edge}} \leftarrow \text{Mask\_Index}$
    $\text{Global}_{\text{pred}}, \text{Node}_{\text{pred}}, \text{Edge}_{\text{pred}}, \text{XYZ}_{\text{pred}} \leftarrow \text{Molecule}_{\text{Predicted}}$
    $\text{Global}_{\text{label}}, \text{Node}_{\text{label}}, \text{Edge}_{\text{label}}, \text{XYZ}_{\text{label}} \leftarrow \text{Molecule}_{\text{Original}}$
    $\text{Replace\_Index}_{\text{node}}, \text{Replace\_Index}_{\text{edge}} \leftarrow \text{Replace\_Index}$
    # Global Loss
    $\text{Loss}_{\text{global}} \leftarrow \text{compute\_Global\_Loss}(\text{Global}_{\text{pred}}, \text{Global}_{\text{label}}, \text{Mask\_Index}_{\text{global}})$
    # Node Loss
    $\text{Loss}_{\text{node}} \leftarrow \text{compute\_Node\_Loss}(\text{Node\_pred}, \text{Node\_label}, \text{Mask\_Index}_{\text{node}}, \text{Replace\_Index}_{\text{node}})$
    # Edge Loss
    $\text{Loss}_{\text{edge}} \leftarrow \text{compute\_Edge\_Loss}(\text{Edge\_pred}, \text{Edge\_label}, \text{Mask\_Index}_{\text{edge}}, \text{Replace\_Index}_{\text{edge}})$
    # Geometry Loss
    $\text{Loss}_{\text{geometry}} \leftarrow \text{compute\_Geometry\_Loss}(\text{XYZ}_{\text{pred}}, \text{XYZ}_{\text{label}})$
    # Total Loss
    $\text{Loss}_{\text{total}} \leftarrow \text{Loss}_{\text{global}} + \text{Loss}_{\text{node}} + \text{Loss}_{\text{edge}} + \text{Loss}_{\text{geometry}}$
    return $\text{Loss}_{\text{total}}$

Algorithm 19: Global Loss

Input: Predicted Global Information, Original Global Information

Output: Gloabl Loss

def compute_Global_Loss($\text{Global}_{\text{pred}}, \text{Global}_{\text{label}}, \text{Mask\_Index}_{\text{global}}$)

    # Prepare

    $\text{Global}^{\text{reg}}_{\text{pred}}, \text{Global}^{\text{cls}}_{\text{pred}} \leftarrow \text{Global}_{\text{pred}}[\text{Mask\_Index}_{\text{global}}]$

    $\text{Global}^{\text{reg}}_{\text{label}}, \text{Global}^{\text{cls}}_{\text{label}} \leftarrow \text{Global}_{\text{label}}[\text{Mask\_Index}_{\text{global}}]$

    # Regression Loss

    $\text{Loss}^{\text{reg}}_{\text{global}} \leftarrow \text{MAE}(\text{Global}^{\text{reg}}_{\text{pred}}, \text{Global}^{\text{reg}}_{\text{label}})$

    # Classification Loss

    $\text{Loss}^{\text{cls}}_{\text{global}} \leftarrow \text{CE}(\text{Global}^{\text{cls}}_{\text{pred}}, \text{Global}^{\text{cls}}_{\text{label}})$

    # Global Loss

    $\text{Loss}_{\text{global}} \leftarrow \text{Loss}^{\text{reg}}_{\text{global}} + \text{Loss}^{\text{cls}}_{\text{global}}$

    return $\text{Loss}_{\text{global}}$

Algorithm 20: Node Loss

Input: Predicted Node Features, Original Node Features

Output: Node Loss

def compute_Node_Loss($\mathrm{Node_{pred}}, \mathrm{Node_{label}}, \mathrm{Mask\_Index_{node}}, \mathrm{Replace\_Index_{node}}$)

  # Prepare

  $\mathrm{Node_{pred}^{reg}}, \mathrm{Node_{pred}^{cls}} \leftarrow \mathrm{Node_{pred}}[\mathrm{Mask\_Index_{node}} \| \mathrm{Replace\_Index_{node}}]$

  $\mathrm{Node_{label}^{reg}}, \mathrm{Node_{label}^{cls}} \leftarrow \mathrm{Node_{label}}[\mathrm{Mask\_Index_{node}} \| \mathrm{Replace\_Index_{node}}]$

  # Regression Loss

  $\mathrm{Loss_{node}^{reg}} \leftarrow \mathrm{MAE}(\mathrm{Node_{pred}^{reg}}, \mathrm{Node_{label}^{reg}})$

  # Classification Loss

  $\mathrm{Loss_{node}^{cls}} \leftarrow \mathrm{CE}(\mathrm{Node_{pred}^{cls}}, \mathrm{Node_{label}^{cls}})$

  # Node Loss

  $\mathrm{Loss_{node}} \leftarrow \mathrm{Loss_{node}^{reg}} + \mathrm{Loss_{node}^{cls}}$

  return $\mathrm{Loss_{node}}$

Algorithm 21: Edge Loss

Input: Predicted Edge Features, Original Edge Features

Output: Edge Loss

def compute_Edge_Loss($\mathrm{Edge}_{\mathrm{pred}}, \mathrm{Edge}_{\mathrm{label}}, \mathrm{Mask\_Index}_{\mathrm{edge}}, \mathrm{Replace\_Index}_{\mathrm{edge}}$)

    # Prepare

    $\mathrm{Edge}^{\mathrm{reg}}_{\mathrm{pred}}, \mathrm{Edge}^{\mathrm{cls}}_{\mathrm{pred}} \leftarrow \mathrm{Edge}_{\mathrm{pred}}[\mathrm{Mask\_Index}_{\mathrm{edge}} \| \ \mathrm{Replace\_Index}_{\mathrm{edge}}]$

    $\mathrm{Edge}^{\mathrm{reg}}_{\mathrm{label}}, \mathrm{Edge}^{\mathrm{cls}}_{\mathrm{label}} \leftarrow \mathrm{Edge}_{\mathrm{label}}[\mathrm{Mask\_Index}_{\mathrm{edge}} \| \ \mathrm{Replace\_Index}_{\mathrm{edge}}]$

    # Regression Loss

    $\mathrm{Loss}^{\mathrm{reg}}_{\mathrm{edge}} \leftarrow \mathrm{MAE}(\mathrm{Edge}^{\mathrm{reg}}_{\mathrm{pred}}, \mathrm{Edge}^{\mathrm{reg}}_{\mathrm{label}})$

    # Classification Loss

    $\mathrm{Loss}^{\mathrm{cls}}_{\mathrm{edge}} \leftarrow \mathrm{CE}(\mathrm{Edge}^{\mathrm{cls}}_{\mathrm{pred}}, \mathrm{Edge}^{\mathrm{cls}}_{\mathrm{label}})$

    # Edge Loss

    $\mathrm{Loss}_{\mathrm{edge}} \leftarrow \mathrm{Loss}^{\mathrm{reg}}_{\mathrm{edge}} + \mathrm{Loss}^{\mathrm{cls}}_{\mathrm{edge}}$

    return $\mathrm{Loss}_{\mathrm{edge}}$

Algorithm 22: Geometry Loss

Input: Denoised Geometry, Original Geometry

Output: Geometry Loss

def compute_Geometry_Loss($\mathrm{XYZ_{denoised}}, \mathrm{XYZ_{label}}$)

  # Prepare

  $\mathrm{Number} = \mathrm{Length}(3XYZ_{denoised})$

  $\mathrm{XYZ_{label}} \leftarrow \mathrm{Tile}(\mathrm{XYZ_{label}}, \mathrm{Number})$

  $\mathrm{XYZ_{denoised}} \leftarrow \mathrm{TranslationRotation}(\mathrm{XYZ_{denoised}}, \mathrm{XYZ_{label}})$

  $\mathrm{r_i^{denoised}} \in \mathrm{XYZ_{denoised}},\ \mathrm{r_i^{label}} \in \mathrm{XYZ_{label}}$

  # RMSD

  $\mathrm{Loss_{RMSD}} \leftarrow \sqrt{\frac{1}{|\mathrm{N}|} \sum_{\mathrm{i} \in \mathrm{N}} \|\mathrm{r_i^{denoised}} - \mathrm{r_i^{label}}\|_2}$

  # Vector Difference

  $\Delta\mathrm{XYZ_{denoised}} \leftarrow \mathrm{r_i^{denoised}} - \mathrm{r_j^{denoised}}, \mathrm{j} \in \mathrm{Neighbor\ of\ i}$

  $\Delta\mathrm{XYZ_{label}} \leftarrow \mathrm{r_i^{label}} - \mathrm{r_j^{label}}, \mathrm{j} \in \mathrm{Neighbor\ of\ i}$

  $\mathrm{VectorDiff} \leftarrow \Delta\mathrm{XYZ_{denoised}} - \Delta\mathrm{XYZ_{label}}$

  $\mathrm{Loss_{PV}} \leftarrow \frac{1}{|\mathrm{N}|^2} \sum_{i \in \mathrm{N}} \sum_{j \in \mathrm{N}} \mathrm{VectorDiff_{ij}}$

  # Distance Difference

  $\mathrm{DistanceDiff} \leftarrow \|\Delta\mathrm{XYZ_{denoised}}\|_2 - \|\Delta\mathrm{XYZ_{label}}\|_2$

  $\mathrm{Loss_{PD}} \leftarrow \frac{1}{|\mathrm{N}|^2} \sum_{i \in \mathrm{N}} \sum_{j \in \mathrm{N}} \mathrm{DistanceDiff_{ij}}$

  # FAPE

  $\mathrm{Loss_{FAPE}} \leftarrow$ compute_FAPE($\mathrm{XYZ_{denoised}}, \mathrm{XYZ_{label}}$)

  # Geometry Loss

  $\mathrm{Loss_{geometry}} \leftarrow \mathrm{Loss_{FAPE}} + \mathrm{Loss_{RMSD}} + \mathrm{Loss_{PD}} + \mathrm{Loss_{PV}}$

  return $\mathrm{Loss_{geometry}}$

Algorithm 23: FAPE Loss

Input: Denoised Geometry, Original Geometry

Output: FAPE Loss

def compute_FAPE($\mathrm{XYZ}_{\mathrm{denoised}}, \mathrm{XYZ}_{\mathrm{label}}$)
  for Frame $\in$ RigidFramesList **do**
    $\mathrm{XYZ}_{\mathrm{denoised}} \leftarrow \mathrm{TranslationRotation}(\mathrm{XYZ}_{\mathrm{denoised}}, \mathrm{Frame})$
    $\mathrm{XYZ}_{\mathrm{label}} \leftarrow \mathrm{TranslationRotation}(\mathrm{XYZ}_{\mathrm{label}}, \mathrm{Frame})$
    $\mathrm{Loss}_{\mathrm{FAPE}} \leftarrow \|\mathrm{XYZ}_{\mathrm{denoised}} - \mathrm{XYZ}_{\mathrm{label}}\|_2$
    $\mathrm{Loss}_{\mathrm{FAPE}} = \mathrm{Clip}(\mathrm{Loss}_{\mathrm{FAPE}}, 10)$
  end
  $\mathrm{Loss}_{\mathrm{FAPE}} = \frac{1}{|3\mathrm{N}|} \sum_{x,y,z} \sum_{i \in N} \mathrm{Loss}_{\mathrm{FAPE}}$
  return $\mathrm{Loss}_{\mathrm{FAPE}}$

Supplementary References

1. Rogers, D. & Hahn, M. Extended-connectivity fingerprints. *Journal of Chemical Information and Modeling* **50,** 742–754. ISSN: 1549-9596. https://pubs.acs.org/doi/abs/10.1021/ci100050t (2010).
2. Gedeck, P., Rohde, B. & Bartels, C. QSAR − How Good Is It in Practice? Comparison of Descriptor Sets on an Unbiased Cross Section of Corporate Data Sets. *Journal of Chemical Information and Modeling* **46.** PMID: 16995723, 1924–1936. eprint: https://doi.org/10.1021/ci050413p. https://doi.org/10.1021/ci050413p (2006).
3. Nilakantan, R., Bauman, N., Dixon, J. S., *et al.* Topological torsion: a new molecular descriptor for SAR applications. Comparison with other descriptors. *Journal of Chemical Information and Computer Sciences* **27,** 82–85. https://pubs.acs.org/doi/pdf/10.1021/ci00054a008 (1987).
4. Carhart, R. E., Smith, D. H. & Venkataraghavan, R. Atom pairs as molecular features in structure-activity studies: definition and applications. *Journal of Chemical Information and Computer Sciences* **25,** 64–73. https://pubs.acs.org/doi/pdf/10.1021/ci00046a002 (1985).
5. Landrum, G., Tosco, P., Kelley, B., *et al. RDKit: 2025_03_2 (Q1 2025) Release* 2025. https://doi.org/10.5281/zenodo.15286010.
6. Durant, J. L., Leland, B. A., Henry, D. R., *et al.* Reoptimization of MDL keys for use in drug discovery. *Journal of Chemical Information and Computer Sciences* **42,** 1273–1280. https://pubs.acs.org/doi/10.1021/ci010132r (2002).
7. Ross, J., Belgodere, B., Chenthamarakshan, V., *et al.* Large-scale chemical language representations capture molecular structure and properties. *Nature Machine Intelligence* **4,** 1256–1264. https://www.nature.com/articles/s42256-022-00580-7 (2022).
8. Rong, Y., Bian, Y., Xu, T., *et al.* Self-Supervised Graph Transformer on Large-Scale Molecular Data. In *Advances in Neural Information Processing Systems (NeurIPS)* **33,** 12559–12571 (2020). https://dl.acm.org/doi/abs/10.5555/3495724.3496777.
9. Xia, J., Zhao, C., Hu, B., *et al.* Mole-BERT: Rethinking Pre-training Graph Neural Networks for Molecules. In *Proceedings of the Eleventh International Conference on Learning Representations (ICLR)* (2023). https://openreview.net/forum?id=jevY-DtiZTR.
10. Zhou, G., Gao, Z., Ding, Q., *et al.* Uni-Mol: A Universal 3D Molecular Representation Learning Framework. In *Proceedings of the Eleventh International Conference on Learning Representations (ICLR)* (2023). https://openreview.net/forum?id=6K2RM6wVqKu.
11. Qiu, J., Chen, Q., Dong, Y., *et al.* GCC: Graph Contrastive Coding for Graph Neural Network Pre-Training. In *Proceedings of the 26th ACM SIGKDD International Conference on Knowledge Discovery and Data Mining (KDD)* 1150–1160 (2020). https://dl.acm.org/doi/10.1145/3394486.3403168.
12. Hu, Z., Dong, Y., Wang, K., *et al.* GPT-GNN: Generative Pre-Training of Graph Neural Networks. In *Proceedings of the 26th ACM SIGKDD International Conference on Knowledge Discovery and Data Mining (KDD)* 1857–1867 (2020). https://dl.acm.org/doi/abs/10.1145/3394486.3403237.
13. You, Y., Chen, T., Sui, Y., *et al.* Graph Contrastive Learning with Augmentations. In *Advances in Neural Information Processing Systems* (eds Larochelle, H., Ranzato, M., Hadsell, R., *et al.*) **33,** 5812–5823 (Curran Associates, Inc., 2020). https://proceedings.neurips.cc/paper/2020/file/3fe230348e9a12c13120749e3f9fa4cd-Paper.pdf.

14. Sun, F.-Y., Hoffman, J., Verma, V., *et al.* InfoGraph: Unsupervised and Semi-supervised Graph-Level Representation Learning via Mutual Information Maximization. In *International Conference on Learning Representations* (2020). https://openreview.net/forum?id=r1lfF2NYvH.
15. You, Y., Chen, T., Shen, Y., *et al.* Graph Contrastive Learning Automated. In *Proceedings of the 38th International Conference on Machine Learning (ICML)* 12121–12132 (2021). https://proceedings.mlr.press/v139/you21a.html.
16. Jiao, R., Han, J., Huang, W., *et al.* Energy-Motivated Equivariant Pretraining for 3D Molecular Graphs. *Proceedings of the AAAI Conference on Artificial Intelligence* **37,** 8096–8104. https://ojs.aaai.org/index.php/AAAI/article/view/25978 (2023).
17. Stärk, H., Beaini, D., Corso, G., *et al.* 3D Infomax improves GNNs for Molecular Property Prediction. In *Proceedings of the NeurIPS 2021 AI for Science Workshop* (2021). https://openreview.net/forum?id=8K23ZyTIKuF.
18. Hu, W., Liu, B., Gomes, J., *et al.* Strategies for Pre-training Graph Neural Networks. In *Proceedings of the Eleventh International Conference on Learning Representations (ICLR)* (2020). https://openreview.net/forum?id=HJlWWJSFDH.
19. Hamilton, W. L., Ying, R. & Leskovec, J. Inductive representation learning on large graphs. In *Proceedings of the 31st International Conference on Neural Information Processing Systems (NeurIPS)* 1025–1035 (2017). https://dl.acm.org/doi/10.5555/3294771.3294869.
20. Liu, S., Wang, H., Liu, W., *et al.* Pre-training Molecular Graph Representation with 3D Geometry. In *International Conference on Learning Representations* (2022). https://openreview.net/forum?id=xQUe1pOKPam.
21. Wang, Y., Wang, J., Cao, Z., *et al.* Molecular contrastive learning of representations via graph neural networks. *Nature Machine Intelligence* **4,** 279–287. ISSN: 2522-5839. https://doi.org/10.1038/s42256-022-00447-x (2022).
22. Luo, S., Chen, T., Xu, Y., *et al.* One Transformer Can Understand Both 2D & 3D Molecular Data. In *The Eleventh International Conference on Learning Representations* (2023). https://openreview.net/forum?id=vZTp1oPV3PC.
23. Li, P., Wang, J., Qiao, Y., *et al.* An effective self-supervised framework for learning expressive molecular global representations to drug discovery. *Briefings in Bioinformatics* **22,** bbab109. ISSN: 1467-5463. https://doi.org/10.1093/bib/bbab109 (2021).
24. Fang, Y., Zhang, Q., Zhang, N., *et al.* Knowledge graph-enhanced molecular contrastive learning with functional prompt. *Nature Machine Intelligence* **5,** 542–553. ISSN: 2522-5839. https://doi.org/10.1038/s42256-023-00654-0 (2023).
25. Wu, Z., Ramsundar, B., Feinberg, E. N., *et al.* MoleculeNet: a benchmark for molecular machine learning. *Chemical Science* **9,** 513–530. ISSN: 2041-6520. http://dx.doi.org/10.1039/C7SC02664A (2018).
26. Huang, K., Fu, T., Gao, W., *et al.* Therapeutics Data Commons: Machine Learning Datasets and Tasks for Drug Discovery and Development. In *Advances in Neural Information Processing Systems (NeurIPS) Datasets and Benchmarks Track* **35** (2021). https://openreview.net/forum?id=8nvgnORnoWr.
27. Van Tilborg, D., Alenicheva, A. & Grisoni, F. Exposing the Limitations of Molecular Machine Learning with Activity Cliffs. *Journal of Chemical Information and Modeling* **62,** 5938–5951. ISSN: 1549-9596. https://doi.org/10.1021/acs.jcim.2c01073 (2022).

28. Axelrod, S. & Gómez-Bombarelli, R. GEOM, energy-annotated molecular conformations for property prediction and molecular generation. *Scientific Data* **9,** 185. ISSN: 2052-4463. https://doi.org/10.1038/s41597-022-01288-4 (2022).

29. Li, H., Zhang, R., Min, Y., *et al.* A Knowledge-Guided Pre-Training Framework for Improving Molecular Representation Learning. *Nature Communications* **14,** 7568. https://doi.org/10.1038/s41467-023-43214-1 (2023).

30. Cheng, Z., Xiang, H., Ma, P., *et al.* MaskMol: Knowledge-Guided Molecular Image Pre-Training Framework for Activity Cliffs with Pixel Masking. *BMC Biology* **23,** 279. https://doi.org/10.1186/s12915-025-02389-3 (2025).

31. Maggiora, G. M. On Outliers and Activity Cliffs—Why QSAR Often Disappoints. *Journal of Chemical Information and Modeling* **46,** 1535. https://doi.org/10.1021/ci060117s (2006).

32. Guha, R. & Van Drie, J. H. Structure–Activity Landscape Index: Identifying and Quantifying Activity Cliffs. *Journal of Chemical Information and Modeling* **48,** 646–658. https://doi.org/10.1021/ci7004093 (2008).

33. Stumpfe, D., Hu, H. & Bajorath, J. Advances in Exploring Activity Cliffs. *Journal of Computer-Aided Molecular Design* **34,** 929–942. https://doi.org/10.1007/s10822-020-00315-z (2020).

34. Bemis, G. W. & Murcko, M. A. The Properties of Known Drugs. 1. Molecular Frameworks. *Journal of Medicinal Chemistry* **39,** 2887–2893. https://doi.org/10.1021/jm9602928 (1996).

35. Fawcett, T. An Introduction to ROC Analysis. *Pattern Recognition Letters* **27,** 861–874. https://doi.org/10.1016/j.patrec.2005.10.010 (2006).

36. Davis, J. & Goadrich, M. The Relationship between Precision–Recall and ROC Curves. In *Proceedings of the 23rd International Conference on Machine Learning* 233–240 (Association for Computing Machinery, 2006). https://doi.org/10.1145/1143844.1143874.

37. Saito, T. & Rehmsmeier, M. The Precision–Recall Plot Is More Informative than the ROC Plot When Evaluating Binary Classifiers on Imbalanced Datasets. *PLOS ONE* **10,** e0118432. https://doi.org/10.1371/journal.pone.0118432 (2015).
